# Intramolecular Interactions between the Pnictogen Groups in a Rigid Ferrocene Phosphinostibine and the Corresponding Phosphine Chalcogenides, Stiboranes, and Their Complexes

**DOI:** 10.1021/acs.inorgchem.5c01332

**Published:** 2025-05-25

**Authors:** David Rezazgui, Jiří Schulz, Petr Štěpnička

**Affiliations:** Department of Inorganic Chemistry, Faculty of Science, 112302 Charles University, Hlavova 2030, Prague 128 00, Czech Republic

## Abstract

Differences in the chemical properties of phosphorus and antimony enable the synthesis of heteroditopic derivatives whose properties can be modified by altering the pnictogen substituents. In this work, 1-(diphenylstibino)-2-(dicyclohexylphosphino)­ferrocene, [Fe­(η^5^-1-Ph_2_Sb-2-Cy_2_PC_5_H_3_)­(η^5^-C_5_H_5_)] (**1**), and the corresponding phosphine chalcogenides [Fe­(η^5^-1-Ph_2_Sb-2-Cy_2_P­(E)­C_5_H_3_)­(η^5^-C_5_H_5_)] (E = O, S, Se) and catecholatostiboranes [Fe­(η^5^-1-Ph_2_(Cl_4_C_6_O_2_)­Sb-2-Cy_2_P­(E)­C_5_H_3_)­(η^5^-C_5_H_5_)] (E = void, O, S, Se) were examined, with a focus on the intramolecular donor–acceptor interactions between the antimony and the phosphorus substituents. Experimental data and theoretical analysis consistently indicated that these interactions can be described as pnictogen bonding between the Lewis acidic antimony and the lone pair at the phosphorus substituent (either at the phosphorus or at the chalcogen atom) and that they are significantly stronger in the stiboranes due to the increased Lewis acidity of the Sb atom. Noncovalent interactions were also observed in the chlorogold­(I) complexes obtained from **1** and catecholatostiborane [Fe­(η^5^-1-Ph_2_(Cl_4_C_6_O_2_)­Sb-2-Cy_2_PC_5_H_3_)­(η^5^-C_5_H_5_)] as P-donors. As shown by experiments in Au-mediated cyclization of *N*-propargylbenzamide, the noncoordinated antimony group influenced the catalytic properties of the Au­(I) complexes. Notably, an intramolecular Cl → Sb pnictogen bond affected the molecular geometry of the Pd­(II) complex [PdCl_2_(**1**-κ^2^ *P*,*Sb*)], which in turn suggested that the structural influence exerted by ligands of this type needs to be assessed with care.

## Graphical abstract

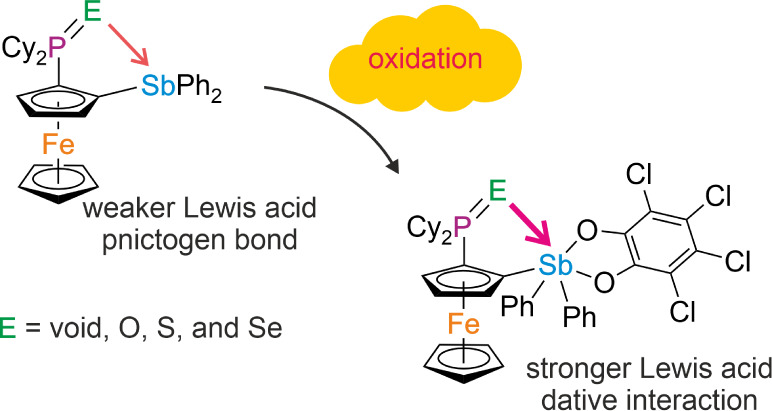

## Introduction

The search for trends and analogies in the periodic system is an indispensable tool for chemistry research and education.^1^ However, while searching for trends and analogies, caution should be exercised to avoid overgeneralizations, as it happened in the case of stibines, which have long been considered mere analogs of the ubiquitous phosphines.^2^ Detailed studies subsequently revealed substantial differences in the ligating properties and reactivities of these two compound classes. Compared with analogous phosphines, stibines are generally worse σ-donors with lower steric demands.^3,4^ At the same time, they exhibit enhanced Lewis acidity,^5^ which is retained even after coordination to a metal center and can be increased by oxidation to the Sb­(V) derivatives, whose chemistry also differs from that of their phosphorus congeners.^6,7^ In this regard, compounds that combine phosphine and stibine donor groups have emerged as attractive hybrid ligands,^8,9^ mainly because of the possibility of direct comparison between the two pnictogen groups and their independent modification and exploitation. Representative examples of such compounds are phosphinostibines **A** ^10^ and **B**,^11^ and their multidonor analogs **C** and **D** ^12,13^ (Scheme 1).

**Scheme 1:**
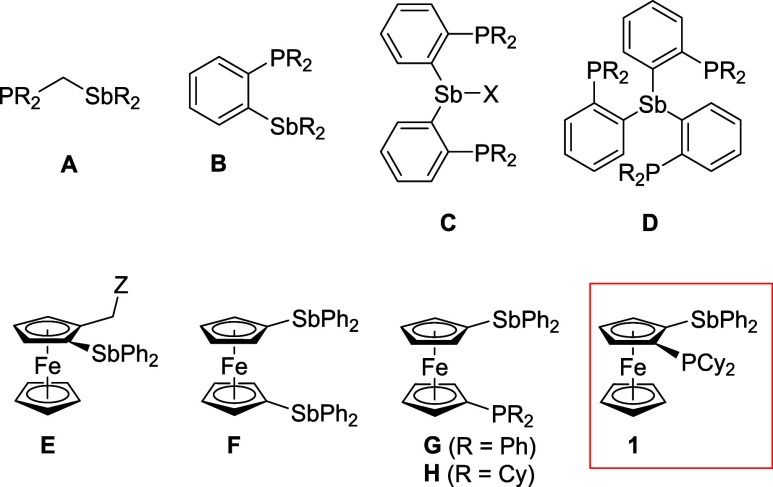
Representative Phosphinostibine Donors, Ferrocene Stibines Studied Previously, and the Structure of **1**, which Is the Parent Compound of the Present Study^a^

a X = Cl, R; Z = NMe_2_, NHR, OH, OR, SR, *etc*.; R = various hydrocarbyl substituents, Cy = cyclohexyl.

Stibine donors have long been rare among ferrocene ligands,^14^ which starkly contrasts with the wide chemistry of ferrocene phosphines.^14a,15^ Early reports focused on compounds of type **E** (Scheme 1) and hypervalent interactions between the stibine group and the additional Lewis donor in these molecules.^16,17^ Fragmentary information on ferrocene stibines led us to focus on this class of compounds. Thus, far, we have reported on the synthesis, reactivity, and coordination behavior of 1,1′-bis­(diphenylstibino)­ferrocene (**F**),^18^ which is an antimony analog of the widely studied and practically utilized 1,1′-bis­(diphenylphosphino)­ferrocene (dppf).^19,20^ In subsequent work, we concentrated on the related, donor-unsymmetric compounds **G** ^21^ and **H**.^22^ The former compound enabled the demonstration of the differences in the reactivity of the pnictine substituents and the preparation of a series of P­(III)/Sb­(V) and P­(V)/Sb­(V) derivatives showing donor–acceptor interactions between the functional groups.^21^ Similar behavior was demonstrated for the latter compound, which was also suitable for coordination studies that further illustrated the differences between the pnictogen groups.^22^

During these studies, a question arose on how the interactions between the phosphine and stibine groups (and their P­(V) and Sb­(V) counterparts) in the ferrocene P,Sb-derivatives could be influenced by changing the substitution pattern from the flexible 1,1′ arrangement^23^ to the rigid 1,2 geometry. To answer this question, a new family of compounds derived from phosphinostibine [Fe­(η^5^-1-Ph_2_Sb-2-Cy_2_PC_5_H_3_)­(η^5^-C_5_H_5_)], compound **1** (Scheme 1), was prepared and examined. The compounds were designed with different substituents at the pnictogen atoms to (1) increase the donor ability of the phosphine moiety through electron-donating cyclohexyl substituents and (2) reduce the tendency of the compounds to form disordered structures, as previously observed for the phenyl-substituted analog **G**.^21^ In this work, we describe the results from our detailed experimental and theoretical analysis of the intramolecular interactions between the P and Sb substituents in a series consisting of compound **1** and derivatives oxidized at the phosphorus and/or antimony atoms, namely [Fe­(η^5^-1-Ph_2_Sb-2-Cy_2_P­(E)­C_5_H_3_)­(η^5^-C_5_H_5_)] (E = BH_3_, O, S, Se) and the corresponding tetrachlorocatecholatostiboranes [Fe­(η^5^-1-Ph_2_(Cl_4_C_6_O_2_)­Sb-2-Cy_2_P­(E)­C_5_H_3_)­(η^5^-C_5_H_5_)] (E = void, BH_3_, O, S, Se). Furthermore, we describe the synthesis, characterization, and catalytic properties of Au­(I) complexes featuring phosphinostibine **1** and the related stiboranes as ligands and also report the preparation and structure of a Pd­(II) complex [PdCl_2_(**1**-κ^2^ *P*,*Sb*)] showing an *intra*molecular Sb···Cl pnictogen interaction.

## Results and Discussion

### Synthesis and Characterization

The synthesis of phosphinostibine **1** and its derivatives with oxidized phosphorus and antimony atoms is outlined in Scheme 2. In the first step, bromoferrocene was *ortho*-lithiated^24^ at approximately – 78 °C with lithium 2,2,6,6-tetramethylpiperidide (LiTMP), which was generated separately from *n*-butyllithium and 2,2,6,6-tetramethylpiperidine. The resulting lithio derivative was directly treated with chloro-dicyclohexylphosphine and then with borane-dimethyl sulfide to protect^25^ the phosphine moiety from unwanted oxidation during workup and storage. The yield of **2**·BH_3_ was 80%. This compound was lithiated again with *n*-butyllithium and reacted with chloro-diphenylstibine^26^ to produce air-stable, P-protected phosphinostibine **1**·BH_3_ in 20% yield. Attempts to improve the yield of this reaction failed despite numerous trials, likely because the sterically demanding phosphine-borane moiety hindered access to the adjacent position at the ferrocene unit. Conversely, removal of the protecting group was achieved in essentially quantitative yield by the action of 1,4-diazabicyclo[2.2.2]­octane (dabco)^27^ in THF at 60 °C. The direct preparation of **1** from 2-(dicyclohexylphosphino)-1-bromoferrocene (i.e., from deprotected **2**·BH_3_) was not successful. Although the desired compound was formed by standard lithiation^28^ with *n*-BuLi and quenching with ClSbPh_2_, it could not be efficiently isolated from the reaction mixture because of extensive decomposition (mainly oxidation of the phosphine group).

**Scheme 2:**
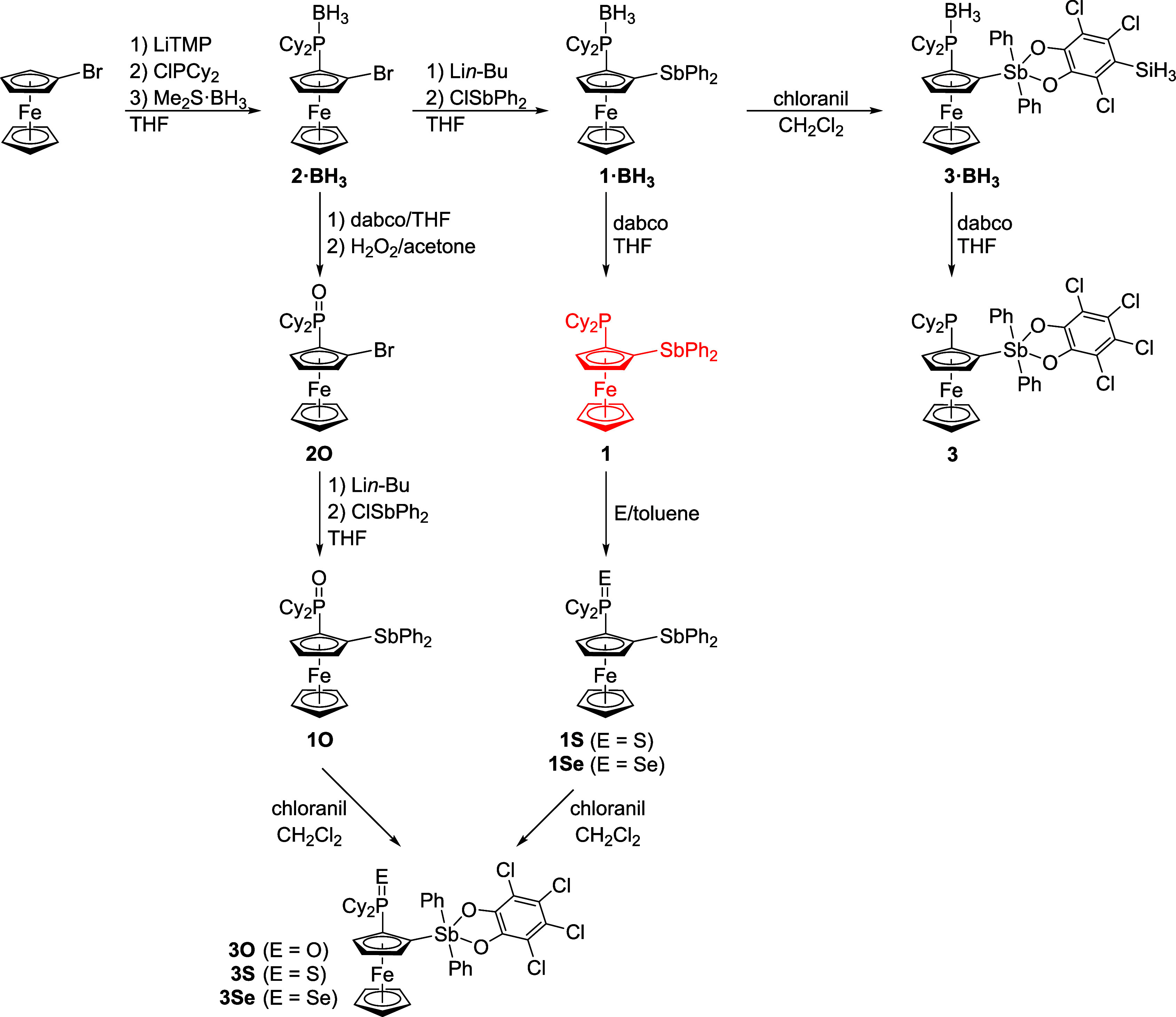
Synthesis of Phosphinostibine **1**, the Corresponding P-Chalcogenides **1E** and Stiboranes **3E** ^a^

a LiTMP = lithium 2,2,6,6-tetramethylpiperidide, dabco = 1,4-diazabicyclo[2.2.2]­octane, chloranil = 3,4,5,6-tetrachloro-1,2-benzoquinone.

The phosphinoyl derivative **1O** was prepared analogously by lithiation of phosphine oxide **2O** and quenching of the lithiated intermediate with ClSbPh_2_. This compound also formed during exposure of the solutions of **1** to air, but the oxidation was slow and incomplete. Stronger oxidants (*e.g*., H_2_O_2_) reacted nonselectively, producing products with oxidized Sb groups,^22,29^ and were therefore avoided. In contrast, compound **1** cleanly reacted with elemental sulfur and gray selenium in hot toluene to afford **1S** and **1Se**, respectively, in approximately 90% yields.

When treated with a stoichiometric amount of *o*-chloranil (3,4,5,6-tetrachloro-1,2-benzoquinone, 1 equiv),^30^ **1**·BH_3_ and all P-chalcogenides **1E** smoothly converted to the respective stiboranes **3**·BH_3_ and **3E** (≈80% yields Scheme 2). The direct oxidation of **1** with 1 mol equiv of *o*-chloranil unexpectedly produced zwitterionic compound **4**, which was formally the product of 2-fold oxidation and rearrangement leading to the formation of an Sb–O bond (Scheme 3). During this transformation, the coordination number of the P and Sb atoms was increased to four (sp^3^, tetrahedral) and six (sp^3^d^2^, octahedral), respectively. While the bridging catecholate unit represents a new structural motif,^31^ the formation of compound **4** corresponds with the tendency of catecholatostiboranes to increase their coordination number through the coordination of hard Lewis acids^32^ and with the relatively low stability of P–O bonds in catecholatophoshoranes, which easily hydrolyze into phosphine oxides and catechols.^33^

**Scheme 3:**
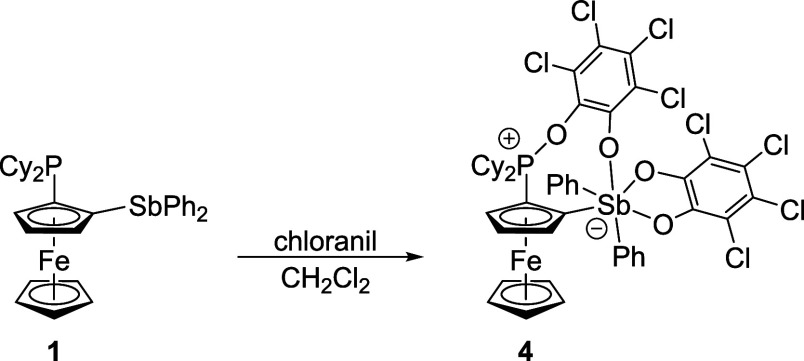
Direct Oxidation of **1** with *o*-Chloranil Producing Compound **4**

Eventually, compound **3** was obtained (Scheme 2) by the deprotection of the borane adduct **3**·BH_3_ with dabco. The relatively lower yield (32% isolated; *cf*. the preparation of **1**) reflected incomplete removal of the borane group during the reaction (THF, 60 °C, overnight) and the presence of impurities, which had to be removed by crystallization. In addition, the compound was prone to oxidation, which also decreased the yield.

All compounds were characterized using multinuclear NMR spectroscopy, mass spectrometry, and elemental analysis. The NMR spectra revealed all anticipated signals. The resonances due to the ferrocene moiety were observed as follows: a sharp singlet due to the unsubstituted cyclopentadienyl ring in both the ^1^H and ^13^C­{^1^H} NMR spectra and a set of three multiplets in the ^1^H NMR spectra and three CH plus two C^ipso^ resonances in the ^13^C­{^1^H} NMR spectra attributed to the 1,2-disubstituted ring. Characteristic signals were also observed for the substituents at the P and Sb atoms,^34^ including those of the 3,4,5,6-tetrachloro-1,2-benzenediolato fragments in **3**, **3**·BH_3_ and **3E**. Because the compounds were planar chiral (albeit racemic), the carbon atoms at the aromatic rings (phenyl and catecholate) became diastereotopic and, hence, anisochronic. This resulted in a doubling of the number of observed resonances.

The ^31^P­{^1^H} NMR chemical shifts (Table 1) determined for **1** and **1E** generally corresponded with those determined for the isomeric compounds **H** and Cy_2_P­(E)­fcSbPh_2_ (E = BH_3_, O, and S; fc = ferrocene-1,1′-diyl; Δδ_P_ ≤ 4.4 ppm in the pairs)^22^ and for FcP­(E)­Cy_2_ (Fc = ferrocenyl; E = void: δ_P_ –6.0; E = O: δ_P_ 46.8; in CDCl_3_).^35^ The oxidation of the stibine moiety with *o*-chloranil increased the electron-withdrawing ability of the Sb substituent. As a result, the ^31^P NMR signals of stiborane **3** were deshielded compared to the corresponding stibine **1** (Δδ_P_ = 34.3 ppm), and a similar change was observed for the signal of phosphine oxide **3O** (Δδ_P_ = 9.8 ppm). Whereas **3S** and **3Se** showed a similar trend (albeit less pronounced), the signal of **3**·BH_3_ was more shielded than that of stibine **1**·BH_3_ (Δδ_P_ = –9.0 ppm). The collected NMR data thus suggested significant interactions between the P and Sb substituents, at least in some compounds. However, the ultimate evidence was obtained from single-crystal X-ray diffraction analysis and DFT calculations (*vide infra*).

**Table 1:** ^31^P NMR Chemical Shifts of Compounds **1E** and **3E** (E = Void, BH_3_, O, S, Se)^a^

Compound	δ_P_	Compound	δ_P_
**1**	–7.9	**3**	26.4
**1**·BH_3_	28.7	**3**·BH_3_	19.7
**1O**	48.7	**3O**	58.5
**1S**	58.5	**3S**	58.6
**1Se**	51.9^b^	**3Se**	54.0^c^

a The ^31^P­{^1^H} NMR spectra were recorded in CDCl_3_ at 25 °C. Except for the borane adducts, the signals were observed as sharp singlets (with ^77^Se satellites for **1Se** and 3**Se**).

b ^1^ *J*_PSe_ = 697 Hz.

c ^1^ *J*_PSe_ = 599 Hz.

Notably, no signs of BH_3_ migration between the proximal phosphine and stibine substituents were observed during the experiments with **1**·BH_3_ and **3**·BH_3_ in both solution and solid-state. This behavior was in line with the relatively lower Lewis basicity of the stibine moiety and its further decrease upon conversion to stiborane. This trend was reflected by methyl cation affinities (MCAs; Table 2)^36^ calculated for **1**, **3**, phosphinoferocenes FcPR_2_ (R = Ph, Cy), and (diphenylstibino)­ferrocene (FcSbPh_2_). Defined as the enthalpy of [LB–CH_3_]^+^ bond dissociation, a larger MCA indicates a stronger Lewis base (LB). The estimated MCA values suggested the phosphine groups to be more Lewis basic than the stibine moieties both in vacuum and in chloroform, as expected. The MCA values calculated for individual substituents in **1** were only slightly greater (relatively <5%) than those for the respective monosubstituted ferrocenes, FcPCy_2_ and FcSbPh_2_. Besides, the MCA calculated for FcPCy_2_ was greater than that for FcPPh_2_, in agreement with the lower electron-withdrawing nature of the alkylphosphine substituent in the former compound. Furthermore, the oxidation of the stibine moiety, such as in **3**, caused a decrease in the MCA value of the phosphine group, indicating its lower basicity. This can be rationalized by the stronger electron-withdrawing properties of the adjacent stiborane moiety that decreased the electron-donating ability of the ferrocene unit and, consequently, made the phosphorus atom a weaker Lewis base. However, the value could also be influenced by the presence (in **3**) or absence (methylated **3**) of the P → Sb interactions, which affected the thermochemical properties. The MCA value calculated for **3** was similar to that of FcPPh_2_.

**Table 2:** Calculated Methyl Cation Affinities for **1** and **3** (MCA; Values in kJ mol^–1^ at 298.15 K)^a^

Compound/Moiety	MCA (isolated molecule)	MCA (CHCl_3_ solution)
**1** (P)	701	560
**1** (Sb)	569	424
**3** (P)	659	517
FcPPh_2_	666	533
FcPCy_2_	690	559
FcSbPh_2_	546	412

a Estimated at the PBE0­(d3)/def2-TZVP/ECP­(Au,Sb) level of theory. The site considered is indicated (P or Sb).

A complementary picture was provided by the fluoride ion affinities (FIA)^37^ calculated for **3**, the model catecholatostiborane, FcSb­(O_2_C_6_Cl_4_)­Ph_2_, and the isomeric (1,1′ instead of 1,2) compounds R_2_PfcSb­(O_2_C_6_Cl_4_)­Ph_2_ (R = Ph, Cy). FIA is defined as the negative reaction enthalpy (−Δ*H*) for the (hypothetical) reaction of fluoride anions with a Lewis acid, and therefore, its value is greater for stronger Lewis acids. The data presented in Table 3 revealed only minor differences among all P,Sb derivatives^38^ and indicated that the phosphine-substituted stiboranes were all weaker (hard) Lewis acids than FcSb­(O_2_C_6_Cl_4_)­Ph_2_. This can be explained by the presence of P → Sb interaction that is canceled during the reaction with the fluoride anion. The cancellation of the structure-stabilizing interaction increases the energy of the system, thereby making the reaction with the fluoride ion less exothermic. For **3**, additional structure-specific factors, such as the repulsion between the fluoride anion and phosphorus lone pair and the steric congestion between the proximal pnictogen groups, can also play a role.

**Table 3:** Fluoride Ion Affinities (FIA) Calculated for Antimony Atoms in **3** and Some Model Compounds (Values in kJ mol^–1^ at 298.15 K)^a^

Compound	FIA (isolated molecule)	FIA (CHCl_3_ solution)^b^
**3**	305	112
FcSb(O_2_C_6_Cl_4_)Ph_2_	341	148
Ph_2_PfcSb(O_2_C_6_Cl_4_)Ph_2_	305	111
Cy_2_PfcSb(O_2_C_6_Cl_4_)Ph_2_	301	116

a Estimated at the PW6B95­(d3bj)/def2-qzvpp//PBE0­(d3)/def2-TZVP/ECP­(Sb) level of theory.

b The solvent effects were approximated using the PCM model (see Supporting Information).

### Solid-State Structures

Compounds **1**, **1**·BH_3_, and **1E** (E = O, S, Se) produced single crystals suitable for structure determination. Compounds **1**·BH_3_ and **1S** crystallized as racemic twins (space groups *P*2_1_ and *Pna*2_1_) with four and two practically identical molecules in the asymmetric unit, respectively. The structures of **1**, **1O,** and **1Se** contained only one crystallographically independent molecule. No two compounds were isostructural despite their overall similarity. The structures are shown in Figure 1, and the key geometric data are listed in Table 4. Additional structure diagrams and geometric parameters are available in the Supporting Information (parameters in ).

**Figure 1:**
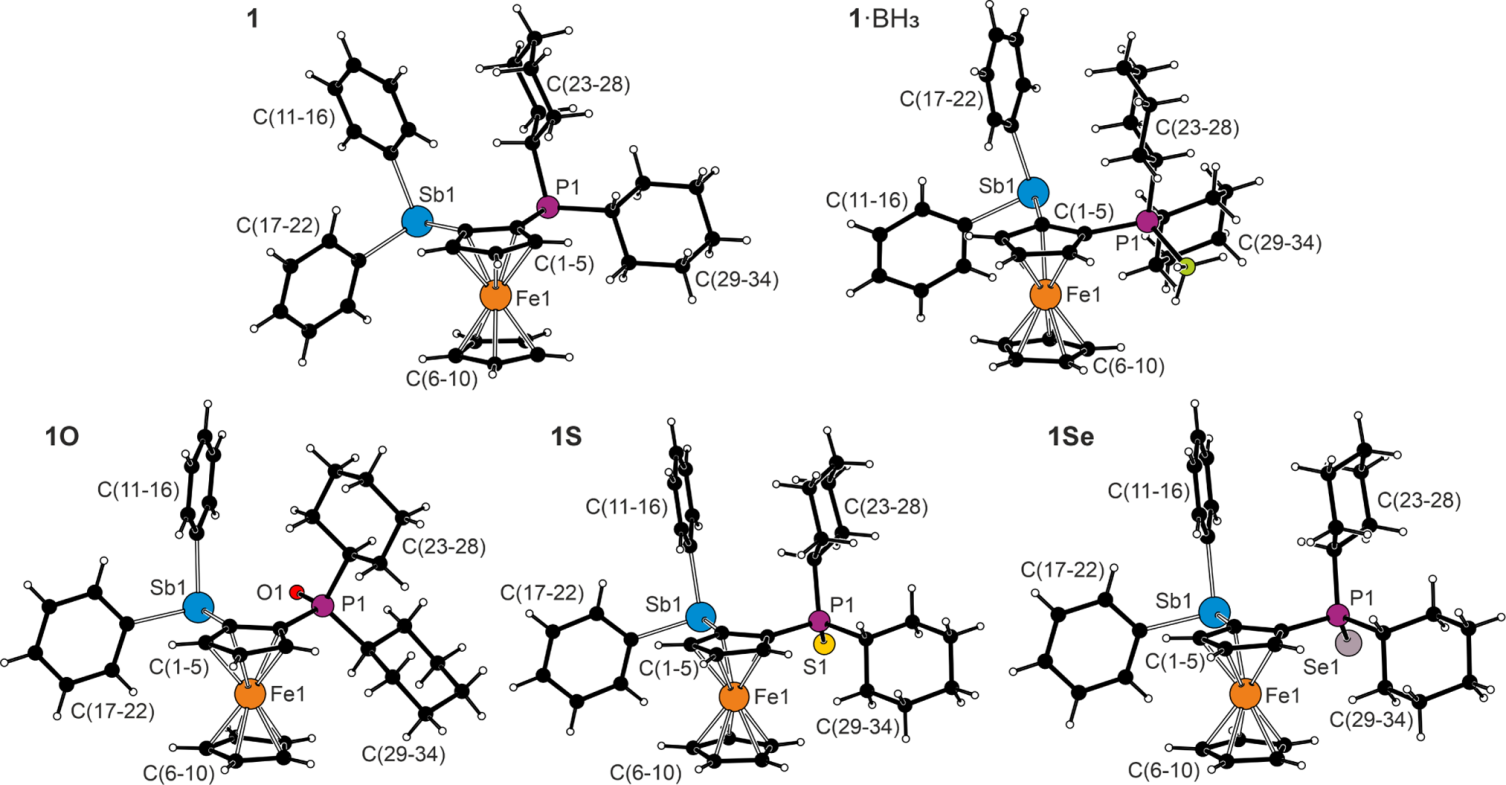
Molecular structures of type **1** compounds (only molecule 1 is shown for compounds **1**·BH_3_ and **1S**).

**Table 4:** Selected Geometric Parameters for Type **1** Compounds (in Å and Deg)^a^

Parameter	**1**·BH_3_	**1**	**1O**	**1S**	**1Se**
E	B	void	O	S	Se
Sb1–C1	2.133(3)-2.147(3)	2.127(2)	2.142(3)	2.131(4)/2.135(3)	2.143(4)
Sb1–C11	2.144(4)-2.161(4)	2.160(2)	2.151(3)	2.158(4)/2.161(4)	2.154(5)
Sb1–C17	2.145(4)-2.159(4)	2.160(2)	2.173(3)	2.171(4)/2.170(4)	2.168(4)
P1–C2	1.798(4)-1.803(4)	1.827(2)	1.790(2)	1.803(3)/1.801(3)	1.802(3)
P1–C23	1.828(4)-1.848(4)	1.872(2)	1.824(3)	1.840(4)/1.843(4)	1.845(4)
P1–C29	1.829(4)-1.845(4)	1.863(2)	1.822(3)	1.848(4)/1.833(4)	1.832(4)
P1–E1	1.925(5)-1.937(5)	n.a.	1.505(2)	1.956(1)/1.958(1)	2.116(1)
Sb1···E1	n.a.	3.6701(6)^b^	2.828(2)	3.707(1)/3.735(1)	3.8275(7)
Σ*r*_cov_	n.a.	2.46	2.05	2.44	2.59
Σ*r*_vdW_	n.a.	3.86	3.58	3.86	3.96
Sb1–C1–C2–P1	2.4(6) to – 6.1(5)	7.6(2)	10.6(3)	8.4(5)/8.9(5)	12.2(6)

a Compound **1**·BH_3_ crystallized with four independent molecules. The values represent ranges of the experimental values. For **1S**, parameters are given for the two crystallographically independent molecules in the structure. Σ*r*_cov_ and Σ*r*_vdW_ are the sums of the respective covalent and van der Waals radii. n.a. = not applicable.

b Sb1···P1 distance is given.

The molecules of all type **1** compounds comprised undistorted ferrocene units with parallel cyclopentadienyl rings. The largest tilt angles were determined for **1**·BH_3_ (6–7° for the four molecules), whereas in the remaining compounds, they remained below 5°. Even so, no significant torsion was observed at the substituted cyclopentadienyl ring, as evidenced by the Sb1–C1–C2–P1 torsion angles of approximately 2–6° for **1**·BH_3_, 7.6(2)° for **1**, and 8–12° for the phosphine chalcogenides **1E**. The Sb–C­(ferrocene) bonds were consistently shorter than the Ph–C­(Ph) bonds, as previously reported.^21,22^ The associated C–Sb–C angles in **1** varied only marginally (≈2°), while those in **1**·BH_3_ and the P-chalcogenides were more differentiated (C­(ferrocene)–Sb–C­(Ph) < C­(Ph)–Sb–C­(Ph)). More importantly, the C–Sb–C angles were consistently narrower than the C–P–C angles. This reflects not only the lower steric demands of the phenyl substituents and longer Sb–C bonds but also the less efficient mixing of the diffuse antimony 5s and 5p orbitals that is responsible for the departure from the ψ-tetrahedral sp^3^ geometry to an orthogonal p^3^ arrangement and increased s character of the lone pair at the Sb atom.^39^ The P–C bond lengths decreased from **1** through **1**·BH_3_/**1S**/**1S** to **1O**,^40^ and an approximately opposite trend was noted for the C–P–C angles.

The compounds differed in the mutual orientation of the pnictogen substituents. In the borane adduct **1**·BH_3_, the P–B bond was directed away from the stibine moiety, but in the remaining compounds, the P–E bonds (E = O, S, and Se) and the phosphorus lone pair were oriented toward the stibine moiety. The E···Sb separations in all compounds exceeded the sum of the covalent radii^41^ but, at the same time, were shorter than the sum of the respective van der Waals radii.^42^ However, whereas the Sb···E distances in **1**, **1S**, and **1Se** only approached the threshold values (see Table 4), the Sb···O distance in **1O** was significantly shorter (by 0.75 Å), indicating a relatively strong intramolecular interaction. This situation was reflected in the bending at the pivotal C1–Sb1 and C2–P1 bonds. For **1**·BH_3_, whose BH_3_ unit was directed away from the Sb atom, the C2–C1–Sb1 and P1–C2–C1 angles were more open than the respective complementary angles C5–C1–Sb1 and C3–C2–P1 (see Figure 2). In **1**, the C2/5–C1–Sb1 angles were close to the ideal value of 126°, but the C2–P1 bond was inclined toward the Sb1 atom, albeit without a significant P → Sb interaction because the phosphorus lone pair was directed below the cyclopentadienyl plane. The P···Sb separation shorter than the sum of the van der Waals radii was thus a structural consequence rather than the result of hypervalent interactions, as confirmed by DFT calculations (*vide infra*).

**Figure 2:**
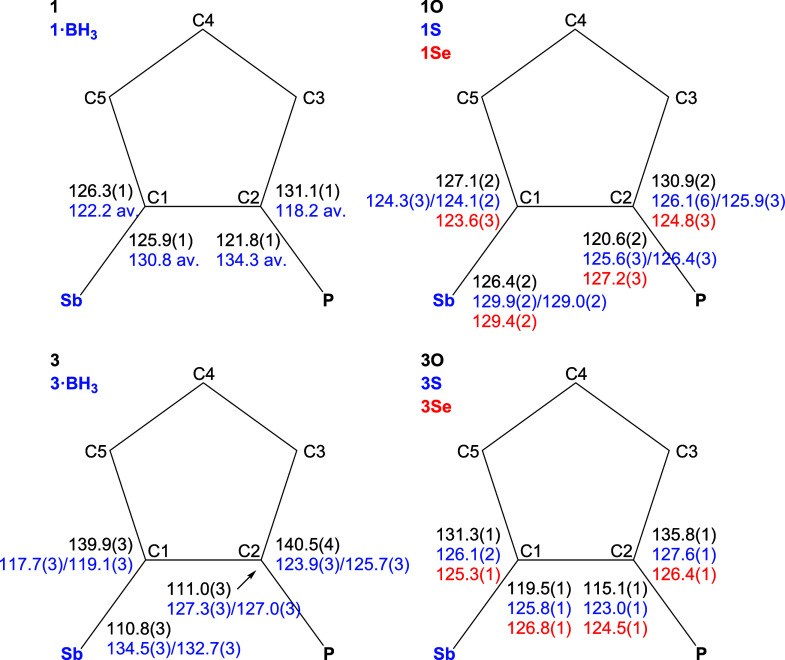
Outer C–C–Sb and C–C–P angles in the molecules of **1**, **1**·BH_3_ and **1E**, and the corresponding catecholatostiboranes **3**, **3**·BH_3_ and **3E** (E = O, S, and Se). The parameters for **1**·BH_3_ are the averages for the four independent molecules (the individual values differ by <1.2°). For **1S** and **3**·BH_3_, values for both independent molecules are given.

Among the phosphine chalcogenides, the most pronounced bending was noted for the C2–P1 bond in **1O** as a result of the phosphoryl oxygen approaching the Sb atom. Conversely, the same angles in **1S** and **1Se** were less affected, except for **1Se**, where the opening of the C2–C1–Sb1 angle was attributed to the presence of a larger selenium atom. Although also affected by the varying P–E distances, the C1–C2–P1–E torsion angles corresponded with these observations and were smaller in **1O** (−7.5(2)°) than in the heavier chalcogenides (**1S**: –47.3(3)°/–48.2(4)°; **1Se**: –50.0(4)°). The P–E bonds (E = BH_3_, S, and Se;^40^ suitable data for E = O were not available^43^ remained within the normal ranges.

Compounds **3**, **3**·BH_3_, and **3E** (E = O, S, Se) were also structurally authenticated using X-ray diffraction analysis (Figure 3, parameters in Tables 5 and ). The oxidation of the stibine moiety led to the shortening of the Sb–C bonds (compared with the parent stibines) and changed the geometry around the Sb atom. In **3**·BH_3_, where the phosphine moiety was unavailable for Lewis pair interactions, the geometry around the Sb atom was square pyramidal with a τ_5_ index of 0.03, which is close to the ideal value (ideal square pyramid and trigonal bipyramid would yield τ_5_ = 0 and 1, respectively).^44^ The phenyl ring C(17–22) occupied the apical position while the C1, C11, O1 and O2 atoms constituted a basal plane (within ≈0.05 Å), from which the Sb atom was displaced by 0.357(1)/0.391(1) Å (parameters for two structurally independent molecules are given; the O1–Sb1–C1 and O2–Sb1–C11 angles were 156.3(2)/155.3(1)° and 158.0(2)/157.2(2)°, respectively). The BH_3_ group was oriented toward the Sb atom (unlike the corresponding stibine **1**·BH_3_, where it pointed away). This change in conformation enabled the uncommon, structure-stabilizing interactions between Sb and the negatively polarized BH hydrogens, B^δ+^–H^δ−^···Sb.^45^ The shortest Sb···H separations of 2.48(5) and 2.32(5) Å for molecules 1 and 2, respectively, were well below the sum of van der Waals radii (3.16 Å).^42^

**Figure 3:**
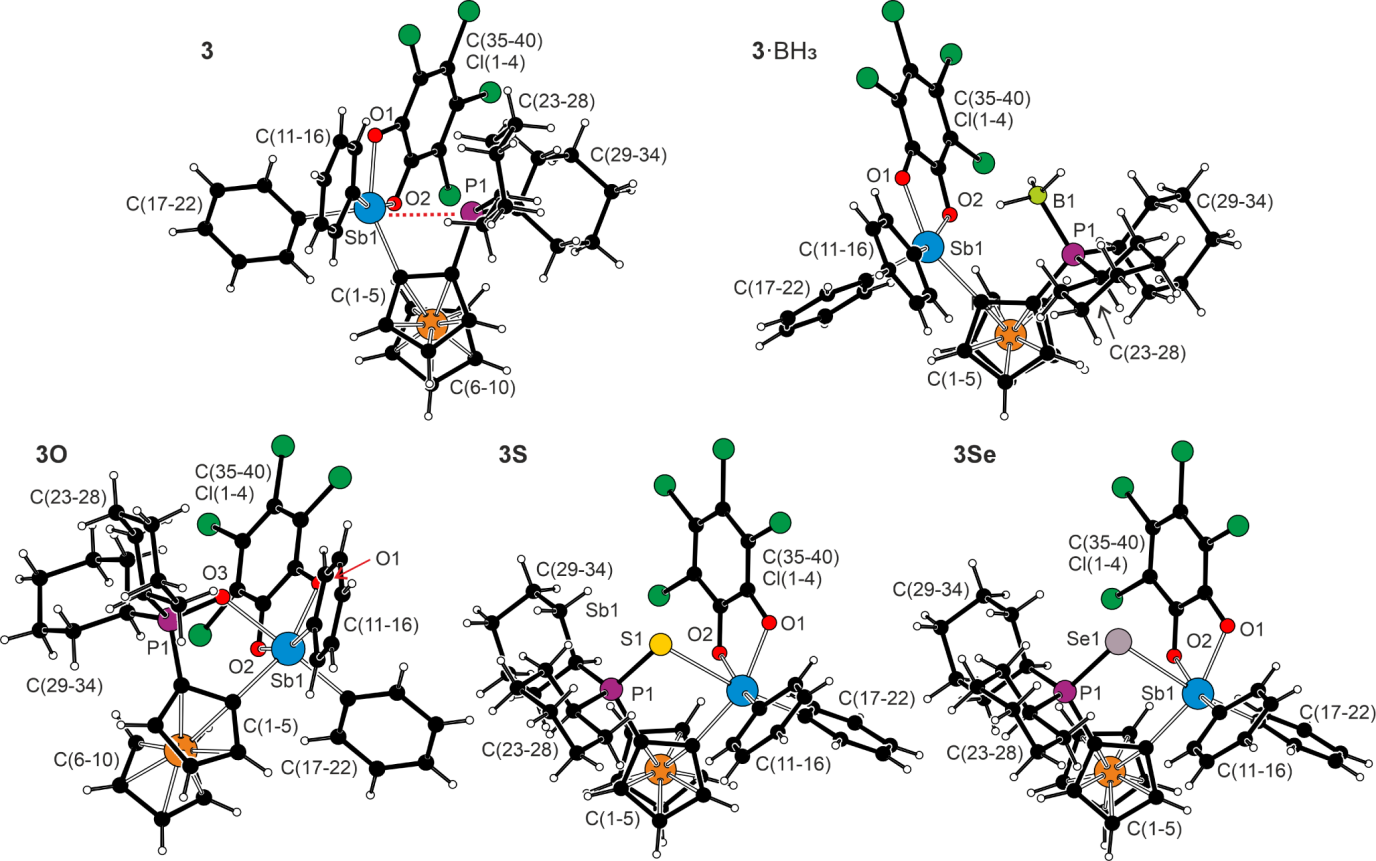
Molecular structures of **3**, **3**·BH_3_, and **3E** (E = O, S, and Se; only molecule 1 is shown for compounds **3**·BH_3_ and **3S**).

**Table 5:** Selected Distances and Angles for Type **3** Compounds (in Å and Deg)

Parameter^a^	**3**·BH_3_	**3**	**3O**	**3S**	**3Se**
E	B1 (mol 1/mol 2)	void	O3	S1	Se1
Sb1–C1	2.125(4)/2.118(4)	2.137(5)	2.118(1)	2.121(2)	2.123(1)
Sb1–C11	2.145(4)/2.142(4)	2.137(4)	2.136(2)	2.138(2)	2.141(2)
Sb1–C17	2.108(5)/2.109(5)	2.117(5)	2.121(1)	2.128(2)	2.137(1)
Sb1–O1	2.054(3)/2.057(3)	2.053(3)	2.058(1)	2.081(1)	2.094(1)
Sb1–O2	2.070(3)/2.069(3)	2.113(3)	2.077(1)	2.084(1)	2.089(1)
P1–C2	1.806(4)/1.800(4)	1.811(5)	1.789(2)	1.795(2)	1.794(1)
P1–C23	1.847(4)/1.841(4)	1.850(5)	1.812(2)	1.834(2)	1.836(1)
P1–C29	1.836(4)/1.842(4)	1.844(4)	1.804(2)	1.826(2)	1.826(2)
P1–E	1.922(5)/1.912(5)	n.a.	1.529(1)	1.9946(6)	2.1475(5)
Sb1···E	n.a.	2.859(1)^b^	2.225(2)	2.8085(5)	2.8960(5)
Sb1–C1–C2–P1	1.8(6)/–5.9(6)	0.2(4)	2.6(2)	–4.9(2)	–2.9(2)
C1–C2–P–E	–6.1(4)/–7.3(4)	n.a.	–8.1(1)	–7.4(2)	–8.1(1)

a For **3**·BH_3_, values for molecule 1/molecule 2 are given. n.a. = not applicable.

b Sb1···P1 distance.

In free phosphine **3**, the arrangement around Sb1 changed toward an octahedral shape, with one site occupied by the phosphorus lone pair. Although the angles in the “basal plane” O1–Sb1–C1 (152.3(2)°) and O2–Sb1–C11 (164.4(4)°) only slightly changed from those of **3**·BH_3_, the τ_5_ index increased to 0.20 (calculated by neglecting the sixth site occupied by the phosphorus lone pair). A comparison of the C–C–Sb/P angles revealed that the substituents in **3** were bent toward each other, indicating a P → Sb interaction (Figure 2). The Sb1···P1 distance of 2.859(1) Å was shorter than the sum of the van der Waals radii (*cf*. 4.015(1)/3.966(1) Å in **3**·BH_3_), and the torsion angle at the C1–C2 bond was only 0.2(4)°.

The structures of chalcogenides **3O**, **3S,** and **3Se** were mutually similar and consisted of hexacoordinate Sb atoms in a distorted octahedral environment. The *cis*-interligand angles in the entire series ranged 77–102°. The smallest angle was always associated with the chelating catecholate ligand^46^ and was compensated for by an expansion of the opposite SbC_3_ triangular face. The E–Sb1–C17 angles were 171.56(5)° in **3O**, 170.54(5)° in **3S**, and 170.02(4)° in **3Se** (*cf*. the P1–Sb1–C17 angle of 162.7(1)° in **3**). Compared with stibines **1E**, the P1–E1 distances in the stiboranes were elongated by 0.024 Å in **3O**, 0.038 Å in **3S**, and 0.032 Å in **3Se**. These changes were rather minor and corresponded to the donation of the nonbonding electron density.^47^ The Sb1–C1–C2–P1 and C1–C2–P1–E torsion angles of 3–5° and ≈8° (in absolute values), respectively, indicated nearly planar C_2_SbPE rings in all chalcogenides (within 0.14 Å). Their conformations could be described as a slightly open envelope with the E atom at the tip position. Finally, the ferrocene units remained unperturbed, showing Fe–C distances in narrow ranges and tilt angles below ca. 6°.

The crystal structure of zwitterion **4** is presented in Figure 4 along with the principal geometric data. Further parameters are available in . The structure revealed the presence of tetracoordinate P (angles around P1: 101–119°) and hexacoordinate Sb atoms, interconnected by a bridging catecholate unit into an eight-membered, C_4_SbPO_2_ ring. The bridging catecholate bonded slightly asymmetrically: the Sb1–C1–C5 and P1–C2–C3 (outer) angles were more acute (119.2(2)° and 122.9(2)°) than those within the ring (Sb1–C1–C2 134.1(2)° and C1–C2–P1 128.5(2)°). In addition, the Sb1–O3 bond was longer than the Sb1–O1/O2 distance in the same molecule but still shorter than the Sb1···O3 separation in **3O**. The P1–O4 bond was significantly shorter than the P–O bonds in square-pyramidal catecholatophosphoranes R_3_P­(O_2_C_6_Cl_4_) (≈1.73 Å;^33,48^ *N.B*. the P–O distances in trigonal bipyramidal Ph_3_P­(O_2_C_6_Cl_4_) were strongly differentiated: ≈1.64 and 1.92 Å)^33b^ as well as in cyclic phosphates (R′_4_N)­[P­(O_2_C_6_R_4_)_3_] (≈1.64 Å; R/R′ = H/Et, Cl/Bu).^49^ At the same time, it exceeded the value typical for phosphine oxides (R_3_P=O: ≈1.49 Å for R = Cy and Ph^47a,50^ ).

**Figure 4:**
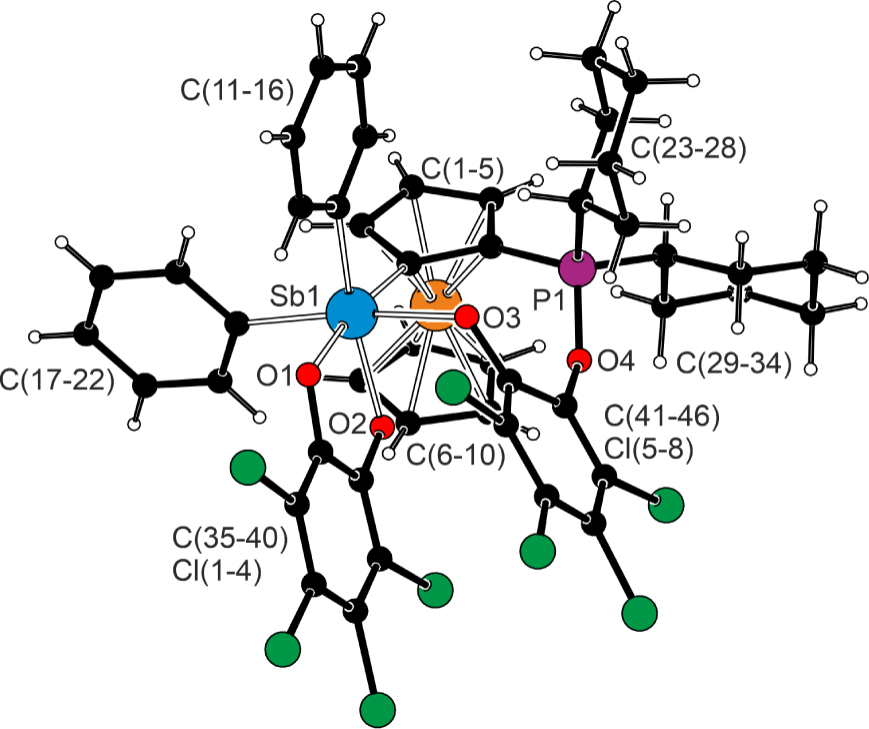
Molecular structure of **4**. Selected distances (in Å): Sb1–O1 2.069(2), Sb1–O2 2.071(2), Sb–O3 2.164(2), Sb1–C1 2.152(2), Sb1–C11 2.135(3), Sb1–C17 2.149(3), P1–O4 1.586(2), P1–C2 1.764(3), P1–C23 1.819(3), and P1–C29 1.813(3).

### Theoretical Analysis of the Bonding Situation in Type 1 and 3 Molecules

Structural features suggesting hypervalent interactions in the molecules of compounds **1/1E** and **3/3E** led us to complement the direct structural information with the results of theoretical calculations. Initially, we visualized the interactions using scatter plots of the reduced density gradient (RDG) versus the product of the electron density and the sign of the second Hessian eigenvalue (sign­(λ_2_)­ρ), obtained by noncovalent interaction (NCI) analysis (Figure 5).^51^ These plots corroborated the presence of attractive interactions in all phosphine chalcogenides **1E**. Regions with negative sign­(λ_2_)­ρ values, indicative of attractive interactions, were subsequently identified between the chalcogenide and antimony atoms (see Figure 6). These interactions were classified as pnictogen bonding, i.e., the interactions between the σ hole at the antimony and the nonbonding electron pair of the chalcogen atom.^52^ This analysis also excluded the presence of a similar interaction in phosphine **1**, in agreement with the crystal structure data.

**Figure 5:**
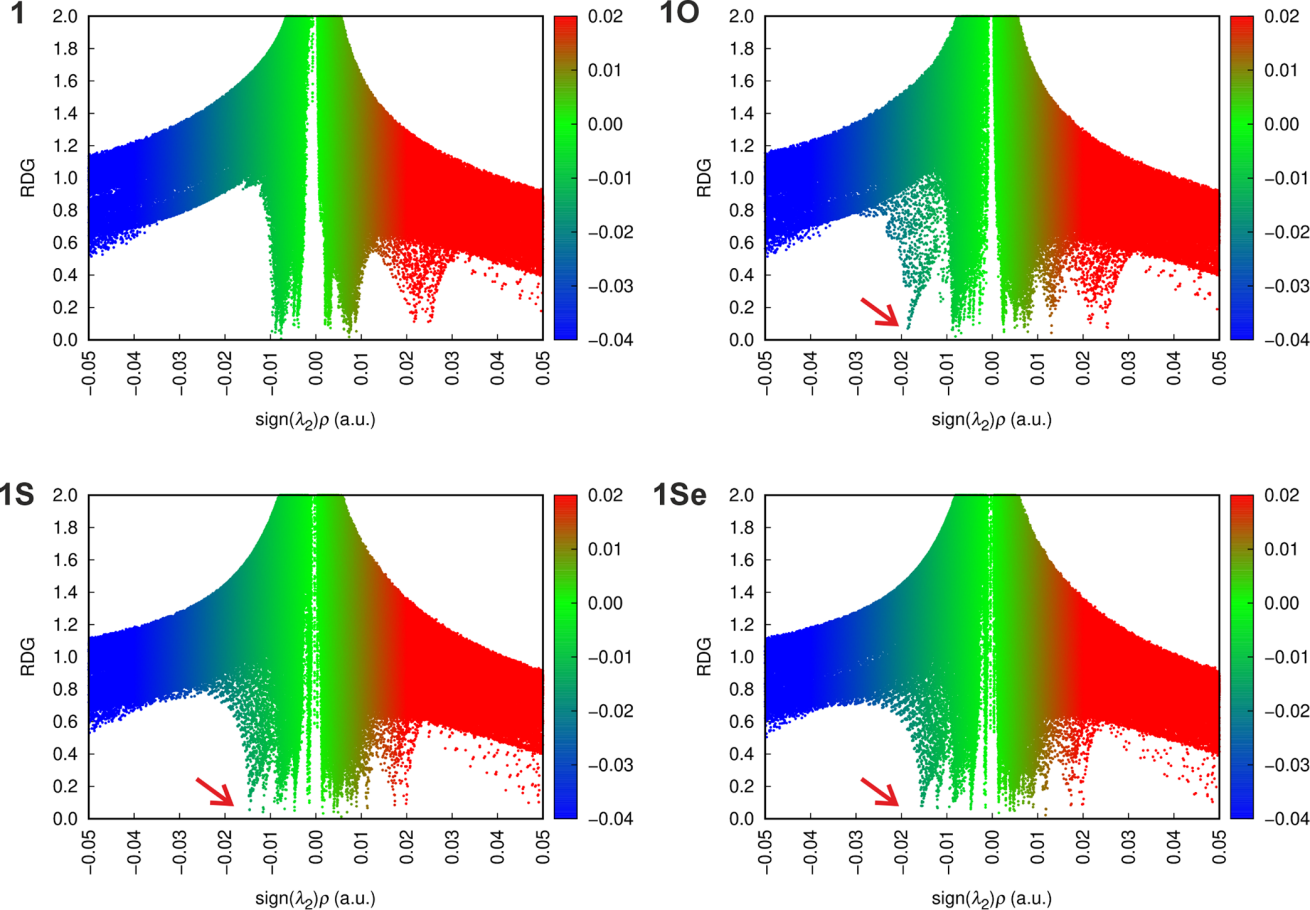
Scatter plots of the reduced density gradient (RDG) versus the product of the electron density and the sign of the second Hessian eigenvalue (sign­(λ_2_)­ρ). The regions of attractive interactions corresponding to pnictogen bonding are indicated by arrows.

**Figure 6:**
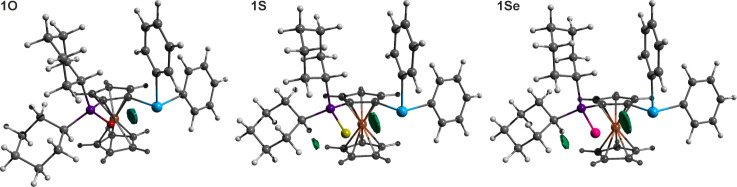
NCI plots of compounds **1E** (E = O, S or Se) showing RDG isosurfaces with *S*(*r*) = 0.5. For clarity, the RDG isosurfaces are shown only for regions with negative sign­(λ_2_)­ρ values (ranging from –0.05 to –0.02), corresponding to attractive noncovalent interactions (cyan regions).

The nature of the intramolecular interactions was further evaluated by topological analysis of the electron density.^53^ The analysis revealed bond critical points (bcps) between the chalcogen atoms and antimony in **1E**. No bond critical point between phosphorus and antimony could be located for **1**, consistently with the NCI analysis. The values of the real space functions (Table 6) at the bond critical points, particularly the low electron densities (ρ_bcp_) and close-to-zero positive values of their Laplacians (∇^2^ρ_bcp_), indicated weak noncovalent interactions.^54^ The nature of the interactions was better reflected by the ratio of the potential and kinetic energy density (|*V*|/*G*), which distinguishes covalent (|*V*|/*G* > 2), dative (2 > |*V*|/*G* > 1), and ionic (|*V*|/*G* < 1) bonding.^55^ The values determined for **1E** (Table 6) suggested a predominantly electrostatic nature of the interactions, which was further supported by the higher kinetic energy density to electron density (*G*/ρ_bcp_) ratios.

**Table 6:** Electron Density (*ρ*_bcp_) and Its Laplacian (∇^2^ *ρ*_bcp_), the Total Electronic Density (*H*), the Ratio of the Potential and Kinetic Energy Density (|*V*|/*G*), and the Ratios of Kinetic (*G*/*ρ*_bcp_) and Total Energy Density (*H*/*ρ*_bcp_) to the Electron Density at the Bond Critical Points Located between Antimony and the Respective Donor Atom (P, O, S, or Se) and the Corresponding Bond Distances

		Bond length [Å]							
Compound	Bond	exp.	calc.^a^	ρ_bcp_ [a.u.]	∇^2^ρ_bcp_ [a.u.]	*H* (a.u.)	|*V*|/*G* (a.u.)	*G*/ρ_bcp_ (a.u.)	*H*/ρ_bcp_ (a.u.)
**1O**	Sb···O	2.828(2)	2.920	0.019	0.052	0.01·10^–2^	0.95	0.66	0.005
**1S**	Sb···S	3.707(1)/3.735(1)^b^	3.437	0.015	0.029	0.04·10^–2^	0.94	0.46	0.027
**1Se**	Sb···Se	3.8275(7)	3.498	0.016	0.025	–0.01·10^–2^	1.03	0.41	–0.008
**3**	Sb···P	2.859(1)	2.844	0.046	0.020	–1.11·10^–2^	1.69	0.35	–0.24
**3O**	Sb···O	2.225(1)	2.259	0.064	0.171	–1.43·10^–2^	1.25	0.89	–0.22
**3S**	Sb···S	2.8085(5)	2.802	0.046	0.038	–0.94·10^–2^	1.50	0.43	–0.22
**3Se**	Sb···Se	2.8960(5)	2.293	0.041	0.026	–0.87·10^–2^	1.58	0.37	–0.21

a Calculated at the PBE0­(d3)/def2-TZVP/ECP­(Sb) level of theory.

b Distances in the two crystallographically independent molecules.

The relatively large discrepancy between the optimized and experimental interatomic E···Sb distances in **1S** and **1Se** (see Table 6) can be ascribed to the limitations of the employed DFT method (especially in the gas phase). The calculations were performed using the PBE0 functional in combination with the def2-TZVP basis set and D3 empirical dispersion correction (for details, see the ), which is generally reliable but can overestimate the strength of weak interactions such as pnictogen bonding. This feature has been reported for analogous noncovalent interactions, where dispersion corrections led to overestimation of attractive forces.^56^ Neglecting of intermolecular interactions and crystal packing forces in gas-phase geometry optimizations also contributed to the differences. We note in passing that repeated optimalization of these structures employing the PCM model^57^ to simulate solvent effects (as suggested by one of the reviewers) led to a better agreement between the experimental and calculated values. Nevertheless, the changes in values of real space functions obtained by topological analysis were only small and did not change the previous conclusions (see ).

The Lewis acidity of the antimony atom in stiboranes **3** and **3E** was significantly enhanced. This strengthened the intramolecular E → Sb interactions (already indicated by a noticeable shortening of the interatomic distances) and even resulted in a distinct interaction of this kind in the molecule of phosphine **3**. The change was confirmed by the calculated values (Table 6; see also ). Stronger interactions were consistently indicated by higher electron densities at the bond critical points (ρ_bcp_). The values of other real space functions implied intermediate strength dative bonds involving heavy elements with rather diffuse valence shells (antimony in the present case).^58,59^ The most significant feature was the change in the ratio of potential and kinetic energy density (|*V*|/*G*) from the range typical for ionic or electrostatic interactions (|*V*|/*G* < 1) to the intermediate region (2 > |*V*|/*G* > 1) characteristic of donor–acceptor interactions. The increased covalent nature of the pnictogen interactions was further supported by the more negative total energy density at the bond critical point and by the negative values of the total energy density to the electron density *H*/ρ_bcp_. Nevertheless, the obtained values also pointed to a greater electrostatic contribution in the case of **3O**. This was reflected not only by the lower ratio of potential and kinetic energy density (|*V*|/*G*) but also by a higher value of the kinetic energy density to the electron density *G*/ρ_bcp_.

The deductions from the topological analysis were corroborated by the intrinsic bond orbital (IBO) approach.^60^ The IBOs corresponding to the interaction between antimony and the respective donor atom are shown in Figure 7. The charge distribution between the bonded atoms reflected varying degrees of electron sharing [*N.B*. an ideal covalent bond is characterized by equal values (1.0/1.0)]. A higher charge localization at the acceptor atom (Sb) indicated a gradual increase in the covalency of the E → Sb interaction from oxide to selenide in chalcogenides **3E**: [O(1.71)/Sb(0.18)] for **3O**, [S(1.64)/Sb(0.23)] for **3S**, and [Se(1.61)/Sb(0.26)] for **3Se**. In compound **3**, the charge distribution [P(1.65)/Sb(0.26)] was comparable to that in **3Se**, suggesting a similar degree of covalency.

**Figure 7:**
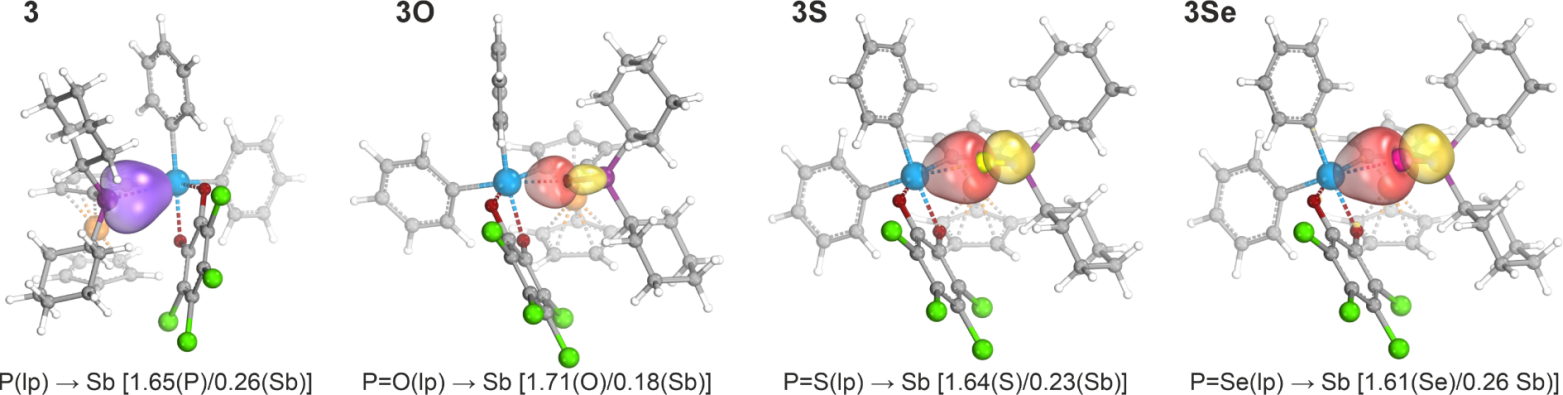
Selected intrinsic bond orbitals (IBOs) of stiboranes **3**, **3O**, **3S**, and **3Se**. The values in parentheses indicate the fraction of the bonding electrons assigned to the individual atoms (lp = lone pair).

### Coordination Study and Catalytic Experiments

To probe the coordination preferences of **1** and the impact of the change of substituents on the catalytic behavior, we prepared a series of gold­(I) complexes (Scheme 4).^61^ In line with the higher Lewis basicity of the phosphine moiety, compound **1** reacted with 1 equiv of [AuCl­(SMe_2_)] to selectively produce phosphine complex **5**. Only when the amount of the gold­(I) precursor was increased to 2 equiv., the reaction afforded the digold­(I) complex **6**. Both complexes were isolated as orange solids. Attempts to purify the bulk materials by crystallization were hindered by a relatively rapid decomposition, visually indicated by the formation of a gold mirror (the solid samples decomposed as well, but relatively slowly). For example, attempted crystallization of complex **6** from chloroform produced several crystals of compound **7**, which was alternatively prepared by oxidation of **5** with SOCl_2_. Complex **7** underwent smooth halide exchange to produce the analogous difluorostiborane **8**. Another stiborane complex, compound **9**, was obtained by the oxidation of **6** with *o*-chloranil. Similar to **5** and **6**, complexes **7**-**9** decomposed in solution.

**Scheme 4:**
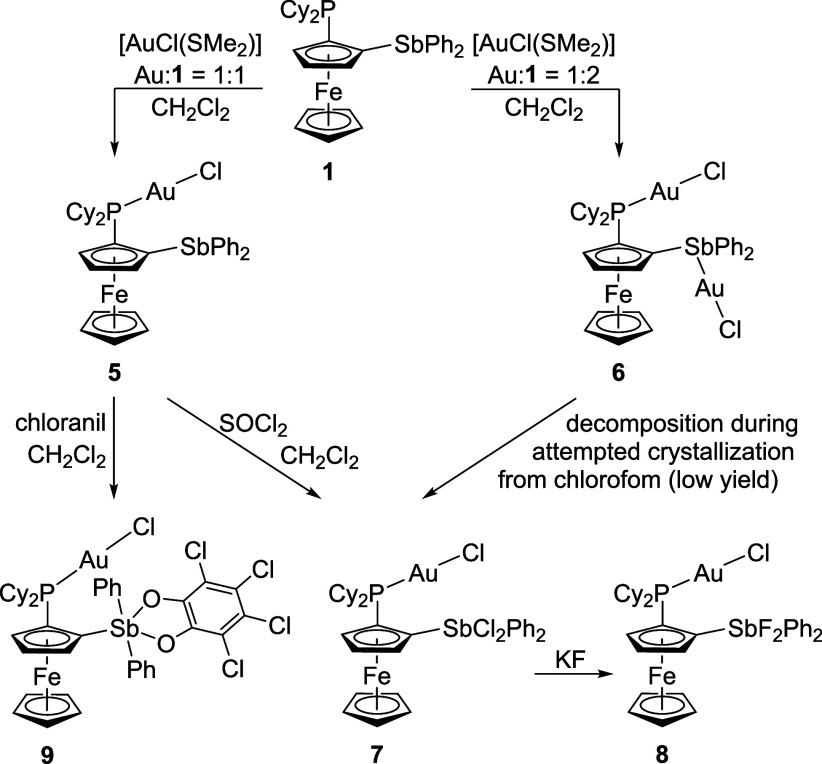
Preparation and Transformations of the Gold­(I) Complexes

The coordination of the phosphine moiety was indicated by deshielded ^31^P NMR signals (δ_P_ 42.7 and 38.6 for **5** and **6**, respectively; in CD_2_Cl_2_), and a further, downfield shift was observed upon oxidation of the stibine moiety in **5** (δ_P_ 45.7 for **7**, 46.9 for **8**, and 44.1 for **9**). The ESI MS spectra corroborated the formation of **5**, **6**, and **8**, whereas those of **8** and **9** were rather inconclusive because of deeper fragmentation. All complexes were crystallized and structurally authenticated by single-crystal X-ray diffraction analysis (see Figure 8 and Table 7).

**Figure 8:**
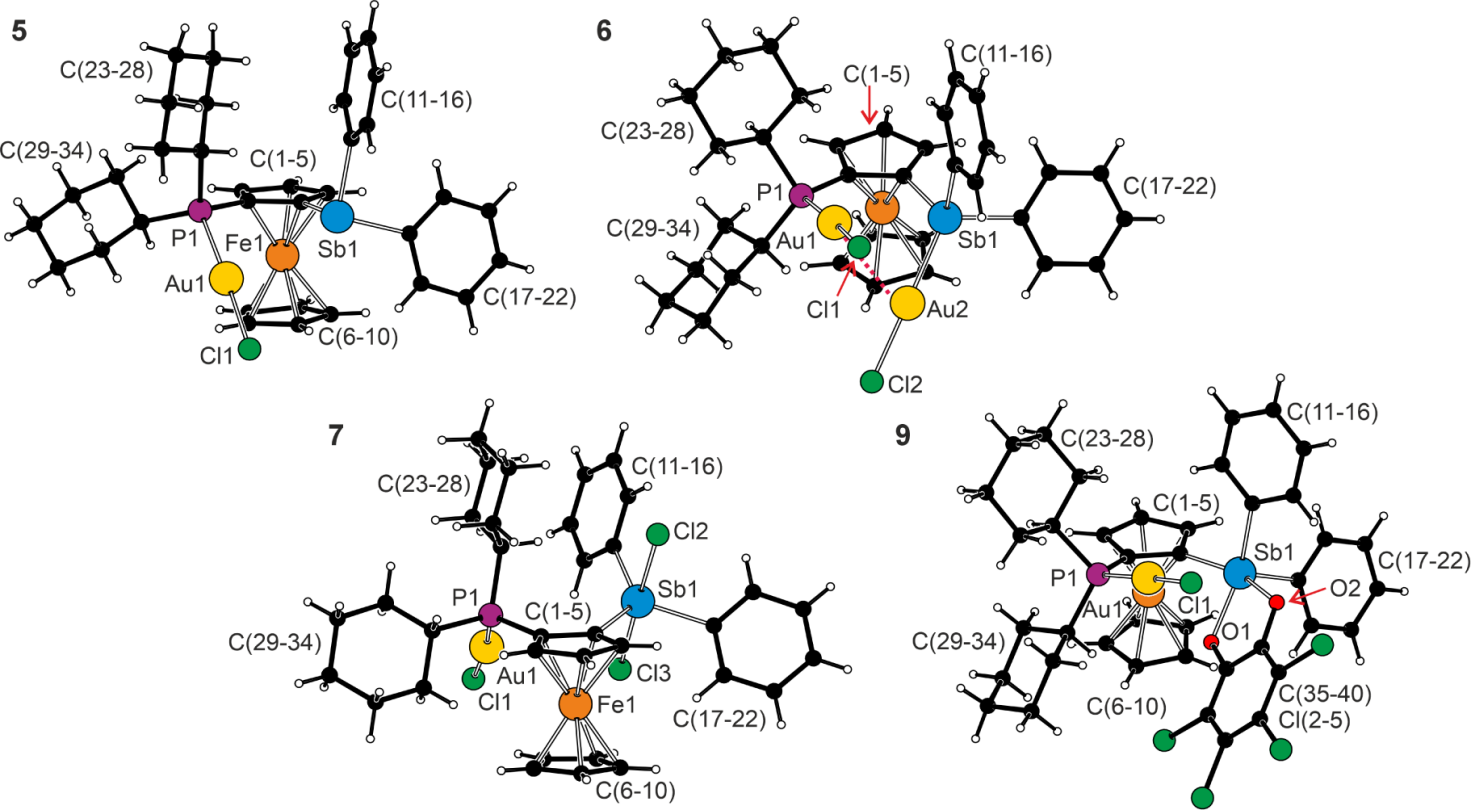
Molecular structures of **5**, **6**, **7**, and **9**. For **5**, only one position of the disordered gold atom is shown for clarity, and the aurophilic interaction in the molecule of **6** is indicated by a red dotted line. The structure of **8** and additional structure diagrams are available in the .

**Table 7:** Selected Distances and Angles for Au­(I) Complexes **5**, **6**, **7**, **8**, and **9** (in Å and Deg)

Parameter^a^	**5** ^b^	**6** ^c^	**7**	**8**	**9**
X1/X2	n.a.	n.a.	Cl2/Cl3	F1/F2	O1/O2
P1–Au1	2.305(2)/2.167(4)	2.2425(6)	2.2337(9)	2.2319(8)	2.2360(8)
Au1–Cl1	2.250(2)/2.407(4)	2.3012(6)	2.297(1)	2.2994(9)	2.2768(8)
P1–Au1–Cl1	175.3(1)/168.6(3)	173.10(3)	174.02(3)	175.72(3)	173.15(4)
Sb1–C1	2.146(4)	2.096(2)	2.107(3)	2.087(3)	2.121(3)
Sb1–C11	2.158(4)	2.113(3)	2.135(3)	2.102(3)	2.125(3)
Sb1–C17	2.160(5)	2.113(2)	2.101(3)	2.106(3)	2.107(3)
Sb1–X1	n.a.	n.a.	2.4582(8)	1.984(2)	2.042(2)
Sb1–X2	n.a.	n.a.	2.472(1)	1.974(2)	2.061(2)
X1–Sb1–X2	n.a.	n.a.	178.55(3)	177.90(8)	78.61(9)
P1–C2	1.799(4)	1.814(2)	1.808(3)	1.803(3)	1.802(3)
P1–C23	1.867(6)	1.839(2)	1.844(3)	1.852(3)	1.837(3)
P1–C29	1.842(5)	1.841(2)	1.836(3)	1.838(3)	1.826(3)
Sb1–C1–C2–P1	1.9(5)	15.8(3)	6.2(5)	–3.9(4)	5.8(4)

a n.a. = not applicable.

b Atom Au1 was refined over two positions due to disorder. The parameters pertaining to both positions are given.

c Further data: Au1···Au2 = 3.0602(8), Sb1–Au2 = 2.4845(9), Au2–Cl2 = 2.274(1), Sb1–Au2–Cl2 = 176.39(3).

The structure determination of **5** revealed a linear P–Au–Cl fragment pointing down the ferrocene unit (Fe···Au ≈ 4 Å) with parameters similar to those reported for [AuCl­(Cy_2_PfcCN-κ*P*)][Bibr ref62] and [(μ­(P,Sb)-**H**)­(AuCl)_2_].^22^ The distance between the gold and antimony atoms was approximately 3.45 Å (the Au atom was disordered). In the structure of digold­(I) complex **6**, the P–Au–Cl arm was oriented above the ferrocene unit, and the Sb–Au–Cl moiety was directed below (Fe1···Au2 = 4.4782(4) Å). This arrangement avoided steric clash between the AuCl units while enabling a structure-stabilizing intramolecular aurophilic interaction^63^ (Au1···Au2 = 3.0602(8)°, Cl1–Au1–Au2–Cl2 = 75.25(3)°). Nevertheless, the compound showed the largest torsion at the C1–C2 bond among the examined compounds (Sb1–C1–C2–P1 = 15.8(3)°), indicating considerable steric tension. Compared with free **1**, the coordination resulted in the opening of the C–E–C angles (E = P and Sb), with varying degrees for the individual parameters (≈1–10°).

Oxidation of the stibine moieties left the P–Au–Cl fragment in **7** and **8** virtually unaffected, but the geometry around the Sb atom changed to trigonal bipyramidal with concomitant shortening of the Sb–C bonds. A departure from an ideal geometry was indicated by the τ_5_ index of 0.85 in both cases and was ascribed to the angular distortion due to steric factors. In both structures, the halogen atoms occupied axial positions. Whereas the X–Sb–X and X–Sb–C angles were all close to 180° and 90°, respectively, the C–Sb–C angles were more differentiated. The C1–Sb1–C11 angle oriented toward the AuCl unit was wider (≈127°) and the adjacent C1–Sb1–C17 angle more acute (≈110°) than 120°; only the C11–Sb1–C17 angle at the exterior remained near the ideal value.

Lastly, the molecule of **9** combined the linear P–Au–Cl unit with a distorted, square pyramidal Sb-catecholate moiety (τ_5_ = 0.13). The geometry of the stiborane fragment was similar to that observed in compounds **3**/**3E** (*vide supra*). The AuCl arm pointed above the ferrocene unit, and the catecholate ring extended below such that the base of the square pyramid was oriented toward Au1 at a Sb1···Au1 distance of 2.3632(7) Å.

Complexes **5** and **9** were evaluated as precatalysts for the Au-mediated cyclization of *N*-propargylbenzamide (**10**) into 4,5-dihydro-5-methylene-2-phenyloxazole (**11**) (Scheme 5).^64^ The experiments were performed using 1 mol% of the gold complexes in CD_2_Cl_2_ at 25 °C, which were activated *in situ* by adding a solution of silver­(I) bis­(trifluoromethanesulfonyl)­imide (AgNTf_2_) in MeCN to the reaction mixture. The progress of the reaction was followed by ^1^H NMR spectroscopy.^62^ No reaction was observed when only the silver salt was used as the catalyst.

**Scheme 5:**
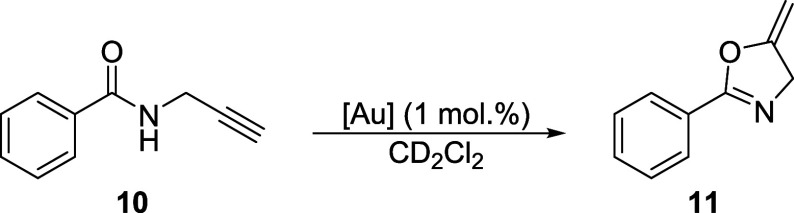
Au-Catalyzed Cyclization of *N*-Propargylbenzamide (**10**)

The reactions proceeded selectively; no other products were detected. The kinetic profiles shown in Figure 9 illustrate the considerably better catalytic performance of **9**, which achieved approximately 75% conversion after 3 h (*cf*. 44% for **5**). The difference between the catalysts resulting from **5** and **9** was attributed to the decreased electron-donating ability of the P-bound stiborane ligand **3** compared with the parent phosphinostibine **1**. As the result, the plausible Au^+^–**3** catalyst was more electrophilic and activated the coordinated triple bond of **10** toward intramolecular nucleophilic attack.^64^

**Figure 9:**
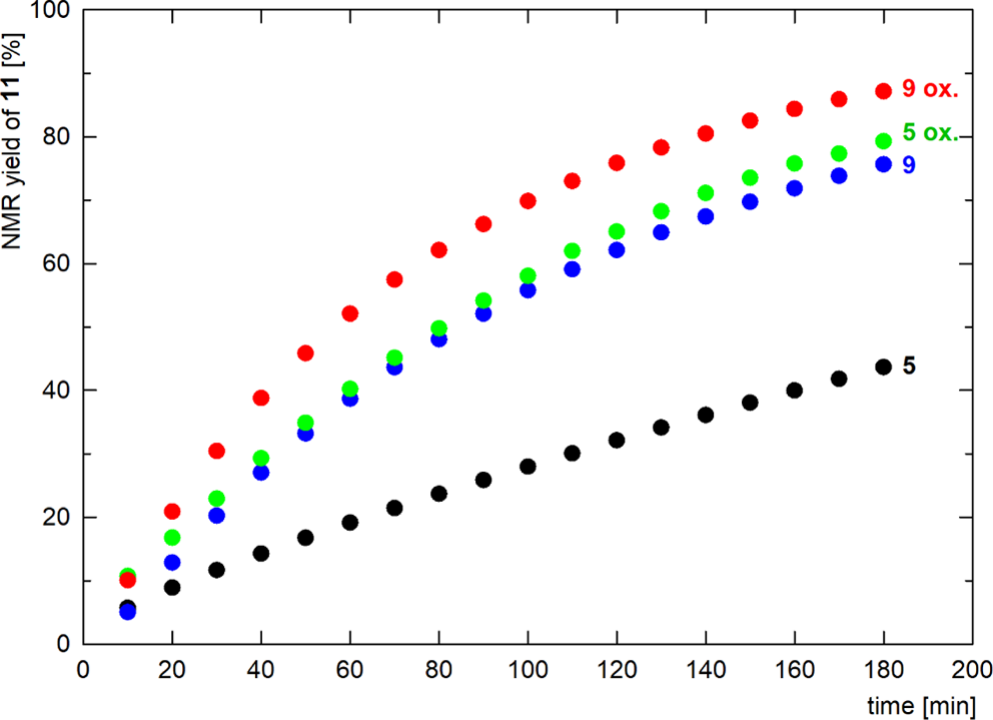
Kinetic profiles for the Au-catalyzed cyclization of **10** into oxazole **11** using catalysts based on complexes **5** and **9** as such and with added ferrocenium tetrafluoroborate (**5 ox**, **9 ox**). The data are an average of two independent runs.

Considering this feature, we added ferrocenium tetrafluoroborate (1 equiv. relative to Au) to the reaction mixture to oxidize the catalysts and make them even less electron-rich (*cf*. the Hammett σ_p_ constants of –0.18 and 0.29 for the ferrocenyl group and the corresponding ferrocenium)^65^ as observed in redox-switchable catalysis^66^ employing ferrocene ligands.^67^ For both complexes, the addition of the oxidant resulted in faster reaction rates, with complex **9** providing the cyclization product in an 87% NMR yield after 3 h. The performance of the catalyst based on oxidized **9** was comparable to that resulting from the acylphosphine complex [(FcC­(O)­PCy_2_-κ*P*)­AuCl] under similar conditions.^68^

Recent studies have shown that gold­(I) can form various noncovalent interactions beyond the well-established aurophilic contacts^63^ in its complexes, including hydrogen,^69^ halogen,^70^ or pnictogen bonds.^71^ Although Au­(I) center in the structure **5** retained its usual linear coordination geometry, the orientation of the stibine group suggested that some type of noncovalent interaction occurred between the Au and Sb atoms. This was supported by NCI analysis (Figure 10), which revealed the presence of an attractive interaction, where the gold atom served as a d-orbital donor toward antimony. Since a similar interaction was observed in complex **9** featuring the stiborane ligand (Figure 10), we questioned whether the oxidation by chloranil strengthened the Au–Sb interaction, similarly to chalcogenides **1E** and their stiborane analogs **3E**. The values of the real space functions at the bond critical points obtained by topological analysis of the electron density (see ) indicated the donor–acceptor character of the observed interactions in both cases, particularly through the |*V*|/*G* values.^72^ However, most of the calculated values were nearly identical and were therefore not suitable for assessing the strength of the interaction in both complexes. Therefore, we resorted to intrinsic bond orbital (IBO) analysis, which better responded to the small differences. In particular, IBO analysis confirmed that polarization of the gold 5d orbital oriented toward the antimony atom was greater for complex **9** than for **5** (Figure 10).

**Figure 10:**
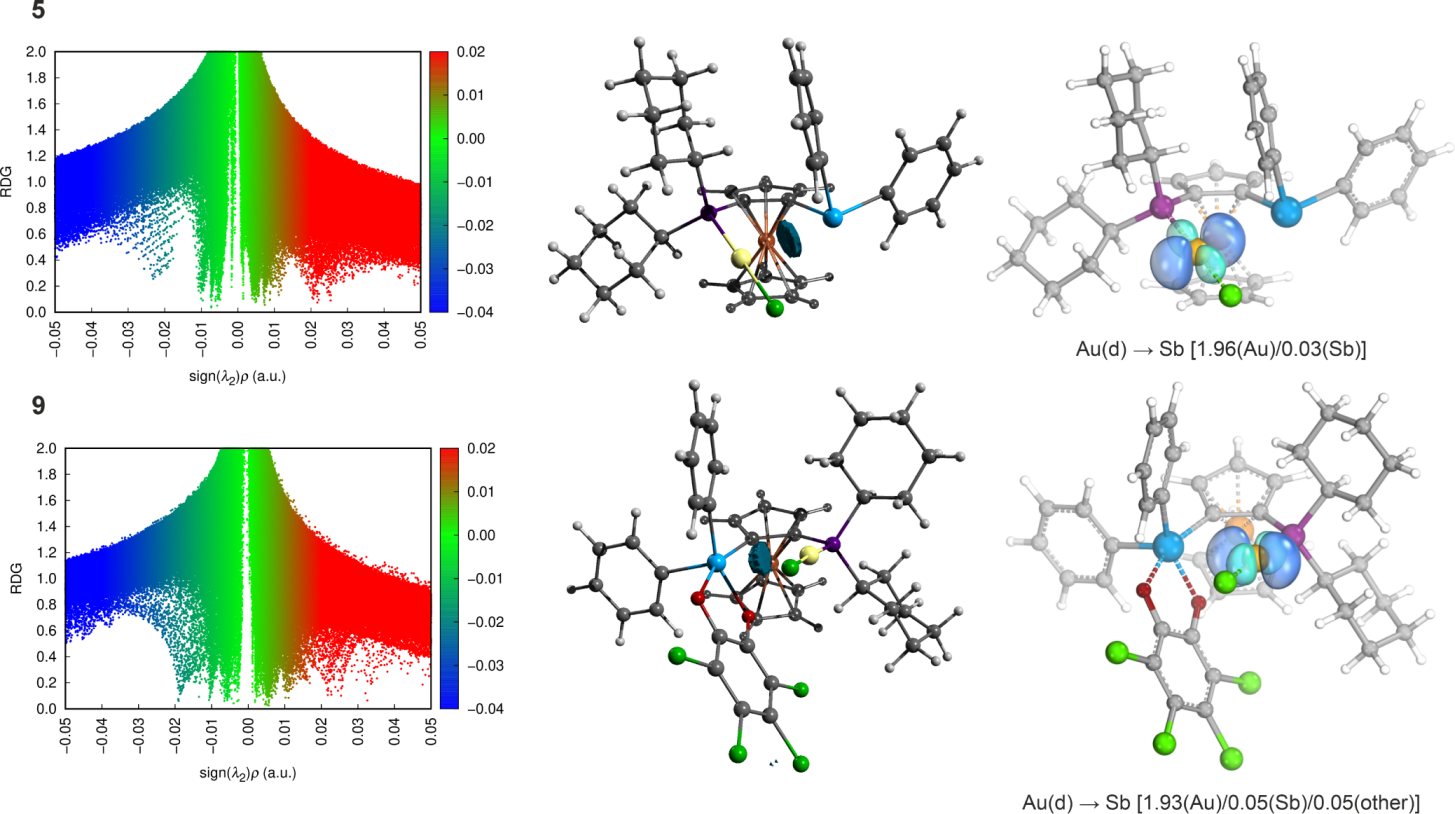
(left) Scatter plots of the reduced density gradient (RDG) versus the product of electron density and the sign of the second Hessian eigenvalue (sign­(λ_2_)­ρ) for complexes **5** and **9**; (middle) NCI plots of **5** and **9** (RDG isosurfaces with *S*(*r*) = 0.5; only regions with negative sign­(λ_2_)­ρ values in the range from –0.050 to –0.015 corresponding to attractive noncovalent interactions are shown); (right) selected IBOs for **5** and **9**. The values in parentheses indicate the fraction of bonding electrons assigned to the individual atoms.

Since IBO analysis has proven useful for qualitatively describing the bonding of various types of cationic gold­(I) complexes with carbenes,^73^ vinylidene,^74^ and allenylidene^75^ ligands, it was also applied here to rationalize the different catalytic activities of **5** and **9**. Specifically, we focused on the bonding in the hypothetical cationic gold­(I) species **5-S** and **9-S** featuring the alkyne substrate (**S**) η^2^-coordinated to gold (Scheme 6).^64^ According to IBO analysis, species **9-S** was a dicoordinate Au­(I) complex without any observable interaction between antimony and gold but with an increased P­(lp) → Au donation [1.42­(P)/0.53­(Au)/0.05­(C)]. Conversely, species **5-S** could be rather described as a tricoordinate gold­(I) species, where the P­(lp) → Au donation [1.54­(P)/0.35­(Au)/0.05­(C)] prevails over the Sb­(lp) → Au interaction [1.78­(Sb)/0.10­(Au)/0.07­(C)] (lp = lone pair; see ). The bonding between the alkyne ligand and gold atom could be described in terms of the standard Dewar–Chatt–Duncanson model,^76^ as a synergistic combination of π­(C≡C) → dσ­(Au) donation and dπ­(Au) → π*­(C≡C) back-donation.^77^ Interestingly, the comparison of the corresponding IBOs in **5-S** and **9-S** showed that the L → M donation and the M → L back-donation components were weaker in the latter species. Gold usually serves as a carbophilic Lewis acid in gold catalysis and activates unsaturated substrates by depleting their π electron density upon coordination.^78^ In the present case, the cumulative charge transferred from the alkyne ligand to the gold atom through both bonding components was significantly greater in complex **9-S** (0.11) than in complex **5-S** (0.03). In particular, the activation of the triple bond by L → M donation was offset by M → L back-donation in the latter species. This finding was consistent with complex **9-S** being a better catalyst and indicated that the extent of back-donation was the deciding factor in this case.

**Scheme 6:**
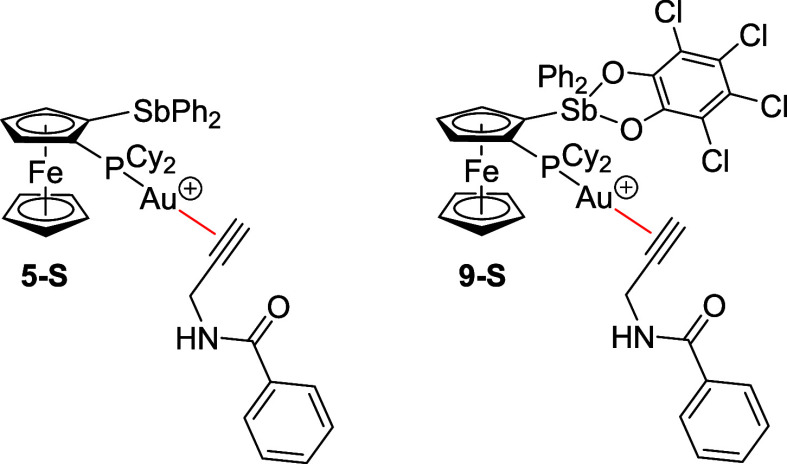
Structures of Plausible Catalytic Intermediates

To compare the structural *trans* influence^79^ of the two pnictine donor groups, we also synthesized the chelating Pd­(II) complex **12** through the reaction of [PdCl_2_(cod)] (cod = cycloocta-1,5-diene) with 1 equiv of phosphinostibine **1** (Scheme 7). The complex was isolated as an air-stable, brown solid with a 96% yield and fully characterized.

**Scheme 7:**
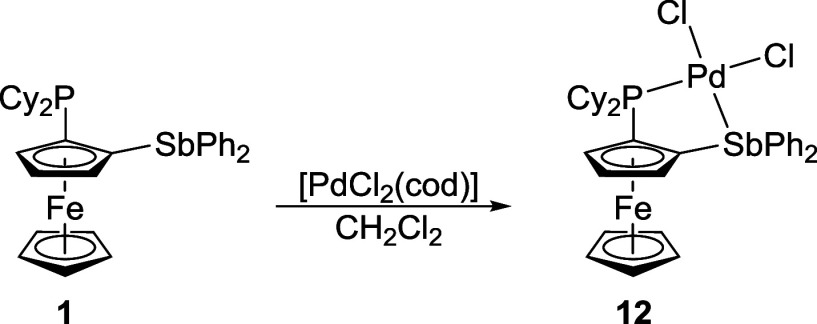
Preparation of Pd­(II) Complex **12** ^a^

a cod = cycloocta-1,5-diene.

In the ^31^P­{^1^H} NMR spectrum, the complex displayed a singlet at δ_P_ 69.3. This resonance was significantly deshielded compared to that of the structurally related complex [PdCl_2_(L-κ^2^ *P*,*P*′)] (δ_P_ 42.0), where L = 1,2-bis­(diphenylphosphino)-4-*t*-butylferrocene.^80^ Other data (NMR and ESI MS) were consistent with the proposed structure.

Palladium and its four ligating atoms in the structure of **12** (Figure 11) were coplanar within ≈0.02 Å. The two different Pd–Cl bonds were only marginally differentiated (by 0.008 Å), which could implicate a comparable *trans* influence of the two pnictogen donor groups.^81^ However, the complex showed varying interligand angles, leading to notable bending along one diagonal (P1–Pd1–Cl1 = 172.72(4)°, Sb1–Pd1–Cl2 = 179.05(3)°) and moving the Cl1 and Sb1 atoms closer to each other (Sb1···Cl1 = 3.201(1) Å). Since no similar bending was detected in the structures of similar “PdCl_2_P_2_” complexes obtained from the ferrocene diphosphines, such as 1,2-bis­(diphenylphosphino)-4-*t*-butylferrocene^80^ and 1,1′,2,2′-tetrakis­(diphenylphosphino)-4,4′-di-*t*-butylferrocene^82^ (the angles in these sterically encumbered complexes departed from 90° by less than ≈3°), the distortion of the coordination sphere in **12** was considered to reflect the intramolecular effects. Indeed, a search for noncovalent interactions using NCI analysis revealed the presence of attractive *intramolecular* contacts between atoms Sb1 and Cl1 (Figures 11 and ). This interaction was classified as a directional, Cl···Sb pnictogen bond involving an antimony atom, whose electrophilicity increased as a result of coordination.^83^

**Figure 11:**
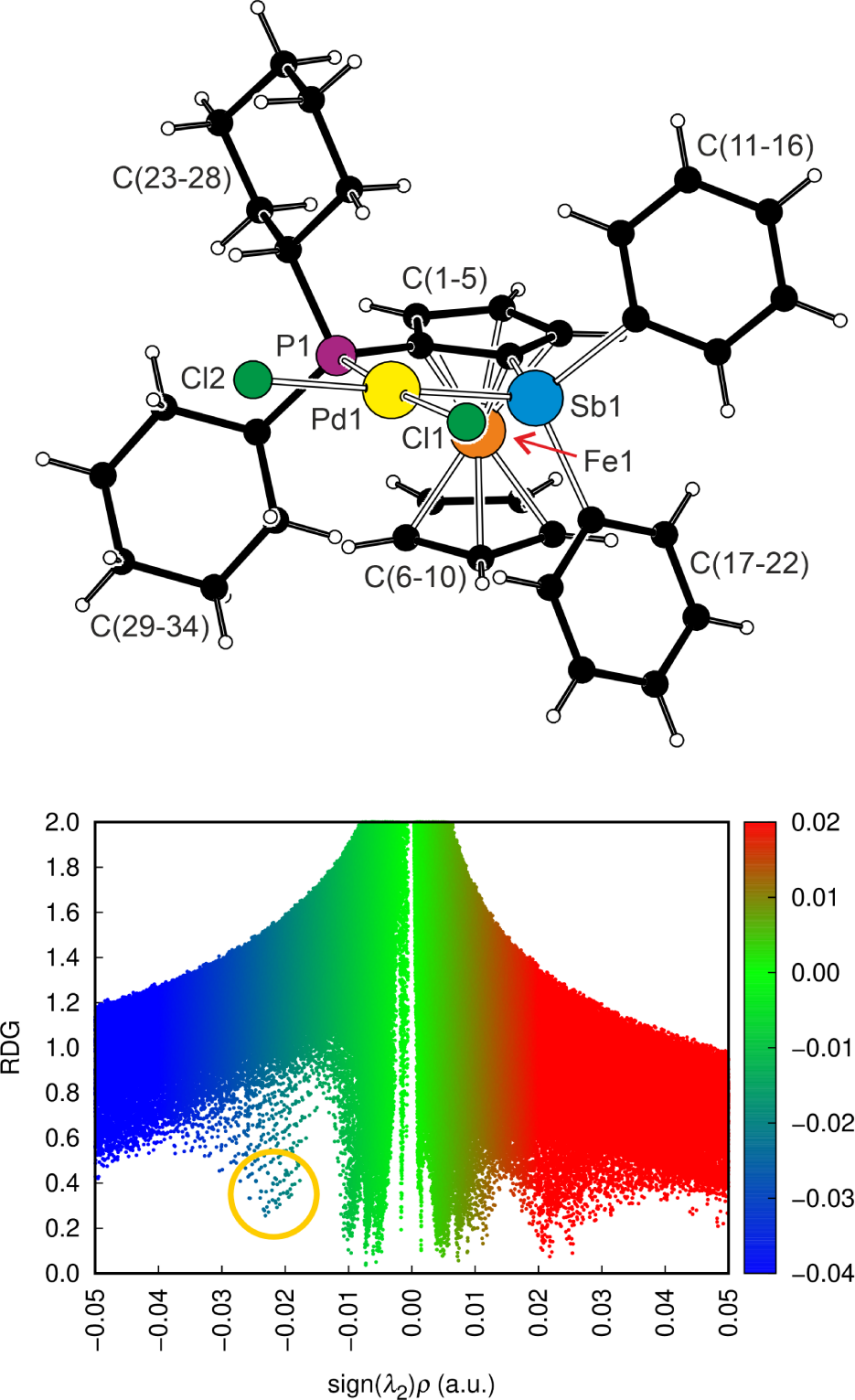
(top) Molecular structure of complex **12**. Coordination geometry parameters (in Å and deg): Pd1–Sb1 2.4595(5), Pd1–P1 2.243(1), Pd1–Cl1 2.363(1), Pd1–Cl2 2.371(1), Sb1–Pd1–P1 89.86(3), Sb1–Pd1–Cl1 83.14(4), P1–Sb1–Cl2 89.86(4), and Cl1–Pd1–Cl2 97.12(5). (bottom) Scatter plot of the reduced density gradient (RDG) against the product of the electron density and the sign of the second Hessian eigenvalue (sign­(λ_2_)­ρ) with an highlighted region of attractive interactions corresponding to Cl···Sb pnictogen bonding.

## Conclusion

Introduction of phosphine and stibine substituents into adjacent positions of the same cyclopentadienyl ring in ferrocene, such in the parent compound of this study, 1-(diphenylstibino)-2-(dicyclohexylphosphino)­ferrocene (**1**), makes the resulting compound planar-chiral and also changes its steric properties, compared to the previously studied, isomeric 1-(diphenylstibino)-1′-(dicyclohexylphosphino)­ferrocene.^21^ The steric strain in compound **1** and the corresponding phosphine chalcogenides [Fe­(η^5^-1-Ph_2_Sb-2-Cy_2_P­(E)­C_5_H_3_)­(η^5^-C_5_H_5_)] (**1E**, E = O, S, Se) hinders the formation of pnictogen interactions between the phosphine or phosphine chalcogenide moieties as the electron donors and the stibine moieties as Lewis acceptors. An increase in Lewis acidity of the Sb atom upon conversion of the stibine into stiborane moiety in compounds [Fe­(η^5^-1-Ph_2_(Cl_4_C_6_O_2_)­Sb-2-Cy_2_PC_5_H_3_)­(η^5^-C_5_H_5_)] (**3**) and [Fe­(η^5^-1-Ph_2_(Cl_4_C_6_O_2_)­Sb-2-Cy_2_P­(E)­C_5_H_3_)­(η^5^-C_5_H_5_)] (**3E**, E = O, S, Se) renders these interactions stronger and thus favors their formation (consequently, the hypervalent interactions can be detected even in compound **3** and the respective borane adduct **3**·BH_3_). DFT calculations show that the intramolecular hypervalent interactions in stiboranes gain a character of standard dative bonds between a Lewis acid and a Lewis base upon oxidation of the Sb atom. Notably, intramolecular interactions of similar types involving chlorine and gold atoms as the donors were observed in complexes featuring phosphinostibine **1** and phosphinostiborane **3** as the P-ligands; these interactions affected the molecular structures of these complexes. In contrast, the catalytic properties of Au­(I) complexes with **1** and **3** were predominantly governed by the electronic influence of the substituents.

## Supplementary Material





## Notes

Complete experimental and characterization data for the prepared compounds, details on structure determination, including a summary of crystallographic parameters, additional structure diagrams, and geometric parameters, and copies of the NMR spectra (PDF)
Coordinate files for the DFT-optimized structures (XYZ)
