# Supplementary material for: Intramolecular Interactions between the Pnictogen Groups in a Rigid Ferrocene Phosphinostibine and the Corresponding Phosphine Chalcogenides, Stiboranes, and Their Complexes
Source: Inorg Chem. 2025 May 25;64(22):11075–92. doi: 10.1021/acs.inorgchem.5c01332 (PMC12152959; doi:10.1021/acs.inorgchem.5c01332)
Supplement: Supplementary file 1 [file ic5c01332_si_001.pdf]

Supporting Information

for

Intramolecular Interactions Between the Pnictogen Groups in a Rigid Ferrocene Phosphinostibine and the Corresponding Phosphine Chalcogenides, Stiboranes and Their Complexes

David Rezazgui, Jiří Schulz and Petr Štěpnička*

Department of Inorganic Chemistry, Faculty of Science, Charles University, Hlavova 2030, 128 00 Prague, Czech Republic; E-mail: stepnic@natur.cuni.cz

Contents

Experimental	S-2
X-ray crystallography	S-19
DFT calculations	S-45
Copies of the NMR spectra	S-51
References	S-89

EXPERIMENTAL

Materials and methods

Unless specified otherwise, all syntheses were performed at ambient temperature under nitrogen atmosphere using the standard Schlenk techniques.[1] Chlorodiphenylstibine,[2] ferrocenium tetrafluoroborate,[3] and *N*-propargylbenzamide[4] were prepared according to the literature procedures. Other chemicals were obtained from commercial suppliers and used without additional purification (Sigma–Aldrich, TCI Chemicals, MCAT GmbH, and Alfa–Aesar). Anhydrous tetrahydrofuran, dichloromethane, toluene, and diethyl ether were dried using an in-house PureSolv MD5 Solvent Purification System (Innovative Technology, Inc., USA). Anhydrous tetrahydrofuran used for deprotection of **1** and **3** was degassed by three freeze-pump-thaw cycles before use.[5] Ethyl acetate and hexane mixtures utilized during purification of **1** and **3** were deoxygenated by a nitrogen flow for 30 min. Solvents used for workup, chromatography, and catalytic experiments were used as received (Lach-Ner, Czech Republic, analytical grade; deuterated solvents from ARMAR Isotopes, Germany).

The NMR spectra were recorded at 25 °C on a Bruker Avance Neo 400 MHz spectrometer, Bruker Avance III HD 400 spectrometer (^{1}H, 400.13 MHz; ^{13}C, 100.61 MHz; ^{31}P, 161.98; ^{19}F, 376.50 MHz), and a Varian UNITY Inova 400 spectrometer (^{1}H, 399.95 MHz; ^{13}C, 100.58 MHz; ^{31}P, 161.90 MHz). NMR spectra recorded during catalytic experiments and for complex **6** were obtained at 25 °C on a Bruker Avance III 600 instrument (^{1}H, 600.17 MHz; ^{13}C, 150.93 MHz; ^{31}P, 242.96 MHz). Chemical shifts (δ/ppm) are expressed relative to internal tetramethylsilane (^{1}H and ^{13}C NMR), external 85% H_3PO_4 (^{31}P), and external neat $CFCl_3$ (^{19}F). In addition to the standard notation of signal multiplicity,[6] vt and vq are used to denote virtual triplets and quartets arising from the second-order AA′BB′ and AA′BB′X spin systems (A, B = ^{1}H, X = ^{31}P) of the cyclopentadienyl rings. The mass spectra were recorded with a Waters ACQUITY QDa spectrometer using a dual orthogonal ESI with a quadrupolar analyzer. High-resolution mass spectra were acquired with Bruker Daltonics Compact QTOF-MS spectrometer. The identity of the observed ionic species was corroborated by comparing the theoretical and experimentally determined isotopic patterns. Elemental analyses were performed with a Perkin-Elmer PE 2400 CHN analyzer. The amount of residual solvent (if applicable) was verified by NMR analysis.

Syntheses

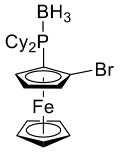

Preparation of 1-bromo-2-(dicyclohexylphosphino)ferrocene–borane (1:1) (2·BH$_3$) from bromoferrocene. The procedure was modified from ref.[7] A flame-dried Schlenk flask equipped with a magnetic stirring bar and an inert gas inlet was charged with bromoferrocene (2.64 g, 10.0 mmol) and the starting material was dissolved in anhydrous tetrahydrofuran (20 mL). The solution was cooled in a dry ice/ethanol bath to −78 °C before a solution of LiTMP was introduced dropwise. The LiTMP solution was prepared in a separate flask by adding *n*-butyllithium (6.9 mL of 1.6 M solution in hexanes, 11.0 mmol) to an ice-cooled solution of 2,2,6,6-tetramethylpiperidine (2 mL, 11.5 mmol) in 15 mL of THF and stirring at 0 °C for 30 min.

The reaction mixture was stirred at −78 °C for 3 h. Neat chlorodicyclohexylphosphine was added dropwise (2.7 mL, 12.0 mmol) and the mixture was allowed to warm to room temperature and left stirring overnight. On the following day, the mixture was cooled to 0 °C, and borane-dimethylsulfide (15 mL of 2 M solution in THF, 30.0 mmol) was slowly added. The resulting mixture was allowed to warm to room temperature while stirring for 1 h and then quenched by successively adding water (10 mL), saturated aqueous sodium hydrogencarbonate (20 mL), and diethyl ether (20 mL). The resulting mixture was transferred into a separatory funnel. The organic phase was separated, washed with brine (3× 20 mL), dried over magnesium sulfate, filtered, and evaporated under reduced pressure. The crude product was purified by flash column chromatography over silica gel using ethyl acetate-hexane (3:97) as the eluent. Yield of **2**·BH$_3$: 3.80 g (80%), orange powder. The crystal used for X-ray diffraction analysis was obtained by crystallization from hot heptane.

^{1}H NMR (CDCl$_3$, 399.95 MHz): δ 0.18-0.76 (br m, 3H, BH$_3$), 0.70-2.00 (m, 20 H, PCy$_2$), 2.54 (m, 1 H, PCy$_2$), 2.81 (m, 1 H, PCy$_2$), 4.33 (s, 5 H, C$_5$H$_5$), 4.39 (m, 1 H, CH of C$_5$H$_3$), 4.51 (m, 1 H, CH of C$_5$H$_3$), 4.65 (m, 1 H, CH of C$_5$H$_3$). ^{13}C{^{1}H} NMR (CDCl$_3$, 100.58 MHz): δ 25.89 (m, CH$_2$^{4} of PCy$_2$), 26.44 (CH$_2$^{4} of PCy$_2$), 26.53-27.18 (m, 7 CH$_2$ of PCy$_2$), 27.87 (CH$_2$ of PCy$_2$), 29.55 (d, J_{CP} = 35 Hz, CH of PCy$_2$), 33.49 (d, J_{CP} = 34 Hz, CH of PCy$_2$), 67.08 (d, J_{CP} = 51 Hz, C^{ipso}-P of C$_5$H$_3$), 69.24 (d, J_{CP} = 7 Hz, CH of C$_5$H$_3$), 72.34 (C$_5$H$_5$), 73.85 (d, J_{CP} = 13 Hz, CH of C$_5$H$_3$), 74.33 (d, J_{CP} = 4 Hz, CH of C$_5$H$_3$), 80.20 (C^{ipso}-Br of C$_5$H$_3$). ^{31}P{^{1}H} NMR (CDCl$_3$, 161.90 MHz): δ 30.0 (br d, J_{CP} = 83 Hz). ESI+ MS: *m/z* 459.90, 461.37 ([M − BH$_3$]$^+$), 473.08, 475.03 ([M − H]$^+$). Anal. Calc. for C$_{22}$H$_{33}$BBrFeP (491.3): C 55.62, H 7.00%. Found: C 55.61, H 7.06%.

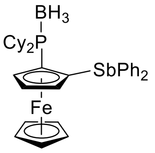

Preparation of 1-(diphenylstibino)-2-(dicyclohexylphosphino)ferrocene–borane (1:1) (1·BH_3) from 2·BH_3. A flame-dried Schlenk flask equipped with a magnetic stirring bar and an inert gas inlet was charged with **2**·BH_3 (6.00 g, 12.7 mmol). The starting material was dissolved in anhydrous tetrahydrofuran (40 mL) and the resulting solution was cooled in a dry ice/ethanol bath to −78 °C before *n*-butyllithium (5.6 mL of 2.5 M solution in hexanes, 14.0 mmol) was added dropwise. During the addition, the reaction mixture turned deep red. After stirring at −78 °C for 2 h, a solution of chlorodiphenylstibine in THF (4.8 g, 15.4 mmol in 20 mL), cooled to −78 °C, was transferred to the reaction mixture via a cannula. The resulting mixture was allowed to warm to room temperature and left to stir overnight. Then, it was quenched by adding saturated aqueous sodium hydrogencarbonate (20 mL) and diethyl ether (20 mL). The mixture was transferred into a separatory funnel. The organic phase was separated, washed with brine (3× 20 mL), dried over magnesium sulfate, filtered, and evaporated under reduced pressure. The crude product was purified by flash column chromatography over silica gel using ethyl acetate-hexane (3:97) as the eluent and further crystallized from hot heptane. Yield of **1**·BH_3: 1.70 g (20%), orange crystals.

^{1}H NMR ($CDCl_3$, 399.95 MHz): δ 0.30-0.71 (br m, 3 H, BH_3), 0.72-1.93 (m, 20 H, PCy_2), 2.16 (m, 1 H, PCy_2), 2.37 (m, 1 H, PCy_2), 4.10 (m, 1 H, CH of C_5H_3), 4.13 (s, 5 H, C_5H_5), 4.57 (m, 1 H, CH of C_5H_3), 4.67 (m, 1 H, CH of C_5H_3), 7.28-7.33 (m, 3 H, $SbPh_2$), 7.34-7.39 (m, 3 H, $SbPh_2$), 7.46-7.52 (m, 2 H, $SbPh_2$), 7.55-7.60 (m, 2 H, $SbPh_2$). ^{13}C{^{1}H} NMR ($CDCl_3$, 100.58 MHz): δ 25.76 (d, J_{CP} = 1 Hz, CH_2^4 of PCy_2), 25.84 (d, J_{CP} = 1 Hz, CH_2^4 of PCy_2), 26.42 (CH_2 of PCy_2), 26.58 (d, J_{CP} = 5 Hz, CH_2 of PCy_2), 26.63-26.79 (m, 2 CH_2 of PCy_2), 26.84 (d, J_{CP} = 2 Hz, CH_2 of PCy_2), 27.10 (d, J_{CP} = 4 Hz, CH_2 of PCy_2), 27.24 (CH_2 of PCy_2), 27.97 (CH_2 of PCy_2), 32.35 (d, J_{CP} = 34 Hz, CH of PCy_2), 33.04 (d, $^1J_{CP}$ = 34 Hz, CH of PCy_2), 70.57 (C_5H_5), 72.70 (d, J_{CP} = 9 Hz, C^{ipso}-Sb of C_5H_3), 73.13 (d, J_{CP} = 7 Hz, CH of C_5H_3), 73.90 (d, J_{CP} = 55 Hz, C^{ipso}-P of C_5H_3), 78.79 (d, J_{CP} = 7 Hz, CH of C_5H_3), 128.58 (CH^{para} of $SbPh_2$), 128.62 (CH^{ortho} of $SbPh_2$), 128.73 (CH^{ortho} of $SbPh_2$), 128.81 (CH^{para} of $SbPh_2$), 136.22 (CH^{meta} of $SbPh_2$), 136.61 (CH^{meta} of $SbPh_2$), 138.30 (C^{ipso} of $SbPh_2$), 139.33 (C^{ipso} of $SbPh_2$); one signal due to CH of C_5H_3 was obscured by the solvent resonance. ^{31}P{^{1}H} NMR ($CDCl_3$, 161.90 MHz): δ 28.7 (br d, J_{BP} = 71 Hz). ESI+ MS: *m/z* 656.07, 658.09 ([M – BH_3]$^+$), 669.05, 671.08 ([M – H]$^+$), 687.08, 689.13 ([M – BH_3 + MeO]$^+$). Anal. Calc. for $C_{34}H_{43}BFePSb$ (671.1): C 60.85, H 6.46%. Found: C 60.91, H 6.48%.

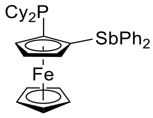

Preparation of 1-(diphenylstibino)-2-(dicyclohexylphosphino)ferrocene (1) from 1·BH_3. A flame-dried Schlenk flask equipped with a magnetic stirring bar and an inert gas inlet was charged with **1**·BH_3 (671 mg, 1.0 mmol) and 1,4-diazabicyclo[2.2.2]octane (dabco; 449 mg, 4.0 mmol). The starting materials were dissolved in degassed anhydrous THF (10 mL) and the reaction mixture was left to react at 60 °C overnight. Then, it was evaporated under reduced pressure and the residue was dissolved in a mixture of deoxygenated hexane and ethyl acetate (10:1). The solution was filtered through a short, nitrogen-flushed silica column and the filtrate was evaporated under reduced pressure yielding pure product as an orange oil. In subsequent reactions, **1** was used in the form of 0.1 M solution in anhydrous dichloromethane. The product readily crystallized from boiling heptane. Yield of **1**: 657 mg (quant.), orange oil.

^{1}H NMR (CD_2Cl_2, 400.13 MHz): δ 0.53-1.04 (m, 5 H, PCy_2), 1.20-1.55 (m, 11 H, PCy_2), 1.68-1.77 (m, 1 H, PCy_2), 1.79-1.89 (m, 2 H, PCy_2), 1.93-2.02 (m, 1 H, PCy_2), 2.05-2.15 (m, 1 H, PCy_2), 2.25-2.35 (m, 1 H, PCy_2), 4.02 (s, 5 H, C_5H_5), 4.06 (m, 1 H, CH of C_5H_3), 4.36 (m, 1 H, CH of C_5H_3), 4.48 (m, 1 H, CH of C_5H_3), 7.24-7.30 (m, 3 H, $SbPh_2$), 7.31-7.36 (m, 3 H, $SbPh_2$), 7.47-7.53 (m, 2 H, $SbPh_2$), 7.58-7.65 (m, 2 H, $SbPh_2$). ^{13}C{^{1}H} NMR (CD_2Cl_2, 100.61 MHz): δ 26.48 (CH_2^4 of PCy_2), 26.94 (CH_2^4 of PCy_2), 27.33 (d, J_{CP} = 9 Hz, CH_2 of PCy_2), 27.57 (d, J_{CP} = 10 Hz, CH_2 of PCy_2), 27. 84 (d, J_{CP} = 7 Hz, CH_2 of PCy_2), 28.28 (d, J_{CP} = 14 Hz, CH_2 of PCy_2), 30.26 (d, J_{CP} = 12 Hz, CH_2 of PCy_2), 30.60 (d, J_{CP} = 6 Hz, CH_2 of PCy_2), 31.66 (d, J_{CP} = 13 Hz, CH_2 of PCy_2), 33.36 (d, J_{CP} = 21 Hz, CH_2 of PCy_2), 35.43 (d, J_{CP} = 12 Hz, CH of PCy_2), 37.31 (d, J_{CP} = 14 Hz, CH of PCy_2), 69.96 (C_5H_5), 72.68 (CH of C_5H_3), 73.26(d, J_{CP} = 4 Hz, CH of C_5H_3), 76.03 (d, J_{CP} = 7Hz, CH of C_5H_3), 77.77 (d, J_{CP} = 37 Hz, C^{ipso}-P of C_5H_3), 86.35 (d, J_{CP} = 15 Hz C^{ipso}-Sb of C_5H_3), 128.55 (2 CH^{para} of $SbPh_2$), 128.73 (CH^{ortho} of $SbPh_2$), 128.88 (CH^{ortho} of $SbPh_2$), 136.62 (CH^{meta} of $SbPh_2$), 137.20 (CH^{meta} of $SbPh_2$), 138.75 (d, J_{CP} = 10 Hz, C^{ipso} of $SbPh_2$), 140.83 (C^{ipso} of $SbPh_2$). ^{31}P{^{1}H} NMR (CD_2Cl_2, 161.98 MHz): δ −7.9 (s). ESI+ MS: *m/z* 657.05, 658.99 ($[M + H]^+$). Anal. Calc. for $C_{34}H_{40}FePSb\cdot 1/6CH_2Cl_2$ (671.4): C 61.12, H 6.05%. Found: C 61.16, H 6.01%.

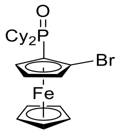

Preparation of 1-bromo-2-(dicyclohexylphosphinoyl)ferrocene (2O) from 2·BH_3. A flame-dried Schlenk flask equipped with a magnetic stirring bar and a gas inlet was charged with **2**·BH_3 (475 mg, 1.0 mmol) and dabco (448 mg, 4.0 mmol) and the solid educts were in anhydrous THF (10 mL). The reaction mixture was heated to 60 °C overnight and then evaporated under reduced

pressure. The residue was dissolved in a mixture of hexane and ethyl acetate (5:1) and the solution was filtered through a short silica column. The filtrate was evaporated under reduced pressure and dissolved in 40 mL of reagent-grade acetone and the solution was cooled to 0° C in an ice bath. Hydrogen peroxide (0.5 ml of 30% solution, ca. 4.9 mmol) was introduced and the reaction mixture was allowed to warm to room temperature while stirring for 1 h. The excess of hydrogen peroxide was destroyed by the addition of saturated aqueous sodium thiosulfate solution (2 mL) and the resulting mixture was concentrated under reduced pressure. The residue was partitioned between dichloromethane and water. The organic phase was separated, washed with brine (3×10 mL), dried over magnesium sulfate, filtered, and evaporated under reduced pressure leaving pure **2O**. Yield of **2O**: 439 mg (92%), brown powder.

Safety note: although we have not encountered any problems, it should be kept in mind that concentrated hydrogen peroxide solutions are strong oxidants and may cause a fire or explosion and that hydrogen peroxide can cause severe damage to skin and eyes.

^{1}H NMR ($CDCl_3$, 400.13 MHz): δ 0.81-1.46 (m, 10 H, PCy_2), 1.57-1.83 (m, 4 H, PCy_2), 1.86-2.15 (m, 3 H, PCy_2), 2.24-2.55 (m, 2 H, PCy_2), 2.88-3.01 (m, 3 H, PCy_2), 4.36 (s, 5 H, C_5H_5), 4.41 (m, 1 H, CH of C_5H_3), 4.57 (m, 1 H, CH of C_5H_3), 4.67 (m, 1 H, CH of C_5H_3). ^{13}C{^{1}H} NMR ($CDCl_3$, 100.61 MHz): δ 24.99 (d, J_{CP} = 3 Hz, CH_2^{4} PCy_2), 25.24 (d, J_{CP} = 4 Hz, CH_2^{4} of PCy_2), 25.79 (d, J_{CP} = 1 Hz, CH_2 of PCy_2), 25.88 (d, J_{CP} = 2 Hz, CH_2 of PCy_2), 26.07 (d, J_{CP} = 2 Hz, CH_2 of PCy_2), 26.20-26.60 (m, 4 CH_2 of of PCy_2), 26.73 (d, J_{CP} = 14 Hz, CH_2 of PCy_2), 35.57 (d, J_{CP} = 69 Hz, CH of PCy_2), 37.23 (d, J_{CP} = 70 Hz, CH of PCy_2), 69.32 (d, J_{CP} = 8 Hz, CH of C_5H_3), 72.14 (d, J_{CP} = 9 Hz, CH of C_5H_3), 72.27 (C_5H_5), 74.18 (d, J_{CP} = 7 Hz, CH of C_5H_3), 78.59 (d, J_{CP} = 10 Hz, C^{ipso}-P of C_5H_3); the signal due to C^{ipso}-Br of C_5H_3 was not observed. ^{31}P{^{1}H} NMR ($CDCl_3$, 161.98 MHz): δ 50.9 (s). ESI+ MS: *m*/*z* 477.02, 478.95 ($[M + H]^+$). Anal. Calc. for $C_{22}H_{30}BrFeOP{\cdot}1/5CH_2Cl_2$ (492.8): C 53.96, H 6.20%. Found: C 53.99, H 6.00%.

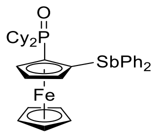

Preparation of 1-(diphenylstibino)-2-(dicyclohexylphosphinoyl)ferrocene (1O) from 2O. A flame-dried Schlenk flask equipped with a magnetic stirring bar and an inert gas inlet was charged with **2O** (126.0 mg, 0.26 mmol) and the starting material was dissolved in anhydrous tetrahydrofuran (10 mL). The resulting solution was cooled in a dry ice/ethanol bath to −78 °C before *n*-butyllithium (0.12 mL of 2.5 M solution in hexanes, 0.30 mmol) was added dropwise. During the addition, the reaction mixture turned deep red. After stirring at −78 °C for 2 h, a solution of chlorodiphenylstibine in THF (109 mg, 0.35 mmol in 10 mL), cooled to −78 °C, was transferred to the reaction mixture via a cannula, and the resulting mixture was allowed to warm

to room temperature and left to react for 2 h. The reaction was terminated by adding saturated aqueous sodium hydrogencarbonate (10 mL) and diethyl ether (10 mL) and the resulting mixture was transferred into a separatory funnel. The organic phase was separated, washed with brine (3× 10 mL), dried over magnesium sulfate, filtered, and evaporated under reduced pressure. The crude product was purified by flash column chromatography over silica gel using ethyl acetate-hexane (1:5). The product was subsequently crystallized by liquid-phase diffusion of pentane into a solution of the product in 0.2 mL of chloroform. Yield of **1O**: 35.0 mg (20%), orange powder.

^{1}H NMR ($CDCl_3$, 400.13 MHz): δ 0.73-1.03 (m, 5 H, PCy_2), 1.19-1.56 (m, 11 H, PCy_2), 1.74-1.82 (m, 1 H, PCy_2), 1.84-1.96 (m, 2 H, PCy_2), 2.18-2.42 (m, 3 H, PCy_2), 4.07 (m, 1 H, CH of C_5H_3), 4.24 (s, 5 H, C_5H_5), 4.29 (m, 1 H, CH of C_5H_3), 4.52 (m, 1 H, CH of C_5H_3), 7.23-7.28 (m, 3 H, $SbPh_2$), 7.31-7.33 (m, 3 H, $SbPh_2$), 7.53-7.57 (m, 2 H, $SbPh_2$), 7.58-7.65 (m, 2 H, $SbPh_2$). $^{13}C\{^{1}H\}$ NMR ($CDCl_3$, 100.61 MHz): δ 25.65 (d, J_{CP} = 1 Hz, CH_2 of PCy_2), 25.80 (d, J_{CP} = 3 Hz, CH_2 of PCy_2), 26.09 (d, J_{CP} = 3 Hz, CH_2 of PCy_2), 26.19-26.52 (m, 4 CH_2 of PCy_2), 26.75 (d, J_{CP} = 2 Hz, CH_2 of PCy_2), 27.07 (d, J_{CP} = 8 Hz, CH_2 of PCy_2), 27.19 (d, $^{2}J_{CP}$ = 7 Hz, CH_2 of PCy_2), 38.67 (d, J_{CP} = 66 Hz, CH of PCy_2), 41.39 (d, J_{CP} = 66 Hz, CH of PCy_2), 69.99 (C_5H_5), 73.57 (d, J_{CP} = 14 Hz, CH of C_5H_3), 73.84 (d, J_{CP} = 8 Hz, CH of C_5H_3), 75.83 (d, J_{CP} = 14 Hz, CH of C_5H_3), 77.04 (d, J_{CP} = 37 Hz, C^{ipso}-Sb of C_5H_3, the signal partly overlaps with the solvent resonance), 78.52 (d, J_{CP} = 100 Hz, C^{ipso}-P of C_5H_3), 128.02 (2 CH^{para} of $SbPh_2$), 128.26 (CH^{ortho} of $SbPh_2$), 128.29 (CH^{ortho} of $SbPh_2$), 136.60 (2 CH^{meta} of $SbPh_2$), 141.61 (C^{ipso} of $SbPh_2$), 141.65 (C^{ipso} of $SbPh_2$). $^{31}P\{^{1}H\}$ NMR ($CDCl_3$, 161.98 MHz): δ 48.7 (s). ESI+ MS: *m/z* 689.08, 691.00 ([M + OH]$^{+}$), 703.17, 705.06 ([M + MeO]$^{+}$). Anal. Calc. for $C_{34}H_{40}FePOSb$ (672.1): C 60.65, H 5.99%. Found: C 61.19, H 5.80%.

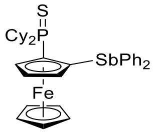

Preparation of 1S from 1. A flame-dried Schlenk flask equipped with a magnetic stirring bar and an inert gas inlet was charged with **1** (65.7 mg, 0.10 mmol) and elemental sulfur (3.2 mg, 0.10 mmol). Anhydrous toluene (5 mL) was added and the resulting solution was heated at gentle reflux for 2 h. After cooling, the solution was evaporated under reduced pressure and the resulting yellow oil was purified by column chromatography over a short silica column, eluting with dichloromethane-methanol (20:1). The product was further crystallized from boiling heptane. Yield of **1S**: 64.8 mg (94%), yellow powder.

^{1}H NMR ($CDCl_3$, 400.13 MHz): δ 0.65-2.29 (m, 22 H, PCy_2), 4.08 (m, 1 H CH of C_5H_3), 4.22 (s, 5 H, C_5H_5), 4.51 (m, 1 H, CH of C_5H_3), 4.53 (m, 1 H, CH of C_5H_3), 7.27-7-32 (m, 3 H, $SbPh_2$), 7.33-7.37 (m, 3 H, $SbPh_2$), 7.50-7.55 (m, 2 H, $SbPh_2$), 7.56-7.60 (m, 2 H, $SbPh_2$). $^{13}C\{^{1}H\}$ NMR ($CDCl_3$, 100.61 MHz): δ 25.63 (d, J_{CP} = 2 Hz, CH_2^{4} of PCy_2), 25.79 (d, J_{CP} = 2 Hz, CH_2^{4} of PCy_2), 26.02-26.86

(m, 5 CH_2 of PCy_2), 27.07 (d, J_{CP} = 3 Hz, CH_2 of PCy_2), 27.30 (d, J_{CP} = 3 Hz, CH_2 of PCy_2), 27.07 (d, J_{CP} = 3 Hz, CH_2 of PCy_2), 38.46 (d, J_{CP} = 50 Hz, CH of PCy_2), 40.81 (d, J_{CP} = 51 Hz, CH of PCy_2), 70.81 (C_5H_5), 72.87 (d, J_{CP} = 8 Hz, CH of C_5H_3), 73.31 (d, J_{CP} = 16 Hz, C^{ipso}-Sb of C_5H_3), 75.40 (d, J_{CP} = 13 Hz, CH of C_5H_3), 78.42 (d, J_{CP} = 11 Hz, CH of C_5H_3), 80.20 (d, J_{CP} = 82 Hz, C^{ipso}-P of C_5H_3), 128.37 (CH^{para} of $SbPh_2$), 128.42 (CH^{para} of $SbPh_2$), 128.47 (CH^{ortho} of $SbPh_2$), 128.67 (CH^{ortho} of $SbPh_2$), 136.42 (CH^{meta} of $SbPh_2$), 136.64 (CH^{meta} of $SbPh_2$), 140.40 (C^{ipso} of $SbPh_2$), 140.70 (C^{ipso} of $SbPh_2$). $^{31}P\{^1H\}$ NMR ($CDCl_3$, 161.98 MHz): δ 58.5 (s). ESI+ MS: *m/z* 705.04, 707.11 ($[M + OH]^+$), 719.06, 720.98 ($[M + MeO]^+$). Anal. Calc. for $C_{34}H_{40}FePSSb·1/2C_7H_{16}$ (739.4): C 60.91, H 6.54%. Found: C 61.00, H 6.08%.

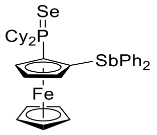

Preparation of 1Se from 1. A flame-dried Schlenk flask equipped with a magnetic stirring bar and an inert gas inlet was charged with **1** (26.3 mg, 0.040 mmol) and grey selenium (3.2 mg, 0.040 mmol). Anhydrous toluene (5 mL) was added and the mixture was refluxed for 2 h. After cooling, the solution was evaporated under reduced pressure and the resulting yellow oil was purified by column chromatography over a short silica column using dichloromethane-methanol (20:1) as the eluent. The product was further crystallized from pentane. Yield of **1Se**: 26.2 mg (89%), yellow powder.

^1H NMR ($CDCl_3$, 400.13 MHz): δ 0.71-1.05 (m, 3 H, PCy_2), 1.07-1.96 (m, 16 H, PCy_2), 2.00-2.23 (m, 3 H, PCy_2), 4.10 (m, 1 H CH of C_5H_3), 4.22 (s, 5 H, C_5H_5), 4.55 (m, 1 H, CH of C_5H_3), 4.57 (m, 1 H, CH of C_5H_3), 7.28-7.32 (m, 3 H, $SbPh_2$), 7.33-7.37 (m, 3 H, $SbPh_2$), 7.50-7.60 (m, 4 H, $SbPh_2$). $^{13}C\{^1H\}$ NMR ($CDCl_3$, 100.61 MHz): δ 25.61 (d, J_{CP} = 2 Hz, $CH_2{}^4$ of PCy_2), 25.72 (d, J_{CP} = 2 Hz, $CH_2{}^4$ of PCy_2), 26.07 (s, CH_2 of PCy_2), 26.22 (d, J_{CP} = 5 Hz, CH_2 of PCy_2), 26.39 (m, 2 CH_2 of PCy_2), 26.52 (d, J_{CP} = 7 Hz, CH_2 of PCy_2), 26.83 (d, J_{CP} = 2 Hz, CH_2 of PCy_2), 27.74 (d, J_{CP} = 3 Hz, CH_2 of PCy_2), 28.02 (d, J_{CP} = 2 Hz, CH_2 of PCy_2), 37.33 (d, J_{CP} = 43 Hz, CH of PCy_2), 40.08 (d, J_{CP} = 48 Hz, CH of PCy_2), 71.08 (C_5H_5), 72.68 (d, J_{CP} = 8 Hz, CH of C_5H_3), 73.25 (d, J_{CP} = 16 Hz, C^{ipso}-Sb of C_5H_3), 76.05 (d, J_{CP} = 13 Hz, CH of C_5H_3), 78.71(d, J_{CP} = 73 Hz, C^{ipso}-P of C_5H_3), 78.78 (d, J_{CP} = 11 Hz, CH of C_5H_3), 128.45 (CH^{para} of $SbPh_2$), 128.51 (CH^{para} and CH^{ortho} of $SbPh_2$), 128.74 (CH^{ortho} of $SbPh_2$), 136.42 (CH^{meta} of $SbPh_2$), 136.64 (CH^{meta} of $SbPh_2$), 140.14 (C^{ipso} of $SbPh_2$), 140.35 (C^{ipso} of $SbPh_2$). $^{31}P\{^1H\}$ NMR ($CDCl_3$, 161.98 MHz): δ 51.8 (s with ^{77}Se satellites, $^1J_{PSe}$ = 697 Hz). ESI+ MS: *m/z* 752.83, 755.04 ($[M + OH]^+$), 766.96, 768.89 ($[M + MeO]^+$). Anal. Calc. for $C_{34}H_{40}FePSeSb·C_5H_{12}$ (808.4): C 57.95, H 6.48%. Found: C 58.39, H 6.01%.

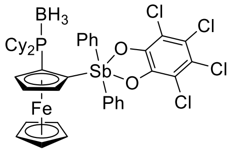

Preparation of 3·BH_3 from 1·BH_3. A flask equipped with a magnetic stirring bar was charged with **1**·BH_3 (335.6 mg, 0.50 mmol). The starting material was dissolved in anhydrous dichloromethane (10 mL) and *o*-chloranil (122.9 mg, 0.50 mmol) was added. The resulting mixture was stirred for 1 h and evaporated under reduced pressure. The crude product was crystallized by liquid diffusion of pentane into a solution in 1 mL of dichloromethane. Yield of **4**: 361.8 mg (79%), orange crystals.

^{1}H NMR ($CDCl_3$, 400.13 MHz): δ 0.19-0.51 (br m, 3H, BH_3), 0.60 (m, 2 H, PCy_2), 0.77-1.66 (m, 12 H, PCy_2), 1.69-2.10 (m, 5 H, PCy_2), 2.21 (m, 1 H, PCy_2), 2.35 (m, 2 H, PCy_2), 4.25 (s, 1 H, CH of C_5H_3), 4.52 (s, 1 H, CH of C_5H_3), 4.59 (s, 5 H, C_5H_5), 4.62 (s, 1 H, CH of C_5H_3), 7.31-7.43 (m, 3 H, $SbPh_2$), 7.43-7.58 (m, 3 H, $SbPh_2$), 7.62-7.70 (m, 2 H, $SbPh_2$), 7.70-7.79 (m, 2 H, $SbPh_2$). $^{13}C\{^{1}H\}$ NMR ($CDCl_3$, 100.61 MHz): δ 25.46 (d, J_{CP} = 2 Hz, $CH_2{}^4$ of PCy_2), 26.01 (d, J_{CP} = 1 Hz, $CH_2{}^4$ of PCy_2), 26.45 (d, J_{CP} = 12 Hz, CH_2 of PCy_2), 26.69 (d, J_{CP} = 12 Hz, CH_2 of PCy_2), 26.80 (s, CH_2 of PCy_2), 27.07 (d, J_{CP} = 10 Hz, CH_2 of PCy_2), 27.35 (s, CH_2 of PCy_2), 27.45-27.58 (m, 2 CH_2 of PCy_2), 28.48 (s, CH_2 of PCy_2), 34.16 (d, J_{CP} = 34 Hz, CH of PCy_2), 37.74 (d, J_{CP} = 34 Hz, CH of PCy_2), 71.99 (C_5H_5), 72.52 (d, J_{CP} = 8 Hz, CH of C_5H_3), 74.54 (d, J_{CP} = 4 Hz, CH of C_5H_3), 75.26 (d, J_{CP} = 10 Hz, CH of C_5H_3), 92.82 (C^{ipso} of C_5H_3, the signal was identified in the 2D spectra), 116.57 (CCl of *o*-chloranil), 119.99 (CCl of *o*-chloranil), 128.87 (CH^{ortho} of $SbPh_2$), 129.36 (CH^{ortho} of $SbPh_2$), 130.36 (CH^{para} of $SbPh_2$), 131.38 (CH^{para} of $SbPh_2$), 134.13 (CH^{meta} of $SbPh_2$), 134.71 (CH^{meta} of $SbPh_2$), 135.85 (CCl of *o*-chloranil),144.77 (C^{ipso} of $SbPh_2$), 145.59 (C^{ipso} of $SbPh_2$); the signals due to one C^{ipso} of C_5H_3 as well as one CCl and two CO signals of *o*-chloranil were not detected. $^{31}P\{^{1}H\}$ NMR ($CDCl_3$, 161.98 MHz): δ 19.7 (s). ESI+ MS: *m/z* 916.97, 918.64 ([M + H]$^+$). Anal. Calc. for $C_{40}H_{43}BCl_4FeO_2PSb$ (917.0): C 52.39, H 4.73%. Found: C 52.08, H 4.49%.

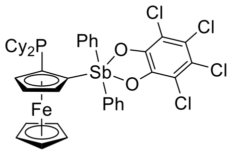

Preparation of 3 from 3·BH_3. A flame-dried Schlenk flask equipped with a magnetic stirring bar and an inert gas inlet was charged with **1**·BH_3 (342.0 mg, 0.37 mmol) and dabco (167.4 mg, 1.49 mmol). The solid educts were dissolved in degassed anhydrous tetrahydrofuran (20 mL) and the reaction mixture was warmed to 60 °C overnight. The resulting mixture was evaporated under reduced pressure and the crude product was dissolved in a mixture of deoxygenated hexane and ethyl acetate (3:1). The solution was filtered through a short, nitrogen-flushed silica column. The

filtrate was then evaporated under reduced pressure and further purified by crystallization from boiling heptane under nitrogen. Yield of **3**: 107.8 mg (32%), yellow crystals.

^{1}H NMR ($CDCl_3$, 400.13 MHz): δ 0.54-1.55 (m, 17 H, PCy_2), 1.57-1.79 (m, 2 H, PCy_2), 1.79-1.92 (m, 1 H, PCy_2), 2.12-2.28 (m, 2 H, PCy_2), 4.37 (t, *J'* = 2 Hz, 1 H, CH of C_5H_3), 4.56 (m, 2 H, CH of C_5H_3), 4.63 (s, 5 H, C_5H_5), 7.31-7.38 (m, 3 H, $SbPh_2$), 7.40-7.51 (m, 3 H, $SbPh_2$), 7.76-7.82 (m, 2 H, $SbPh_2$), 7.84-7.89 (m, 2 H, $SbPh_2$). ^{13}C{^{1}H} NMR ($CDCl_3$, 100.61 MHz): δ 25.47 (d, J_{CP} = 2 Hz, $CH_2{}^4$ of PCy_2), 26.20 (d, J_{CP} = 1 Hz, $CH_2{}^4$ of PCy_2), 26.64-27.05 (m, 3 CH_2 of PCy_2), 27.39 (d, J_{CP} = 15 Hz, CH_2 of PCy_2), 28.64 (d, J_{CP} = 8 Hz, CH_2 of PCy_2), 29.32 (d, J_{CP} = 4 Hz, CH_2 of PCy_2), 30.42 (d, J_{CP} = 12 Hz, CH_2 of PCy_2), 31.06 (d, J_{CP} = 12 Hz, CH_2 of PCy_2), 32.61 (d, J_{CP} = 3 Hz, CH of PCy_2), 39.66 (d, J_{CP} = 6 Hz, CH of PCy_2), 71.05 (CH of C_5H_3), 71.10 (C_5H_5), 71.37 (d, J_{CP} = 13 Hz, C^{ipso}-Sb of C_5H_3), 72.38 (d, J_{CP} = 5 Hz, CH of C_5H_3), 73.68 (d, J_{CP} = 2 Hz, CH of C_5H_3), 79.99 (d, J_{CP} = 12 Hz, C^{ipso}-P of C_5H_3), 109.18 (CCl of *o*-chloranil), 110.26 (CCl of *o*-chloranil), 116.91 (CCl of *o*-chloranil), 119.56 (CCl of *o*-chloranil), 128.86 (CH^{ortho} of $SbPh_2$), 129.16 (CH^{ortho} of $SbPh_2$), 130.10 (CH^{para} of $SbPh_2$), 130.85 (CH^{para} of $SbPh_2$), 134.00 (CH^{meta} of $SbPh_2$), 134.48 (CH^{meta} of $SbPh_2$), 139.05 (d, J_{CP} = 6 Hz, C^{ipso} of $SbPh_2$), 145.31 (m, 2 CO of *o*-chloranil), 146.56 (d, J_{CP} = 18 Hz, C^{ipso} of $SbPh_2$). ^{31}P{^{1}H} NMR ($CDCl_3$, 161.98 MHz): δ 26.4 (s). ESI+ MS: *m/z* 902.62, 904.79 ([M + H]$^+$). Anal. Calc. for $C_{40}H_{40}Cl_4FeO_2PSb{\cdot}1/3C_7H_{16}$ (936.5) C 54.29, H 4.88%. Found C 54.11, H 4.89%.

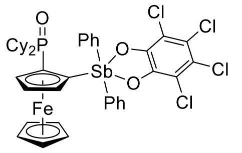

Preparation of 3O from 1O. A flask equipped with a magnetic stirring bar was charged with **1O** (12.0 mg, 0.02 mmol). The starting material was dissolved in anhydrous dichloromethane (5 mL) and *o*-chloranil (4.4 mg, 0.02 mmol) was added. The resulting mixture was stirred for 1 h and evaporated under reduced pressure. The crude product was crystallized by liquid-phase diffusion of pentane into a solution of the compound in 0.2 mL of chloroform. Yield of **3O**: 13.2 mg (78%), orange crystals.

^{1}H NMR ($CDCl_3$, 400.13 MHz): δ 0.10-0.26 (m, 1 H, PCy_2), 0.67-1.07 (m, 7 H, PCy_2), 1.12-1.67 (m, 8 H, PCy_2), 1.70-2.24 (m, 5 H, PCy_2), 2.69 (m, 1 H, PCy_2), 4.34 (m, 1 H CH of C_5H_3), 4.57 (m, 1 H, CH of C_5H_3), 4.66 (m, 1 H, CH of C_5H_3), 4.68 (s, 5 H, C_5H_5), 7.26-7.32 (m, 3 H, $SbPh_2$), 7.33-7.39 (m, 3 H, $SbPh_2$), 7.73-7.82 (m, 4 H, $SbPh_2$). ^{13}C{^{1}H} NMR ($CDCl_3$, 100.61 MHz): δ 24.65 (d, J_{CP} = 3 Hz, CH_2 of PCy_2), 24.98-25.24 (m, 3 CH_2 of PCy_2), 25.74-26.45 (m, 6 CH_2 of PCy_2), 36.07 (d, J_{CP} = 64 Hz, CH of PCy_2), 40.42 (d, J_{CP} = 65 Hz, CH of PCy_2), 69.39 (d, J_{CP} = 13 Hz, CH of C_5H_3), 71.42 (C_5H_5),73.38 (d, J_{CP} = 10 Hz, CH of C_5H_3), 74.75 (d, J_{CP} = 7 Hz, CH of C_5H_3), 93.76 (d, J_{CP} = 13 Hz, C^{ipso}-P of C_5H_3), 114.99 (CCl of *o*-chloranil), 116.45 (CCl of *o*-chloranil), 118.39 (CCl of *o*-chloranil),

118.65 (CCl of *o*-chloranil), 128.09 (CH^{ortho} of $SbPh_2$), 128.65 (CH^{ortho} of $SbPh_2$), 129.23 (CH^{para} of $SbPh_2$), 129.87 (CH^{para} of $SbPh_2$), 133.81 (CH^{meta} of $SbPh_2$), 134.03 (CH^{meta} of $SbPh_2$), 143.23 (C^{ipso} of $SbPh_2$), 146.05 (C^{ipso} of $SbPh_2$), 146.50 (CO of *o*-chloranil), 146.69 (CO of *o*-chloranil); the signal due to C^{ipso}-Sb of C_5H_3 was not detected. $^{31}P\{^1H\}$ NMR ($CDCl_3$, 161.98 MHz): δ 58.5 (s). ESI+ MS: *m/z* 918.83, 920.44 ($[M + H]^+$). Anal. Calc. for $C_{40}H_{40}Cl_4FeO_3PSb$ (919.1): C 52.27, H 4.39%. Found: C 52.86, H 4.30%.

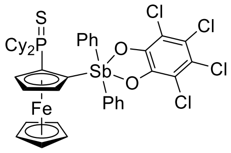

Preparation of 3S from 1S. A flask equipped with a magnetic stirring bar was charged with **1S** (64.8 mg, 0.01 mmol). The starting material was dissolved in anhydrous dichloromethane (5 mL) and *o*-chloranil (23.1 mg, 0.01 mmol) was added. The resulting mixture was stirred for 1 h and evaporated under reduced pressure. The crude product was crystallized by liquid-phase diffusion of pentane into a solution of the compound in 0.4 mL of chloroform. Yield of **3S**: 73.0 mg (83%), orange crystals.

^1H NMR ($CDCl_3$, 400.13 MHz): δ 0.46-0.63 (m, 1 H, PCy_2), 0.74-1.72 (m, 14 H, PCy_2), 1.74-1.97 (m, 3 H, PCy_2), 2.05 (m, 1 H, PCy_2), 2.18 (m, 1 H, PCy_2), 2.43 (m, 1 H, PCy_2), 2.57 (m, 1 H, PCy_2), 4.39 (m, 1 H, CH of C_5H_3), 4.43 (m, 1 H, CH of C_5H_3), 4.60 (s, 5 H, C_5H_5), 4.65 (m, 1 H, CH of C_5H_3), 7.28-7.43 (m, 6 H, $SbPh_2$), 7.61-7.66 (m, 2 H, $SbPh_2$), 7.69-7.75 (m, 2 H, $SbPh_2$). $^{13}C\{^1H\}$ NMR ($CDCl_3$, 100.61 MHz): δ 25.06 (m, 2 CH_2 of PCy_2), 25.89 (s, CH_2 of PCy_2), 26.03 (d, J_{CP} = 2 Hz, CH_2 of PCy_2), 26.27 (d, J_{CP} = 2 Hz, CH_2 of PCy_2), 26.49-26.93 (m, 4 CH_2 of PCy_2), 27.60 (s, CH_2 of PCy_2), 37.83 (d, J_{CP} = 47 Hz, CH of PCy_2), 43.01 (d, J_{CP} = 47 Hz, CH of PCy_2), 71.11 (d, J_{CP} = 12 Hz, CH of C_5H_3), 71.78 (C_5H_5), 74.33 (d, J_{CP} = 12 Hz, CH of C_5H_3), 75.20 (d, J_{CP} = 7 Hz, CH of C_5H_3), 96.31 (C^{ipso} of C_5H_3, the signal was identified in the 2D spectra), 119.10 (CCl of *o*-chloranil), 119.23 (CCl of *o*-chloranil), 128.30 (CH^{ortho} of $SbPh_2$), 128.69 (CH^{ortho} of $SbPh_2$), 129.38 (CH^{para} of $SbPh_2$), 130.09 (CH^{para} of $SbPh_2$), 133.71 (CH^{meta} of $SbPh_2$), 134.22 (CH^{meta} of $SbPh_2$), 136.43 (CCl of *o*-chloranil), 136.65 (CCl of *o*-chloranil), 141.03 (C^{ipso} of $SbPh_2$), 145.50 (CO of *o*-chloranil), 146.11 (CO of *o*-chloranil), 148.82 (C^{ipso} of $SbPh_2$); one signal due to C^{ipso} of C_5H_3 was not observed. $^{31}P\{^1H\}$ NMR ($CDCl_3$, 161.98 MHz): δ 58.6 (s). ESI+ MS: *m/z* 934.86, 936.62 ($[M + H]^+$). Anal. Calc. for $C_{40}H_{40}Cl_4FeO_2PSSb$ (935.2): C 51.37, H 4.31%. Found: C 51.30, H 4.25%.

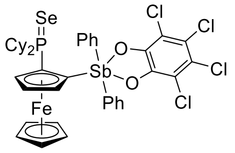

Preparation of 3Se from 1Se. A flask equipped with a magnetic stirring bar was charged with **1Se** (26.2 mg, 0.04 mmol). The starting material was dissolved in anhydrous dichloromethane (5 mL) and *o*-chloranil (8.9 mg, 0.04 mmol) was added. The resulting mixture was stirred for 1 h and evaporated under reduced pressure. The crude product was crystallized by liquid-phase diffusion of pentane into a solution of the compound in 0.4 mL of chloroform. Yield of **3Se**: 27.9 mg (79%), orange crystals.

^{1}H NMR ($CDCl_3$, 400.13 MHz): δ 0.53-1.74 (m, 14 H, PCy_2), 1.78-1.97 (m, 4 H, PCy_2), 2.05 (m, 1 H, PCy_2), 2.28 (m, 1 H, PCy_2), 2.45-2.74 (m, 2 H, PCy_2) 4.40 (m, 2 H, CH of C_5H_3), 4.58 (s, 5 H, C_5H_5), 4.69 (m, 1 H, CH of C_5H_3), 7.27-7.41 (m, 6 H, $SbPh_2$), 7.58-7.65 (m, 2 H, $SbPh_2$), 7.67-7.74 (m, 2 H, $SbPh_2$). $^{13}C\{^1H\}$ NMR ($CDCl_3$, 100.61 MHz): δ 25.63 (s, CH_2 of PCy_2), 25.73 (d, J_{CP} = 3 Hz, CH_2 of PCy_2), 25.84 (d, J_{CP} = 3 Hz, CH_2 of PCy_2), 25.99 (d, J_{CP} = 5 Hz, CH_2 of PCy_2), 26.15-27.32 (m, 4 CH_2 of PCy_2), 28.95 (s, CH_2 of PCy_2), 29.74 (s, CH_2 of PCy_2), 37.33 (d, J_{CP} = 41 Hz, CH of PCy_2), 43.13 (d, J_{CP} = 40 Hz, CH of PCy_2), 70.96 (d, J_{CP} = 11 Hz, CH of C_5H_3), 71.83 (C_5H_5), 72.28 (d, J_{CP} = 10 Hz, C^{ipso}-Sb of C_5H_3), 74.69 (d, J_{CP} = 12 Hz, CH of C_5H_3), 75.52 (d, J_{CP} = 7 Hz, CH of C_5H_3), 98.57 (d, J_{CP} = 20 Hz, C^{ipso}-P of C_5H_3), 116.44 (CCl of *o*-chloranil), 117.24 (CCl of *o*-chloranil), 119.08 (CCl of *o*-chloranil), 119.19 (CCl of *o*-chloranil), 128.26 (CH^{ortho} of $SbPh_2$), 128.62 (CH^{ortho} of $SbPh_2$), 129.29 (CH^{para} of $SbPh_2$), 129.93 (CH^{para} of $SbPh_2$), 133.55 (CH^{meta} of $SbPh_2$), 134.30 (CH^{meta} of $SbPh_2$), 141.38 (C^{ipso} of $SbPh_2$), 145.48 (CO of *o*-chloranil), 146.06 (CO of *o*-chloranil), 149.73 (C^{ipso} of $SbPh_2$). $^{31}P\{^1H\}$ NMR ($CDCl_3$, 161.98 MHz): δ 54.0 (s with ^{77}Se satellites, $^1J_{PSe}$ = 599 Hz). ESI+ MS: *m/z* 982.64, 980.71, 984.71 ($[M + H]^+$). Anal. Calc. for $C_{40}H_{40}Cl_4FeO_2PSeSb$ (982.1): C 48.92, H 4.11%. Found: C 49.27, H 4.10%.

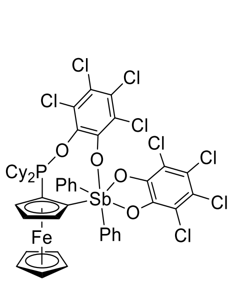

Preparation of 4 from 1. A flask equipped with a magnetic stirring bar was charged with **1** (65.7 mg, 0.10 mmol). The starting material was dissolved in anhydrous dichloromethane (5 mL) and *o*-chloranil (24.6 mg, 0.10 mmol) was added. The resulting mixture was stirred for 1 h and evaporated under reduced pressure. The crude product was crystallized by liquid-phase diffusion of pentane into a solution of the compound in 0.4 mL of dichloromethane. Yield of **4**: 25.3 mg (44%), orange crystals.

^1H NMR ($CDCl_3$, 400.13 MHz): δ 0.50-1.04 (m, 4 H, PCy_2), 1.09-1.67 (m, 10 H, PCy_2), 1.87-2.20 (m, 4 H, PCy_2), 2.30 (m, 1 H, PCy_2), 2.78 (m, 1 H, PCy_2), 3.04 (m, 1 H, PCy_2), 3.31 (m, 1 H, PCy_2), 4.48 (m, 1 H, CH of C_5H_3), 4.54 (s, 5 H, C_5H_5), 4.68 (m, 1 H, CH of C_5H_3), 4.79 (vq, J' = 3 Hz, 1 H, CH of C_5H_3), 7.26-7.33 (m, 3 H, $SbPh_2$), 7.35-7.41 (m, 3 H, $SbPh_2$), 7.42-7.48 (m, 2 H, $SbPh_2$), 7.86-7.93 (m, 2 H, $SbPh_2$). $^{13}C\{^1H\}$ NMR ($CDCl_3$, 100.61 MHz): δ 25.35-25.49 (m, 2 CH_2^4 of PCy_2), 26.28 (d, J_{CP} = 13 Hz, PCy_2), 26.55 (d, J_{CP} = 16 Hz, PCy_2), 26.74-27.16 (m, 4 CH_2 of PCy_2), 27.72 (d, J_{CP} = 5 Hz, CH_2 of PCy_2), 27.95 (d, J_{CP} = 2 Hz, CH_2 of PCy_2), 39.80 (d, J_{CP} = 62 Hz, CH of PCy_2), 41.98 (d, J_{CP} = 46 Hz, CH of PCy_2), 65.98 (d, J_{CP} = 111 Hz, C^{ipso}-P of C_5H_3), 72.77 (C_5H_5), 74.30 (d, J_{CP} = 12 Hz, CH of C_5H_3), 75.63 (d, J_{CP} = 23 Hz, CH of C_5H_3), 81.40 (d, J_{CP} = 13 Hz, CH of C_5H_3), 91.18 (d, J_{CP} = 13 Hz C^{ipso}-Sb of C_5H_3), 114.51 (CCl of *o*-chloranil), 115.32 (CCl of *o*-chloranil), 117.57 (CCl of *o*-chloranil), 119.33 (CCl of *o*-chloranil), 120.66 (CCl of *o*-chloranil), 126.67 (CCl of *o*-chloranil), 128.10 (CH^{ortho} of $SbPh_2$), 128.4 (CH^{ortho} of $SbPh_2$), 129.03 (CH^{para} of $SbPh_2$), 129.16 (CH^{para} of $SbPh_2$), 132.98 (CH^{meta} of $SbPh_2$), 133.65 (CH^{meta} of $SbPh_2$), 139.85 (CO of *o*-chloranil), 139.95 (CO of *o*-chloranil), 145.44 (CCl of *o*-chloranil), 146.74 (2 C^{ipso} of $SbPh_2$), 147.71 (CCl of *o*-chloranil), 148.94 (CO of *o*-chloranil), 148.96 (CO of *o*-chloranil). $^{31}P\{^1H\}$ NMR (CD_2Cl_2, 161.98 MHz): δ 93.5 (s). ESI+ MS: *m/z* 1148.57, 1149.99 ($[M + H]^+$), 902.75, 904.80 ($[M - C_6Cl_4O_2 + H]^+$). Anal. Calc. for $C_{46}H_{40}Cl_8FeO_4PSb$ (1149.0): C 48.08, H 3.51%. Found: C 47.93, H 3.61%.

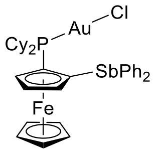

Preparation of 5. A flask equipped with a magnetic stirring bar was charged with **1** (65.7 mg, 0.10 mmol). The starting material was dissolved in anhydrous dichloromethane (5 mL) and chloro(dimethylsulfide)gold(I) (29.5 mg, 0.10 mmol) was added as a solid. The resulting mixture was stirred for 30 minutes and evaporated under reduced pressure. The residue was taken up with 1 mL of anhydrous dichloromethane and precipitated by addition into 15 mL of pentane. The precipitate was decanted and dried under a nitrogen stream. Yield of **5**: 83.6 mg (94%), yellow powder. The crystal used for X-ray diffraction analysis was obtained by liquid-phase diffusion of pentane into a dichloromethane solution of the complex.

^1H NMR (CD_2Cl_2, 400.13 MHz): δ 0.58-0.73 (m, 1 H, PCy_2), 0.75-0.94 (m, 2 H, PCy_2), 1.03-1.81 (m, 14 H, PCy_2), 1.87-2.00 (m, 2 H, PCy_2), 2.03-2.13 (m, 1 H, PCy_2), 2.37 (m, 1 H, PCy_2), 2.61 (m, 1 H, PCy_2), 4.17 (s, 5 H, C_5H_5), 4.23 (m, 1 H, CH of C_5H_3), 4.46 (m, 1 H, CH of C_5H_3), 4.65 (t, J' = 3 Hz,1 H, CH of C_5H_3), 7.32 (m, 3 H, $SbPh_2$), 7.38 (m, 3 H, $SbPh_2$), 7.54 (m, 2 H, $SbPh_2$), 7.65 (m, 2 H, $SbPh_2$). $^{13}C\{^1H\}$ NMR (CD_2Cl_2, 100.61 MHz): δ 27.33 (CH_2^4 of PCy_2), 27.68 (CH_2^4 of PCy_2), 28.09 (d, J_{CP} = 13 Hz, CH_2 of PCy_2), 28.35 (d, J_{CP} = 14 Hz, CH_2 of PCy_2), 28.77 (d, J_{CP} = 12 Hz, CH_2 of PCy_2), 29.03 (d, J_{CP} = 15 Hz, CH_2 of PCy_2), 31.10 (CH_2 of PCy_2), 31.82 (CH_2 of PCy_2), 32.42 (CH_2 of PCy_2),

35.02 (d, J_{CP} = 3 Hz, CH_2 of PCy_2), 38.08 (d, J_{CP} = 36 Hz, CH of PCy_2), 40.65 (d, J_{CP} = 33 Hz, CH of PCy_2), 72.71 (C_5H_5), 75.84 (d, J_{CP} = 6 Hz, CH of C_5H_3), 76.13 (d, J_{CP} = 7 Hz, CH of C_5H_3), 79.74 (d, J_{CP} = 10 Hz, CH of C_5H_3), 130.55 (CH^{para} of $SbPh_2$), 130.63 (CH^{ortho} of $SbPh_2$), 130.73 (CH^{ortho} of $SbPh_2$), 130.79 (CH^{para} of $SbPh_2$), 138.35 (CH^{meta} of $SbPh_2$), 138.64 (CH^{meta} of $SbPh_2$), 141.16 (C^{ipso} of $SbPh_2$), 141.47 (C^{ipso} of $SbPh_2$); the signals due to C^{ipso} of C_5H_3 were not detected. $^{31}P\{^1H\}$ NMR (CD_2Cl_2, 161.98 MHz): δ 42.7 (s). ESI+ MS: *m*/*z* 889.89, 888.14 (M^+), 853.50, 855.34 ($[M - Cl]^+$). Anal. Calc. for $C_{34}H_{40}AuClFePSb{\cdot}CH_2Cl_2$ (974.61): C 43.13, H 4.34%. Found: C 43.52, H 4.28%.

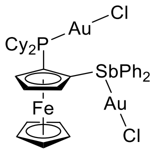

Preparation of 6. A flask equipped with a magnetic stirring bar was charged with **5** (89.0 mg, 0.10 mmol). The starting material was dissolved in anhydrous dichloromethane (5 mL) and chloro(dimethylsulfide)gold(I) (29.5 mg, 0.10 mmol) was added. The reaction mixture was stirred for 30 min and evaporated under reduced pressure, leaving analytically pure **6**. Yield of **6**: 99.9 mg (89%), orange powder. The crystal used for X-ray diffraction analysis was grown by liquid-phase diffusion of pentane into a solution of the compound in dichloromethane.

^1H NMR (CD_2Cl_2, 600.17 MHz): δ 0.83-1.07 (m, 3 H, PCy_2), 1.16-1.89 (m, 13 H, PCy_2), 1.99 (m, 2 H, PCy_2), 2.26 (m, 2 H, PCy_2), 2.47 (m, 1 H, PCy_2), 2.62 (m, 1 H, PCy_2), 4.28 (s, 5 H, C_5H_5), 4.43 (m, 1 H, CH of C_5H_3), 4.58 (m, 1 H, CH of C_5H_3), 4.83 (t, J' = 3 Hz, 1 H, CH of C_5H_3), 7.43-7.51 (m, 3 H, $SbPh_2$), 7.52-7.59 (m, 3 H, $SbPh_2$), 7.63 (m, 2 H, $SbPh_2$), 7.81 (m, 2 H, $SbPh_2$). $^{13}C\{^1H\}$ NMR (CD_2Cl_2, 150.93 MHz): δ 25.85 (CH_2^4 of PCy_2), 26.15 (CH_2^4 of PCy_2), 26.60 (d, J_{CP} = 14 Hz, CH_2 of PCy_2), 26.98 (d, J_{CP} = 13 Hz, CH_2 of PCy_2), 27.34 (m, CH_2 of PCy_2), 27.77 (d, J_{CP} = 15 Hz, CH_2 of PCy_2), 29.46 (CH_2 of PCy_2), 30.36 (CH_2 of PCy_2), 30.61 (CH_2 of PCy_2), 33.65 (d, J_{CP} = 4 Hz, CH_2 of PCy_2), 39.11 (d, J_{CP} = 11 Hz, CH of PCy_2), 39.33 (d, J_{CP} = 10 Hz, CH of PCy_2), 70.29 (d, J_{CP} = 20 Hz, C^{ipso}-Sb of C_5H_3), 72.01 (C_5H_5), 75.36 (d, J_{CP} = 5 Hz, CH of C_5H_3), 76.06 (d, J_{CP} = 5 Hz, CH of C_5H_3), 77.31 (d, J_{CP} = 9 Hz, CH of C_5H_3), 77.75 (d, J_{CP} = 58 Hz, C^{ipso}-P of of C_5H_3), 129.96 (CH^{ortho} of $SbPh_2$), 130.22 (CH^{ortho} of $SbPh_2$), 131.18 (C^{ipso} of $SbPh_2$), 131.44 (CH^{para} of $SbPh_2$), 131.62 (C^{ipso} of $SbPh_2$), 131.80 (CH^{para} of $SbPh_2$) 136.19 (CH^{meta} of $SbPh_2$), 136.41 (CH^{meta} of $SbPh_2$). $^{31}P\{^1H\}$ NMR (CD_2Cl_2, 242.96 MHz): δ 38.6 (s). ESI+ MS: *m*/*z* 1086.9, 1085.3 ([M – Cl]). Anal. Calc. for $C_{34}H_{40}Au_2Cl_2FePSb$ (1122.1): C 32.28, H 3.37%. Found: C 32.07, H 2,97%.

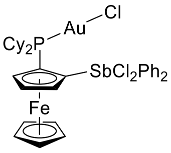

Preparation of 7. Complex **5** (89.0 mg, 0.10 mmol) was dissolved in anhydrous dichloromethane (5 mL) in a flask equipped with a magnetic stirring bar and the solution was cooled to 0 °C. Thionyl chloride (7.3 µL, 0.1 mmol) was introduced and the reaction mixture was allowed to warm to room temperature while stirring for 1 h. The resulting mixture was evaporated under reduced pressure and the residue was taken up with 1 mL of anhydrous dichloromethane and precipitated by addition into 15 mL of pentane. The precipitate was decanted and dried by a stream of nitrogen. Yield of **7**: 84.5 mg (88%), orange powder. The crystal used for X-ray diffraction analysis was grown by liquid-phase diffusion of pentane into a solution of the compound in dichloromethane.

^{1}H NMR ($CDCl_3$, 400.13 MHz): δ 0.37 (m, 1 H, PCy_2), 0.79-0.96 (m, 1 H, PCy_2), 1.03 (m, 1 H, PCy_2), 1.16-1.62 (m, 11 H, PCy_2), 1.62 (m, 1 H, PCy_2), 1.78-2.07 (m, 5 H, PCy_2), 2.13-2.26 (m, 2 H, PCy_2), 4.57 (s, 5 H, C_5H_5), 4.76 (m, 1 H, CH of C_5H_3), 4.80 (m, 1 H, CH of C_5H_3), 4.95 (m, 1 H, CH of C_5H_3), 7.59 (m, 6 H, $SbPh_2$), 8.49 (m, 4 H, $SbPh_2$). ^{13}C{^{1}H} NMR ($CDCl_3$, 100.61 MHz): δ 25.31 ($CH_2{}^4$ of PCy_2), 25.47-26.06 (m, 3 CH_2 of PCy_2), 26.37-26.88 (m, 2 CH_2 of PCy_2), 28.07 (CH_2 of PCy_2), 31.22 (CH_2 of PCy_2), 32.43 (d, J_{CP} = 3 Hz, CH_2 of PCy_2), 32.63 (d, J_{CP} = 3 Hz, CH_2 of PCy_2), 35.92 (d, J_{CP} = 34 Hz, CH of PCy_2), 37.56 (d, J_{CP} = 35 Hz, CH of PCy_2), 71.91 (d, J_{CP} = 7 Hz, CH of C_5H_3), 73.58 (C_5H_5), 76.31 (d, J_{CP} = 14 Hz, CH of C_5H_3), 77.19 (CH of C_5H_3, the signal is obscured by the solvent resonance and was identified in the 2D spectra), 102.11 (d, $^1J_{CP}$ = 12 Hz, C^{ipso}-P of C_5H_3), 129.81 (CH^{ortho} of $SbPh_2$), 132.09 (CH^{para} of $SbPh_2$), 134.96 (CH^{meta} of $SbPh_2$), 139.26 (C^{ipso} of $SbPh_2$); the signal due to C^{ipso}-Sb was not observed. ^{31}P{^{1}H} NMR ($CDCl_3$, 161.98 MHz): δ 45.7 (s). ESI+ MS: *m/z* 906.88, 904.78 908.15 ([M – 2Cl + OH]$^+$). Anal. Calc. for $C_{34}H_{40}AuCl_3FePSb{\cdot}1/2CHCl_3$ (1020.3): C 40.61, H 4.00%. Found: C 40.83, H 3.73%.

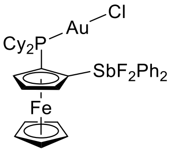

Preparation of 8. A flask equipped with a magnetic stirring bar was charged with **7** (48.0 mg, 0.050 mmol) and the starting material was dissolved in a mixture of dichloromethane (1 mL), acetone (2 mL), and methanol (1 mL). Next, a solution of potassium fluoride in methanol (11.6 mg, 0.20 mmol in 1.5 mL) was added and the resulting mixture was stirred for 90 min and evaporated under reduced pressure. The residue was taken up with 1 mL of anhydrous dichloromethane, filtered through a PTFE filter (0.45 µm porosity), and precipitated by addition

into 15 mL of pentane. The precipitate was decanted and dried under a nitrogen stream. Yield of **8**: 42.7 mg (92%), orange powder.

^{1}H NMR ($CDCl_3$, 400.13 MHz): δ 0.77-2.37 (m, 22 H, PCy_2), 4.31 (s, 5 H, C_5H_5), 4.73 (m, 1 H, CH of C_5H_3), 4.77 (m, 1 H, CH of C_5H_3), 5.00 (m, 1 H, CH of C_5H_3), 7.59 (m, 6 H, CH of $SbPh_2$), 8.28 (m, 4 H, $SbPh_2$). $^{13}C\{^1H\}$ NMR ($CDCl_3$, 100.61 MHz): δ 25.54 (d, J_{CP} = 2 Hz, CH_2^4 of PCy_2), 25.64 (d, J_{CP} = 2 Hz, CH_2^4 of PCy_2), 26.22-26.74 (m, 4 CH_2 of PCy_2), 27.74 (CH_2 of PCy_2), 30.19 (CH_2 of PCy_2), 31.05 (d, J_{CP} = 4 Hz, CH_2 of PCy_2), 32.33 (d, J_{CP} = 4 Hz, CH_2 of PCy_2), 35.47 (d, J_{CP} = 35 Hz, CH of PCy_2), 37.32 (d, J_{CP} = 36 Hz, CH of PCy_2), 72.07 (C_5H_5), 73.22 (d, J_{CP} = 57 Hz, C^{ipso}-Sb of C_5H_3), 73.28 (d, J_{CP} = 7 Hz, CH of C_5H_3), 78.70 (d, J_{CP} = 6 Hz, CH of C_5H_3), 129.86 (CH^{ortho} of $SbPh_2$), 132.54 (CH^{para} of $SbPh_2$), 134.54 (t, $^2J_{CF}$ = 15 Hz, C^{ipso} of $SbPh_2$), 135.80 (t, $^5J_{CF}$ = 5 Hz, CH^{meta} of $SbPh_2$); the signals due to one CH and C^{ipso}-P of C_5H_3 were not observed. ^{19}F NMR ($CDCl_3$, 376.50 MHz): δ –131.6 (s). $^{31}P\{^1H\}$ NMR ($CDCl_3$, 161.98 MHz): δ 46.9 (s). ESI+ MS: *m/z* 967.13, 964.96 ([M + K]$^+$). Anal. Calc. for $C_{34}H_{40}AuF_2ClFePSb$ (926.1): C 44.02, H 4.35%. Found: C 44.03, H 3.84%.

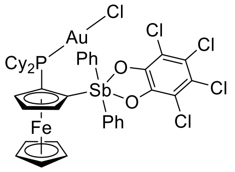

Preparation of 9. A flask equipped with a magnetic stirring bar was charged with **5** (89.0 mg, 0.10 mmol). The starting material was dissolved in anhydrous dichloromethane (5 mL) and *o*-chloranil (24.6 mg, 0.10 mmol) was added. The resulting mixture stirred for 1 h and was evaporated under reduced pressure. The solid residue was dissolved in 1 mL of anhydrous dichloromethane and precipitated by addition into 15 mL of pentane. The precipitate was decanted and dried by a stream of nitrogen. Yield of **9**: 98.8 mg (87%), brown powder. The crystal used for X-ray diffraction analysis was obtained by liquid-phase diffusion of pentane into a dichloromethane solution of the compound.

^{1}H NMR ($CDCl_3$, 400.13 MHz): δ 0.50-2.49 (m, 21 H, PCy_2), 2.70 (m, 1 H, PCy_2), 4.33 (m, 1 H, CH of C_5H_3), 4.53 (s, 5 H, C_5H_5), 4.54 (m, 1 H, CH of C_5H_3), 4.69 (m, 1 H, CH of C_5H_3), 7.37-7.64 (m, 8 H, $SbPh_2$), 7.86 (m, 2 H, $SbPh_2$). $^{13}C\{^1H\}$ NMR ($CDCl_3$, 100.61 MHz): δ 25.15 (CH_2^4 of PCy_2), 25.79 (CH_2^4 of PCy_2), 25.96 (d, J_{CP} = 13 Hz, CH_2 of PCy_2), 26.21 (d, J_{CP} = 13 Hz, CH_2 of PCy_2), 26.80 (d, J_{CP} = 11 Hz, CH_2 of PCy_2), 27.38 (d, J_{CP} = 16 Hz, CH_2 of PCy_2), 27.65 (d, J_{CP} = 5 Hz, CH_2 of PCy_2), 29.90 (CH_2 of PCy_2), 30.81 (d, J_{CP} = 3 Hz, CH_2 of PCy_2), 32.83 (d, J_{CP} = 4 Hz, CH_2 of PCy_2), 36.69 (d, J_{CP} = 36 Hz, CH of PCy_2), 40.90 (d, J_{CP} = 34 Hz, CH of PCy_2), 71.91 (C_5H_5), 73.25 (d, J_{CP} = 6 Hz, CH of C_5H_3), 74.77 (d, J_{CP} = 6 Hz, CH of C_5H_3), 75.06 (d, J_{CP} = 12 Hz, CH of C_5H_3), 76.79 (d, C^{ipso} of C_5H_3, the second component of the doublet was observable in CD_2Cl_2, J_{CP} = 63 Hz), 94.05 (d, J_{CP} = 26 Hz, C^{ipso}-P of C_5H_3), 116.92 (CCl of *o*-chloranil), 120.58 (CCl of *o*-chloranil), 129.27 (CH^{ortho} of $SbPh_2$),

129.69 (CH^{ortho} of $SbPh_2$), 131.64 (CH^{para} of $SbPh_2$), 131.83 (CH^{para} of $SbPh_2$), 132.97 (CH^{meta} of $SbPh_2$), 134.76 (CCl of *o*-chloranil), 135.68 (CH^{meta} of $SbPh_2$), 141.86 (CO of *o*-chloranil), 144.26 (CO of *o*-chloranil); the signals due to C^{ipso} of $SbPh_2$ and one CCl resonance of *o*-chloranil were not observed, possibly due to overlaps. $^{31}P\{^1H\}$ NMR ($CDCl_3$, 161.98 MHz): δ 44.1 (s). ESI+ MS: *m*/*z* 966.92, 969.03 ([M – Cl – $C_6Cl_4O_2$ + 3MeOH + H_2O]$^+$). Anal. Calc. for $C_{40}H_{40}AuCl_5FeO_2PSb\cdot1/2C_5H_{12}$ (1171.6): C 43.57, H 3.96%. Found: C 43.74, H 3.90%.

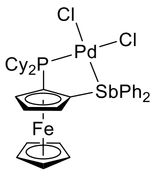

Preparation of 12. Solid dichloro(1,5-cyclooctadiene)palladium(II) (28.6 mg, 0.10 mmol) was added to a solution of ligand **1** (10 mL of 0.10 M solution in dry dichloromethane, 1.0 mmol) in a flask equipped with a stirring bar and the reaction mixture was stirred for 30 min and, subsequently, evaporated under reduced pressure. The residue was dissolved in 1 mL of anhydrous dichloromethane and precipitated by addition into 15 mL of pentane. The precipitate was decanted and dried by a stream of nitrogen. Yield of **12**: 83.5 mg (96%), brown powder. The crystal used for structure determination was grown by liquid-phase diffusion of pentane into a solution of the complex in dichloromethane.

^1H NMR ($CDCl_3$, 400.13 MHz): δ 0.95-1.22 (m, 5 H, PCy_2), 1.31-1.70 (m, 7 H, PCy_2), 1.85 (m, 1 H, PCy_2), 1.91-2.25 (m, 7 H, PCy_2), 2.73-2.89 (m, 2 H, PCy_2), 3.98 (s, 5 H, C_5H_5), 4.48 (d, J' = 2 Hz, 1 H, CH of C_5H_3), 4.77 (d, J' = 2 Hz, 1 H, CH of C_5H_3), 4.95 (vt, J' = 2 Hz, 1 H, CH of C_5H_3), 7.37-7.48 (m, 3 H, $SbPh_2$), 7.50-7.55 (m, 3 H, $SbPh_2$), 7.72-7.81 (m, 2 H, $SbPh_2$), 7.95-8.05 (m, 2 H, $SbPh_2$). $^{13}C\{^1H\}$ NMR ($CDCl_3$, 100.61 MHz): δ 25.42 (CH_2^4 of PCy_2), 26.29 (d, $^4J_{CP}$ = 2 Hz, CH_2^4 of PCy_2), 26.48 (d, J_{CP} = 3 Hz, CH_2 of PCy_2), 26.61 (d, J_{CP} = 5 Hz, CH_2 of PCy_2), 27.12 (d, J_{CP} = 15 Hz, CH_2 of PCy_2), 27.36 (d, J_{CP} = 11 Hz, CH_2 of PCy_2), 29.49-29.71 (m, 3 CH_2 of PCy_2), 29.90 (d, J_{CP} = 2 Hz, CH_2 of PCy_2), 36.41 (d, J_{CP} = 29 Hz, CH of PCy_2), 39.54 (d, J_{CP} = 28 Hz, CH of PCy_2), 70.70 (C_5H_5), 71.83 (d, J_{CP} = 15 Hz, CH of C_5H_3), 73.70 (d, J_{CP} = 4 Hz, CH of C_5H_3), 78.89 (d, J_{CP} = 42 Hz, C^{ipso}-Sb of C_5H_3), 80.49 (d, J_{CP} = 4 Hz, CH of C_5H_3), 84.54 (d, J_{CP} = 49 Hz, C^{ipso}-P of C_5H_3), 129.65 (2 CH^{ortho} of $SbPh_2$), 130.43 (C^{ipso} of $SbPh_2$), 130.96 (C^{ipso} of $SbPh_2$), 131.13 (CH^{para} of $SbPh_2$), 131.55 (CH^{para} of $SbPh_2$), 134.96 (CH^{meta} of $SbPh_2$), 135.74 (CH^{meta} of $SbPh_2$). $^{31}P\{^1H\}$ NMR ($CDCl_3$, 161.98 MHz): δ 69.3 (s). ESI+ MS: *m*/*z* 799.32, 800.65, 796.94 ([M – Cl]$^+$). Anal. Calc. for $C_{34}H_{40}Cl_2FePPdSb\cdot1/3C_8H_{12}$ (870.6): C 50.58, H 5.09%. Found: C 50.85, H 5.12%.

Gold-catalyzed cyclization of *N*-propargylbenzamide. A small glass vial was charged with the respective gold complex (2.0 μmol), *N*-propargylbenzamide (31.8 mg, 0.20 mmol), and CD_2Cl_2 (0.70 mL). The mixture was transferred into an NMR tube and a stock solution of silver(I)

bis(trifluoromethanesulfonyl)imide ($AgNTf_2$) in acetonitrile was added (10 µL of 0.2 M $AgNTf_2$, 2.0 µmol) into the NMR tube. The sample was shaken and inserted into the NMR spectrometer. ^1H NMR spectra were recorded at 25 °C every 10 min for the next 3 h. The yield was determined by integration of the ^1H NMR signals due to the CH_2 group of the starting material (δ_H 4.25 ppm) and the CH_2 group of the product (δ_H 4.65 ppm).

Analytical data for the **substrate**. ^1H NMR ($CDCl_3$, 600.17 MHz): δ 2.28 (t, *J* = 2.5 Hz, 1 H), 4.25 (dd, *J* = 2.5 Hz, 5.1 Hz, 2 H), 6.35-6.50 (bs, 1 H), 7.40-7.46 (m, 2 H), 7.48-7.53 (m, 1 H), 7.79 (d, *J* = 7.0 Hz, 2 H).

Analytical data for the **product**. ^1H NMR ($CDCl_3$, 600.17 MHz): δ 4.36 (q, *J* = 2.7 Hz, 1 H), 4.65 (t, *J* = 2.9 Hz, 2 H), 4.81 (q, *J* = 3.0 Hz, 1 H) 7.40-7.47 (m, 2 H) 7.47-7.54 (m, 1 H), 7.94-8.01 (m, 2 H). The data match those reported in the literature.[8]

Gold-catalyzed cyclization of *N*-propargylbenzamide with concomitant oxidation of the gold complex. A small glass vial was charged with the respective gold complex (2.0 µmol) and ferrocenium tetrafluoroborate (0.55 mg, 2.0 µmol), *N*-propargylbenzamide (31.8 mg, 0.20 mmol), and CD_2Cl_2 (0.70 mL). The mixture was transferred into an NMR tube and a stock solution of silver(I) bis(trifluoromethanesulfonyl)imide ($AgNTf_2$) in acetonitrile was introduced (10 µL of 0.2 M $AgNTf_2$, 2.0 µmol) into the NMR tube. The sample was shaken, and inserted into the NMR spectrometer. The ^1H NMR spectra were recorded at 25 °C every 10 minutes for the next 3 h. The yield was determined by integration of the ^1H NMR signals due to the CH_2 group of the starting material (δ_H 4.25 ppm) and the CH_2 group of the product (δ_H 4.65 ppm).

X-RAY CRYSTALLOGRAPHY

Crystallographic details and refinement parameters

Full-set diffraction data (±*h*±*k*±*l*, 2θ ≤ 26° or 27.5°) were collected with a Bruker D8 VENTURE Kappa Duo instrument equipped with a Cryostream Cooler using Mo Kα radiation (λ = 0.71073 Å). The structures were solved by direct methods using SHELXT-2018[9] and refined with SHELXL-2017.[10] All nonhydrogen atoms were refined with anisotropic displacement parameters. Hydrogens in the BH_3 groups were identified on the electron density maps and refined freely with U_{iso}(H) = 1.5U_{eq}(B). Hydrogen atoms in the CH_n groups were included in their theoretical positions and refined as riding atoms with U_{iso}(H) set to 1.5U_{eq} (C) (methyl groups) or 1.2U_{eq} (aromatic CH and CH_2 groups) and the standard parameters implemented in SHELXL. Additional, structure-specific details are as follows.

Compound **1**·BH_3 crystallized with four independent molecules in the asymmetric unit and as a racemic twin (space group: $P2_1$). The refined contributions from the two enantiomeric domains were 85:15. Compound **1S** also crystallized as a racemic twin (45:55; space group $Pna2_1$) with two crystallographically independent molecules. Complex **5** crystallized with a slight disorder at the Au atom, which was refined over two sites with a 0.26 Å position difference perpendicular to the P-Cl line and a 51:49 occupancy. Compound **1O** crystallized as the solvate **1O**·0.9$CHCl_3$ with heavily disordered chloroform molecules. The contribution of the solvent to the scattering was numerically eliminated using PLATON SQUEEZE.[11] In total, 107 electrons were removed per the unit cell which corresponds with 2× 57 electrons expected for two chloroform molecules. Compounds **1Se** and **3S** crystallized as solvates **1Se**·1/2C_5H_{12} and **3S**·2.7$CHCl_3$, respectively. Even in this case, the contribution of disordered solvent molecules to the overall scattering was eliminated by PLATON SQUEEZE (**1Se**: 168 electrons were removed per the unit cell, which matches the expected value, 4× 42 electrons for four pentane molecules; **3S**: 157 electrons were eliminated, 3× 58 electrons expected for three molecules of $CHCl_3$). Complex **8** also crystallized with a disordered solvent as the solvate **8**·1.7$CHCl_3$. The scattering arising from the disordered solvent was removed using the same strategy. In total, 83 electrons were removed, which is close to the expected value of 2× 58 representing two chloroform molecules. A similar problem was encountered for **9** which crystallized as a disordered solvate **9**·1.2C_5H_{12}. The contribution of pentane molecules was eliminated as above, resulting in the removal of 806 electrons per unit cell, matching the theoretical value (20× 42 electrons).

Selected crystallographic data and structure refinement parameters are presented in Table S1. All geometric data and structural diagrams were obtained using a recent version of the PLATON program.[12] The numerical values were rounded to one decimal place with respect to the estimated standard deviations.

Table S1. Selected crystallography data and structure refinement parameters.

Compound	**1**·BH_3	**1**	**2**·BH_3
Formula	$C_{34}H_{43}BFePSb$	$C_{34}H_{40}FePSb$	$C_{22}H_{33}BBrFeP$
M	671.06	657.23	475.02
Crystal system	monoclinic	orthorhombic	monoclinic
Space group	$P2_1$ (no. 4)[c]	*Pbca* (no. 61)	$P2_1/c$ (no. 14)
T [K]	120(2)	120(2)	120(2)
a [Å]	16.8252(9)	11.3704(3)	12.9328(3)
b [Å]	21.445(1)	18.3753(5)	10.0858(2)
c [Å]	17.3740(9)	27.8781(7)	17.1093(4)
α [°]	90	90	90
β [°]	100.532(2)	90	106.606(1)
γ [°]	90	90	90
V [$Å^3$]	6163.1(5)	5824.7(3)	2138.62(8)
Z	8	8	4
μ(Mo Kα) [mm^{-1}]	1.421	1.502	2.652
F(000)	2752	2688	984
Diffrns collected	178702	78481	25596
Independent diffrns	28256	6675	4896
Observed[a] diffrns	27458	6414	4641
R_{int}[b] [%]	3.94	3.23	2.36
No. of parameters	1434	334	244
R[b] obsd diffrns [%]	2.36	2.25	2.31
R, w*R*[b] all data [%]	2.48, 8.52	2.37, 5.07	2.47, 5.69
Δρ [e $Å^{-3}$]	0.74, –0.60	0.36, –0.59	0.78, –0.93
CCDC no.	2429386	2429387	2429388

[a] Diffractions with $I > 2\sigma(I)$. [b] Definitions: $R_{int} = \Sigma|F_o^2 - F_o^2(\text{mean})|/\Sigma F_o^2$, where F_o^2 (mean) is the average intensity of symmetry-equivalent diffractions. $R = \Sigma||F_o| - |F_c||/\Sigma|F_o|$, $wR = [\Sigma\{w(F_o^2 - F_c^2)^2\}/\Sigma w(F_o^2)^2]^{1/2}$. [c] The compound crystallized as a racemic twin.

Table S1 continued

Compound	**1O**·0.9$CHCl_3$	**1S**	**1Se**·½C_5H_{12}
Formula	$C_{34.9}H_{40.9}Cl_{2.7}FeOPSb$	$C_{34}H_{40}FePSbS$	$C_{36.5}H_{46}FePSbSe$
M	780.70	689.29	736.19
Crystal system	triclinic	orthorhombic	monoclinic
Space group	*P*–1 (no. 2)	*Pna*2_1 (no. 33)[c]	*C*2/*c* (no. 15)
T [K]	120(2)	120(2)	120(2)
a [Å]	10.2718(7)	19.2731.(7)	41.482(4)
b [Å]	11.9683(7)	8.7899(3)	8.9125(7)
c [Å]	15.1151(9)	35.632(2)	19.180(2)
α [°]	70.791(2)	90	90
β [°]	89.883(2)	90	115.468(3)
γ [°]	71.100(2)	90	90
V [$Å^3$]	1648.7(2)	6036.3(4)	6402(1)
Z	2	8	8
μ(Mo Kα) [mm^{-1}]	1.554	1.520	2.505
F(000)	688	2816	2960
Diffrns collected	46973	101404	167417
Independent diffrns	7576	13838	7350
Observed[a] diffrns	7312	13491	6897
R_{int}[b] [%]	3.99	4.55	5.55
No. of parameters	343	686	343
R[b] obsd diffrns [%]	3.24	2.25	3.73
R, w*R*[b] all data [%]	3.35, 8.57	2.36, 5.11	3.97, 9.54
Δρ [e $Å^{-3}$]	1.49, –1.26	0.68, –0.36	2.12, –1.03
CCDC no.	2429389	2429390	2429391

Table S1 continued

Compound	**3**·BH_3	**3**·C_7H_{16}	**3O**
Formula	$C_{40}H_{43}BCl_4FeO_2PSb$	$C_{47}H_{56}Cl_4FeO_2PSb$	$C_{40}H_{40}Cl_4FeO_3PSb$
M	916.92	1003.28	919.09
Crystal system	monoclinic	monoclinic	monoclinic
Space group	$P2_1/c$ (no. 14)	$P2_1/c$ (no. 14)	$P2_1/c$ (no. 14)
T [K]	120(2)	120(2)	120(2)
a [Å]	19.2155(8)	20.746(1)	17.2193(6)
b [Å]	23.5388(9)	13.5814(9)	11.9529(4)
c [Å]	17.2619(6)	16.678(1)	18.7851(5)
α [°]	90	90	90
β [°]	90.838(1)	110.147(3)	97.679(1)
γ [°]	90	90	90
V [$Å^3$]	7806.9(5)	4411.7(5)	3831.7(2)
Z	8	4	4
μ(Mo Kα) [mm^{-1}]	1.413	1.258	1.442
F(000)	3712	2056	1856
Diffrns collected	118361	62953	87061
Independent diffrns	17887	10123	8785
Observed[a] diffrns	14361	8323	8224
R_{int}[b] [%]	9.71	6.15	3.17
No. of parameters	919	507	451
R[b] obsd diffrns [%]	5.11	4.99	1.82
R, w*R*[b] all data [%]	6.91, 12.6	6.58, 10.94	2.02, 4.48
Δρ [e $Å^{-3}$]	1.69, –0.78	2.20, –0.91	0.40, –0.33
CCDC no.	2429392	2429393	2429394

Table S1 continued

Compound	**3S**·2.7$CHCl_3$	**3Se**	**4**
Formula	$C_{42.7}H_{42.7}Cl_{12.1}FeO_2PSSb$	$C_{40}H_{40}Cl_4FeO_2PSbSe$	$C_{46}H_{40}Cl_8FeO_4PSb$
M	1257.52	1101.42	1148.96
Crystal system	monoclinic	monoclinic	monoclinic
Space group	$P2_1/c$ (no. 14)	$P2_1/c$ (no. 14)	$P2_1/c$ (no. 14)
T [K]	120(2)	120(2)	120(2)
a [Å]	10.4303(4)	10.4515(3)	17.4541(7)
b [Å]	19.1099(8)	19.2509(5)	15.1642(6)
c [Å]	20.6368(7)	20.8725(6)	17.4865(6)
α [°]	90	90	90
β [°]	90.474(1)	92.335(1)	101.606(1)
γ [°]	90	90	90
V [$Å^3$]	4113.2(3)	4196.1(2)	4533.7(3)
Z	4	4	4
μ(Mo Kα) [mm^{-1}]	1.929	2.377	1.467
F(000)	1888	2192	2304
Diffrns collected	59321	108273	71007
Independent diffrns	9431	9620	10375
Observed[a] diffrns	9105	9009	9085
R_{int}[b] [%]	2.41	3.70	5.22
No. of parameters	451	487	550
R[b] obsd diffrns [%]	2.24	1.75	3.50
R, wR[b] all data [%]	2.35, 4.99	1.96, 3.97	4.35, 7.31
Δρ [e $Å^{-3}$]	0.45, –0.74	0.36, –0.28	0.81, –0.49
CCDC no.	2429395	2429396	2429397

Table S1 continued

Compound	**5**	**6**·CH_2Cl_2	**7**·$2CHCl_3$
Formula	$C_{34}H_{40}AuClFePSb$	$C_{35}H_{42}Au_2Cl_4FePSb$	$C_{36}H_{42}AuCl_9FePSb$
M	889.64	1206.99	1199.30
Crystal system	orthorhombic	monoclinic	triclinic
Space group	*Pbca* (no. 61)	*C*2/*c* (no. 15)	*P*–1 (no. 2)
T [K]	120(2)	120(2)	120(2)
a [Å]	10.588(1)	38.909(1)	9.3266(3)
b [Å]	22.030(3)	8.9988(3)	15.5047(6)
c [Å]	27.139(5)	21.9958(7)	15.7495(6)
α [°]	90	90	66.871(1)
β [°]	90	103.797(1)	78.234(1)
γ [°]	90	90	76.797(1)
V [$Å^3$]	6330(2)	7479.3(4)	2022.4(1)
Z	8	8	2
μ(Mo Kα) [mm^{-1}]	6.081	9.269	5.297
F(000)	3456	4560	1164
Diffrns collected	86258	120078	52868
Independent diffrns	7220	8561	9270
Observed[a] diffrns	6316	8244	9084
R_{int}[b] [%]	3.24	4.35	2.49
No. of parameters	362	397	442
R[b] obsd diffrns [%]	3.06	1.46	2.30
R, w*R*[b] all data [%]	3.87, 7.61	1.57, 3.56	2.35, 6.36
Δρ [e $Å^{-3}$]	1.84, –1.96	0.50, –1.05	1.22, –2.05
CCDC no.	2429398	2429399	2429400

Table S1 continued

Compound	**8**·1.7CHCl$_3$	**9**·1.2C$_5$H$_{12}$	**12**
Formula	$C_{36.7}H_{42.7}AuCl_{9.1}F_2FePSb$	$C_{46}H_{54.4}AuCl_5FeO_2PSb$	$C_{34}H_{40}FePSbPdCl_2$
M	1250.00	1222.14	834.53
Crystal system	triclinic	monoclinic	orthorhombic
Space group	*P*–1 (no. 2)	*C*2/*c* (no. 15)	$P2_12_12_1$ (no. 19)[d]
T [K]	120(2)	120(2)	120(2)
a [Å]	9.5736(8)	31.781(1)	9.7905(3)
b [Å]	15.087(1)	15.2223(6)	17.7847(6)
c [Å]	15.183(1)	24.0939(9)	18.3791(5)
α [°]	70.844(3)	90	90
β [°]	74.180(3)	125.434(2)	90
γ [°]	81.677(3)	90	90
V [$Å^3$]	1989.3(3)	9497.2(7)	3200.2(2)
Z	2	8	4
μ(Mo Kα) [mm^{-1}]	5.403	4.293	2.081
F(000)	1016	4416	1664
Diffrns collected	81164	91950	59958
Independent diffrns	9106	10861	7349
Observed[a] diffrns	8986	9868	7327
R_{int}[b] [%]	4.27	4.72	2.68
No. of parameters	406	460	361
R[b] obsd diffrns [%]	2.40	2.17	2.15
R, w*R*[b] all data [%]	2.43, 5.28	2.58, 5.09	2.16, 5.49
Δρ [e $Å^{-3}$]	2.35, –1.79	0.96, –0.64	1.90, –0.78
CCDC no.	2429401	2429402	2429403

[d] Flack's enantiomorph parameter: 0.004(4).

Table S2. Selected distances and angles for **1E** series (in Å and deg).

Parameter[a]	**1**	**1O**	**1S** (mol 1/mol 2)[c]	**1Se**
E	void	O	S	Se
Sb1-C1	2.127(2)	2.142(3)	2.131(4)/2.135(3)	2.143(4)
Sb1-C11	2.160(2)	2.151(3)	2.158(4)/2.161(4)	2.154(5)
Sb1-C17	2.160(2)	2.173(3)	2.171(4)/2.170(4)	2.168(4)
C1-Sb1-C11	95.88(7)	95.7(1)	94.6(1)/94.5(1)	95.1(1)
C1-Sb1-C17	96.00(7)	93.8(1)	93.6(1)/92.7(1)	93.1(1)
C11-Sb1-C17	94.11(7)	97.9(1)	97.5(1)/97.9(2)	97.2(2)
P1-C2	1.827(2)	1.790(2)	1.803(3)/1.801(3)	1.802(3)
P1-C23	1.872(2)	1.824(3)	1.840(4)/1.843(4)	1.845(4)
P1-C29	1.863(2)	1.822(3)	1.848(4)/1.833(4)	1.832(4)
C2-P1-C23	96.51(8)	107.7(1)	99.8(2)/100.6(2)	101.5(2)
C2-P1-C29	102.26(8)	112.0(1)	105.3(2)/104.7(2)	104.5(2)
C23-P1-C29	104.44(8)	109.6(1)	108.9(2)/108.3(2)	108.5(2)
P1-E1	n.a.	1.505(2)	1.956(1)/1.958(1)	2.116(1)
Sb1⋯E1	3.6701(6)[b]	2.828(2)	3.707(1)/3.735(1)	3.8275(7)
Sb1-C1-C2-P1	7.6(2)	10.6(3)	8.4(5)/8.9(5)	12.2(6)
Fe1-C(1-10)	2.032(2)-2.051(2)	2.042(3)-2.065(3)	2.032(4)-2.059(4)/ 2.032(4)-2.056(4)	2.028(4)-2.058(4)
tilt	1.4(1)	4.6(2)	3.7(2)/1.7(2)	1.8(3)

[a] Definitions: tilt is the dihedral angle of the least-squares cyclopentadienyl planes; n.a. = not applicable. [b] Sb1⋯P1 distance. [c] Compound **1S** crystallized with two independent molecules in the unit cell.

Table S3. Selected distances and angles for the four crystallographically independent molecules in the structure of **1**·BH_3 (in Å and deg).[a]

Parameter[a]	molecule 1	molecule 2	molecule 3	molecule 4
Sb1-C1	2.133(3)	2.136(3)	2.140(3)	2.147(3)
Sb1-C11	2.144(4)	2.147(4)	2.151(5)	2.161(4)
Sb1-C17	2.149(4)	2.153(4)	2.145(4)	2.159(4)
C1-Sb1-C11	94.3(2)	95.5(1)	93.5(2)	92.7(1)
C1-Sb1-C17	93.7(2)	93.5(1)	94.5(1)	95.6(1)
C11-Sb1-C17	100.2(2)	99.2(1)	98.9(2)	99.8(1)
P1-C2	1.803(4)	1.803(3)	1.800(4)	1.798(4)
P1-C23	1.843(4)	1.841(4)	1.848(4)	1.828(4)
P1-C29	1.829(4)	1.831(4)	1.832(4)	1.845(4)
C2-P1-C23	104.7(2)	104.8(2)	104.7(2)	109.4(2)
C2-P1-C29	110.7(2)	109.7(2)	110.3(2)	104.2(2)
C23-P1-C29	103.9(2)	104.8(2)	103.7(2)	105.0(2)
P1-B1	1.937(5)	1.936(5)	1.926(5)	1.925(5)
Sb1-C1-C2-P1	2.4(6)	4.4(6)	−3.8(6)	−6.1(5)
Fe1-C(1-10)	2.030(4)-2.063(4)	2.024(4)-2.063(4)	2.022(4)-2.057(4)	2.023(4)-2.057(4)
tilt	6.1(2)	6.0(2)	7.1(2)	5.6(2)

[a] Definitions: tilt is the dihedral angle of the least-squares cyclopentadienyl planes. The atomic labeling of the four molecules is strictly analogous.

Table S4. Selected distances and angles for type **3** compounds (in Å and deg).

Parameter[a]	**3**·BH_3[b]	**3**	**3O**	**3S**	**3Se**
E	B1 (mol 1/mol 2)	void	O3	S1	Se1
Sb1-C1	2.125(4)/2.118(4)	2.137(5)	2.118(1)	2.121(2)	2.123(1)
Sb1-C11	2.145(4)/2.142(4)	2.137(4)	2.136(2)	2.138(2)	2.141(2)
Sb1-C17	2.108(5)/2.109(5)	2.117(5)	2.121(1)	2.128(2)	2.137(1)
C1-Sb1-C11	100.3(2)/96.4(2)	97.6(2)	98.28(6)	99.02(7)	98.75(6)
C1-Sb1-C17	101.2(2)/102.1(2)	106.9(2)	100.11(5)	102.00(6)	101.55(5)
C11-Sb1-C17	101.4(2)/104.8(2)	98.1(2)	101.26(6)	100.44(6)	99.31(6)
Sb1-O1	2.054(3)/2.057(3)	2.053(3)	2.058(1)	2.081(1)	2.094(1)
Sb1-O2	2.070(3)/2.069(3)	2.113(3)	2.077(1)	2.084(1)	2.089(1)
O1-Sb1-O2	78.6(1)/77.9(1)	78.3(1)	78.72(4)	77.73(5)	77.54(4)
P1-C2	1.806(4)/1.800(4)	1.811(5)	1.789(2)	1.795(2)	1.794(1)
P1-C23	1.847(4)/1.841(4)	1.850(5)	1.812(2)	1.834(2)	1.836(1)
P1-C29	1.836(4)/1.842(4)	1.844(4)	1.804(2)	1.826(2)	1.826(2)
C2-P1-C23	101.7(2)/104.4(2)	109.1(2)	107.36(7)	104.38(8)	105.03(7)
C2-P1-C29	108.3(2)/107.8(2)	108.8(2)	110.13(7)	109.45(8)	109.48(7)
C23-P1-C29	110.9(2)/108.3(2)	107.3(2)	112.67(7)	111.99(8)	112.09(7)
P1-E	1.922(5)/1.912(5)	n.a.	1.529(1)	1.9946(6)	2.1475(5)
Sb1···E	n.a.	2.859(1)[c]	2.225(2)	2.8085(5)	2.8960(5)
Sb1-C1-C2-P1	1.8(6)/−5.9(6)	0.2(4)	2.6(2)	−4.9(2)	−2.9(2)
C1-C2-P-E	−6.1(4)/−7.3(4)	n.a.	−8.1(1)	−7.4(2)	−8.1(1)
Fe1-C(1-10)	2.032(5)-2.058(4)/ 2.016(4)-2.054(4)	2.035(4)- 2.067(5)	2.044(2)- 2.061(2)	2.042(2)- 2.059(2)	2.040(2)- 2.059(2)
tilt	5.4(3)/2.3(2)	5.5(3)	5.08(9)	5.2(1)	6.23(9)
anil vs. C_5H_3	68.3(2)/71.4(2)	70.5(2)	68.13(8)	55.57(9)	58.06(8)

[a] tilt is the dihedral angle of the least-squares cyclopentadienyl planes and anil *vs.* C_5H_3 is the dihedral angle of the C(1-5) and C(35-40) planes; n.a. = not applicable. [b] Values for molecule 1/molecule 2. [c] Sb1···P1 distance.

Table S5. Selected distances and angles for compound **4** (in Å and deg).[a]

Distances		Angles	
Sb1-C1	2.152(2)	C1-Sb1-C11	100.8(1)
Sb1-C11	2.135(3)	C1-Sb1-C17	97.7(1)
Sb1-C17	2.149(3)	C11-Sb1-C17	95.6(1)
Sb1-O1	2.069(2)	O1-Sb1-O2	79.38(7)
Sb1-O2	2.071(2)	C1-Sb1-O3	87.97(9)
Sb-O3	2.164(2)	C11-Sb1-O3	84.12(9)
P1-O4	1.586(2)	O1-Sb1-O3	87.44(8)
P1···Sb1	4.0514(8)	O2-Sb1-O3	82.21(7)
P1-C2	1.764(3)	C2-P1-C23	107.1(1)
P1-C23	1.819(3)	C2-P1-C27	111.1(1)
P1-C29	1.813(3)	C23-P1-C27	110.0(1)
Fe1-C(1-10)	2.040(3)-2.058(3)	tilt	4.8(2)

[a] tilt is the dihedral angle of the least-square cyclopentadienyl planes.

Table S6. Selected distances and angles for Au(I) complexes **5**, **6**, **7**, **8**, and **9** (in Å and deg).

Parameter[a]	**5**[b]	**6**[c]	**7**	**8**	**9**
X1/X2	n.a.	n.a.	Cl2/Cl3	F1/F2	O1/O2
Sb1-C1	2.146(4)	2.096(2)	2.107(3)	2.087(3)	2.121(3)
Sb1-C11	2.158(4)	2.113(3)	2.135(3)	2.106(3)	2.125(3)
Sb1-C17	2.160(5)	2.113(2)	2.101(3)	2.102(3)	2.107(3)
C1-Sb1-C11	94.3(2)	103.55(9)	121.6(1)	126.8(1)	98.1(1)
C1-Sb1-C17	93.8(2)	104.02(9)	127.6(1)	109.7(1)	102.1(1)
C11-Sb1-C17	97.6(2)	98.46(9)	110.6(1)	123.4(1)	103.5(1)
Sb1-X1	n.a.	n.a.	2.472(1)	1.984(2)	2.042(2)
Sb1-X2	n.a.	n.a.	2.4582(8)	1.974(2)	2.061(2)
X1-Sb1-X2	n.a.	n.a.	178.55(3)	177.90(8)	78.61(9)
P1-C2	1.799(4)	1.814(2)	1.808(3)	1.803(3)	1.802(3)
P1-C23	1.867(6)	1.839(2)	1.844(3)	1.852(3)	1.837(3)
P1-C29	1.842(5)	1.841(2)	1.836(3)	1.838(3)	1.826(3)
C2-P1-C23	103.3(2)	101.71(9)	100.8(1)	100.8(1)	103.3(2)
C2-P1-C29	105.4(2)	108.2(1)	104.2(2)	104.3(1)	107.0(1)
C23-P1-C29	115.0(2)	110.7(1)	109.4(1)	109.2(1)	110.5(1)
P1-Au1	2.305(2)/2.167(4)	2.2425(6)	2.2337(9)	2.2319(8)	2.2360(8)
Au1-Cl1	2.250(2)/2.407(4)	2.3012(6)	2.297(1)	2.2994(9)	2.2768(8)
P1-Au1-Cl1	175.3(1)/168.6(3)	173.10(3)	174.02(3)	175.72(3)	173.15(4)
Sb1···Au1	3.387(2)/3.546(4)	3.2833(6)	4.3743(4)	4.2255(6)	3.3632(7)
Sb1-C1-C2-P1	1.9(5)	15.8(3)	6.2(5)	–3.9(4)	5.8(4)
Fe1-C(1-10)	2.032(4)-2.057(4)	2.048(2)-<br>2.059(3)	2.028(3)-<br>2.064(4)	2.029(3)-<br>2.060(3)	2.036(2)-<br>2.064(4)
tilt	2.3(3)	4.6(2)	4.7(2)	1.8(2)	6.8(2)

[a] tilt is the dihedral angle of the least-square cyclopentadienyl planes; n.a. = not applicable. [b] The gold atom Au1 was refined over two positions due to disorder. Parameters pertaining to both positions are given. [c] Additional data: Au1···Au2 = 3.0602(8), Sb1-Au2 = 2.4845(9), Au2-Cl2 = 2.274(1), Sb1-Au2-Cl2 = 176.39(3).

Table S7. Selected distances and angles for Pd(II) complex **12** (in Å and deg).

Distances		Angles	
Pd1-Sb1	2.4595(5)	Sb1-Pd1-P1	89.86(3)
Pd1-P1	2.243(1)	Sb1-Pd1-Cl1	83.14(4)
Pd1-Cl1	2.363(1)	P1-Sb1-Cl2	89.86(4)
Pd1-Cl2	2.371(1)	Cl1-Pd1-Cl2	97.12(5)
Sb1-C1	2.085(4)	C1-Sb1-C11	104.0(2)
Sb1-C11	2.113(4)	C1-Sb1-C17	108.1(1)
Sb1-C17	2.111(4)	C11-Sb1-C17	104.2(2)
P1-C2	1.822(4)	C2-P1-C23	105.2(2)
P1-C23	1.837(4)	C2-P1-C29	106.8(2)
P1-C29	1.853(4)	C23-P1-C29	109.9(2)
Fe1-C(1-10)	2.038(4)-2.061(4)	tilt[a]	6.3(3)

[a] tilt is the dihedral angle of the least-square cyclopentadienyl planes.

Structure diagrams

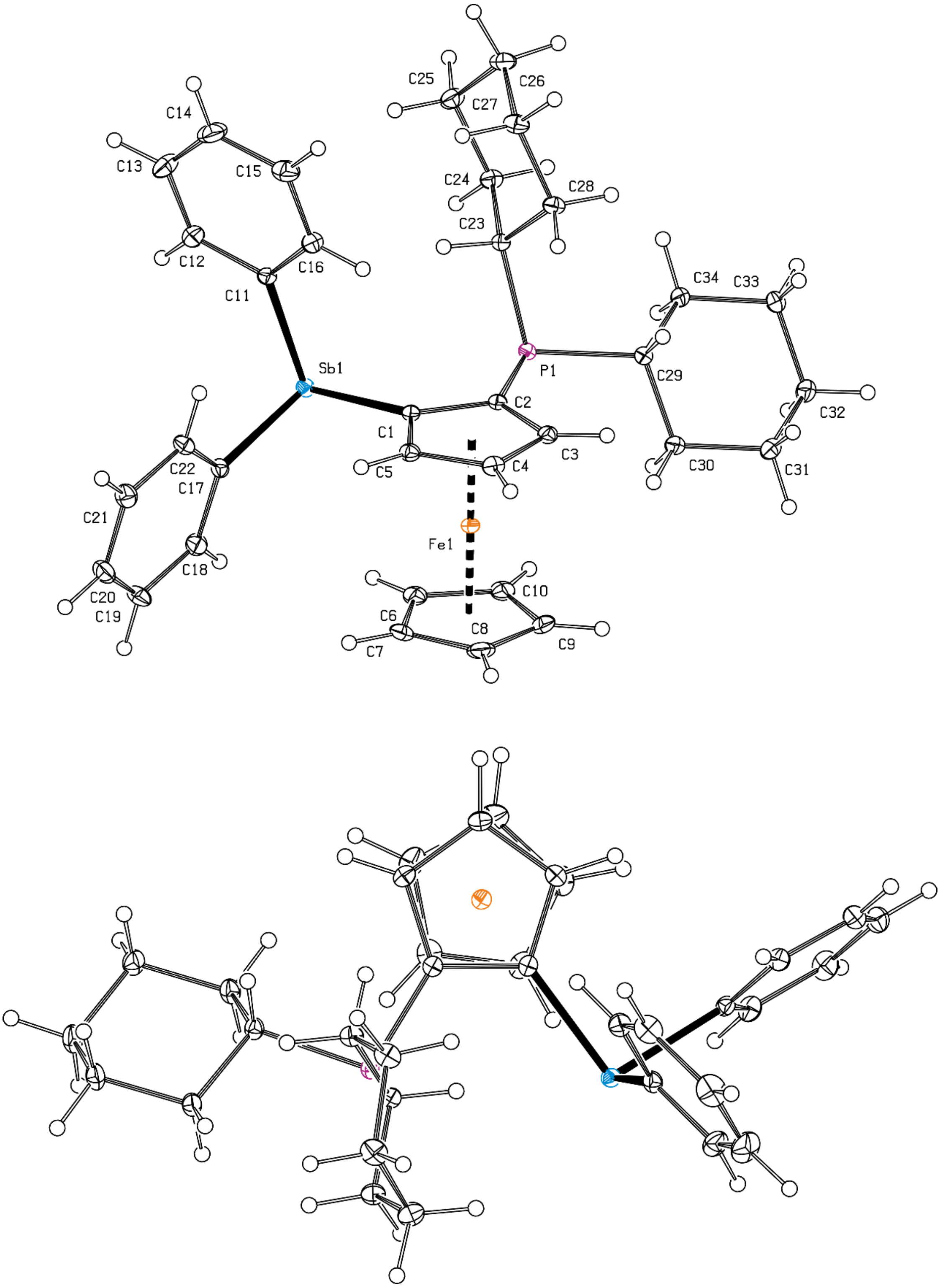

Figure S1. PLATON plot of **1** showing 30% probability ellipsoids; a general (top) and a top view (bottom) are shown to illustrate orientation of the two functional groups at the ferrocene unit.

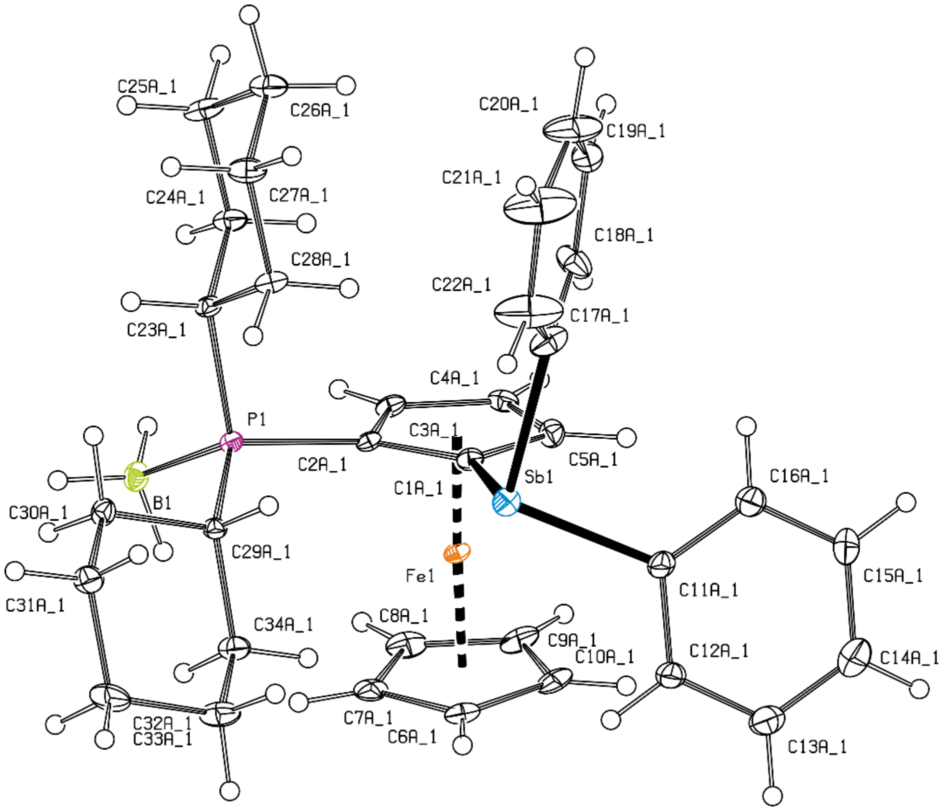

Figure S2. PLATON plot of molecule 1 in the of **1**·BH_3 showing 30% probability ellipsoids.

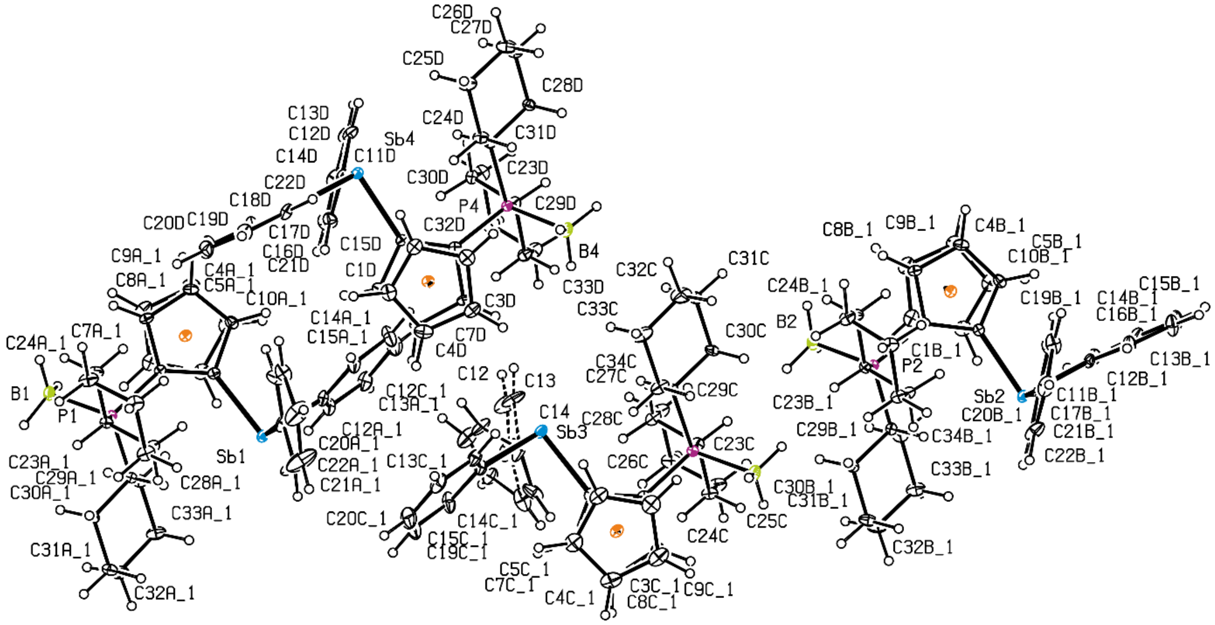

Figure S3. Complete structure diagram for **1**·BH_3 with 30% probability ellipsoids.

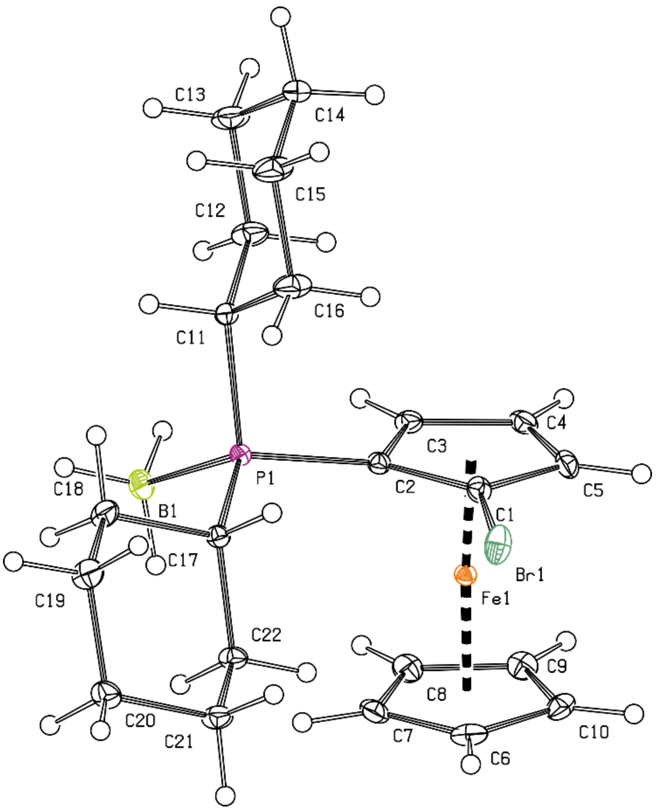

Figure S4. PLATON plot of **2**·BH_3 showing 30% probability ellipsoids.

Compound **2**·BH_3 was racemic and crystallized with both enantiomeric molecules in the centric monoclinic cell (space group $P2_1/c$). The ferrocene unit in its molecule adopted its regular geometry with the Fe1-C(1-10) distances in the range 2.015(2)-2.054(2) Å and tilt angle of 3.2(1)°. The C1-Br1 and C2-P1 were 1.888(2) Å and 1.800(2) Å, respectively. The geometry around the phosphorus atom was approximately tetrahedral (C-P-C angles: 103.84(7)-110.90(7)°, B-P-C angles: 111.01(8)-114.04(8)°); the P1-B1 and P1-C11/C17 distances were 1.923(2) Å and 1.847(2)/1.834(2) Å, respectively. The torsion angle Br1-C1-C2-P1 was 8.4(2)°, indicating only minor distortion of the disubstituted cyclopentadienyl ring.

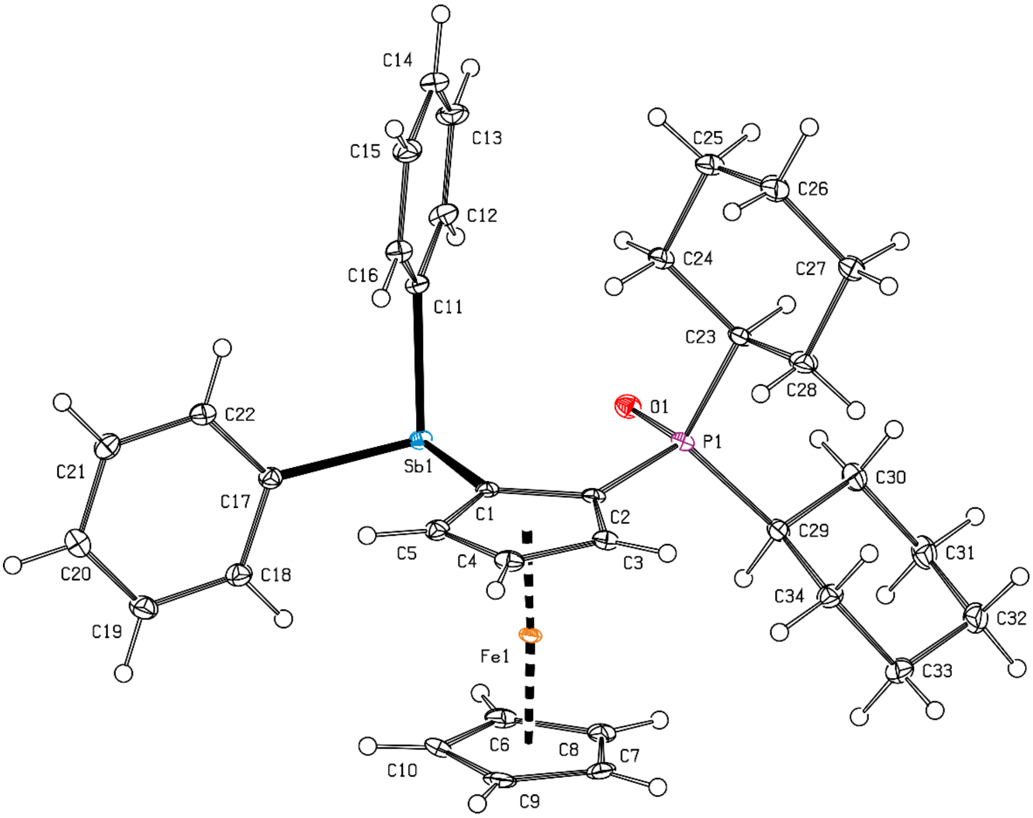

Figure S5. PLATON plot of **1O**·0.9$CHCl_3$ showing 30% probability ellipsoids. The solvent was eliminated using PLATON SQUEEZE.

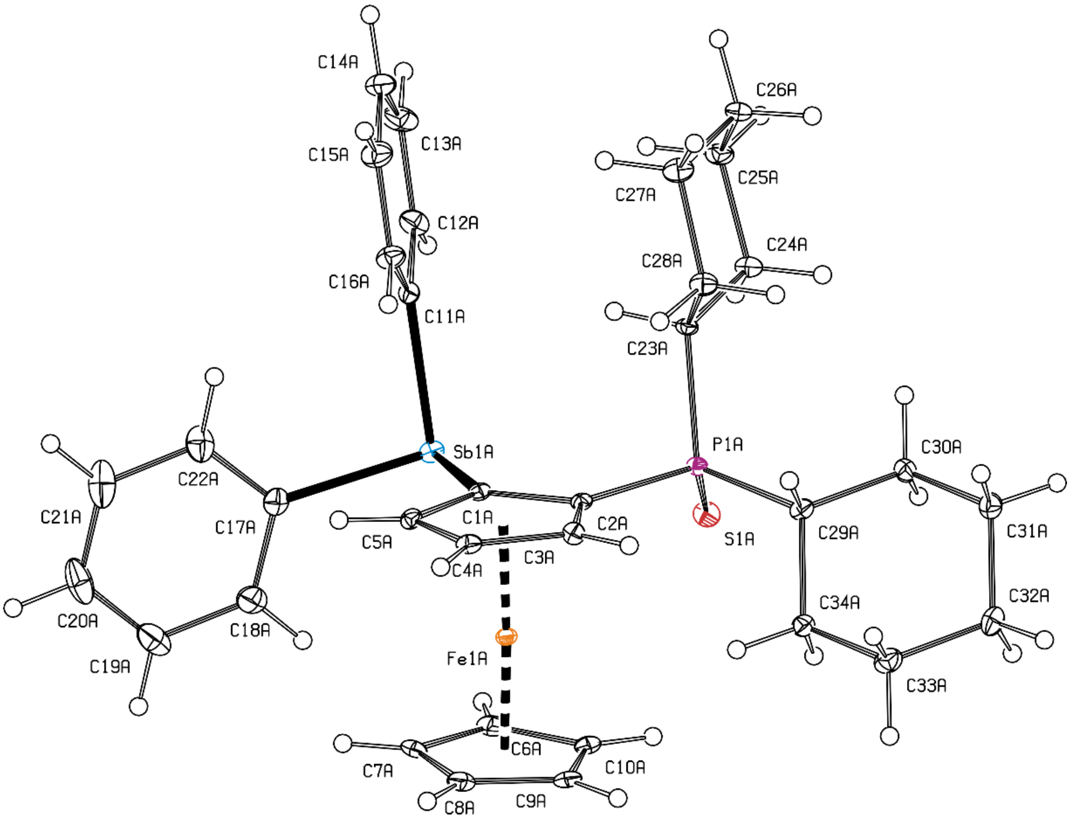

Figure S6. PLATON plot of molecule 1 in the structure of **1S** showing 30% probability ellipsoids.

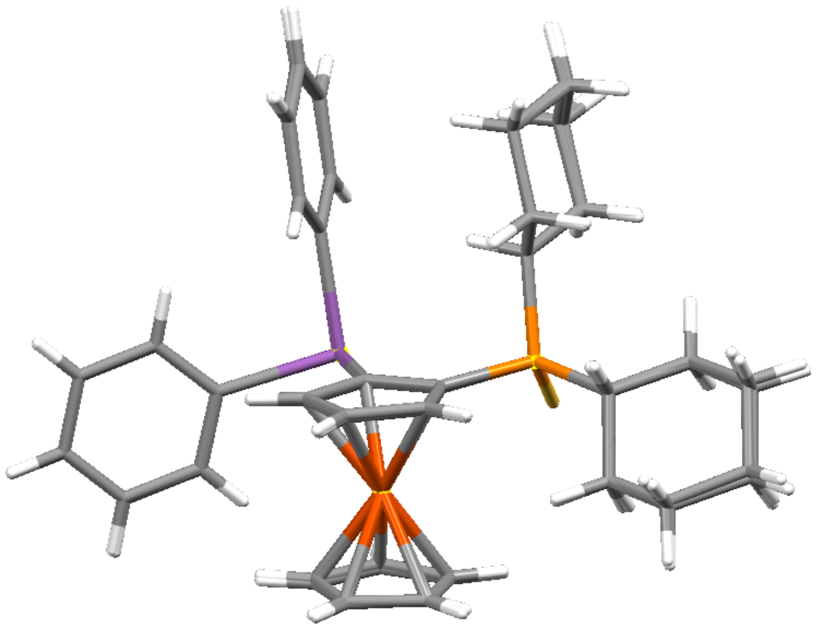

Figure S7. Overlap of the crystallographically independent molecules in the structure of **1S.**

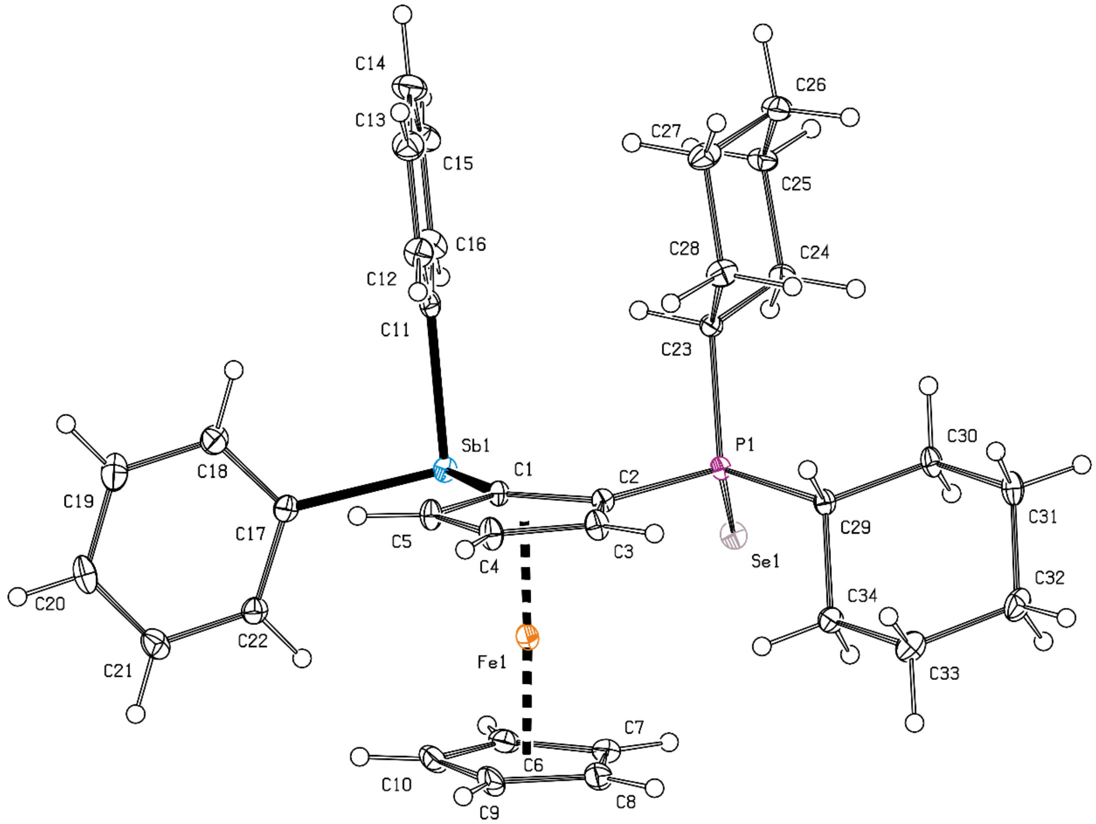

Figure S8. PLATON plot of **1Se**·½C_5H_{12} showing 30% probability ellipsoids. The solvent was eliminated by PLATON SQUEEZE.

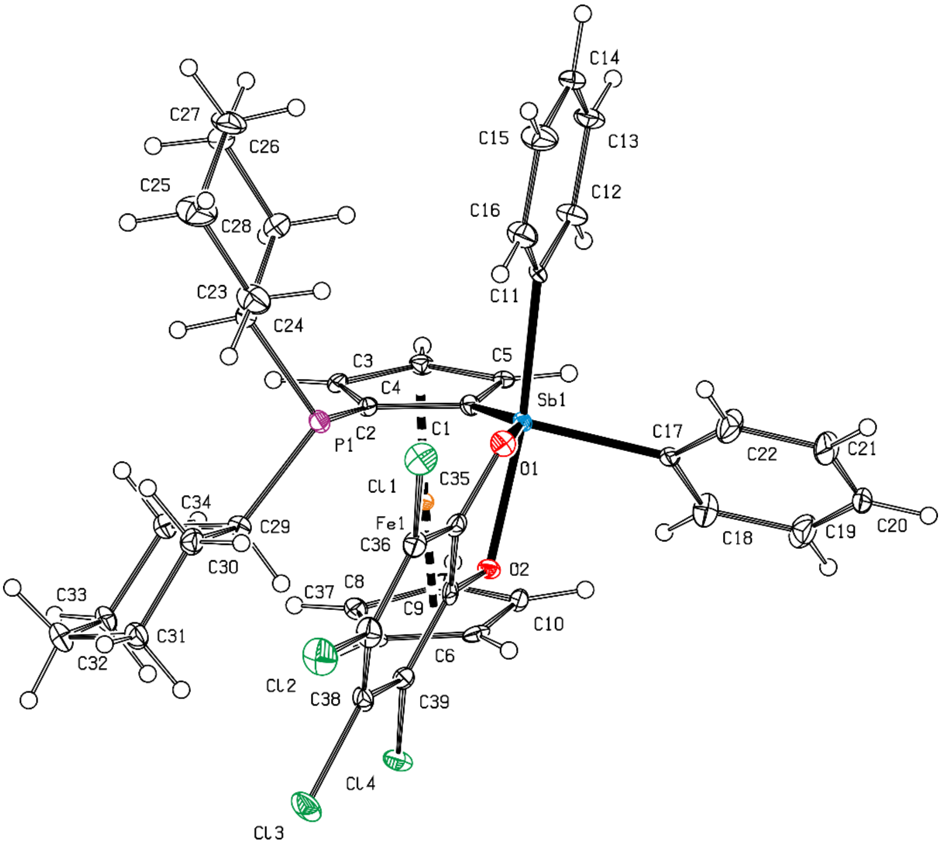

Figure S9. PLATON plot of the molecular structure of **3**·C_7H_{16} showing 30% probability ellipsoids. The solvent molecule was omitted for clarity.

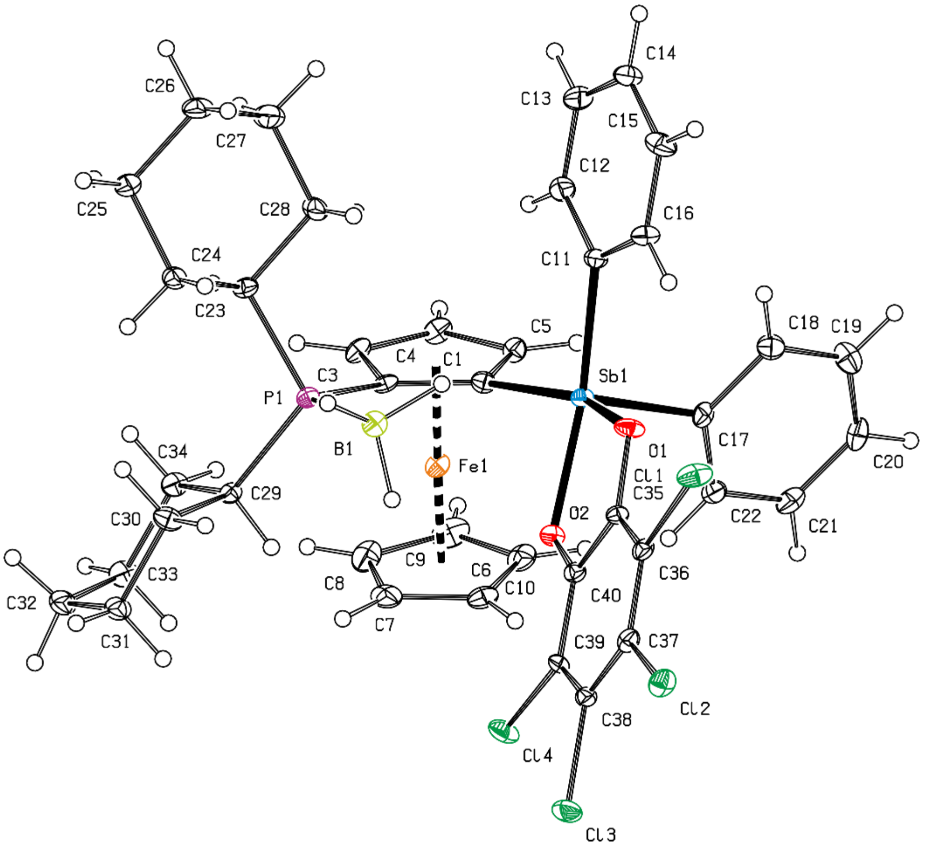

Figure S10. PLATON plot of molecule 1 in the structure of **3**·BH_3 (30% probability ellipsoids).

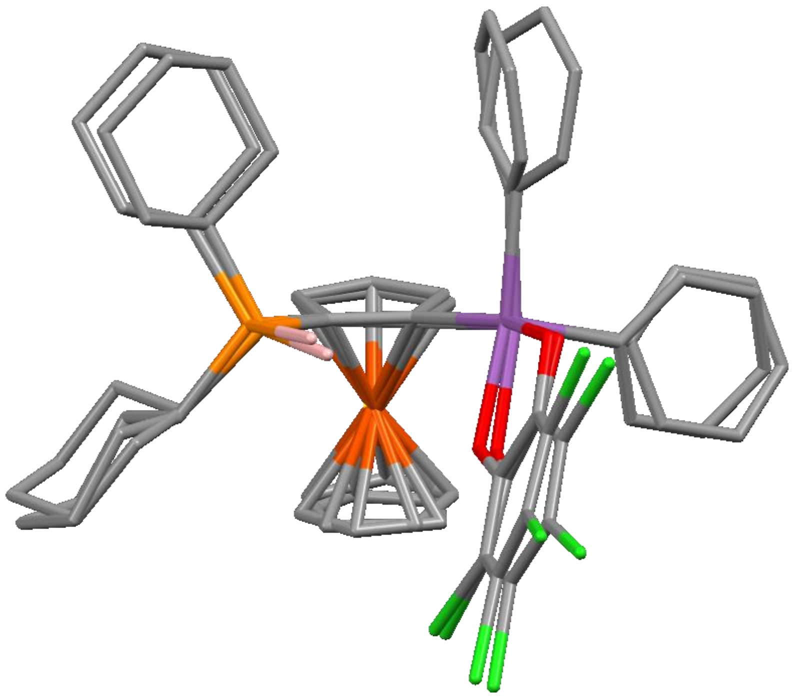

Figure S11. Overlap of the crystallographically independent molecules in the structure of **3**·BH_3.

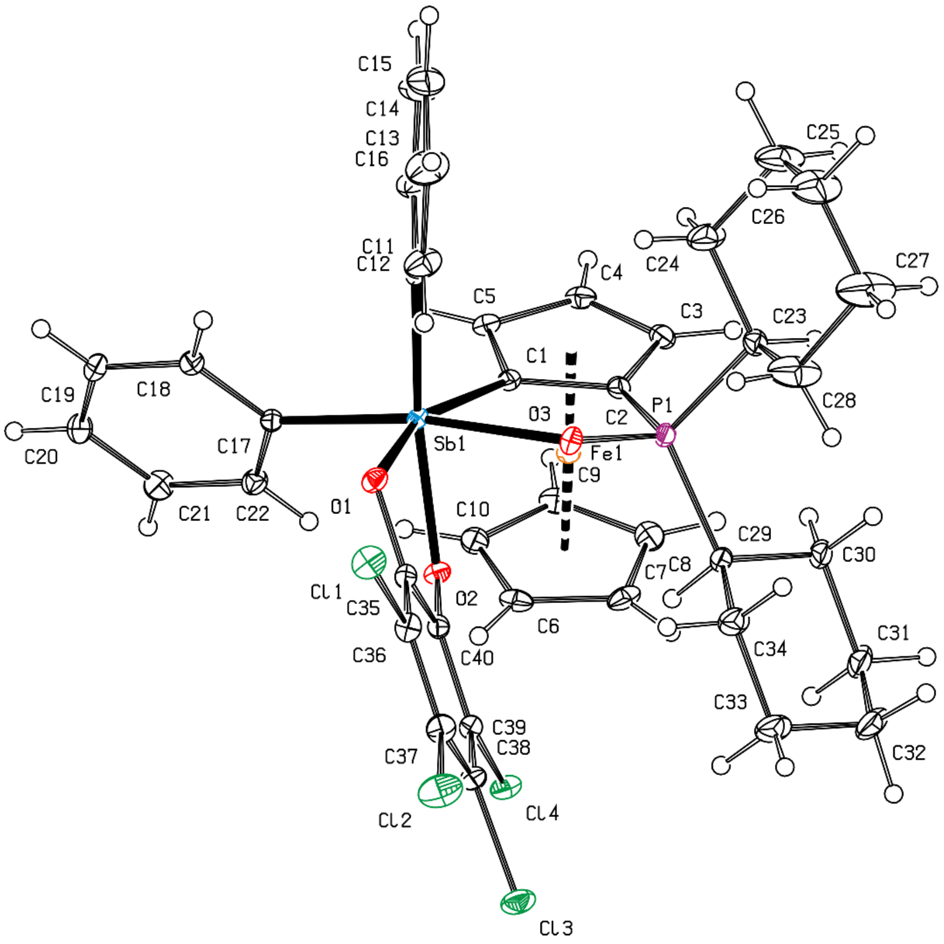

Figure S12. PLATON plot of the molecular structure of **3O** showing 30% probability ellipsoids.

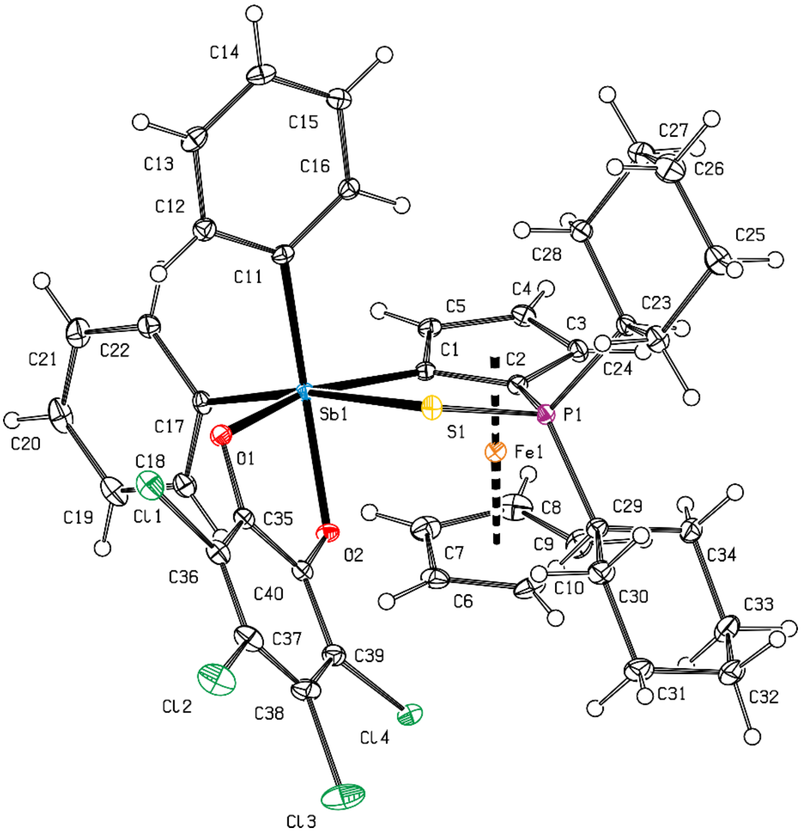

Figure S13. PLATON plot of the molecular structure of **3S**·2.7CHCl$_3$ showing 30% probability ellipsoids. The solvent was eliminated using PLATON/SQUEEZE.

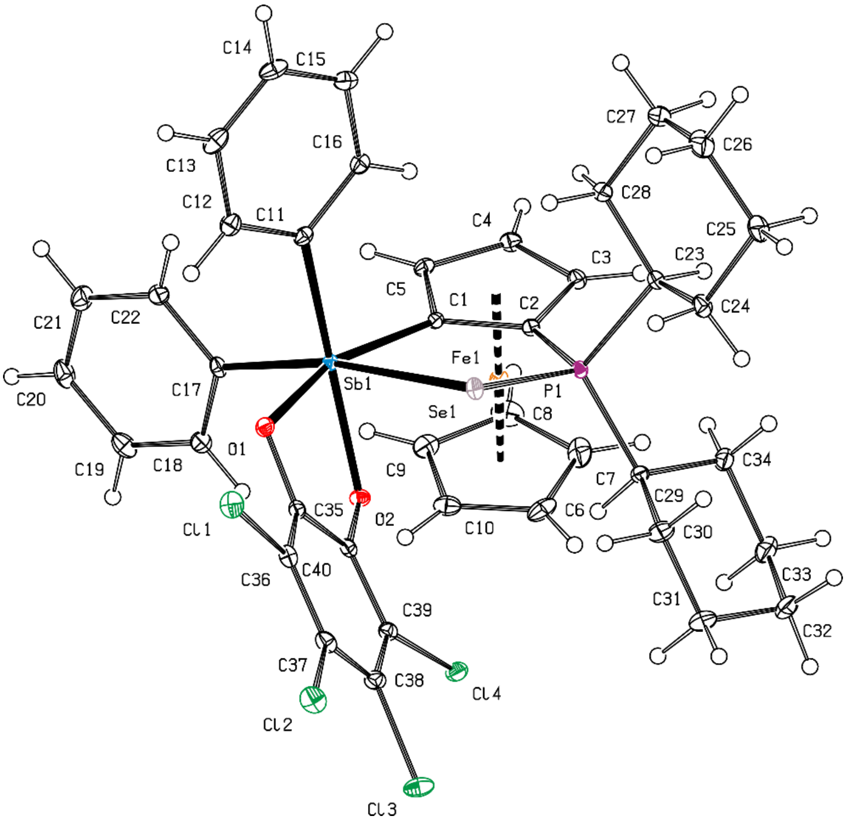

Figure S14. PLATON plot of the molecular structure of **3Se** showing 30% probability ellipsoids. The solvent was omitted for clarity.

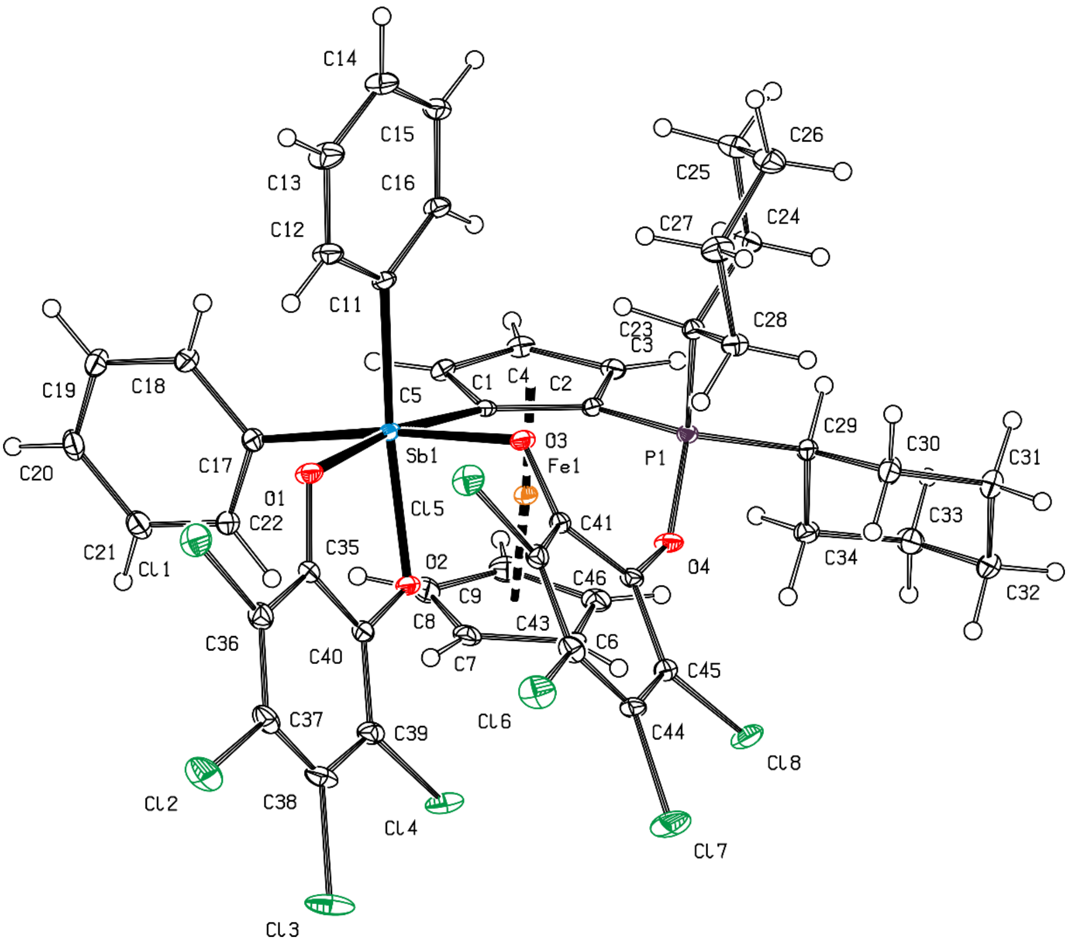

Figure S15. PLATON plot of the molecular structure of **4** showing 30% probability ellipsoids.

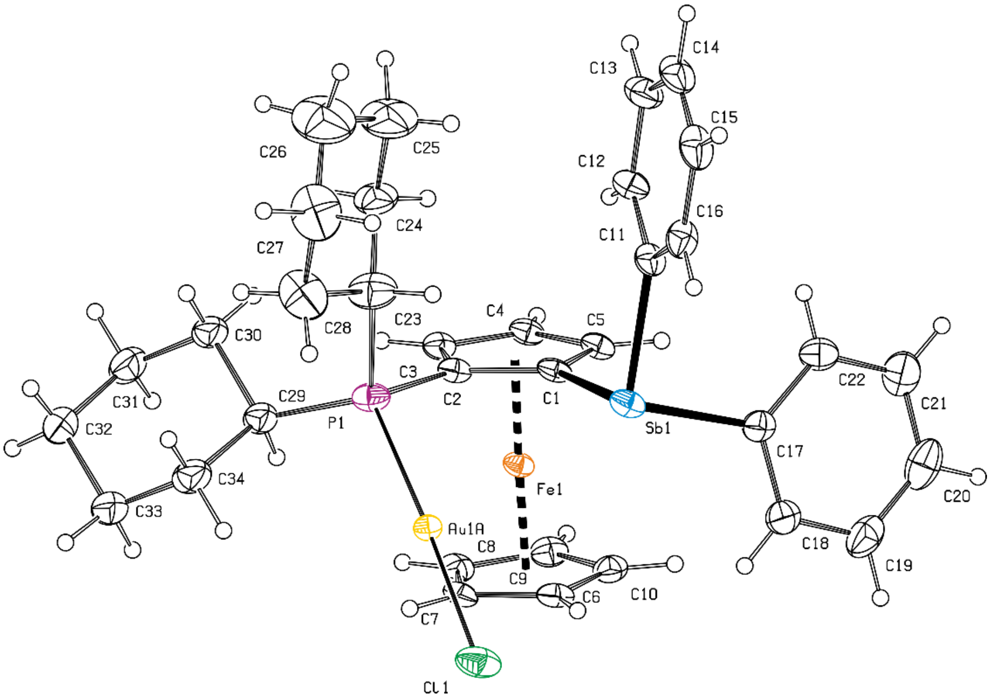

Figure S16. PLATON plot of the molecular structure of **5** showing 30% probability ellipsoids. For clarity, only one position of the disordered gold atom is depicted.

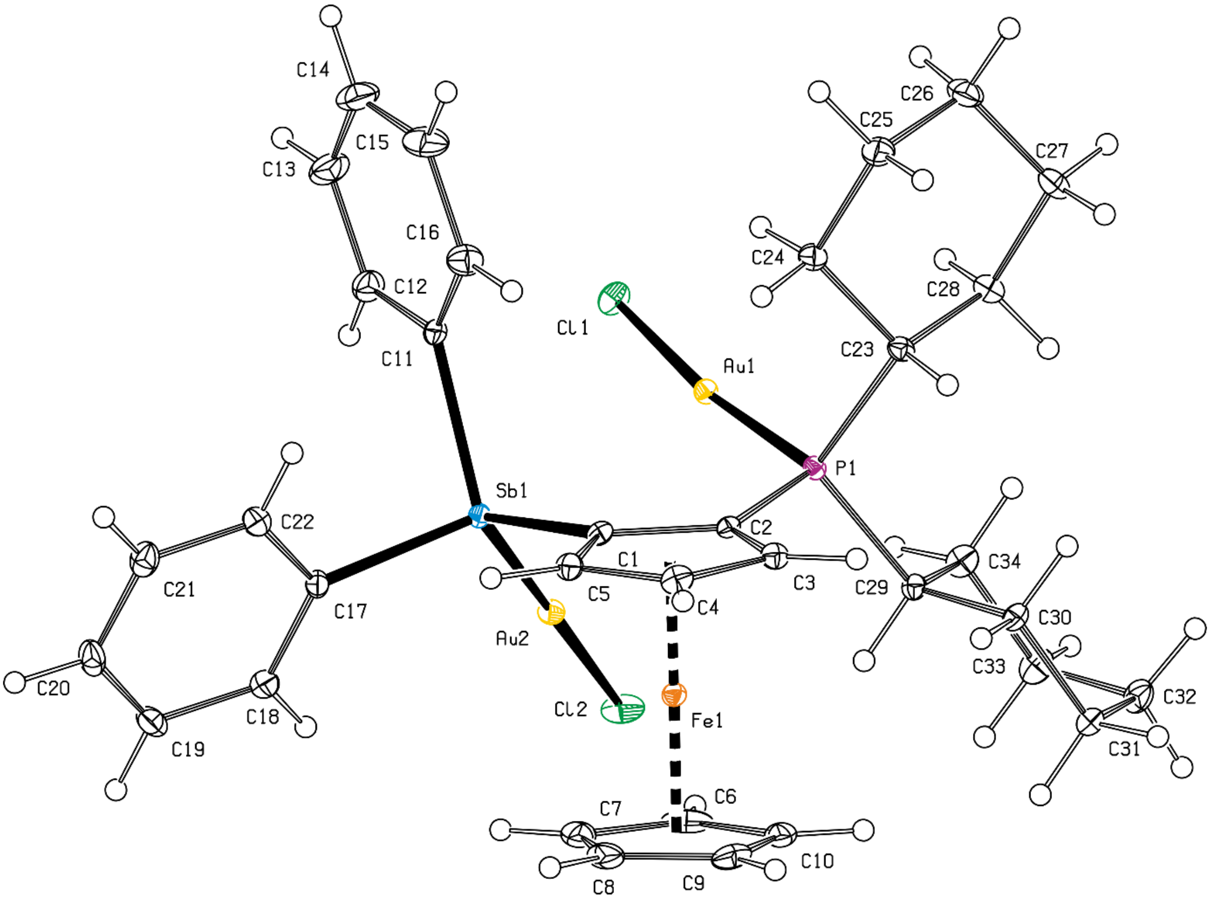

Figure S17. PLATON plot of the complex molecule in the structure of **6**·CH_2Cl_2 showing 30% probability ellipsoids.

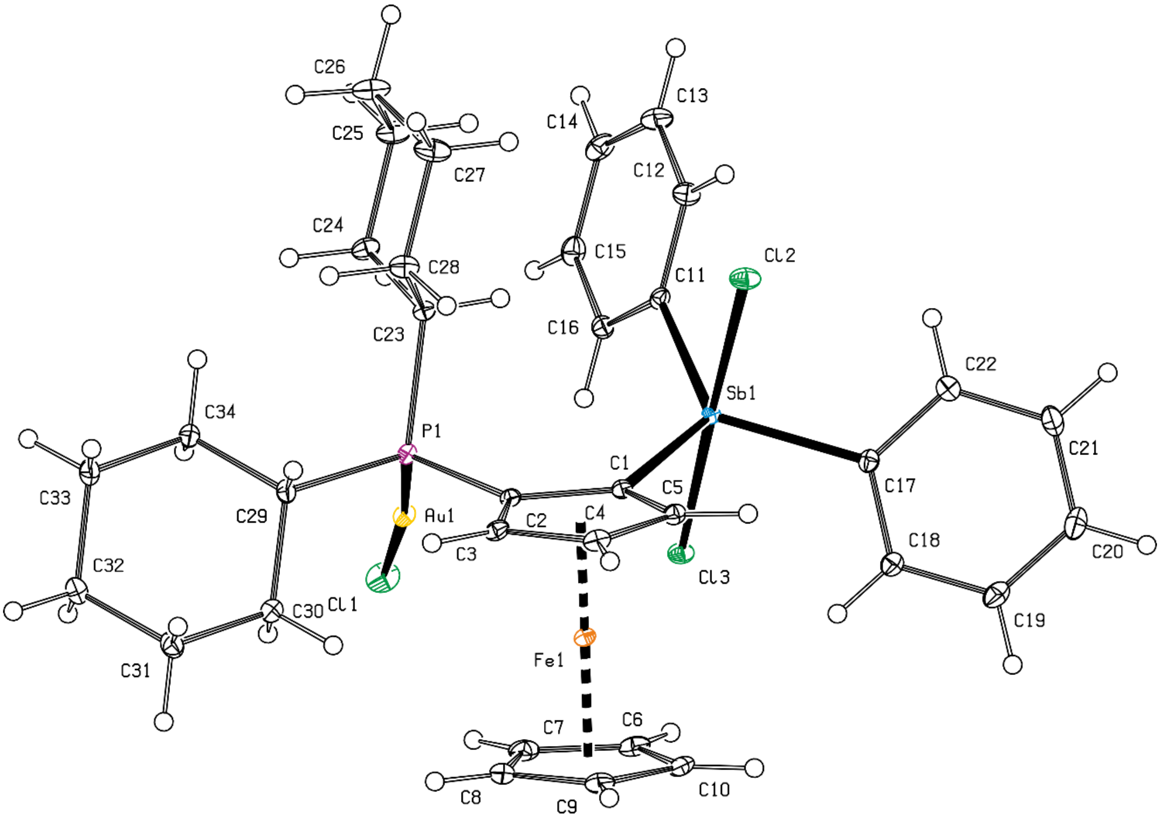

Figure S18. PLATON plot of the molecular structure of **7**·$2CHCl_3$ showing 30% probability ellipsoids. The solvent was omitted for clarity.

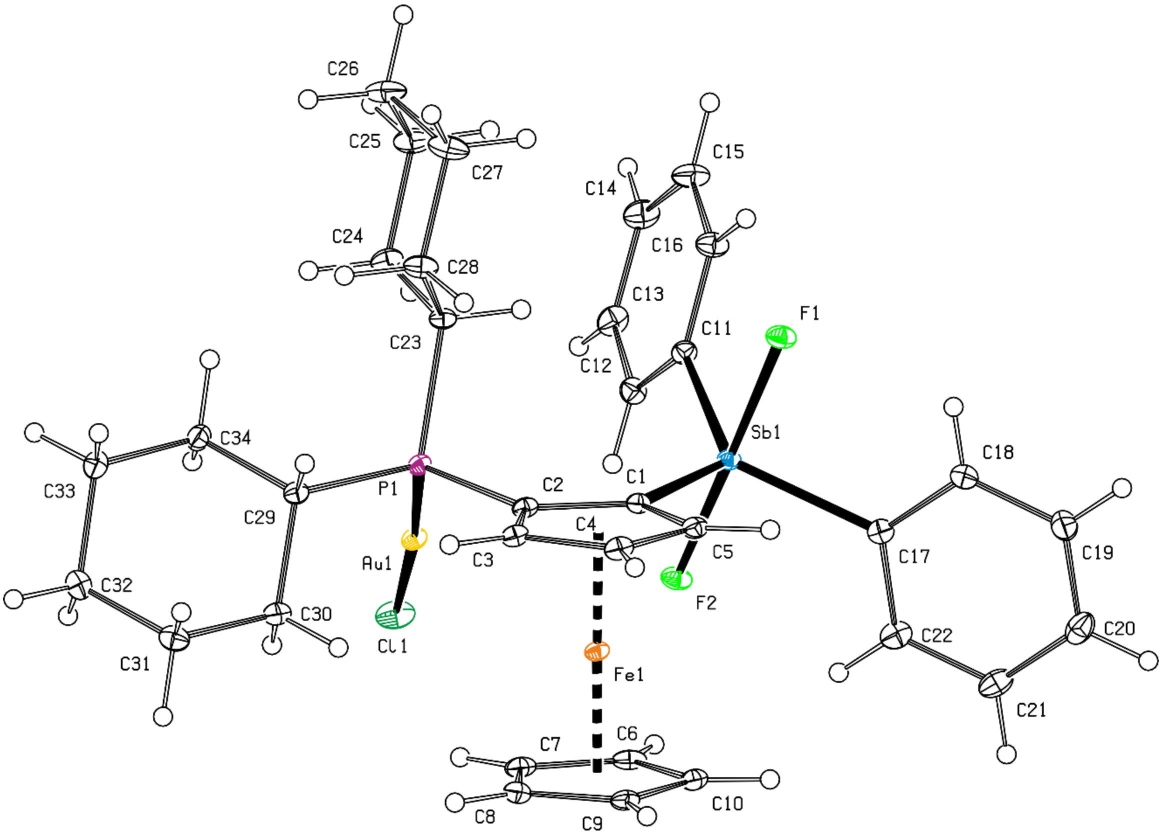

Figure S19. PLATON plot of the molecular structure of **8**·$1.7CHCl_3$ showing 30% probability ellipsoids. The solvent was eliminated by PLATON/SQUEEZE.

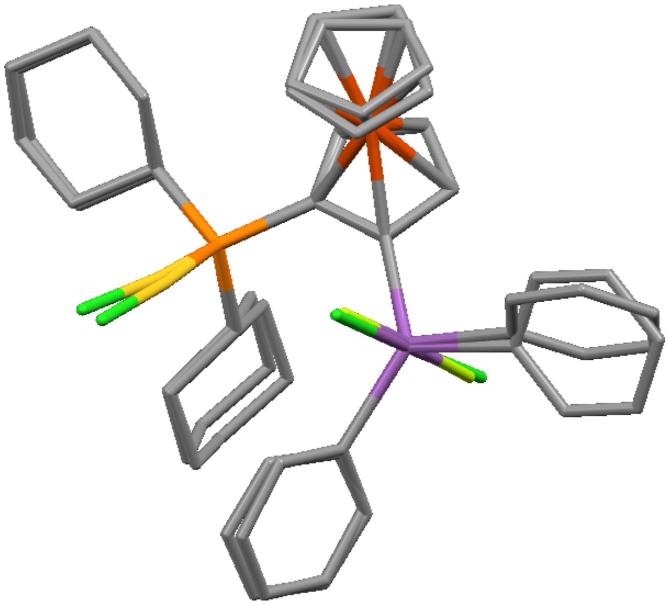

Figure S20. Overlap of the molecules of stiboranes **7** and **8** (F – yellow green, Cl – green).

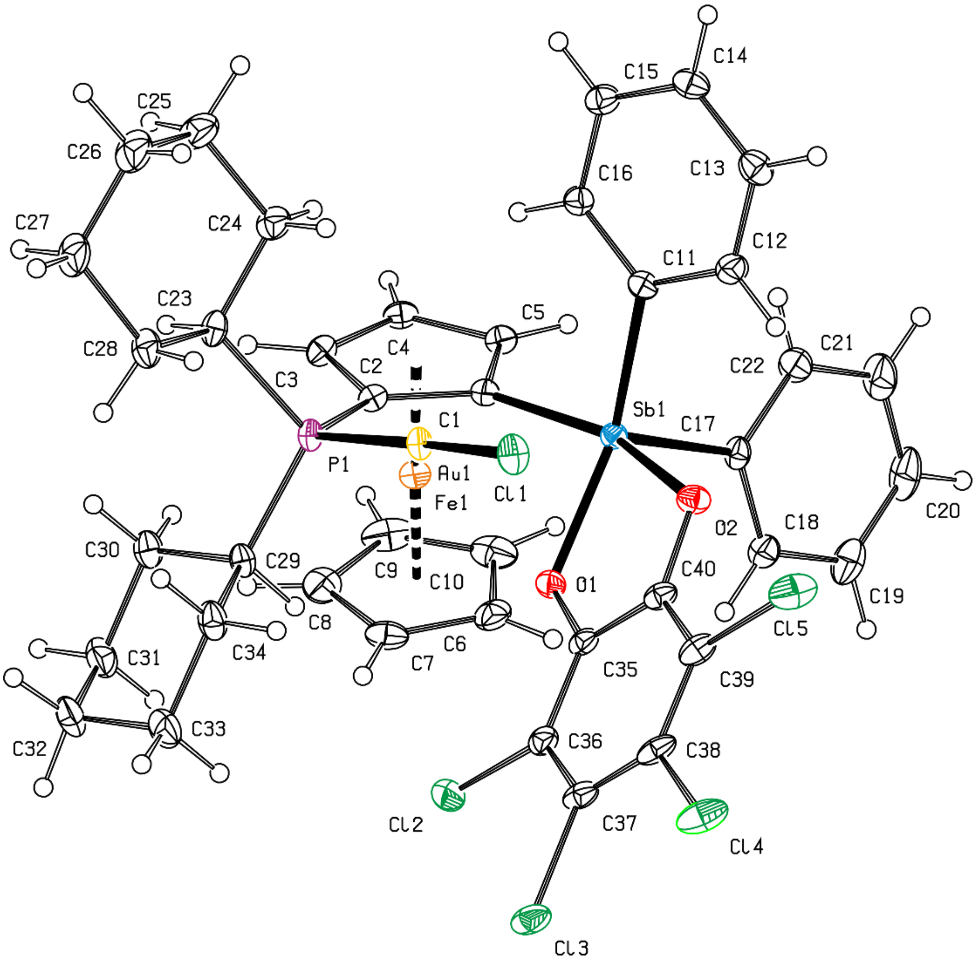

Figure S21. PLATON plot of the molecular structure of **9**·$1.2C_5H_{12}$ showing 30% probability ellipsoids. The solvent was eliminated by PLATON SQUEEZE.

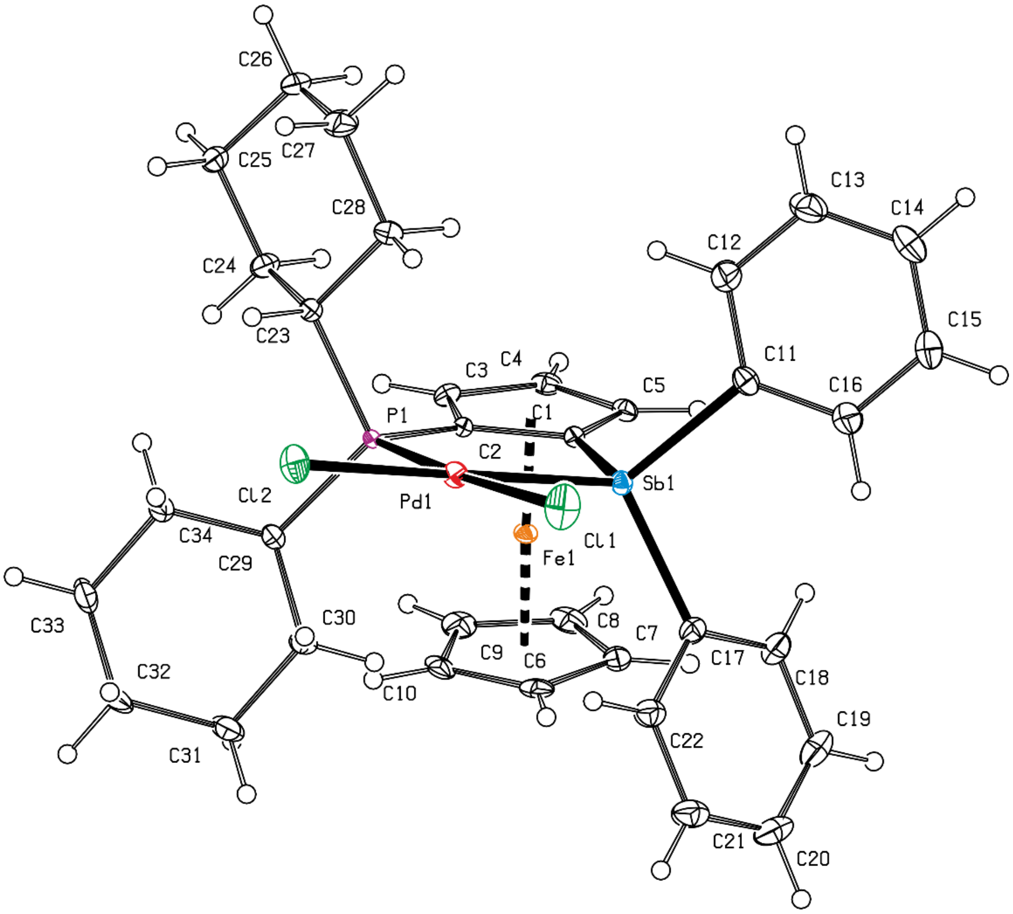

Figure S22. PLATON plot of the molecular structure of **12** showing 30% probability ellipsoids.

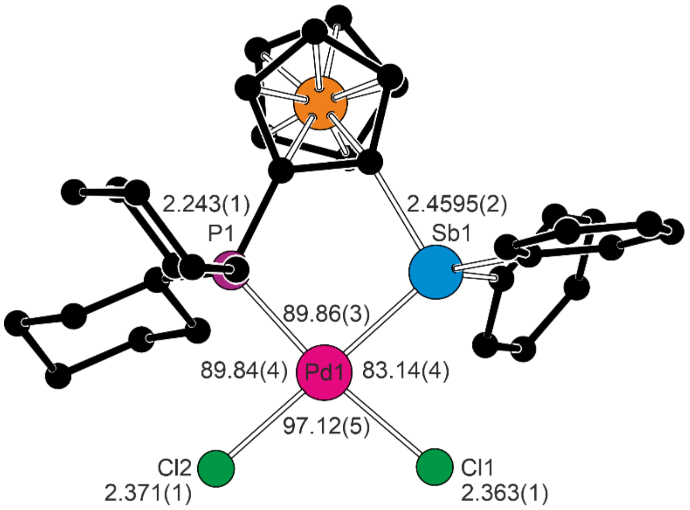

Figure S23. Pd-donor distances (in Å) and interligand angles (in deg) in the molecule of **12**.

DFT CALCULATIONS

Theoretical calculations were performed using the Gaussian 16 program package.[13] When available, the geometry optimizations were started from atomic coordinates determined by single-crystal X-ray diffraction analysis, using PBE0[14] density functional in conjunction def2-TZVP basis set[15] and associated effective core potential (ECP) for the heavy elements (Au, Sb)[16] with added Grimme's D3 dispersion correction.[17] Solvent effects (chloroform) have been approximated using the PCM model.[18] The analysis of calculated electron densities by the Atoms in Molecules approach (AIM) were performed using the Multiwfn software package (version 3.8).[19] The methyl (MCA) and fluoride affinities (FA) were calculated according to the literature procedures.[20,21] The fluoride affinities were calculated using the geometries and the thermal corrections obtained at the PBE0(d3)/def2-TZVP:sdd(Fe,Sb) level of theory combined with the PW6B95(d3bj)/def2-qzvpp single-point electronic energies.[22] Molecular orbitals were visualized using the Avogadro programme.[23] Intrinsic bond orbital (IBO) analysis and visualization of the obtained orbitals were performed using the IboView software.[24]

Table S8. Electron density (ρ_{bcp}) and its Laplacian ($\nabla^2\rho_{bcp}$), the total electronic density (H), the ratio of the potential and kinetic energy density ($|V|/G$), and the ratios of kinetic (G/ρ_{bcp}) and total energy density (H/ρ_{bcp}) to the electron density at the bond critical points located between antimony and the respective donor atom (O, S, and Se) and the corresponding bond distances.

Compd	Bond	Bond length [Å]		ρ_{bcp}	$\nabla^2\rho_{bcp}$	H	$\|V\|/G$	G/ρ_{bcp}	H/ρ_{bcp}
		exp.	calc.[b]	[a.u.]	[a.u.]	(a.u.)	(a.u.)	(a.u.)	(a.u.)
**1O**	Sb···O	2.828(2)	2.902	0.019	0.053	$0.05\cdot10^{-2}$	0.96	0.67	0.028
**1S**	Sb···S	3.707(1)/ 3.735(1)[a]	3.678	0.010	0.021	$0.07\cdot10^{-2}$	0.86	0.46	0.064
**1Se**	Sb···Se	3.8275(7)	3.748	0.011	0.019	$0.04\cdot10^{-2}$	0.92	0.42	0.034

[a] Distances for two crystallographically independent molecules. [b] Calculated at the PBE0(d3)/def2-TZVP/ECP(Sb)/PCM($CHCl_3$) level of theory.

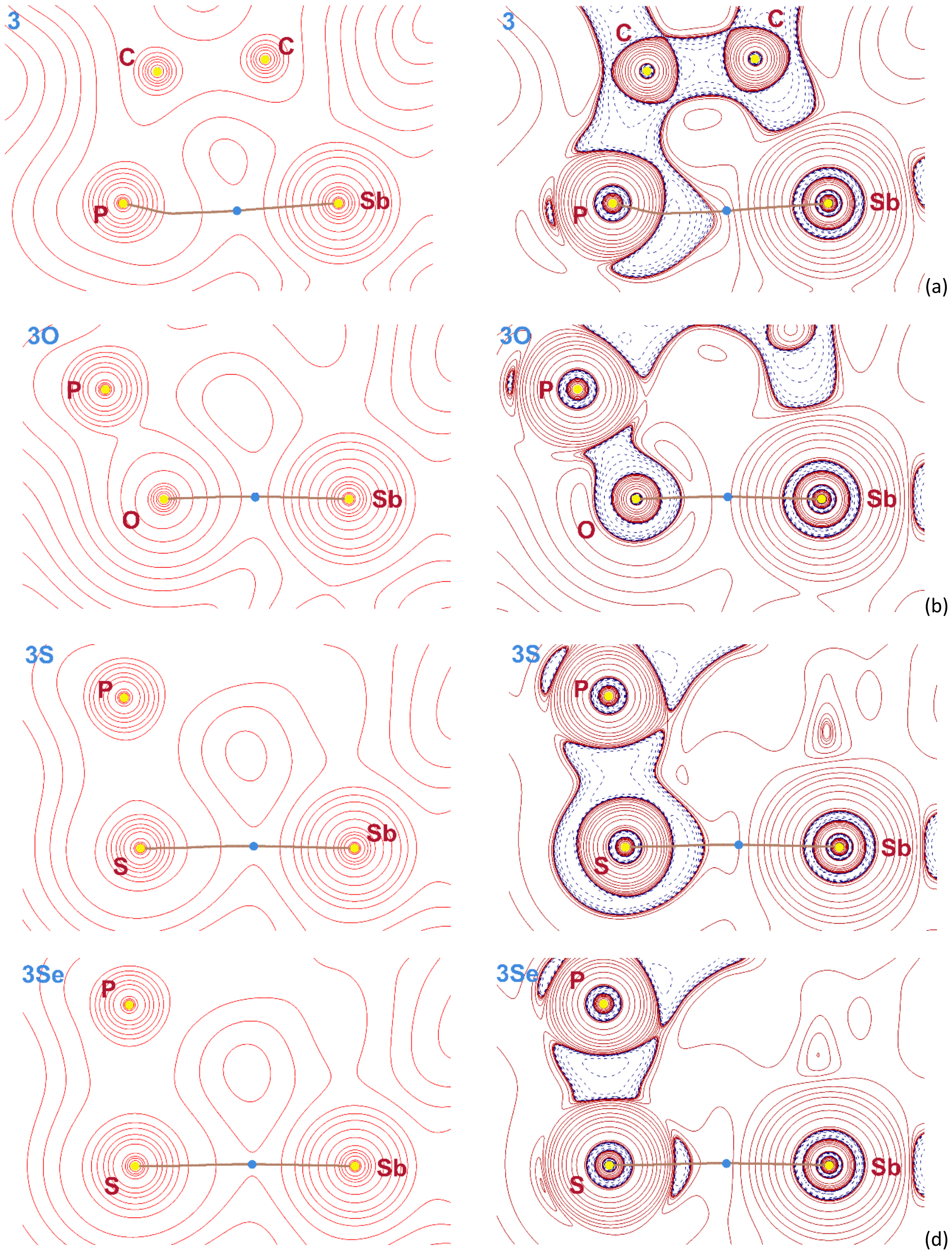

Figure S24. Contour plots of electron density ρ_{bcp} (left) and the corresponding Laplacians $\nabla^2\rho_{bcp}$ (right; positive – full red lines, negative – dashed blue lines) in the plane defined either by the pivotal C, P and Sb atoms (**3**) or by the chalcogen atoms (O, S or Se), P and Sb (**3O**, **3S**, and **3Se**). The yellow and blue dots indicate the positions of nuclear and bond critical points, respectively; brown lines highlight the bond paths.

Table S9. Electron density (ρ_{bcp}), Laplacian of the electron density ($\nabla^2\rho_{bcp}$), total electronic density (H.), the ratio of potential and kinetic energy density ($|V|/G$), and the ratios of kinetic (G/ρ_{bcp}) and total energy density (H/ρ_{bcp}) to electron density at the bond critical points located between the antimony and gold atoms in **5** and **9**, and the corresponding interatomic distances.

Compd	Bond	Bond length [Å]		ρ_{bcp}	$\nabla^2\rho_{bcp}$	H_{bcp}	$\|V\|/G$	G/ρ_{bcp}	H/ρ_{bcp}
		exp.	calc.[b]	[a.u.]	[a.u.]	(a.u.)	(a.u.)	(a.u.)	(a.u.)
**5**	Au···Sb	–[a]	3.256	0.022	0.038	$-0.15\cdot10^{-2}$	1.38	0.51	−0.07
**9**	Au···Sb	3.363	3.282	0.019	0.030	$-0.14\cdot10^{-2}$	1.16	0.47	−0.07

[a] Value affected by disorder. [b] Calculated at the PBE0(d3)/def2-TZVP/ECP(Sb) level of theory.

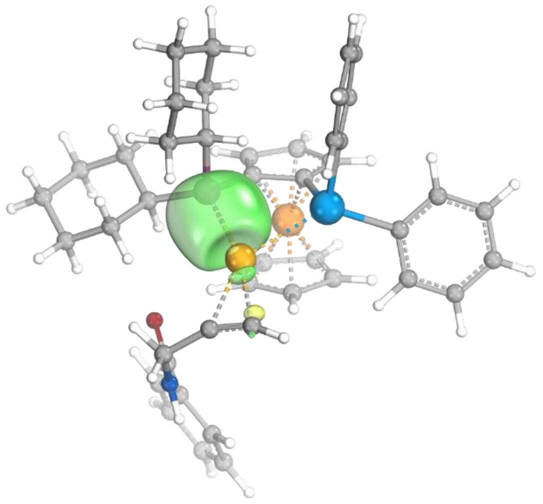

P(lp) → d(Au) [1.54(P)/0.35(Au)/0.05(C)]

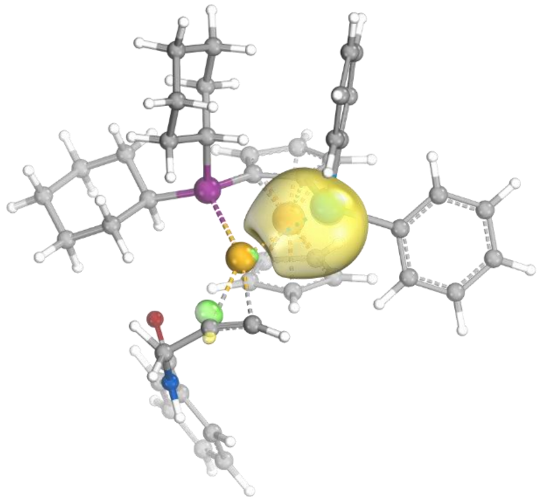

Sb(lp) → d(Au) [1.78(Sb)/0.10(Au)/0.07(C)]

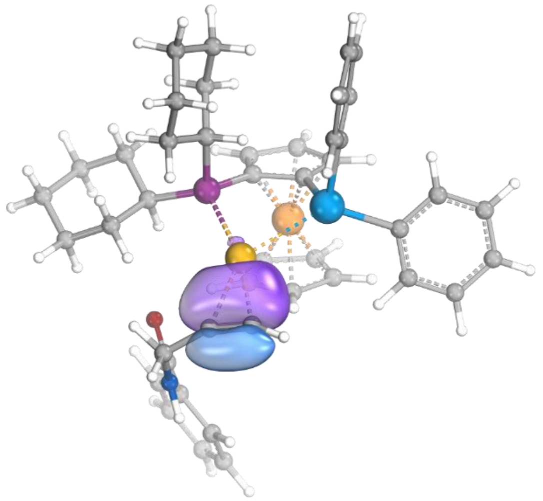

π(C≡C) → d(Au) [0.94(C):0.78(C)/0.26(Au)]

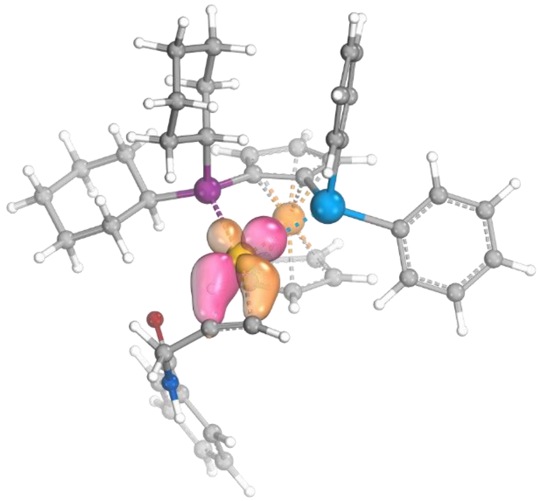

d(Au) → π*(C≡C) [1.74(Au)/0.10(C):0.13(C)]

Figure S25. Selected intrinsic bond orbitals (IBOs) of the hypothetical cationic alkyne-gold(I) complex **5-S**. Values in parentheses indicate the fraction of bonding electrons assigned to the individual atoms.

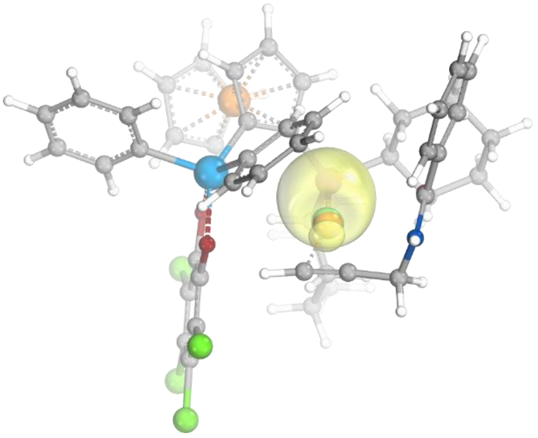

P(lp) → d(Au) [1.42(P)/0.53(Au)/0.05(other)]

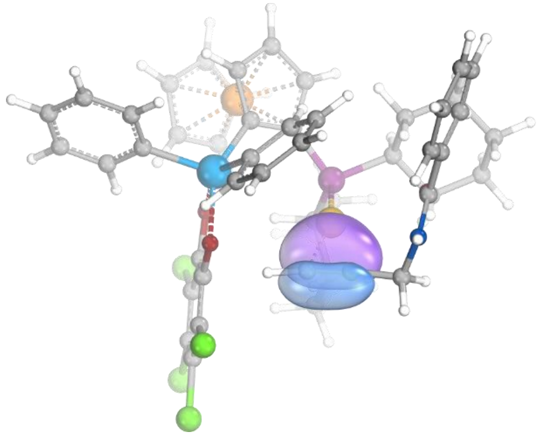

π(C≡C) → d(Au) [0.92(C):0.86(C)/0.19(Au)]

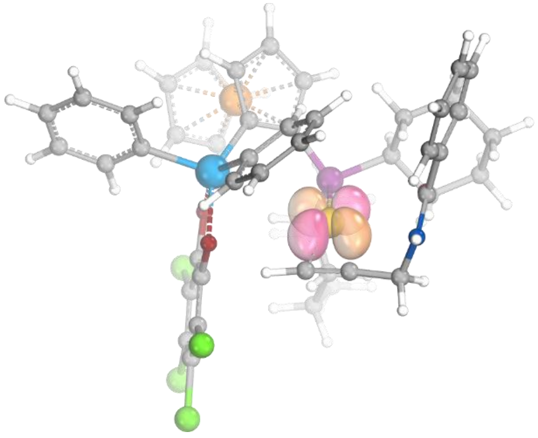

d(Au) → π*(C≡C) [1.89(Au)/0.04(C):0.04(C)]

Figure S26. Selected intrinsic bond orbitals (IBOs) of hypothetical cationic alkyne-gold(I) complex **9-S**. Values in parentheses indicate the fraction of bonding electrons assigned to the individual atoms.

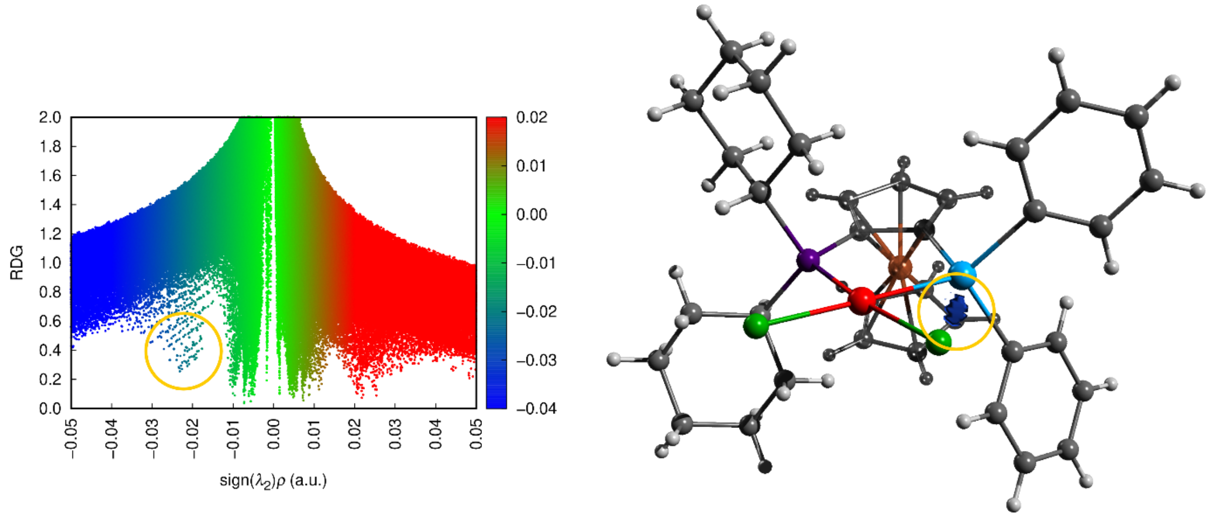

Figure S27. (left) Scatter plot of the reduced density gradient (RDG) versus the product of electron density and the sign of the second Hessian eigenvalue (sign(λ_2)ρ) and (right) NCI plot of complex **12**. For clarity, only RDG isosurface with $S(r)$ = 0.5 and the sign(λ_2)ρ ranging from −0.050 to −0.015 (corresponding to attractive noncovalent interactions) is shown.

COPIES OF THE NMR SPECTRA

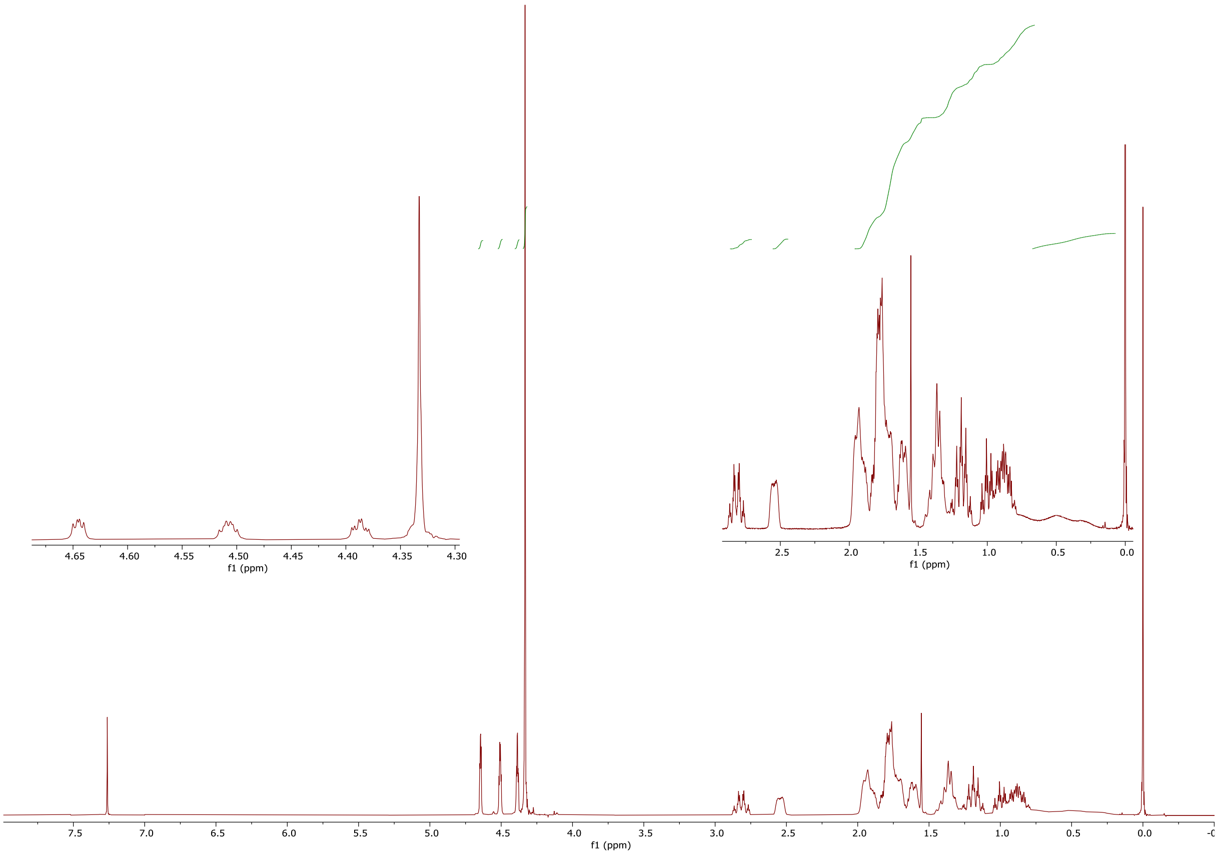

Figure S28. ^{1}H NMR spectrum (399.95 MHz, $CDCl_3$) of **2**·BH_3.

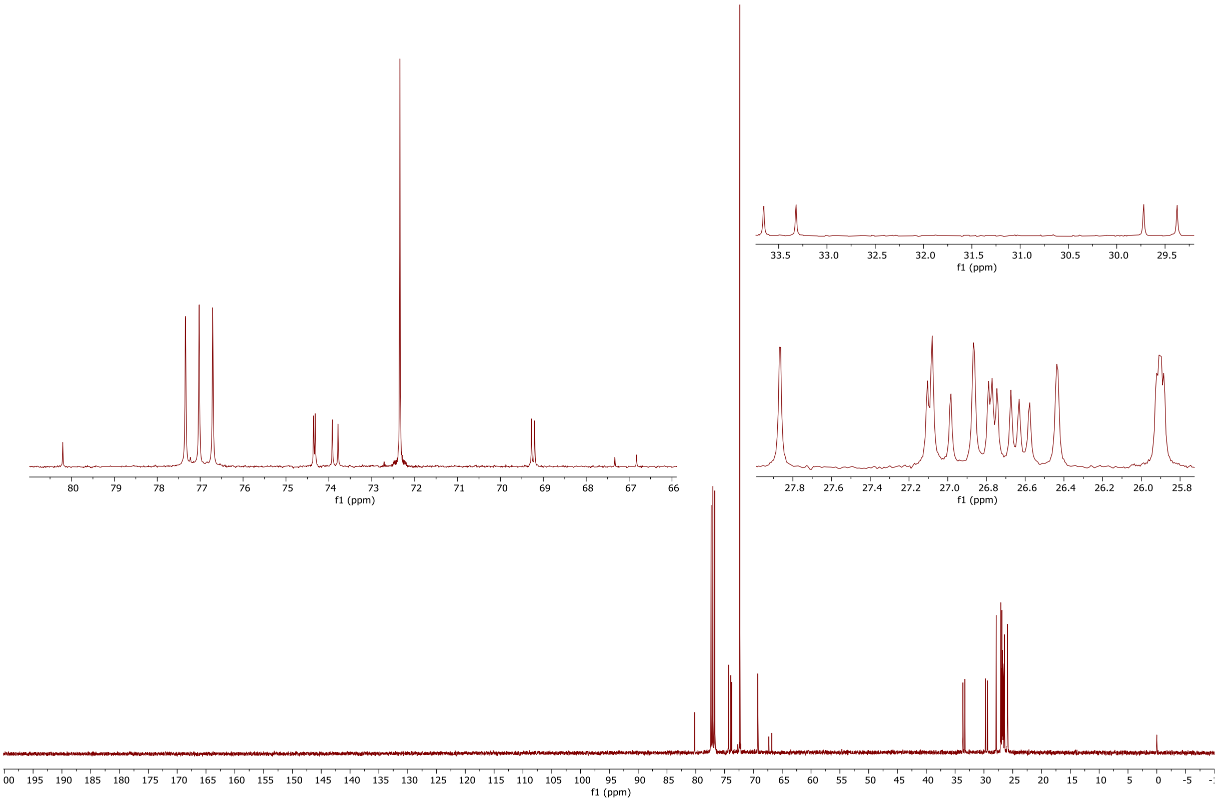

Figure S29. $^{13}C\{^{1}H\}$ NMR spectrum (100.58 MHz, $CDCl_3$) of **2**·BH_3.

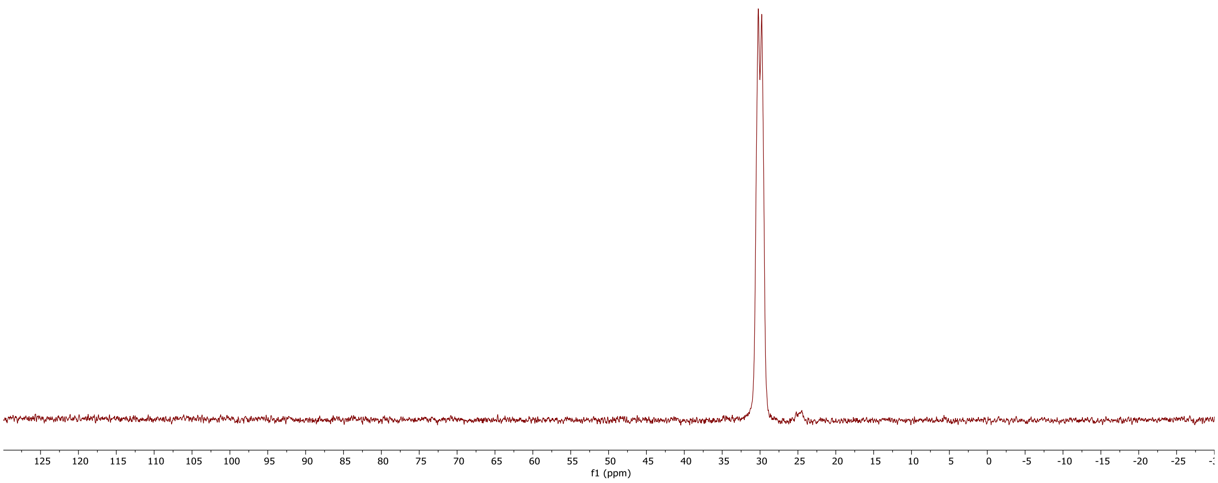

Figure S30. $^{31}P\{^{1}H\}$ NMR spectrum (161.90 MHz, $CDCl_3$) of **2**·BH_3.

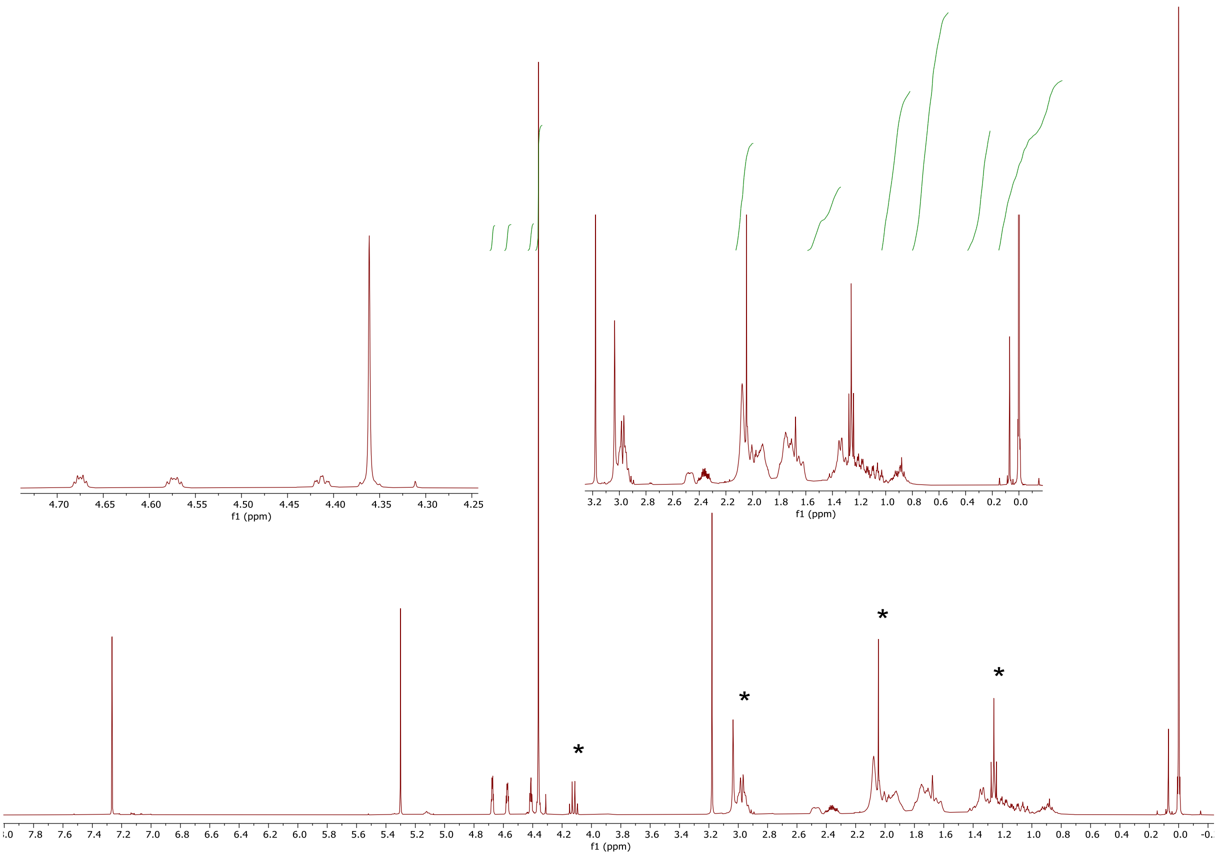

Figure S31. ^{1}H NMR spectrum (400.13 MHz, $CDCl_3$) of **2O**.

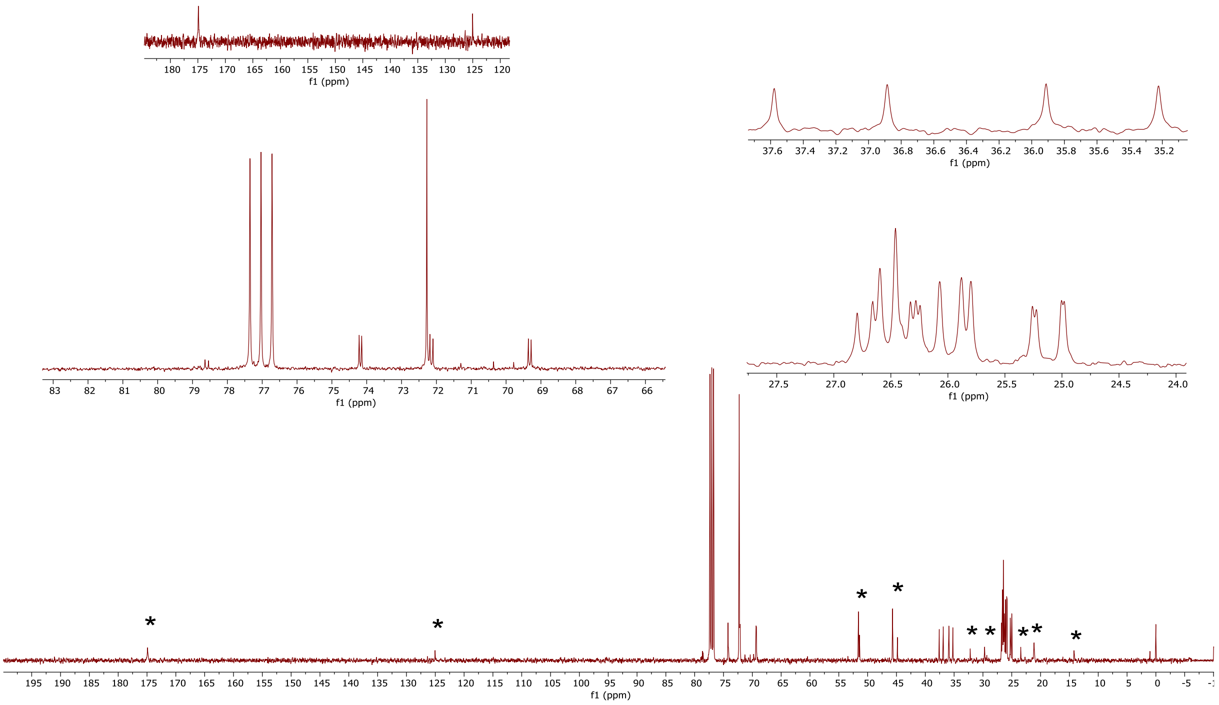

Figure S32. $^{13}C\{^{1}H\}$ NMR spectrum (100.61 MHz, $CDCl_3$) of **2O**.

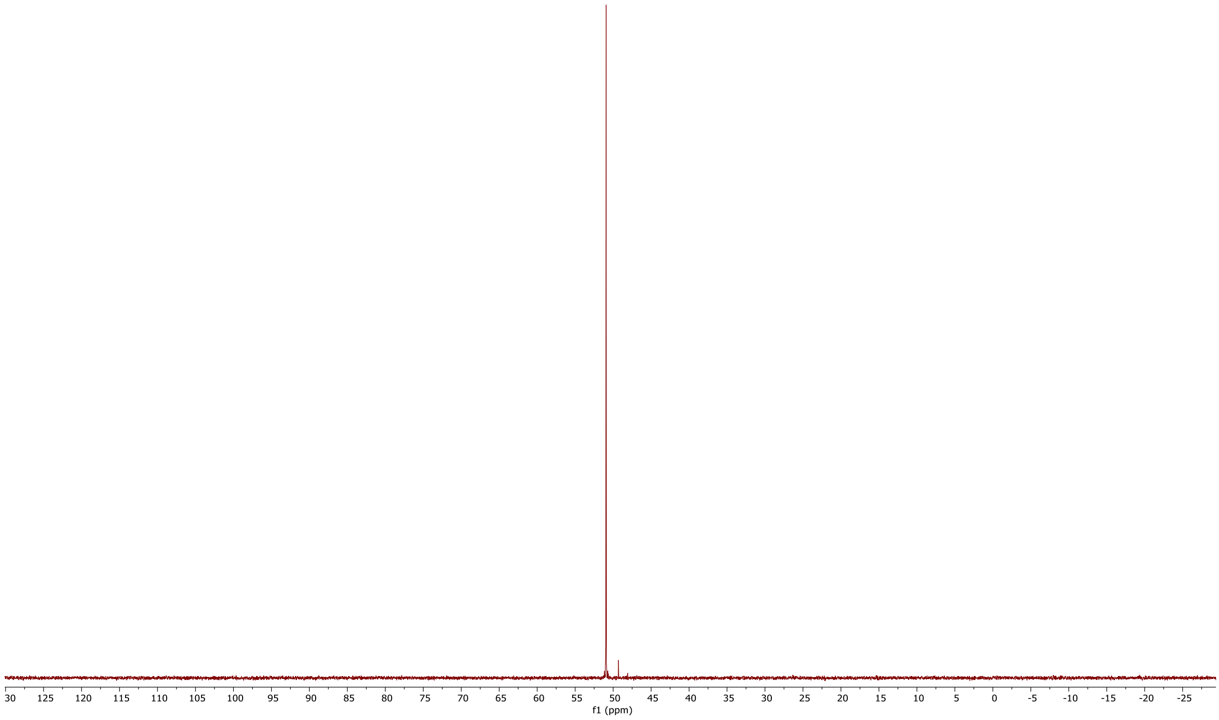

Figure S33. $^{31}P\{^{1}H\}$ NMR spectrum (161.98 MHz, $CDCl_3$) of **2O**.

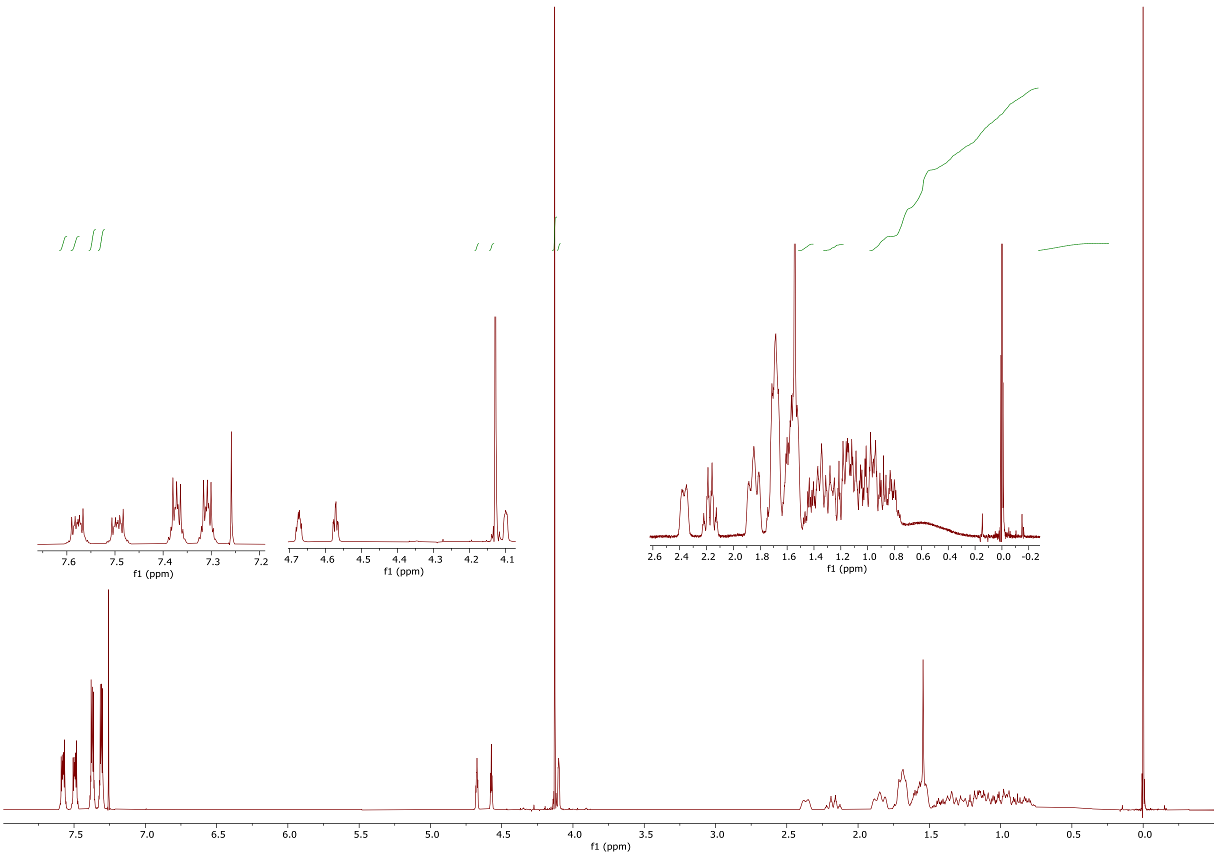

Figure S34. ^{1}H NMR spectrum (399.95 MHz, $CDCl_3$) of **1**·BH_3.

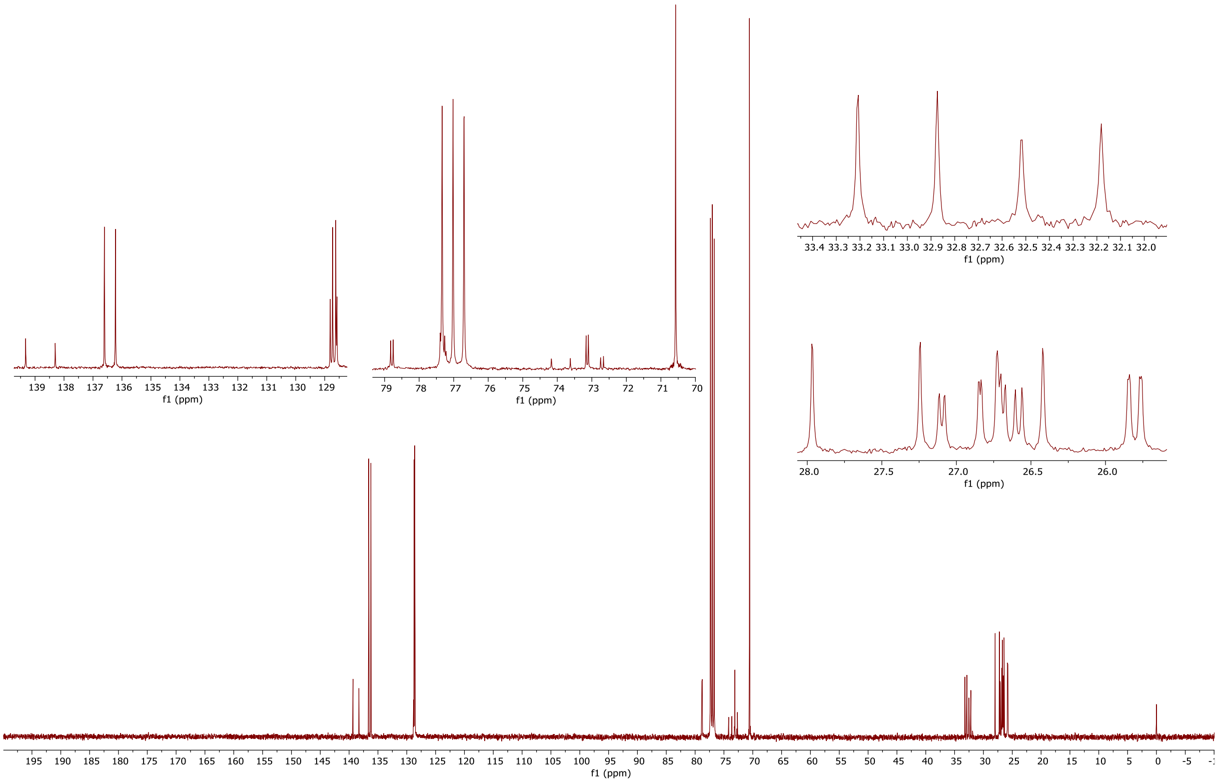

Figure S35. $^{13}C\{^{1}H\}$ NMR spectrum (100.58 MHz, $CDCl_3$) of **1**·BH_3.

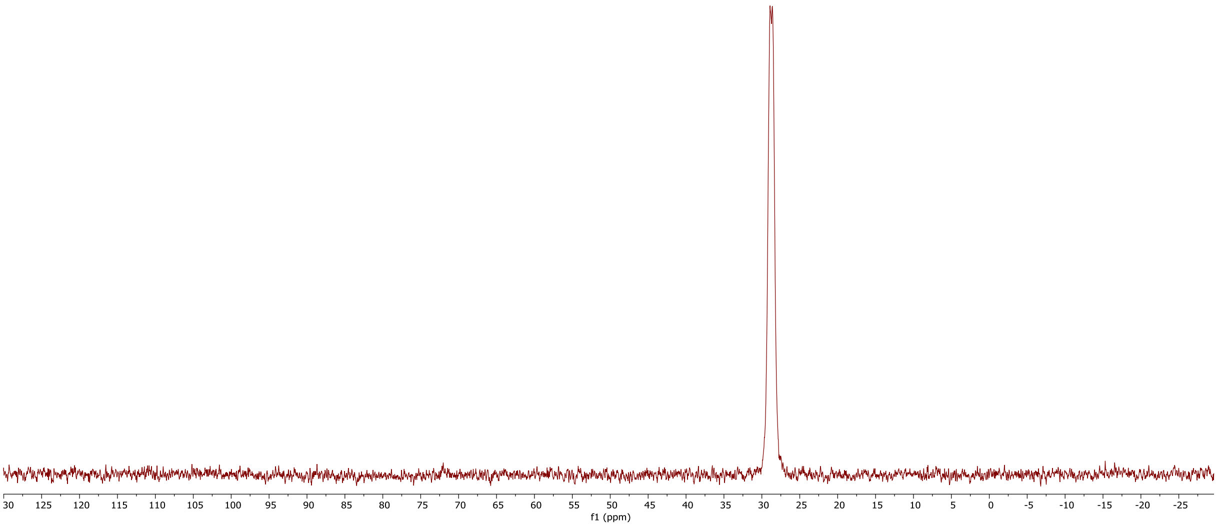

Figure S36. $^{31}P\{^{1}H\}$ NMR spectrum (161.90 MHz, $CDCl_3$) of **1**·BH_3.

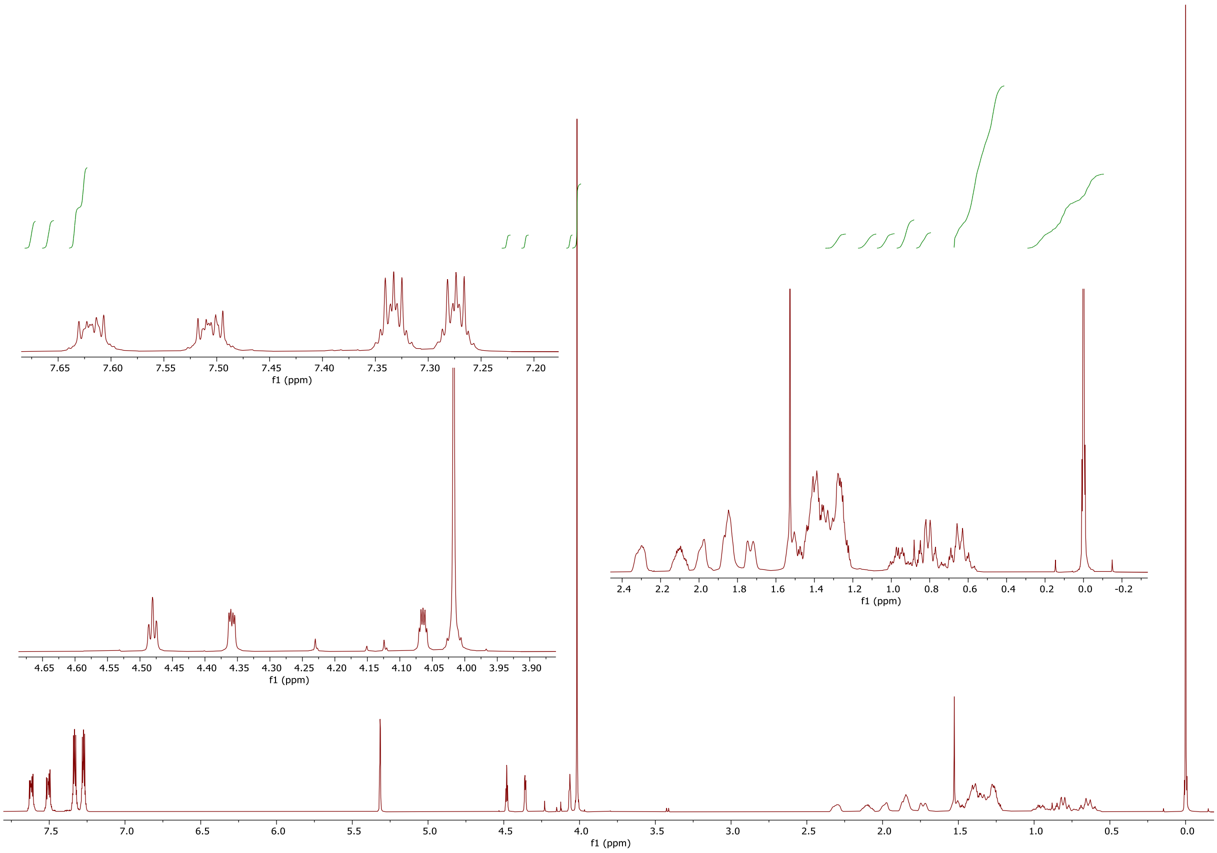

Figure S37. ^{1}H NMR spectrum (400.13 MHz, CD_2Cl_2) of **1**.

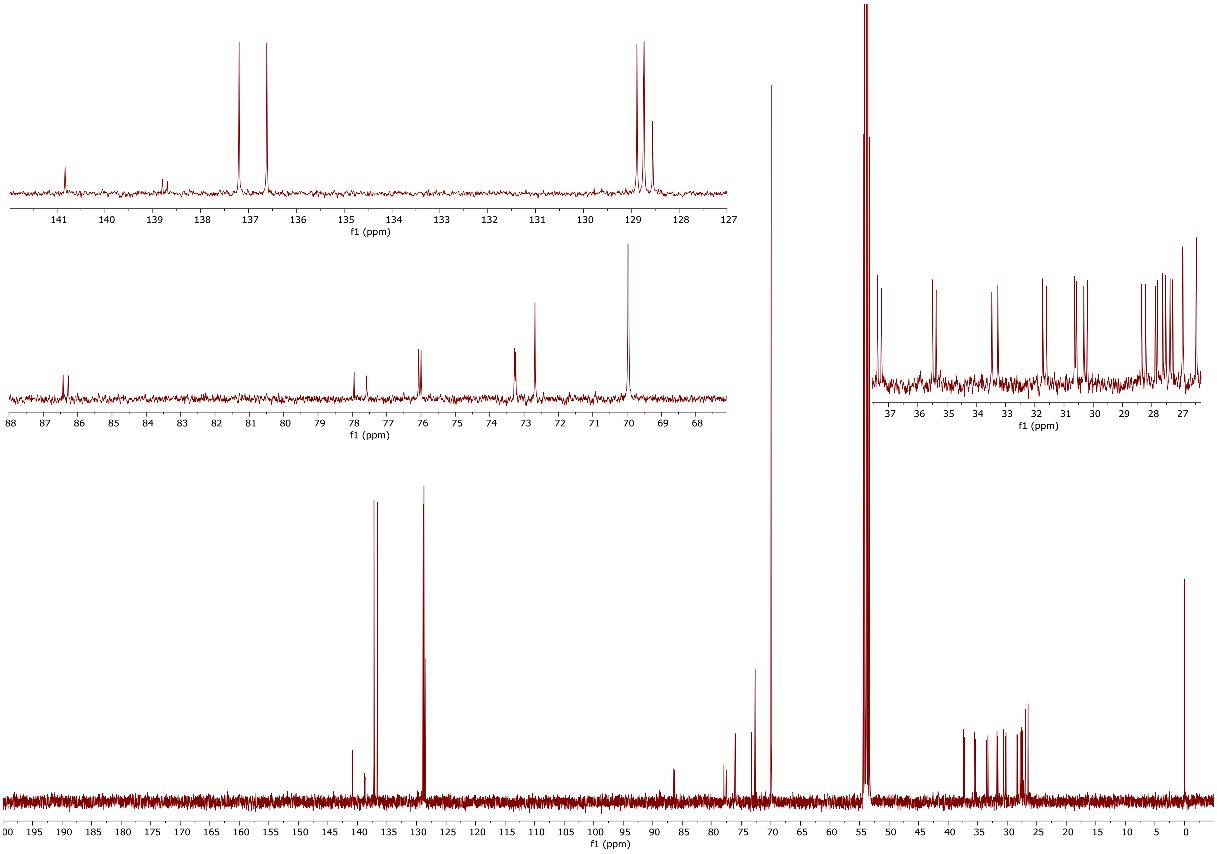

Figure S38. $^{13}C\{^{1}H\}$ NMR spectrum (100.61 MHz, CD_2Cl_2) of **1**.

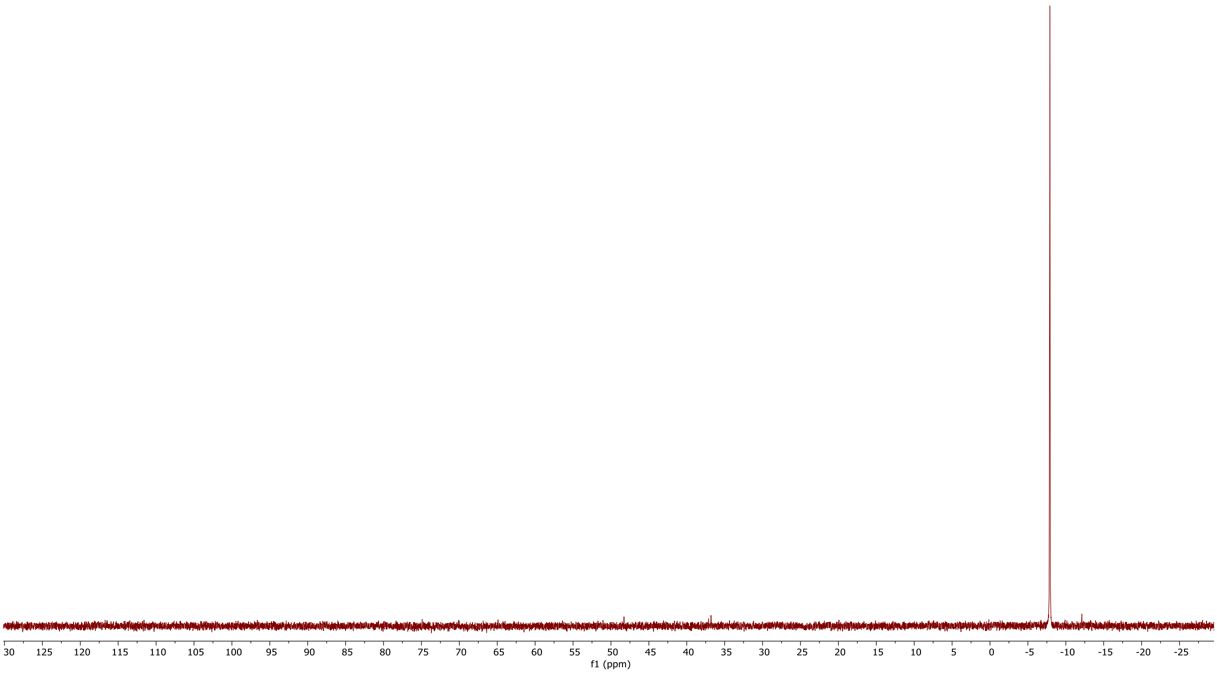

Figure S39. $^{31}P\{^1H\}$ NMR spectrum (161.98 MHz, CD_2Cl_2) of **1**.

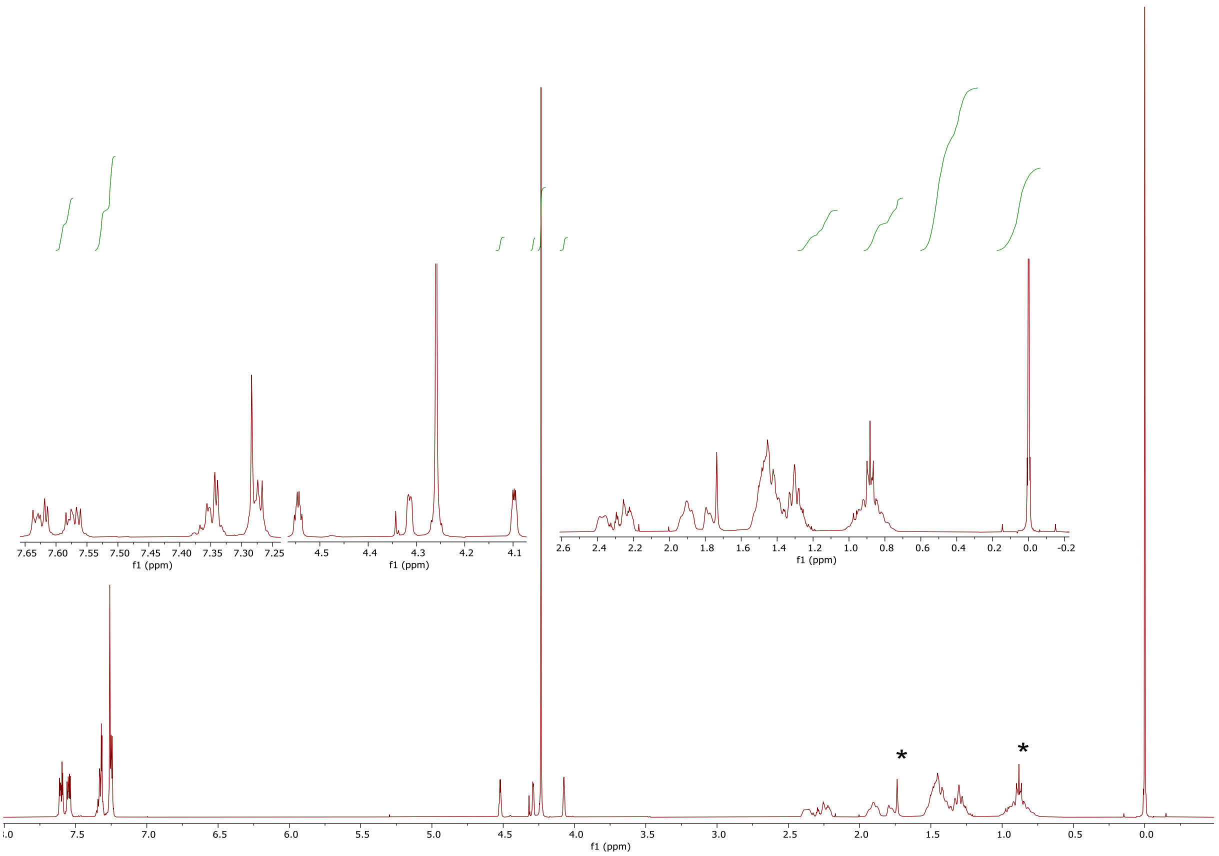

Figure S40. ^{1}H NMR spectrum (400.13 MHz, $CDCl_3$) of **1O**.

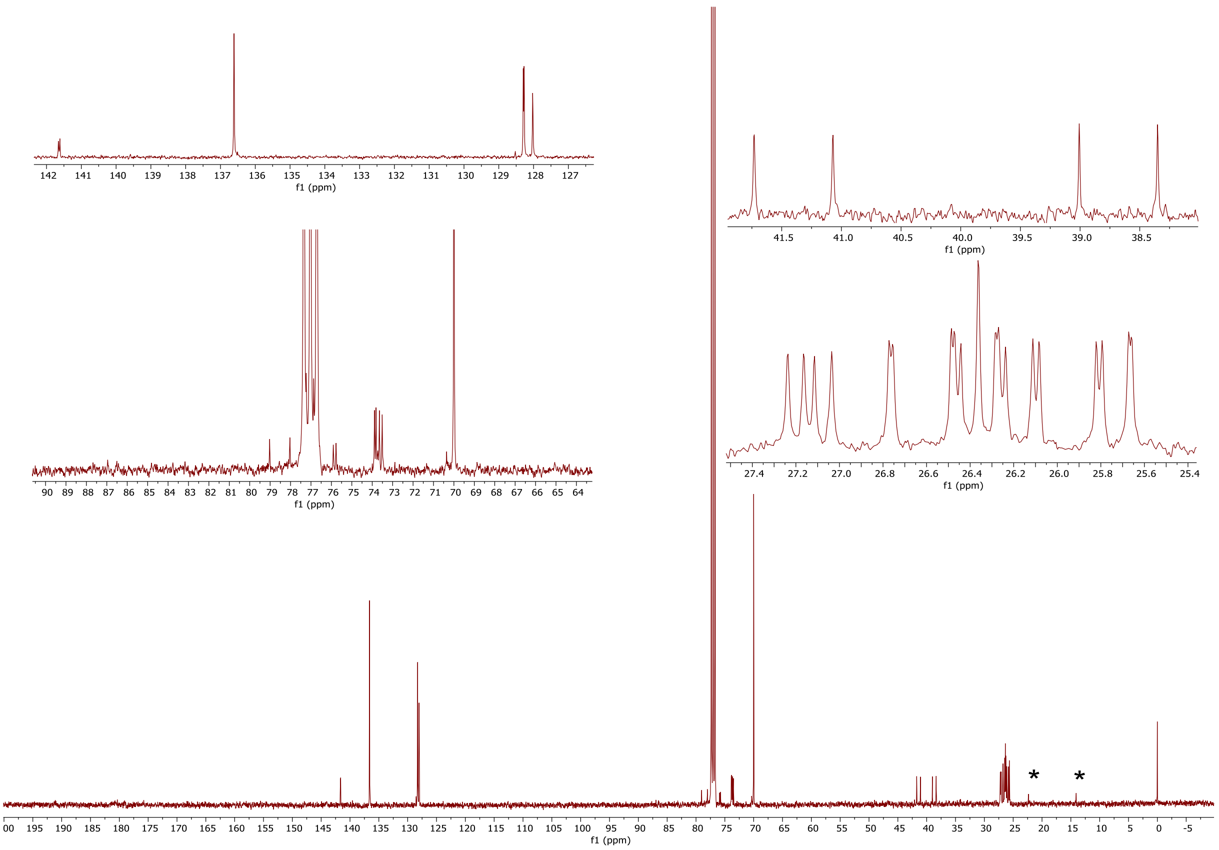

Figure S41. $^{13}C\{^{1}H\}$ NMR spectrum (100.61 MHz, $CDCl_3$) of **1O**.

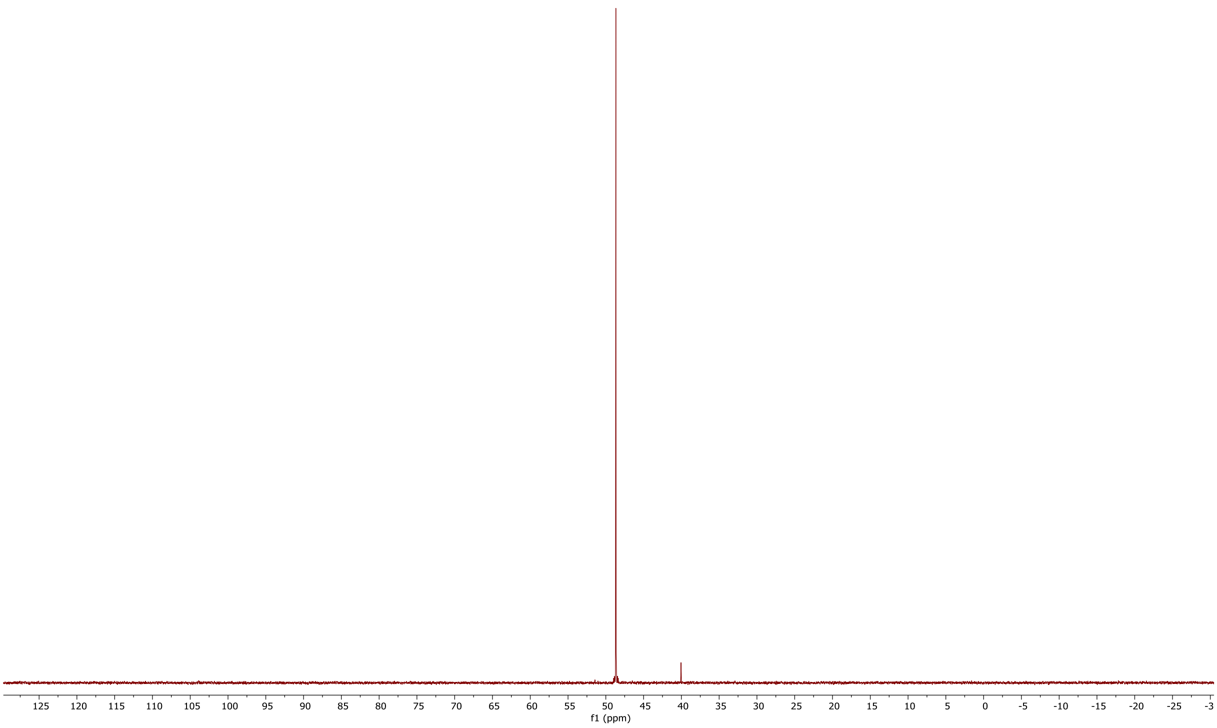

Figure S42. $^{31}P\{^{1}H\}$ NMR spectrum (161.98 MHz, $CDCl_3$) of **1O**.

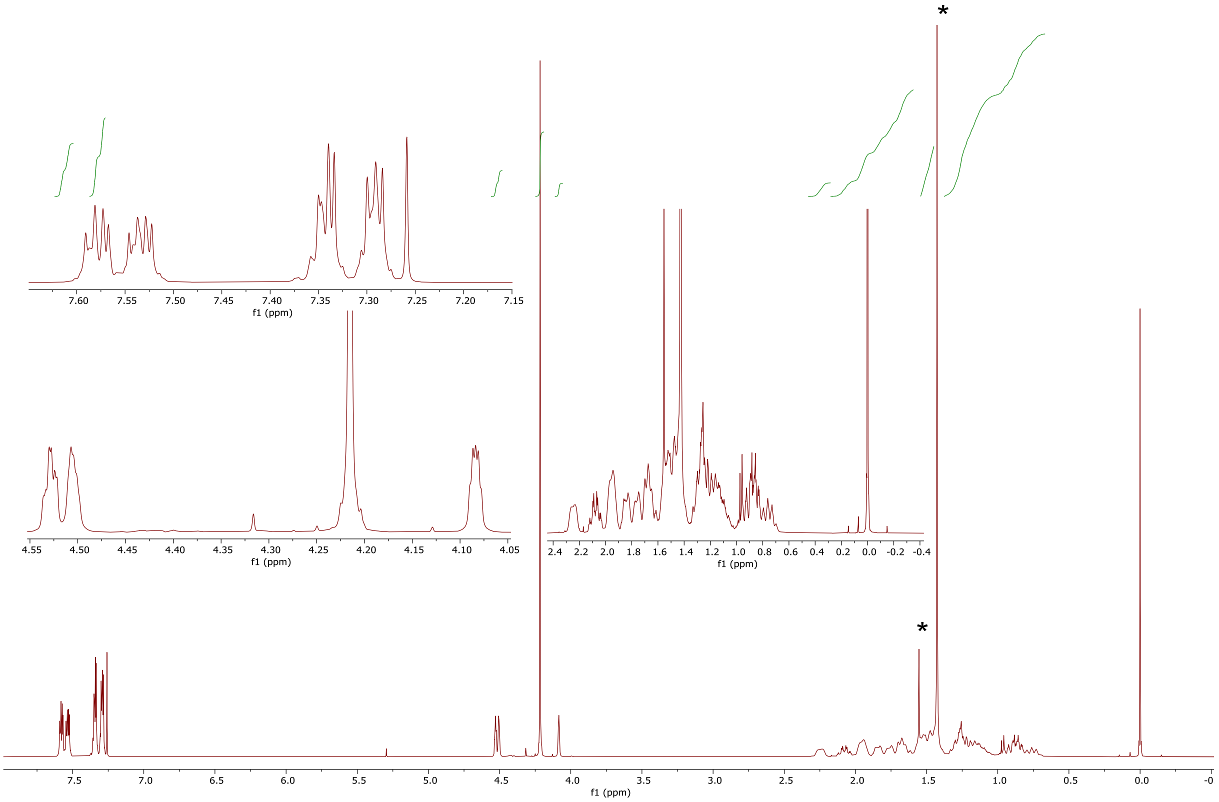

Figure S43. ^{1}H NMR spectrum (400.13 MHz, $CDCl_3$) of **1S**.

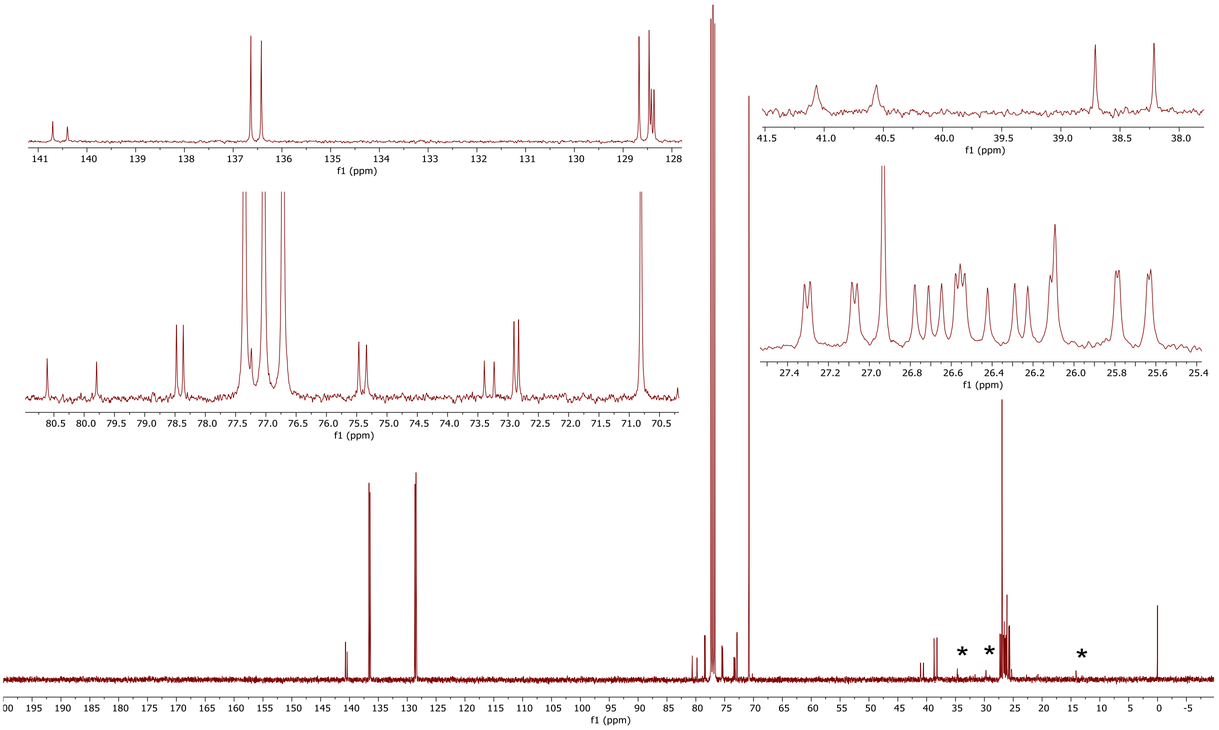

Figure S44. $^{13}C\{^{1}H\}$ NMR spectrum (100.61 MHz, $CDCl_3$) of **1S**.

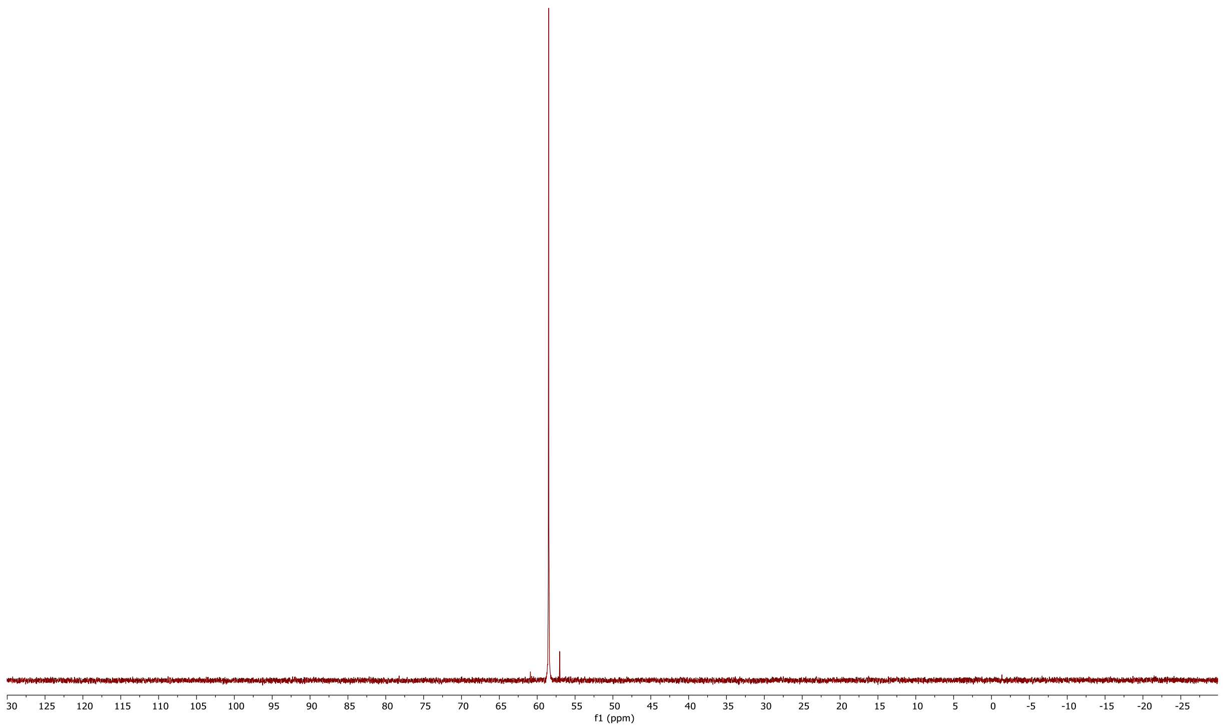

Figure S45. $^{31}P\{^{1}H\}$ NMR spectrum (161.98 MHz, $CDCl_3$) of **1S**.

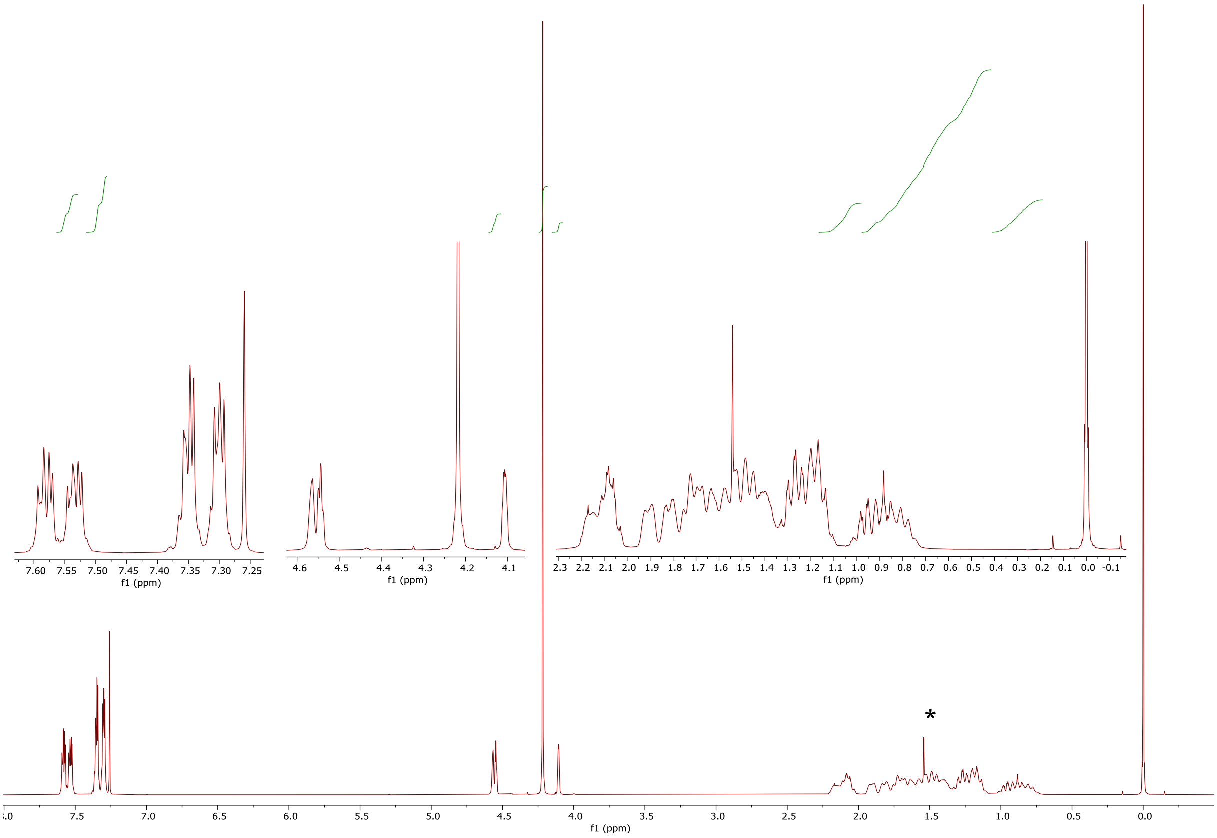

Figure S46. ^{1}H NMR spectrum (400.13 MHz, $CDCl_3$) of **1Se**.

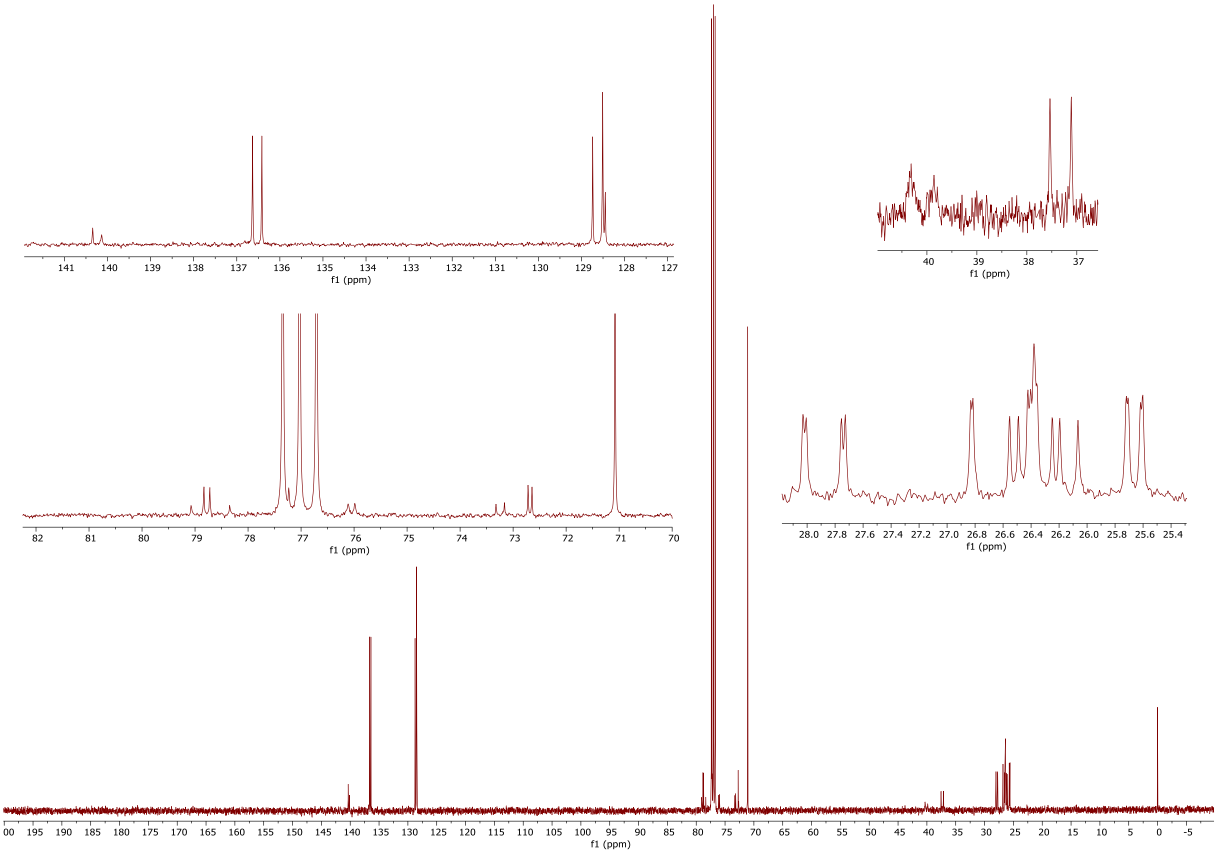

Figure S47. $^{13}C\{^{1}H\}$ NMR spectrum (100.61 MHz, $CDCl_3$) of **1Se**.

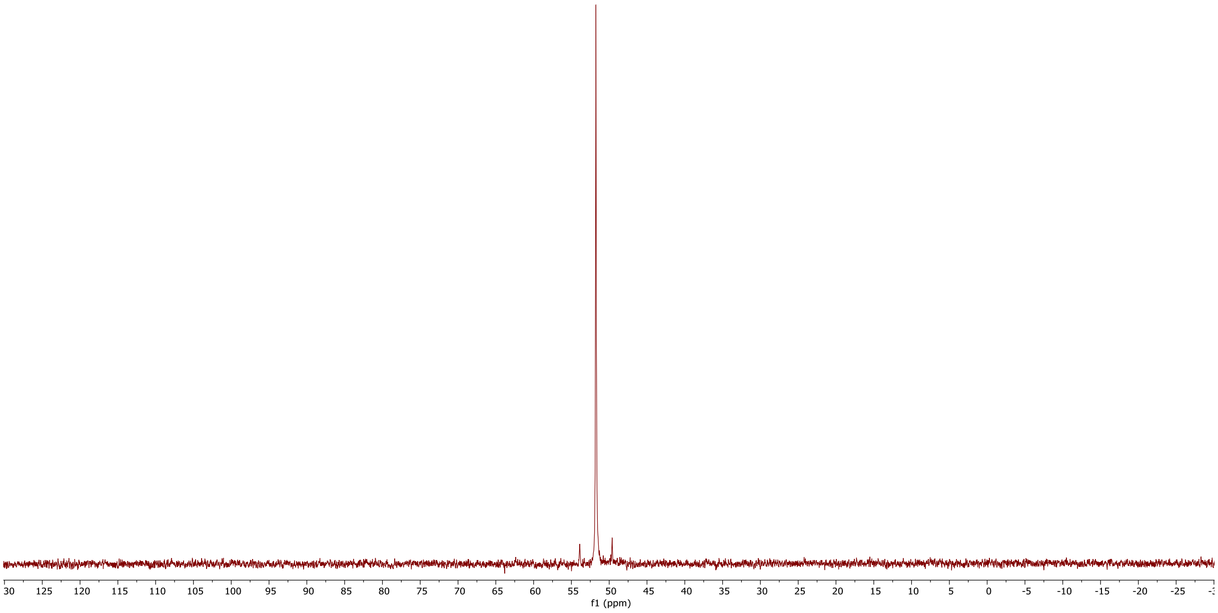

Figure S48. $^{31}P\{^{1}H\}$ NMR spectrum (161.98 MHz, $CDCl_3$) of **1Se**.

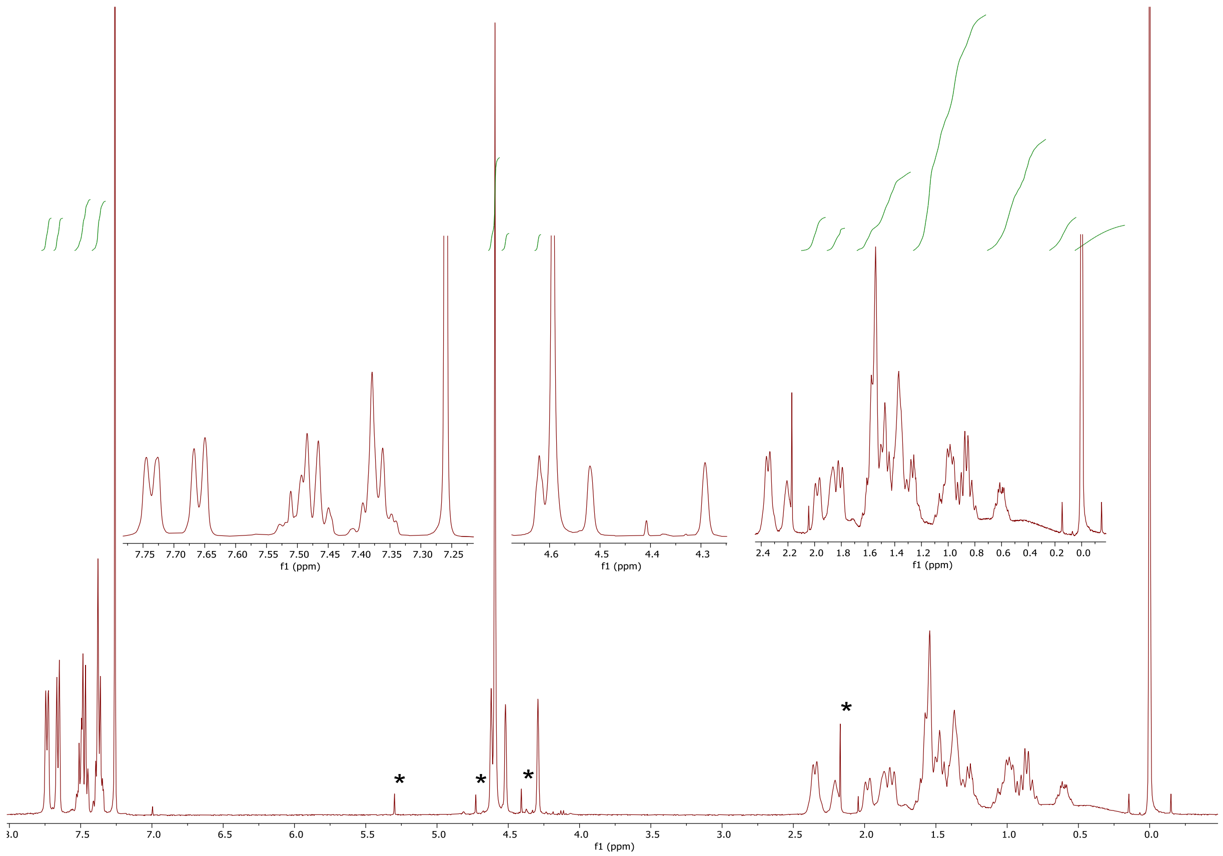

Figure S49. ^{1}H NMR spectrum (400.13 MHz, $CDCl_3$) of **3**·BH_3.

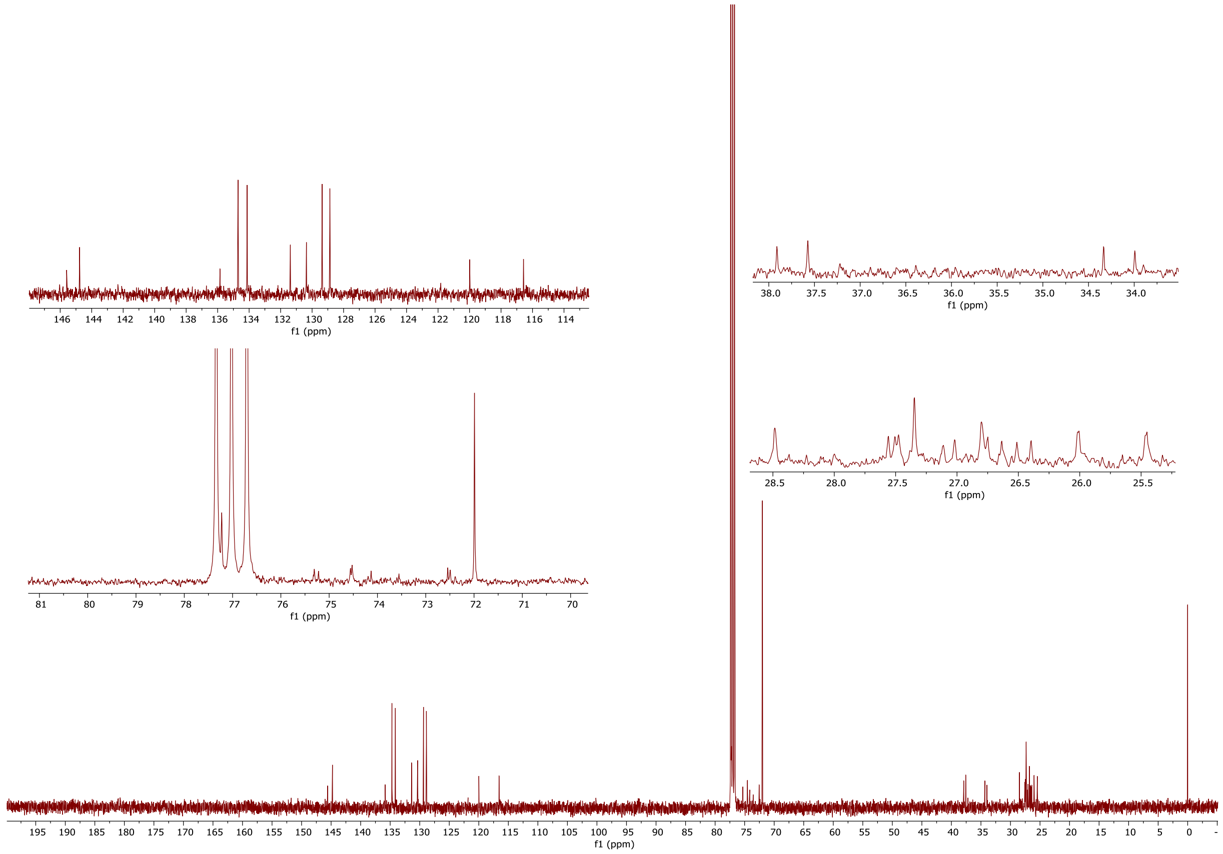

Figure S50. $^{13}C\{^{1}H\}$ NMR spectrum (100.61 MHz, $CDCl_3$) **3**·BH_3.

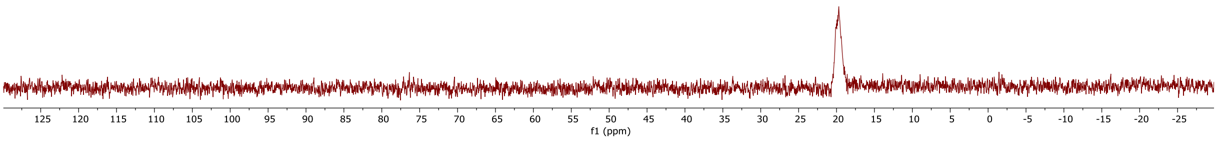

Figure S51. $^{31}P\{^{1}H\}$ NMR spectrum (161.98 MHz, $CDCl_3$) **3**·BH_3.

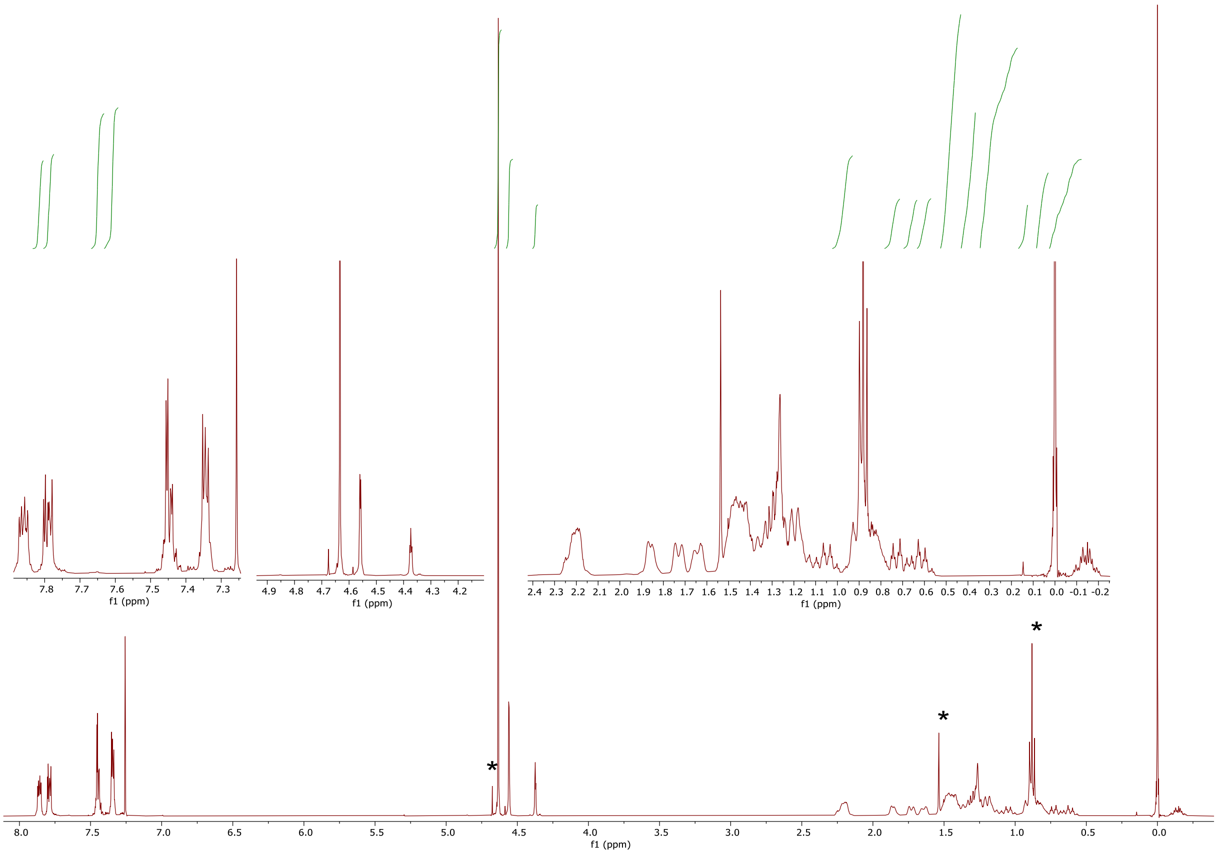

Figure S52. ^{1}H NMR spectrum (400.13 MHz, $CDCl_3$) of **3**.

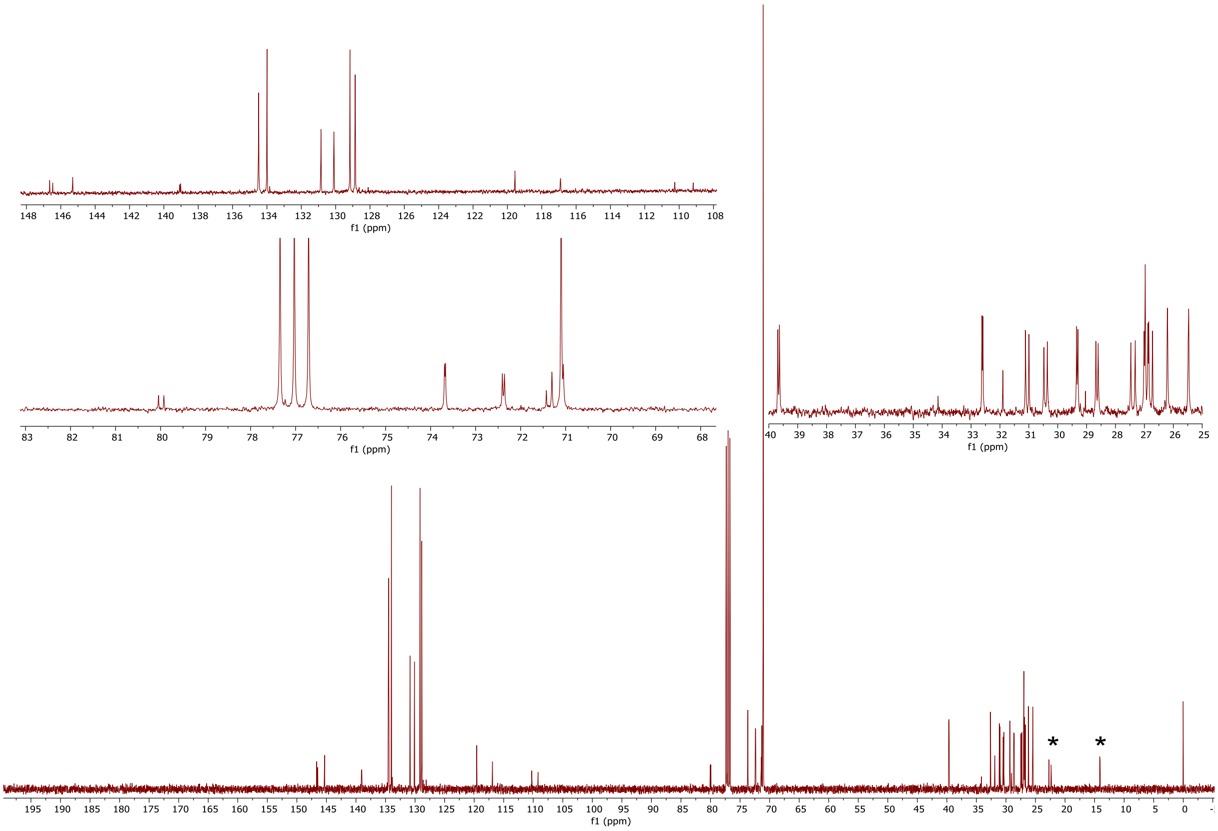

Figure S53. $^{13}C\{^{1}H\}$ NMR spectrum (100.61 MHz, $CDCl_3$) of **3**.

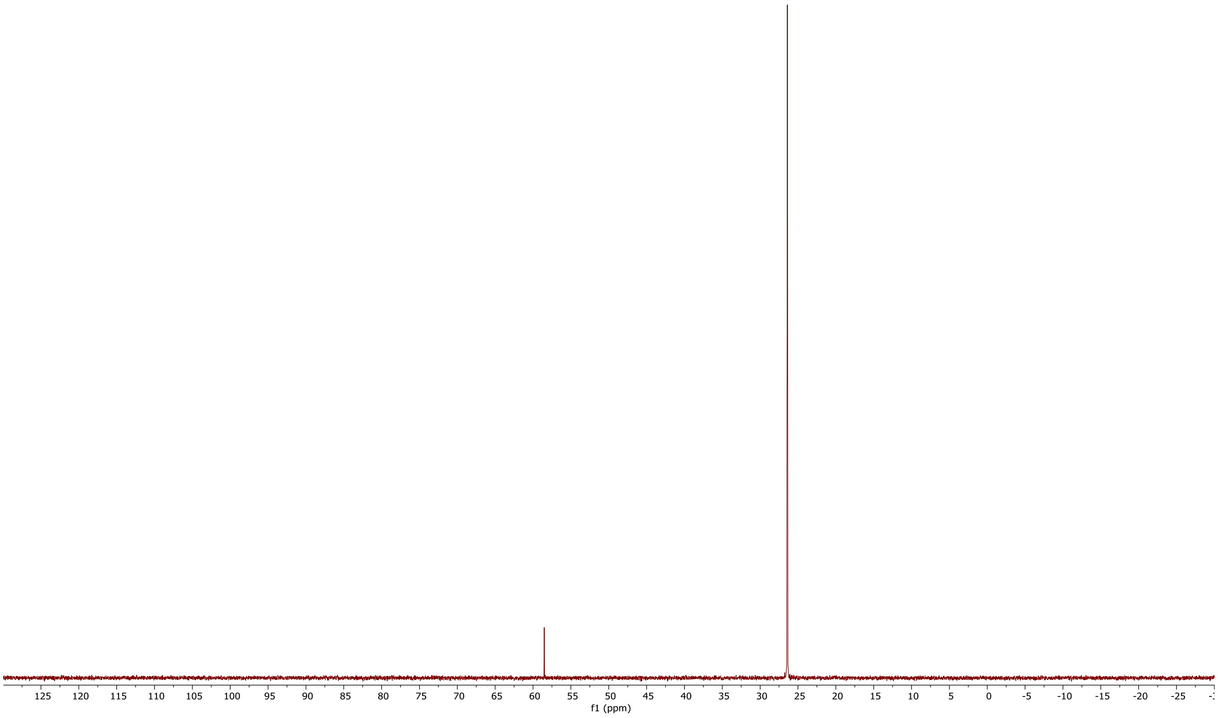

Figure S54. $^{31}P\{^{1}H\}$ NMR spectrum (161.98 MHz, $CDCl_3$) of **3**.

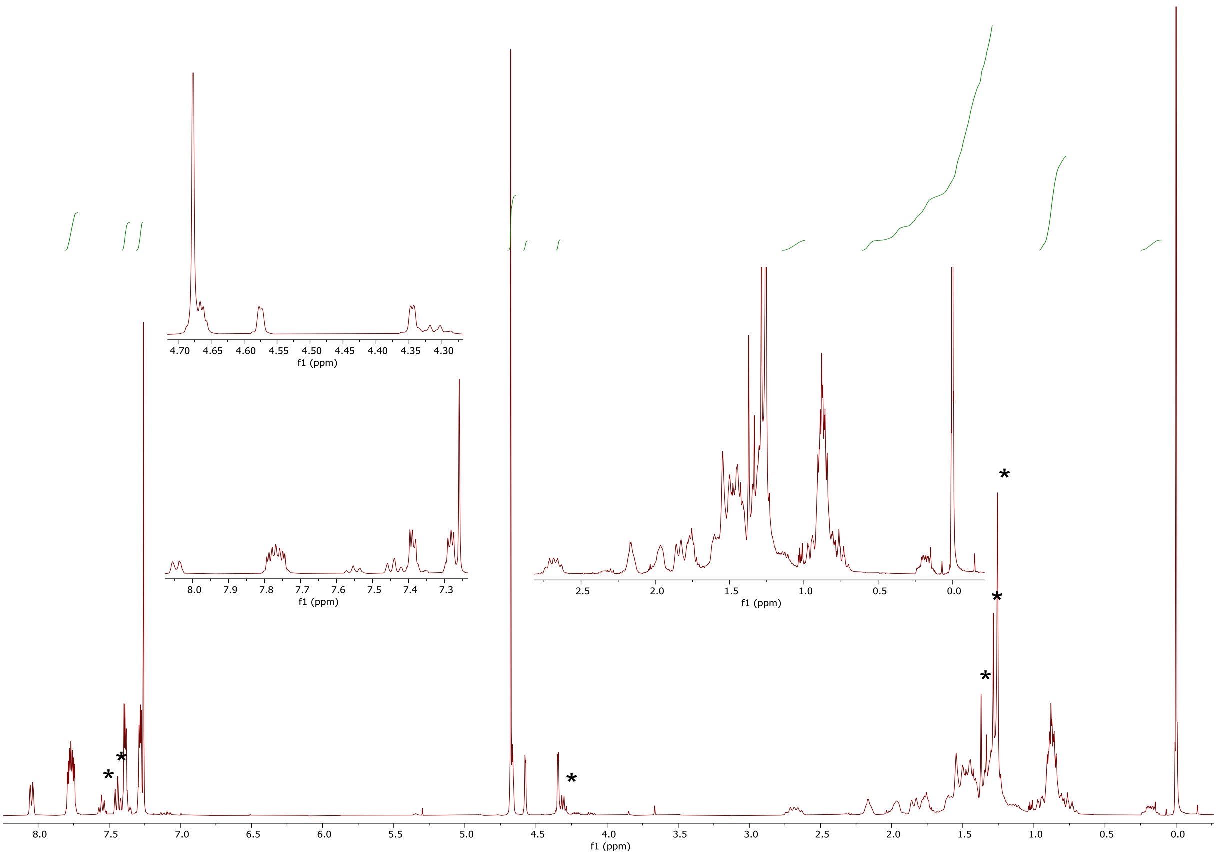

Figure S55. ^{1}H NMR spectrum (400.13 MHz, $CDCl_3$) of **3O**.

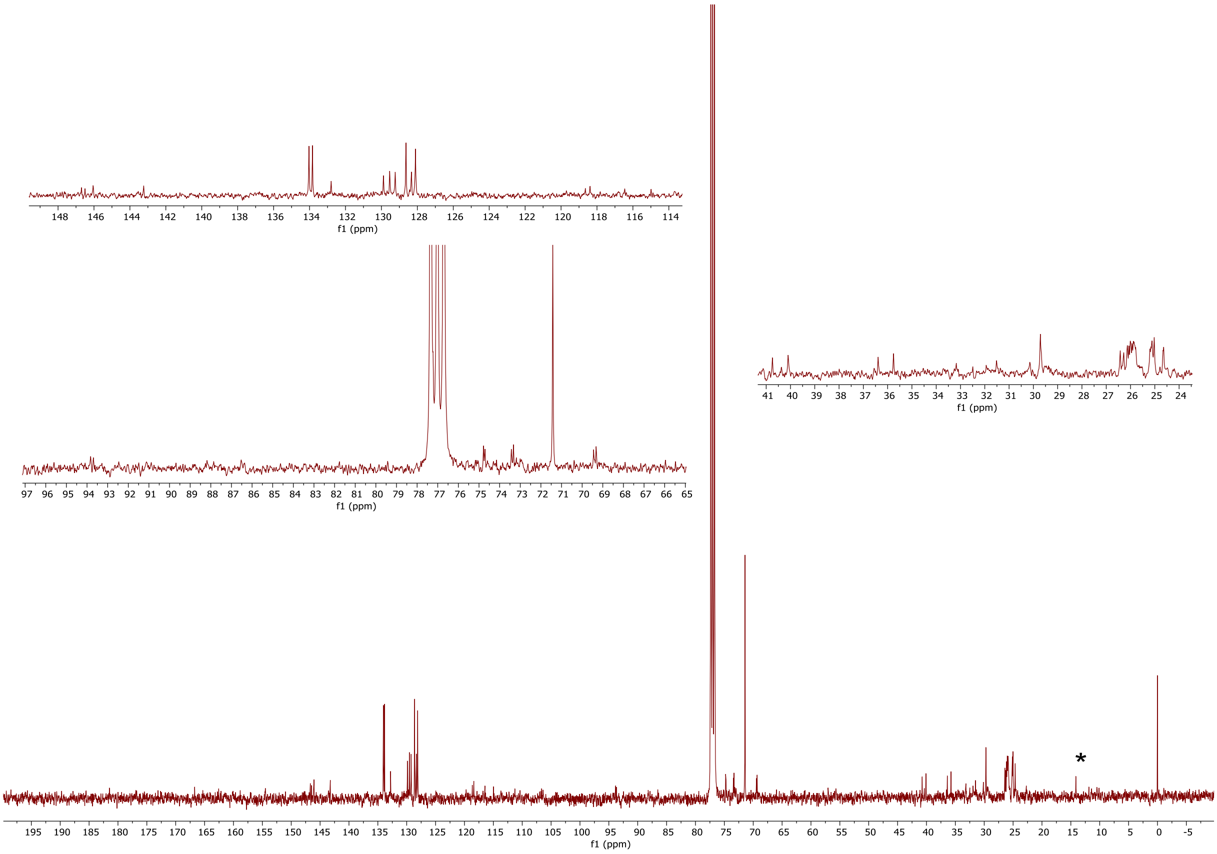

Figure S56. $^{13}C\{^{1}H\}$ NMR spectrum (100.61 MHz, $CDCl_3$) of **3O**.

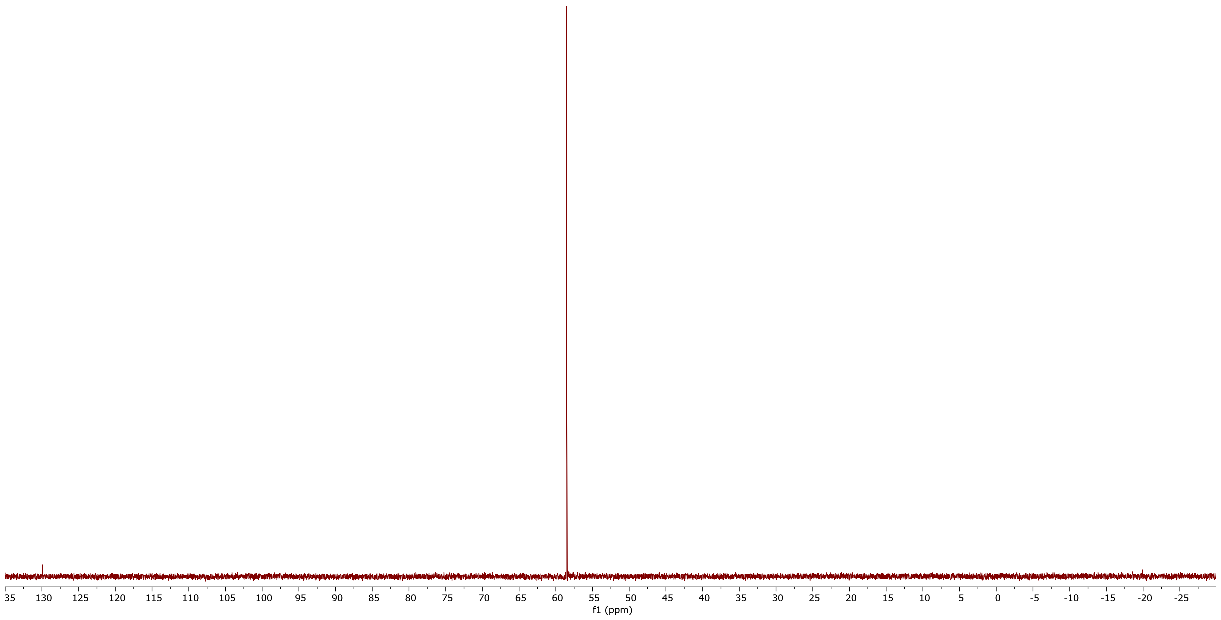

Figure S57. $^{31}P\{^{1}H\}$ NMR spectrum (161.98 MHz, $CDCl_3$) of **3O**.

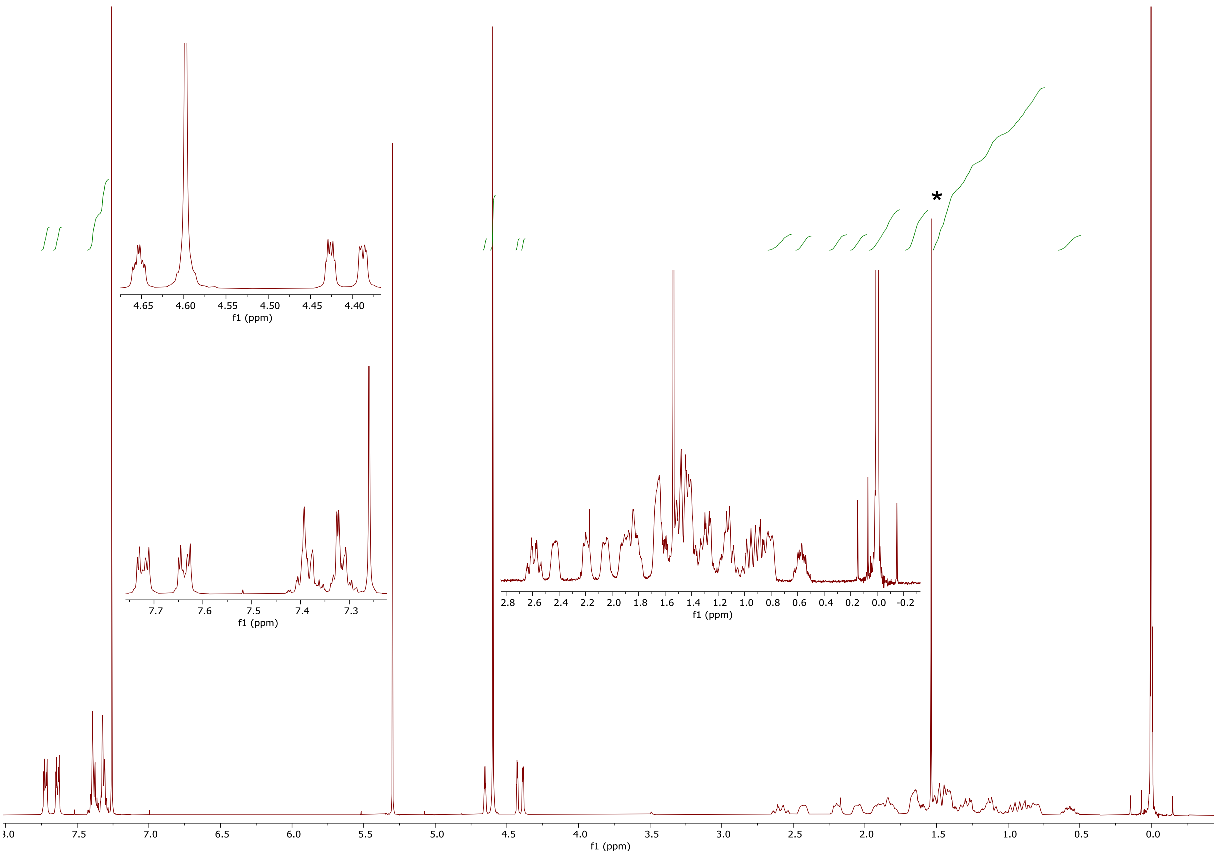

Figure S58. ^{1}H NMR spectrum (400.13 MHz, $CDCl_3$) of **3S**.

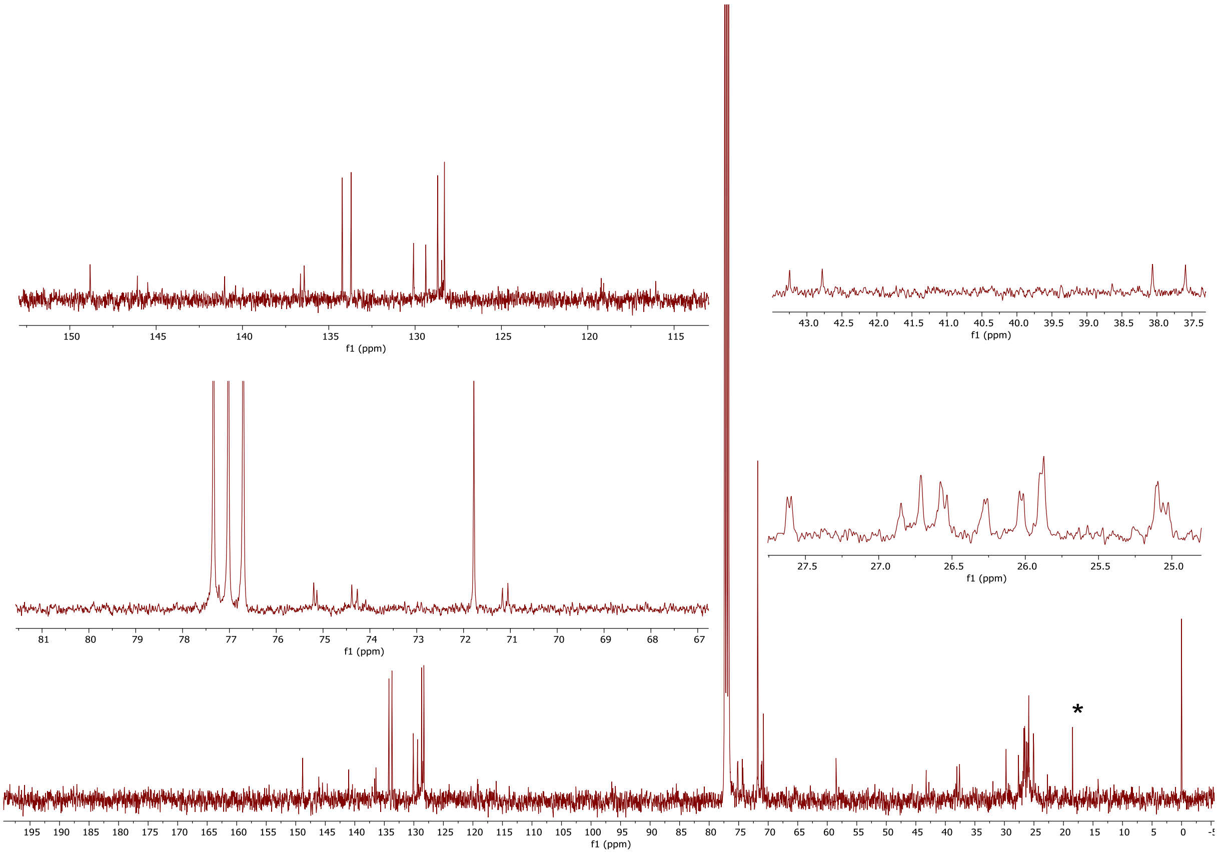

Figure S59. $^{13}C\{^{1}H\}$ NMR spectrum (100.61 MHz, $CDCl_3$) of **3S**.

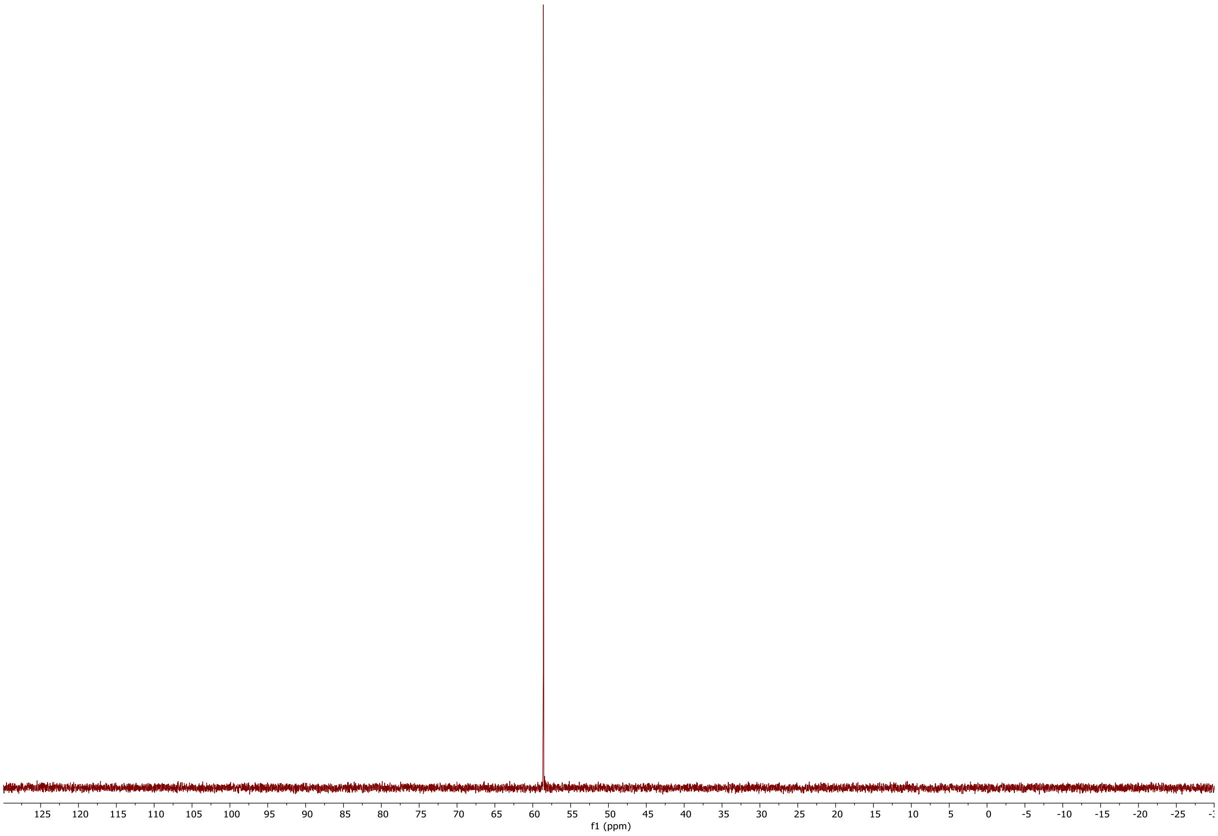

Figure S60. $^{31}P\{^{1}H\}$ NMR spectrum (161.98 MHz, $CDCl_3$) of **3S**.

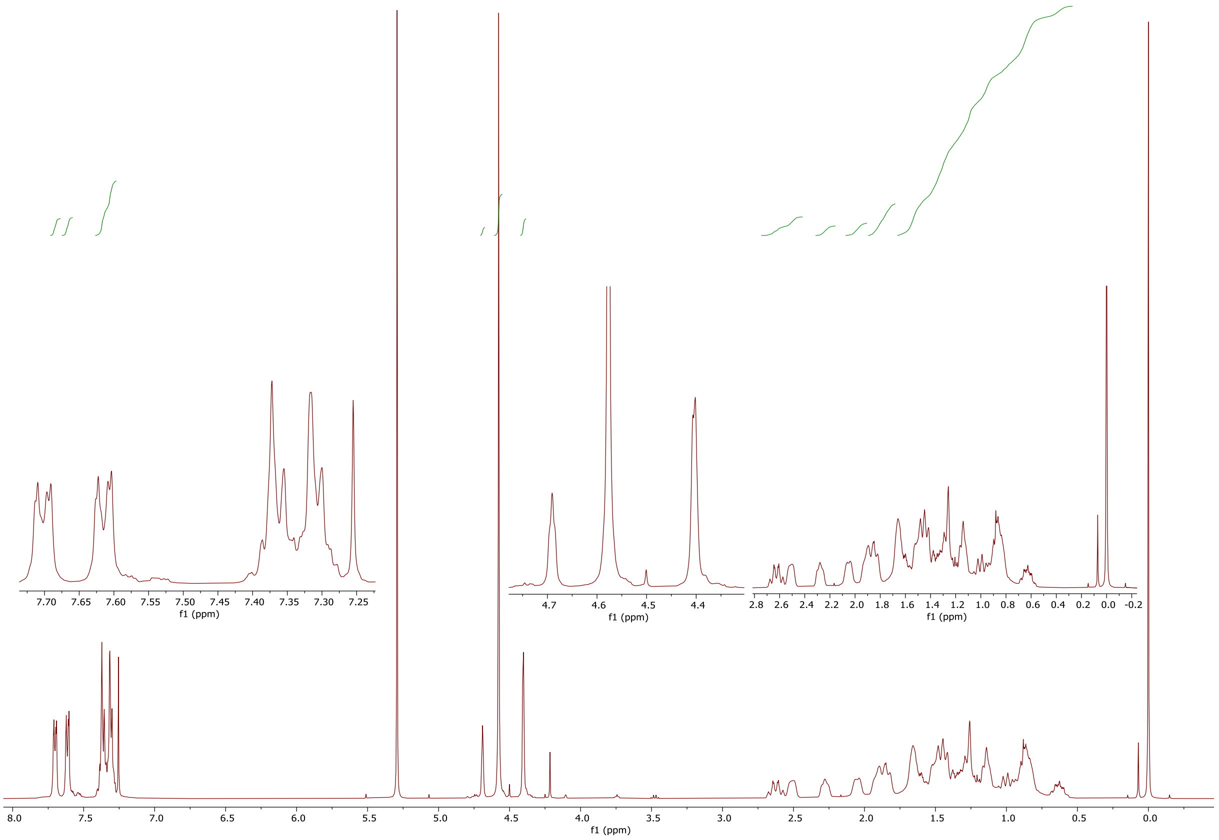

Figure S61. ^{1}H NMR spectrum (400.13 MHz, $CDCl_3$) of **3Se**.

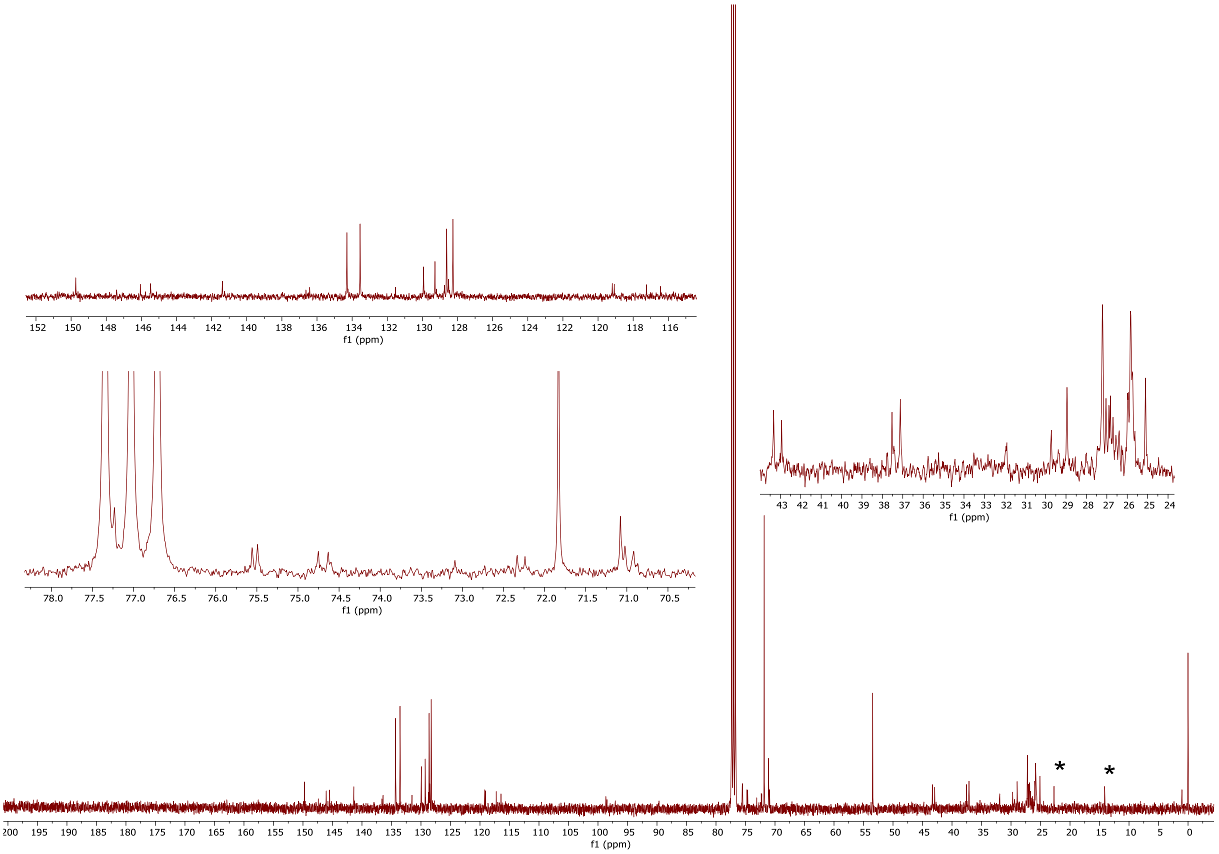

Figure S62. $^{13}C\{^{1}H\}$ NMR spectrum (100.61 MHz, $CDCl_3$) of **3Se**.

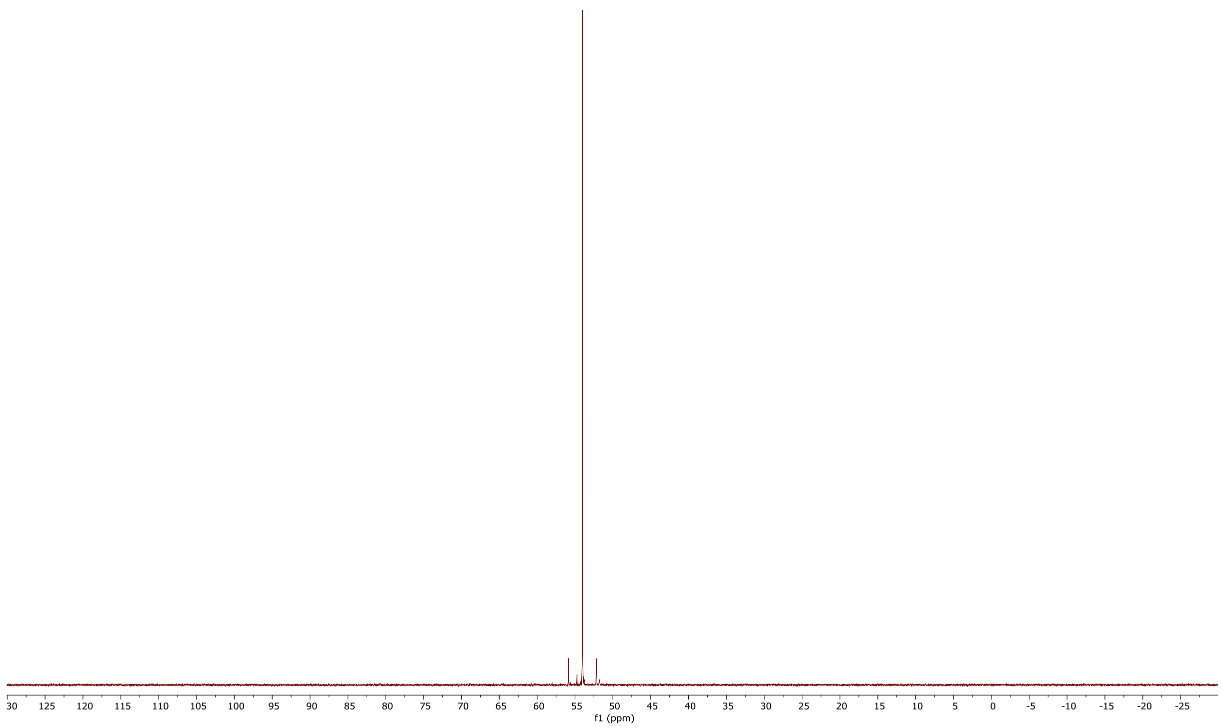

Figure S63. $^{31}P\{^{1}H\}$ NMR spectrum (161.98 MHz, $CDCl_3$) of **3Se**.

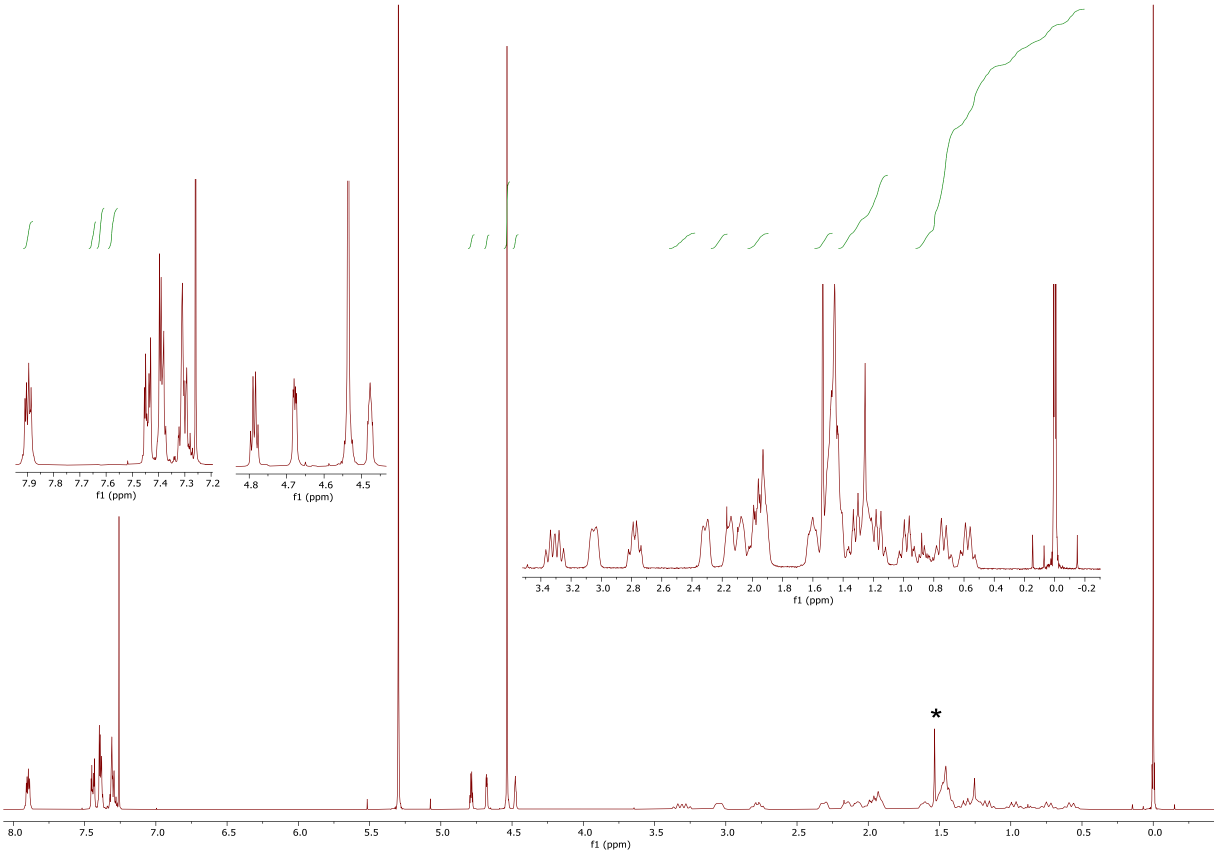

Figure S64. ^{1}H NMR spectrum (400.13 MHz, $CDCl_3$) of **4**.

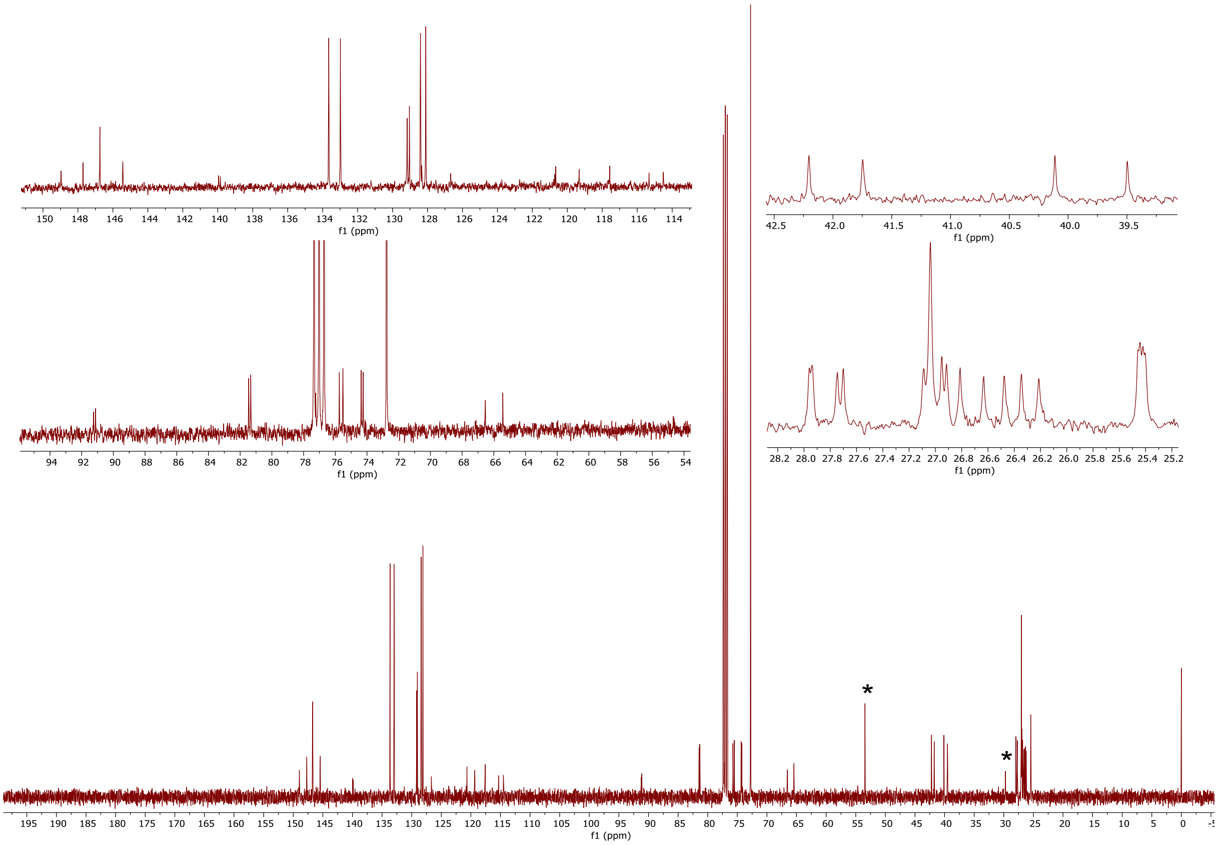

Figure S65. $^{13}C\{^{1}H\}$ NMR spectrum (100.61 MHz, $CDCl_3$) of **4**.

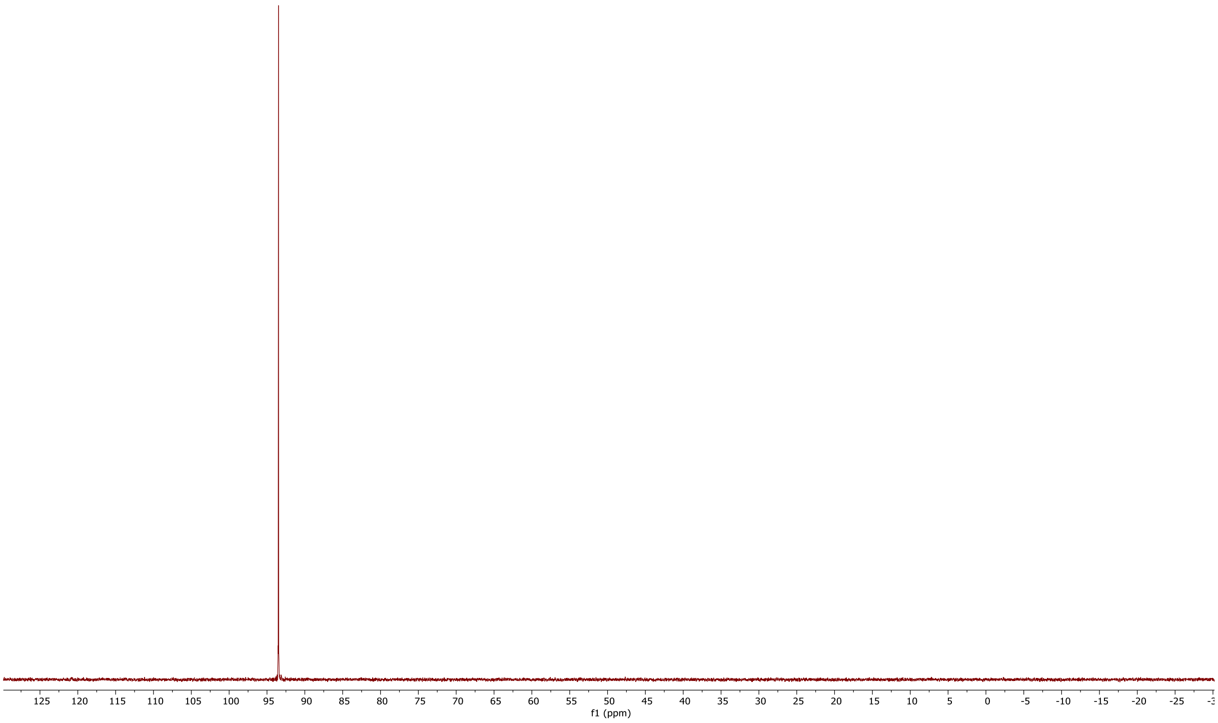

Figure S66. $^{31}P\{^{1}H\}$ NMR spectrum (161.98 MHz, $CDCl_3$) of **4**.

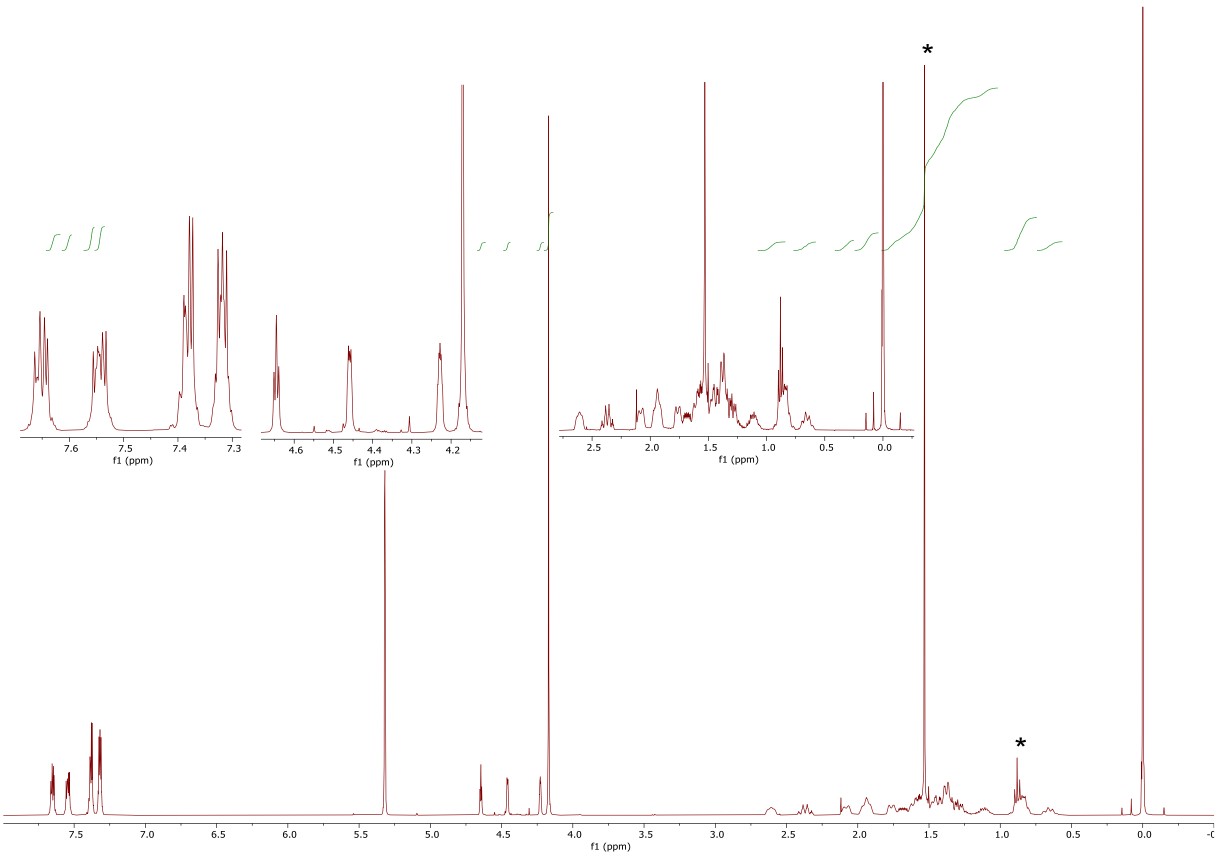

Figure S67. ^{1}H NMR spectrum (400.13 MHz, CD_2Cl_2) of **5**.

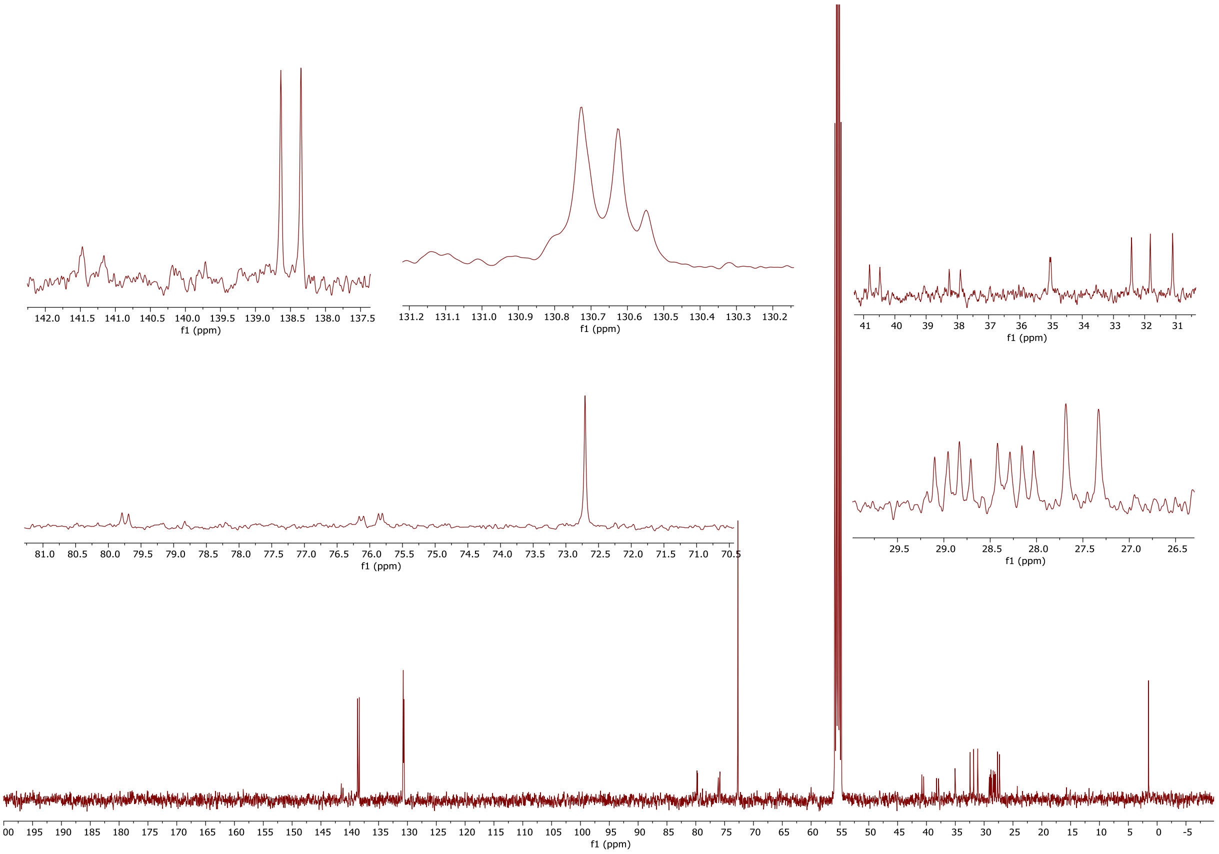

Figure S68. $^{13}C\{^{1}H\}$ NMR spectrum (100.61 MHz, CD_2Cl_2) of **5**.

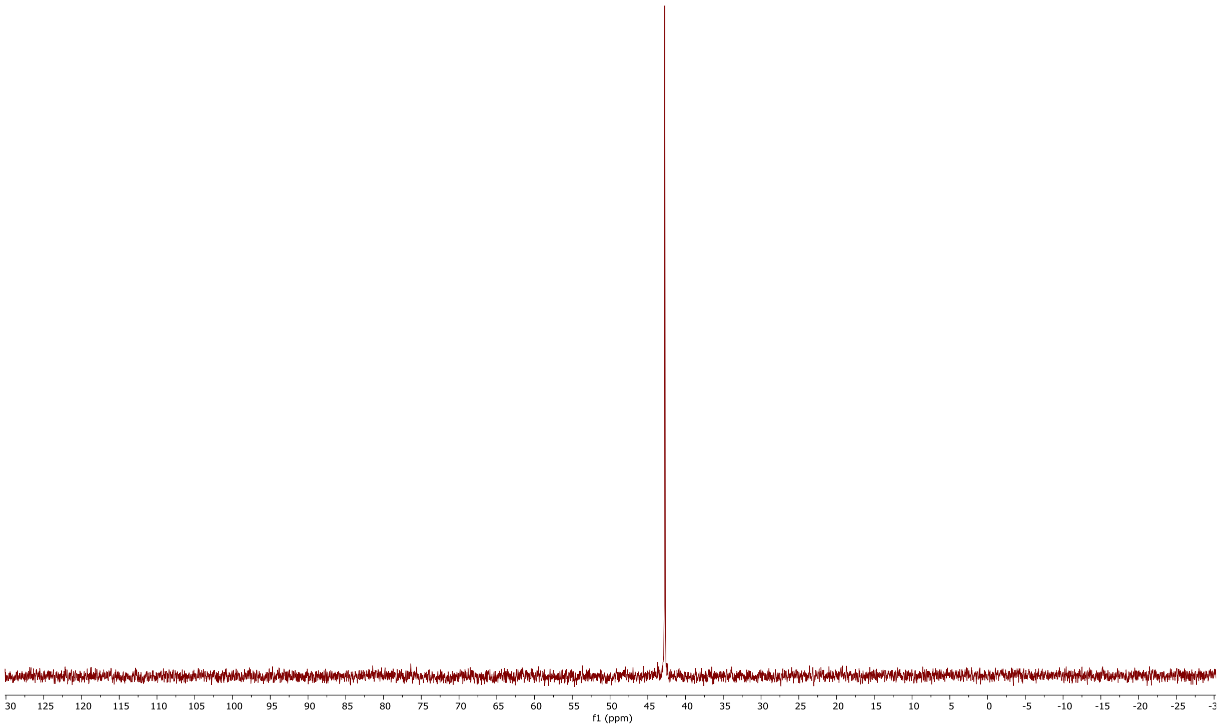

Figure S69. $^{31}P\{^{1}H\}$ NMR spectrum (161.98 MHz, CD_2Cl_2) of **5**.

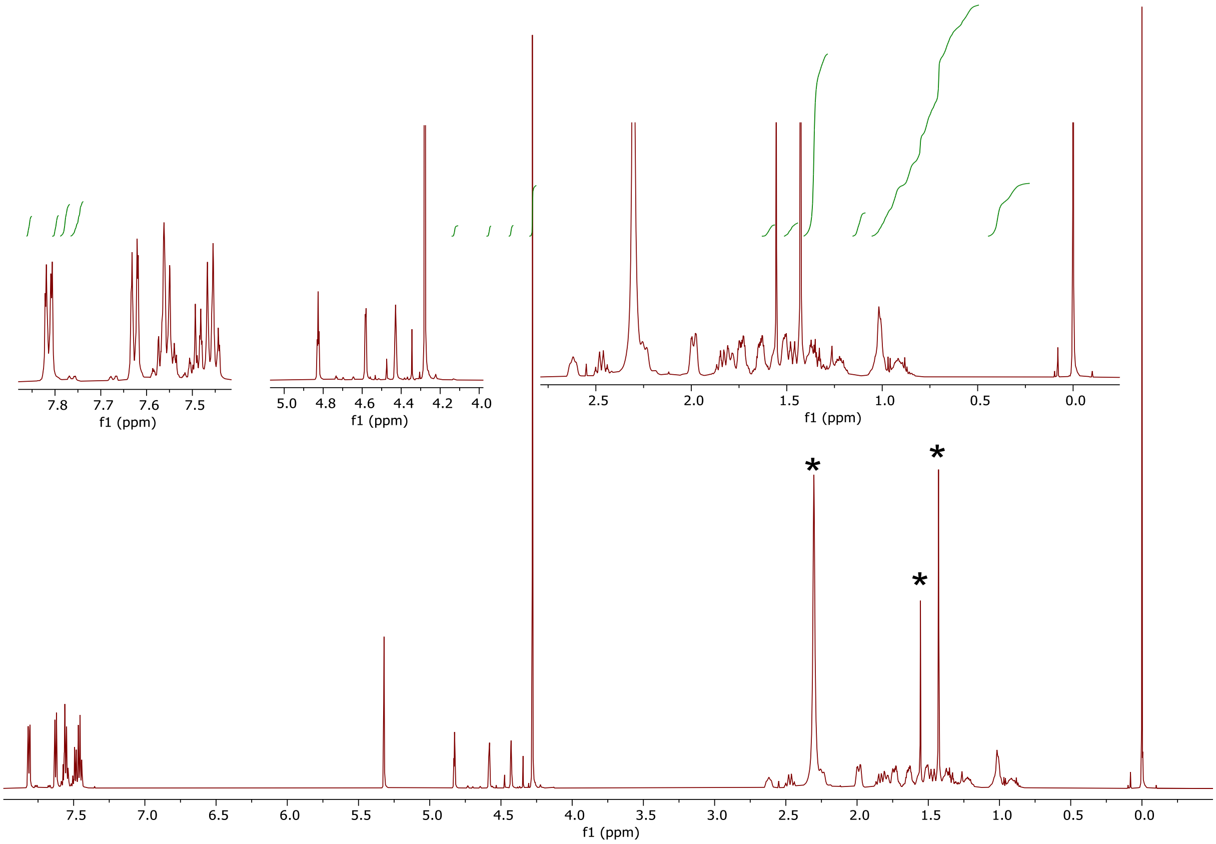

Figure S70. ^{1}H NMR spectrum (600.17 MHz, CD_2Cl_2) of **6**.

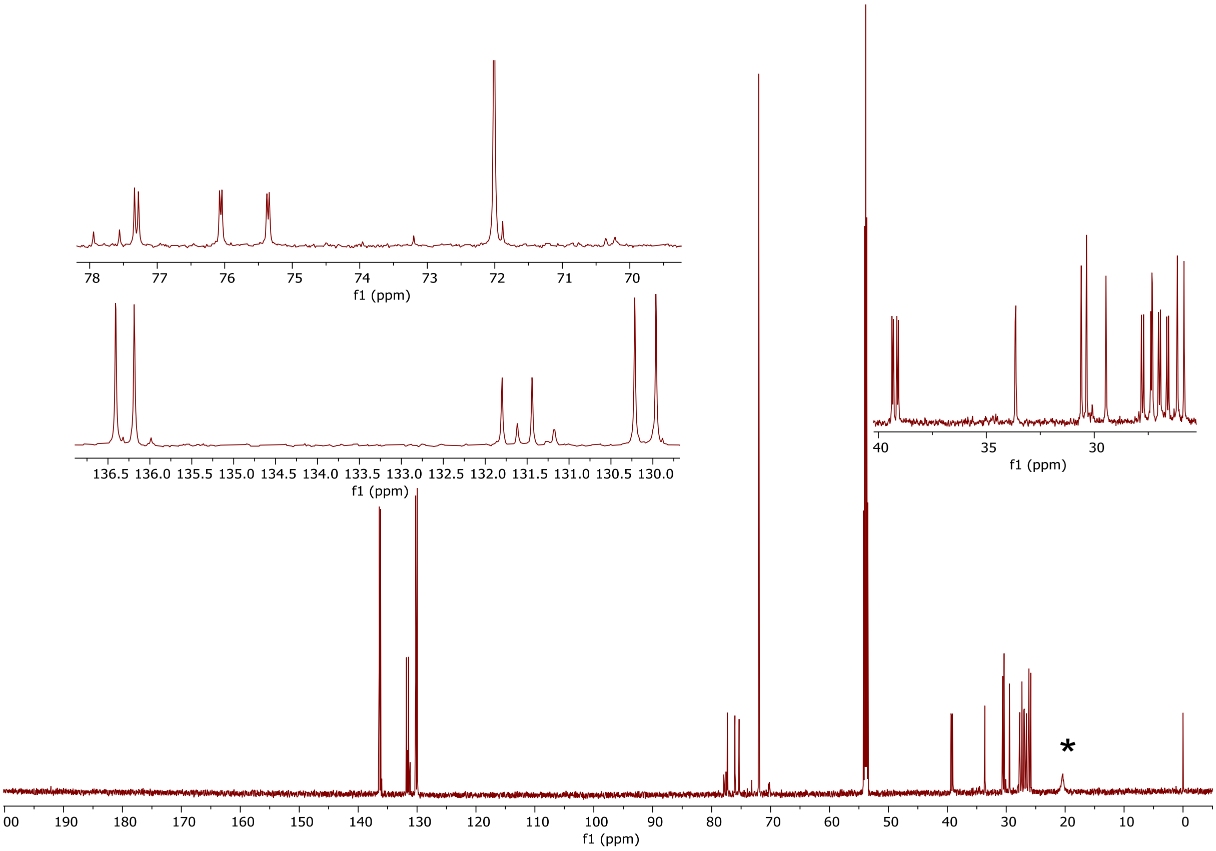

Figure S71. $^{13}C\{^{1}H\}$ NMR spectrum (150.93 MHz, CD_2Cl_2) of **6**.

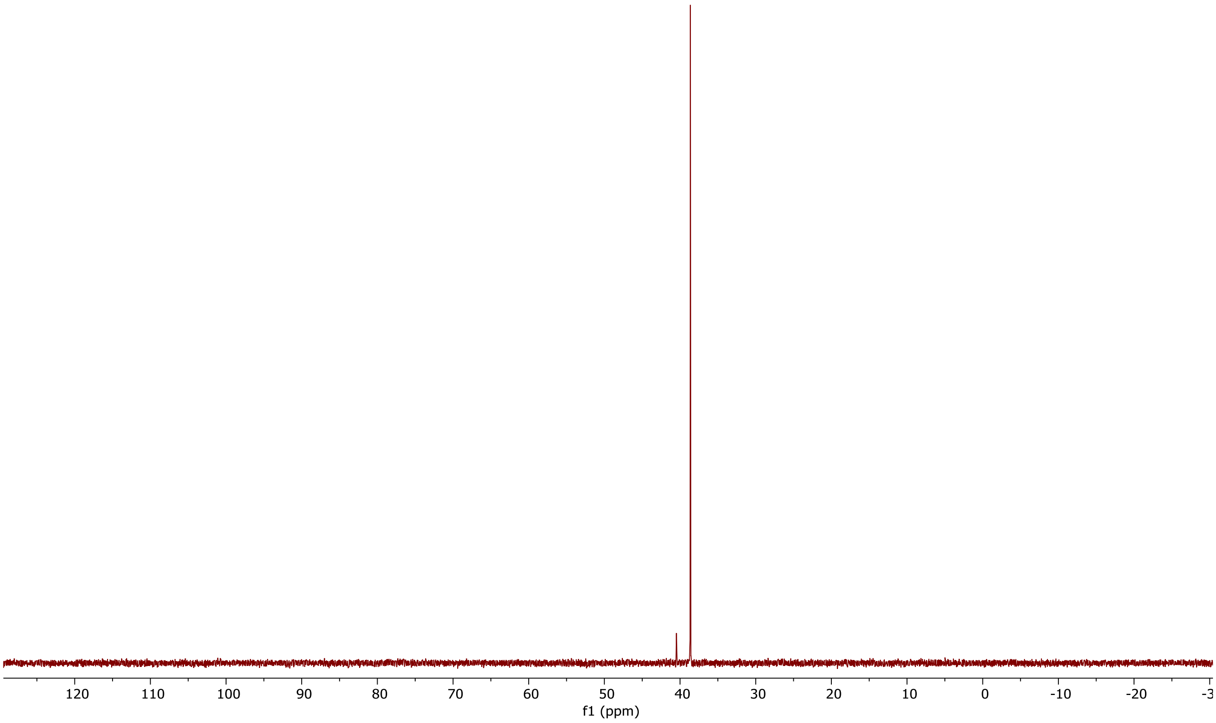

Figure S72. $^{31}P\{^{1}H\}$ NMR spectrum (242.96 MHz, CD_2Cl_2) of **6**.

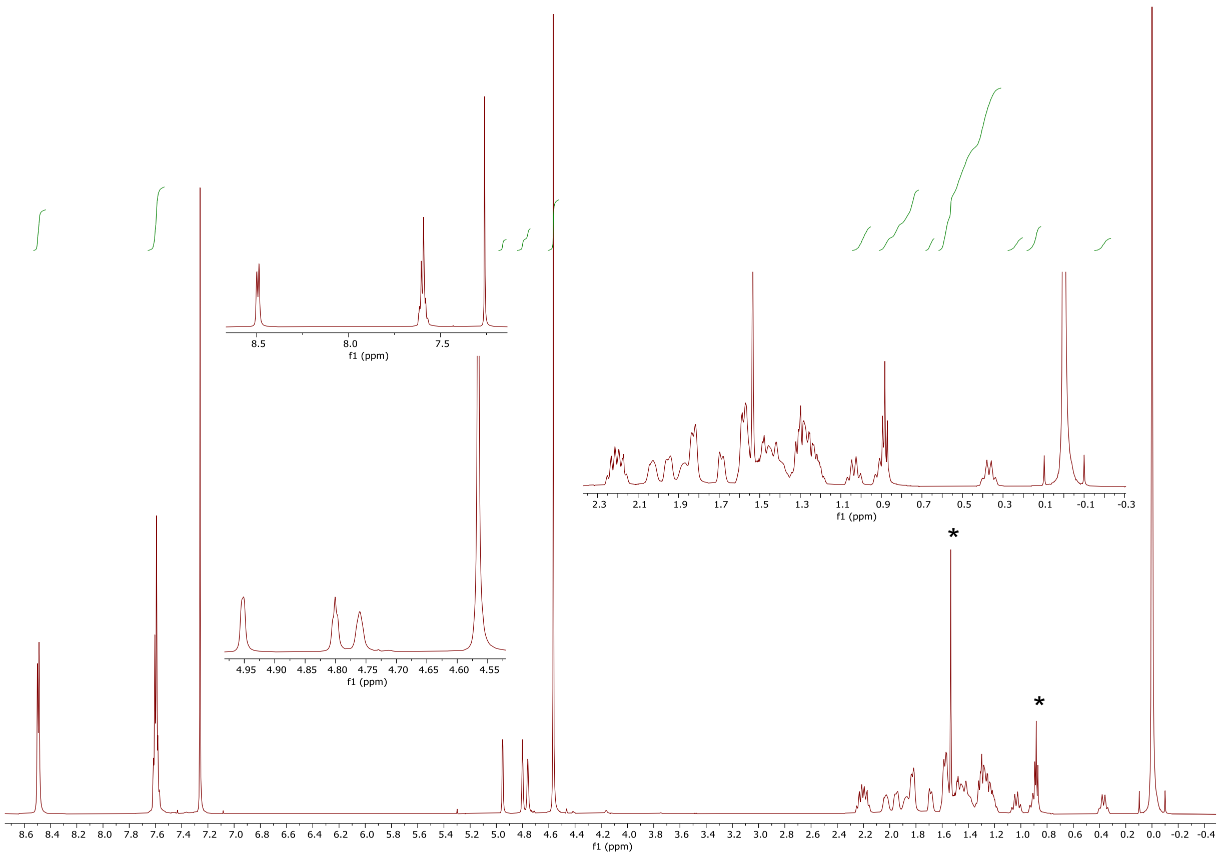

Figure S73. ^{1}H NMR spectrum (400.13 MHz, $CDCl_3$) of **7**.

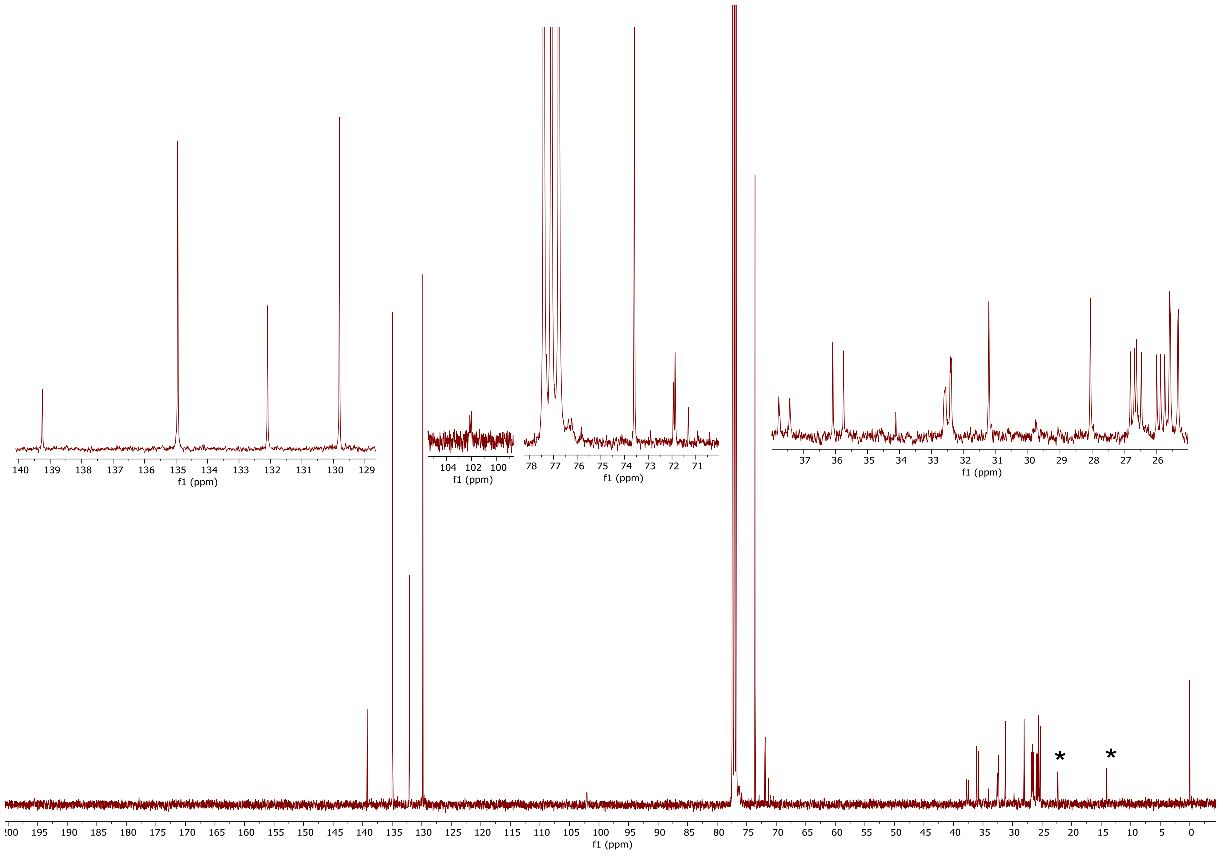

Figure S74. $^{13}C\{^{1}H\}$ NMR spectrum (100.61 MHz, $CDCl_3$) of **7**.

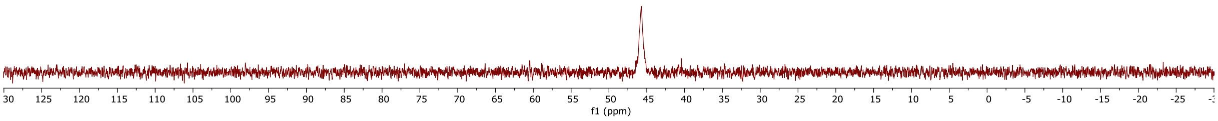

Figure S75. $^{31}P\{^{1}H\}$ NMR spectrum (161.98 MHz, $CDCl_3$) of **7**.

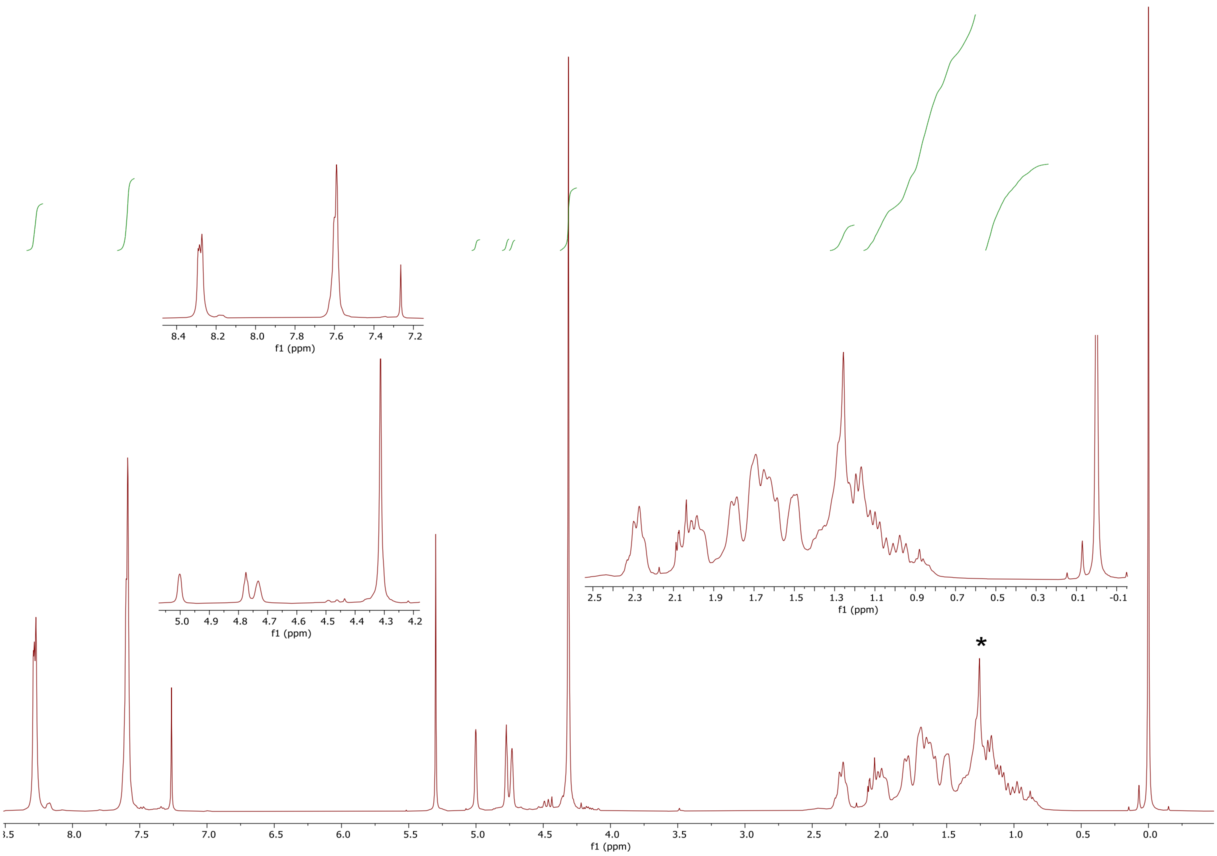

Figure S76. ^{1}H NMR spectrum (400.13 MHz, $CDCl_3$) of **8**.

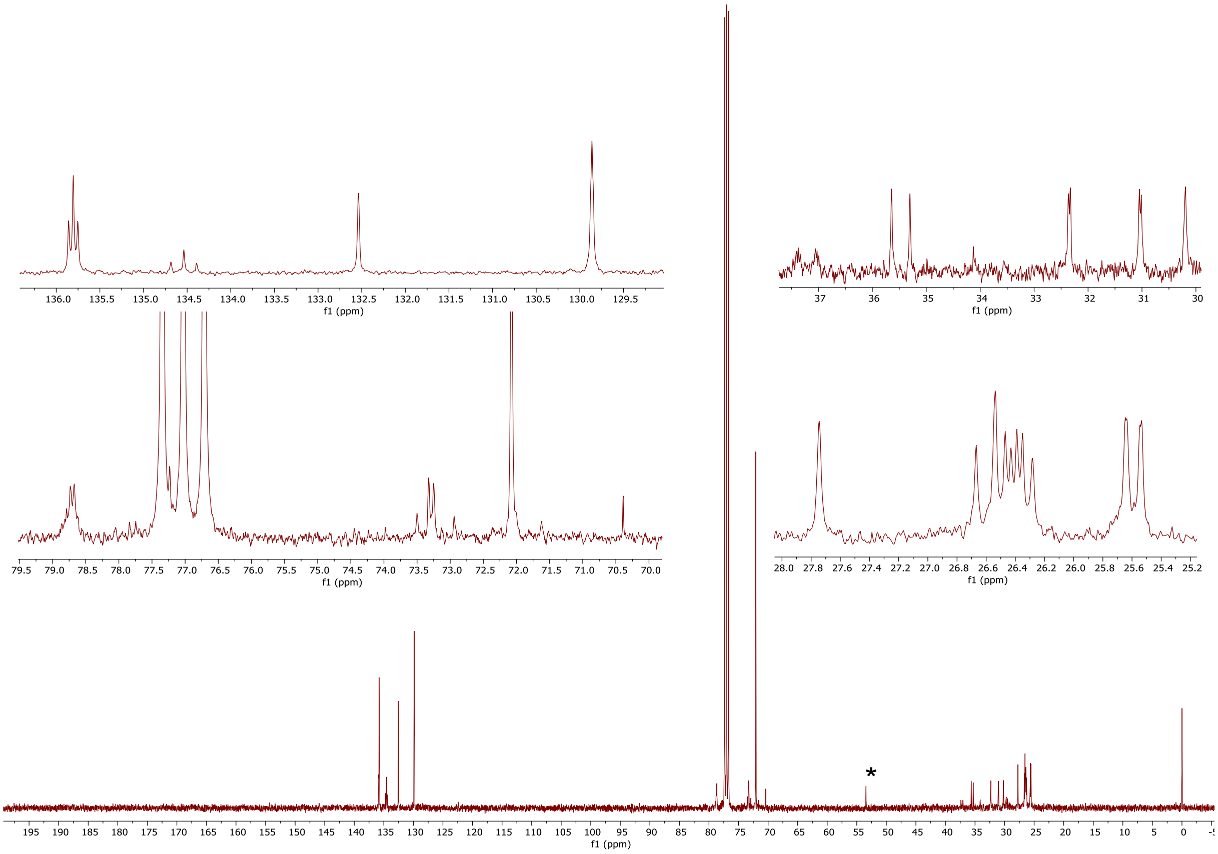

Figure S77. $^{13}C\{^{1}H\}$ NMR spectrum (100.61 MHz, $CDCl_3$) of **8**.

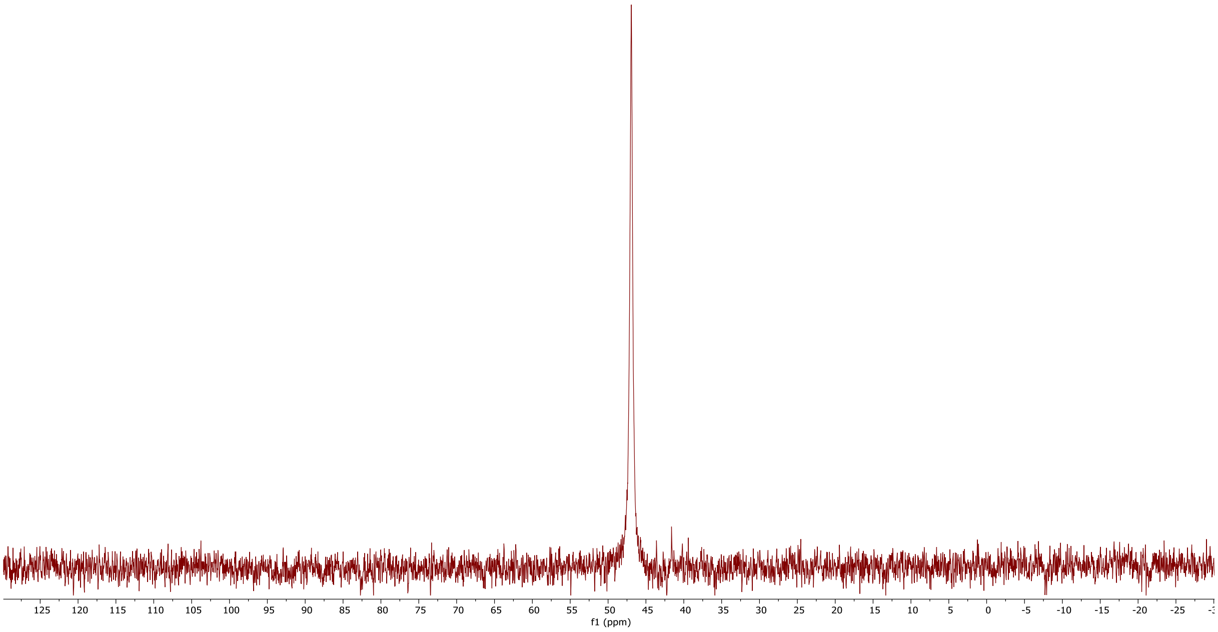

Figure S78. $^{31}P\{^{1}H\}$ NMR spectrum (161.98 MHz, $CDCl_3$) of **8**.

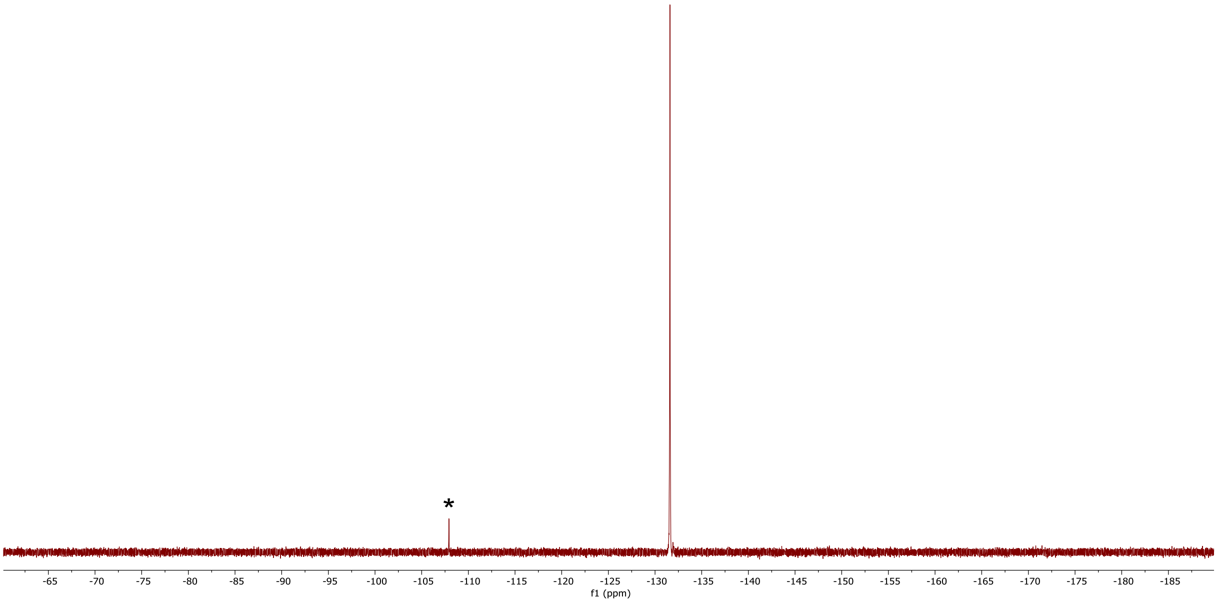

Figure S79. ^{19}F NMR spectrum (376.50 MHz, $CDCl_3$) of **8**.

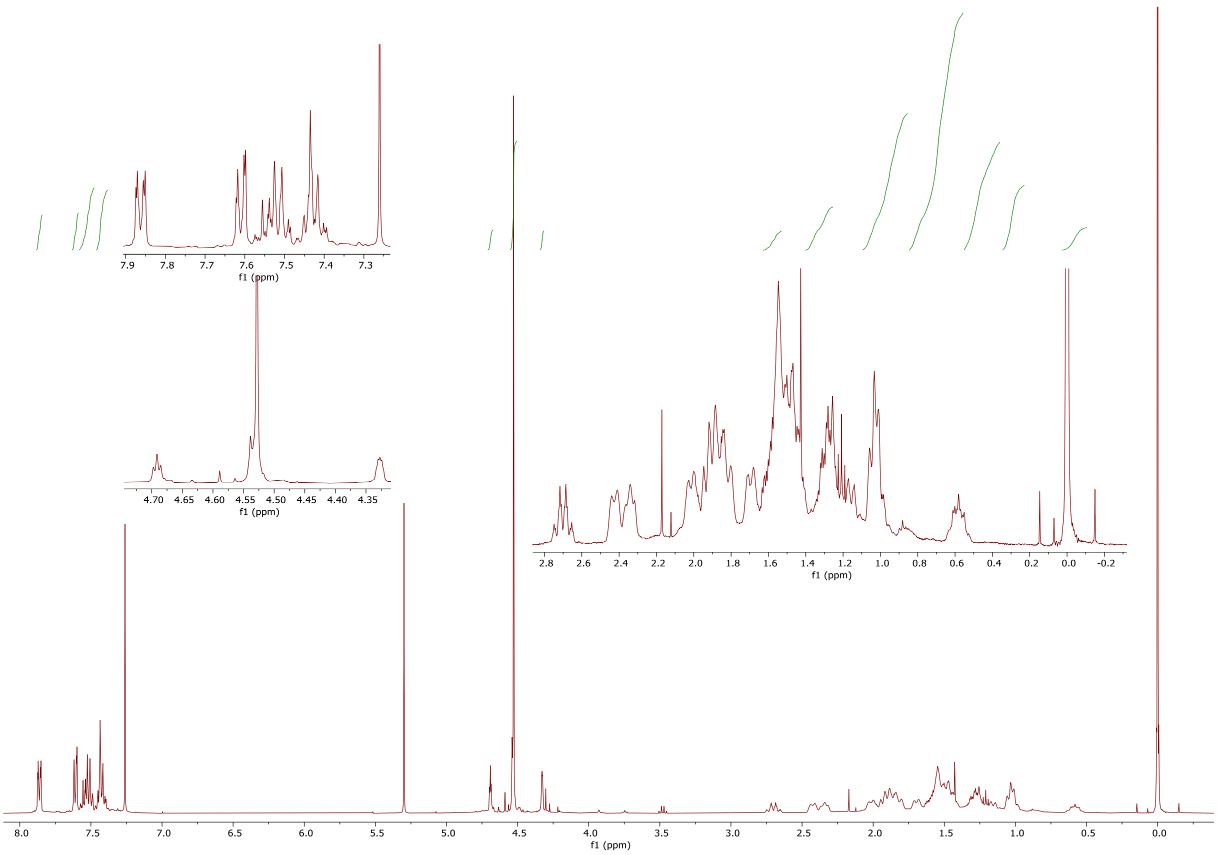

Figure S80. ^{1}H NMR spectrum (400.13 MHz, $CDCl_3$) of **9**.

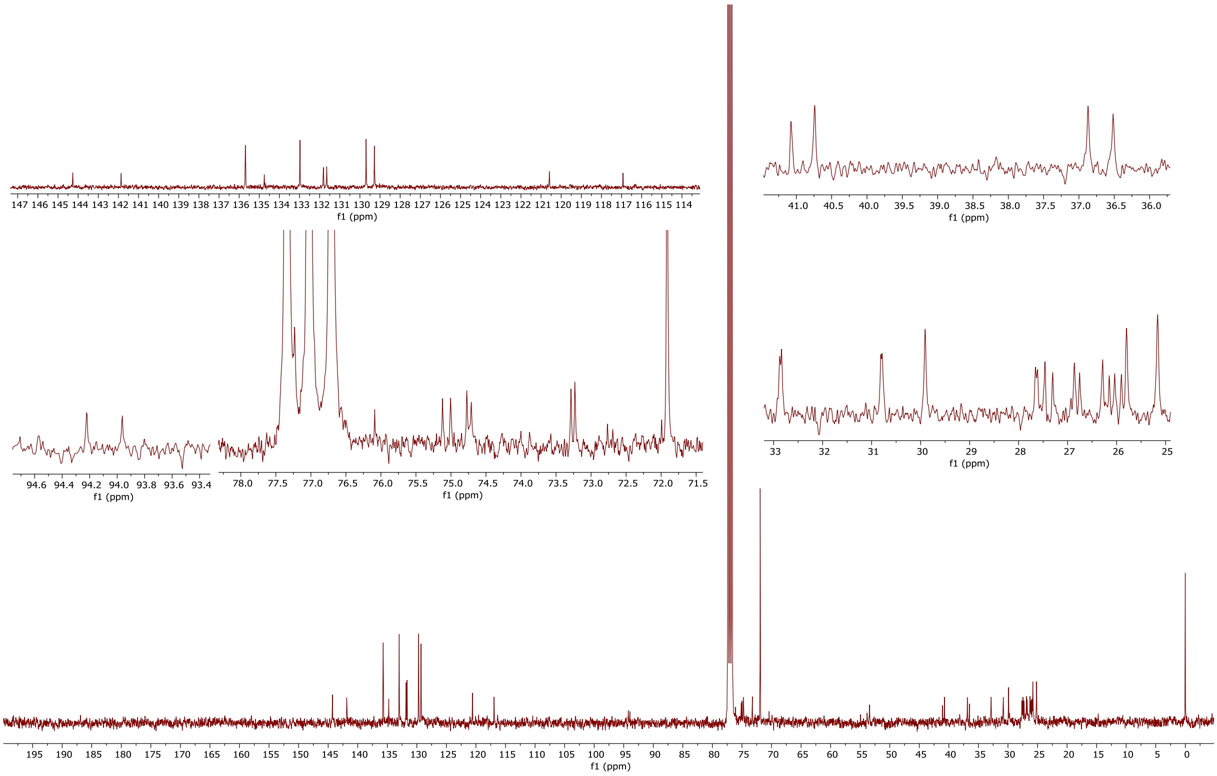

Figure S81. $^{13}C\{^{1}H\}$ NMR spectrum (100.61 MHz, $CDCl_3$) of **9**.

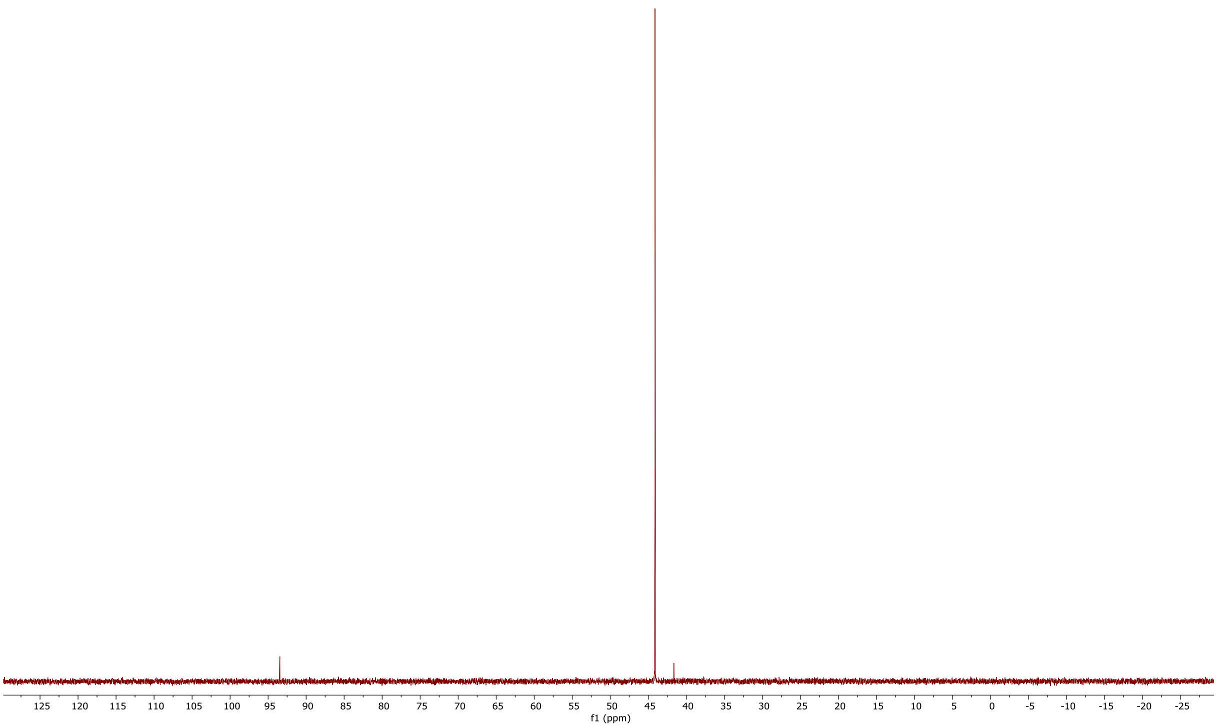

Figure S82. $^{31}P\{^{1}H\}$ NMR spectrum (161.98 MHz, $CDCl_3$) of **9**.

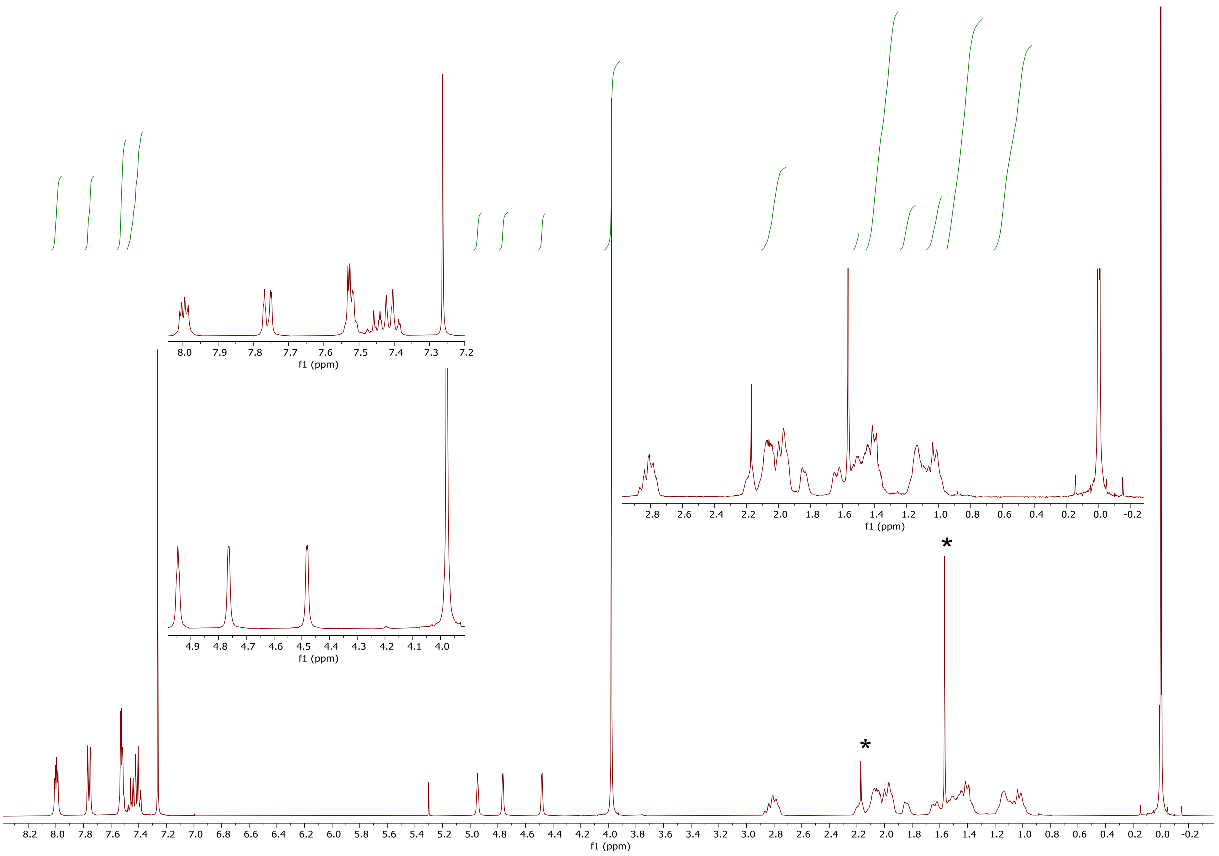

Figure S83. ^{1}H NMR spectrum (400.13 MHz, $CDCl_3$) of **12**.

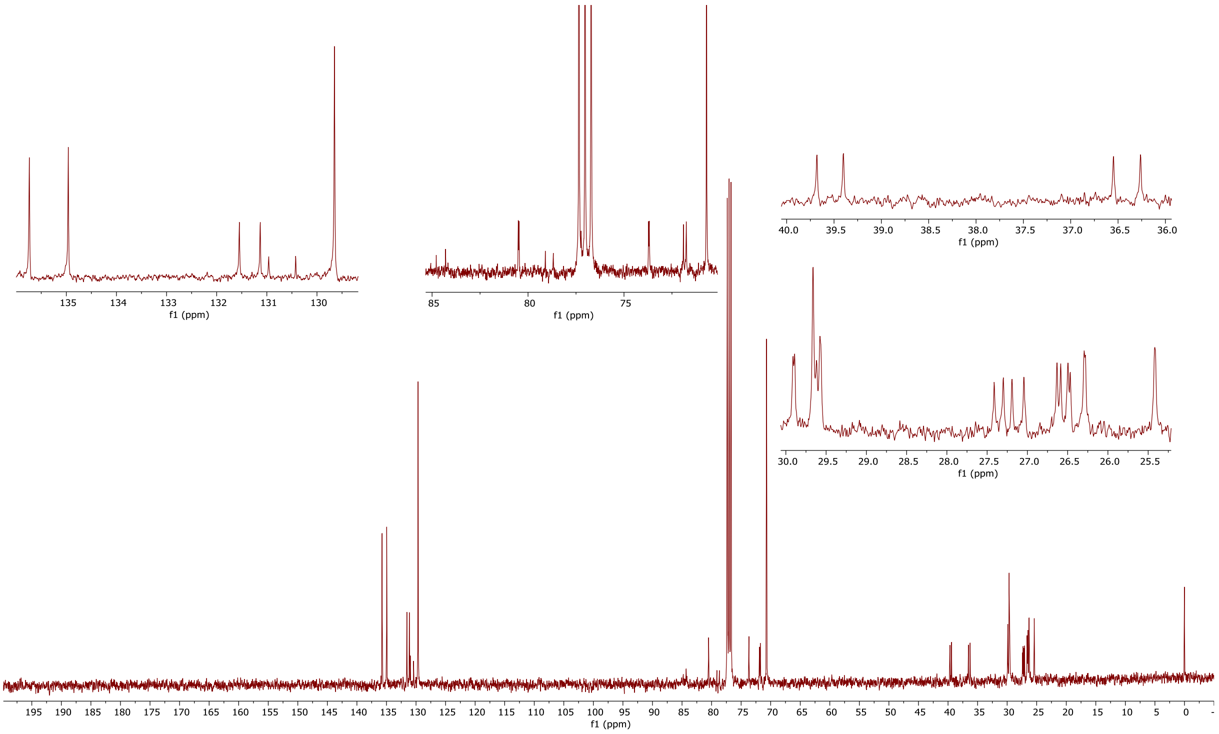

Figure S84. $^{13}C\{^{1}H\}$ NMR spectrum (100.61 MHz, $CDCl_3$) of **12**.

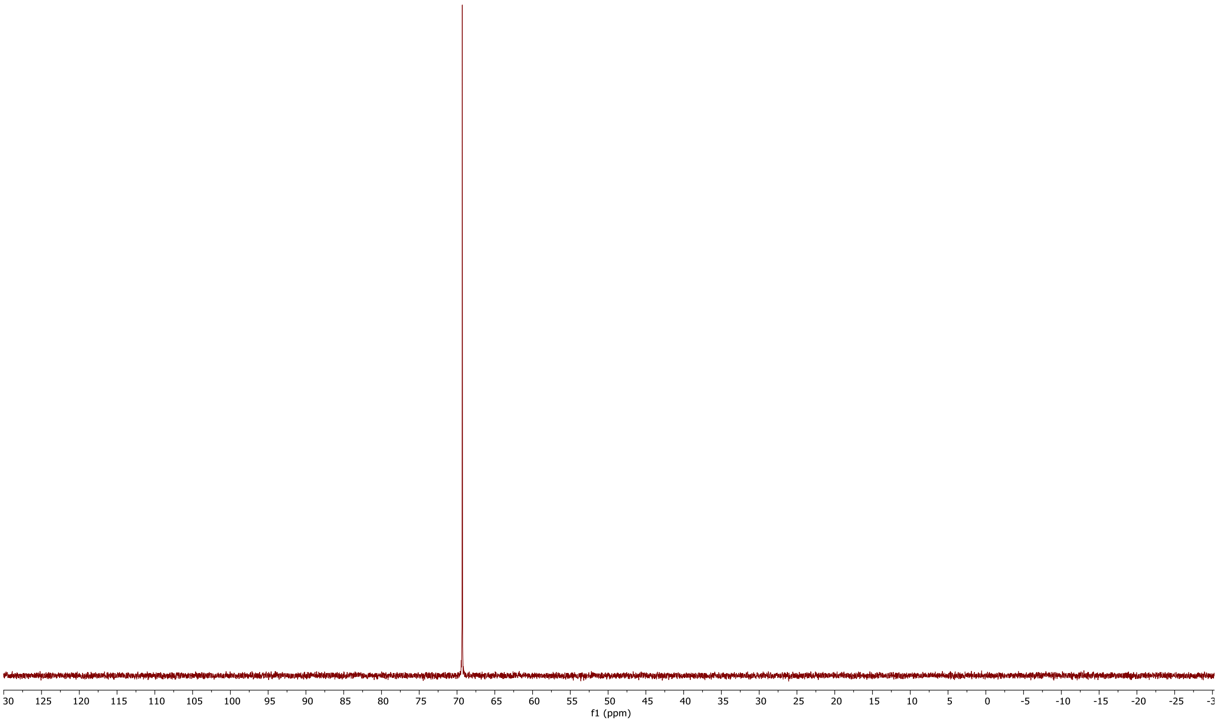

Figure S85. $^{31}P\{^{1}H\}$ NMR spectrum (161.98 MHz, $CDCl_3$) of **12**.

REFERENCES

1 Shriver, D. F.; Drezdzon, M. A. The Manipulation of Air-Sensitive Compounds, 2nd ed., J. Wiley & Sons, New York, **1986**.

2 Chalmers, B. A.; Bühl, M.; Arachchige, K. S. A.; Slawin, A. M. Z.; Kilian, P. Structural, Spectroscopic and Computational Examination of the Dative Interaction in Constrained Phosphine–Stibines and Phosphine–Stiboranes. *Chem. Eur. J.* **2015**, *21*, 7520–7531.

3 Scholz, S.; Scheibitz, M.; Schödel, F.; Bolte, M.; Wagner, M.; Lerner, H. Difference in reactivity of triel halides EX_3 towards ferrocene. *Inorg. Chim. Acta* **2007**, *360*, 3323-3329.

4 Bárta, O.; Císařová, I.; Schulz, J.; Štěpnička, P. Assessing the influence of phosphine substituents on the catalytic properties of self-stabilised digold(I) complexes with supporting ferrocene phosphinonitrile ligands. *New J. Chem*. **2019**, *43*, 11258-11262.

5 Borys, A. M. An Illustrated Guide to Schlenk Line Techniques. *Organometallics* **2023**, *42*, 182-196.

6 Silverstein, R. M.; Webster, F. X.; Kiemle, D. J. Spectrometric Identification of Organic Compounds, 7th Ed., Wiley, New York, **2005**; chapter 3, p. 127 ff.

7 Carrión, M. C.; Torres, J.; Jalón, F. A.; Rodríguez, A. M.; Zirakzadeh, A.; Manzano, B. R. Phosphinofulvene Enolate Ligands in Ruthenium Complexes by Ferrocene Photolysis under Solar Radiation. *Eur. J. Inorg. Chem.* **2017**, 8, 1153-1162.

8 Meng, X.; Kim, S. Tungsten and molybdenum catalyst-mediated cyclisation of *N*-propargyl amides. *Org. Biomol. Chem*. **2011**, *9*, 4429-4431.

9 Sheldrick, G. M. SHELXT – Integrated space-group and crystal-structure determination. *Acta Crystallogr., Sect. A: Found. Adv*. **2015**, *71*, 3-8.

10 Sheldrick, G. M. Crystal Structure Refinement with SHELXL. *Acta Crystallogr., Sect. C: Struct. Chem*. **2015**, *71*, 3-8.

11 Spek, A. L. PLATON SQUEEZE: a tool for the calculation of the disordered solvent contribution to the calculated structure factors. *Acta Crystallogr., Sect. C: Struct. Chem*. **2015**, *71*, 9-18.

12 (a) Spek, A. L. Single-crystal structure validation with the program PLATON. *J. Appl. Crystallogr.* **2003**, *36*, 7-13; (b) Spek, A. L. Structure validation in chemical crystallography. *Acta Crystallogr. D, Biol. Crystallogr*. **2009**, *65*, 148-155.

13 Frisch, M. J.; Trucks, G. W.; Schlegel, H. B.; Scuseria, G. E.; Robb, M. A.; Cheeseman, J. R.; Scalmani, G.; Barone, V.; Petersson, G. A.; Nakatsuji, H.; Li, X.; Caricato, M.; Marenich, A. V.; Bloino, J.; Janesko, B. G.; Gomperts, R.; Mennucci, B.; Hratchian, H. P.; Ortiz, J. V.; Izmaylov, A. F.; Sonnenberg, J. L.; Williams-Young, D.; Ding, F.; Lipparini, F.; Egidi, F.; Goings, J.; Peng, B.; Petrone, A.; Henderson, T.; Ranasinghe, D.; Zakrzewski, V. G.; Gao, J.; Rega, N.; Zheng, G.;

Liang, W.; Hada, M.; Ehara, M.; Toyota, K.; Fukuda, R.; Hasegawa, J.; Ishida, M.; Nakajima, T.; Honda, Y.; Kitao, O.; Nakai, H.; Vreven, T.; Throssell, K.; Montgomery, J. A., Jr.; Peralta, J. E.; Ogliaro, F.; Bearpark, M. J.; Heyd, J. J.; Brothers, E. N.; Kudin, K. N.; Staroverov, V. N.; Keith, T. A.; Kobayashi, R.; Normand, J.; Raghavachari, K.; Rendell, A. P.; Burant, J. C.; Iyengar, S. S.; Tomasi, J.; Cossi, M.; Millam, J. M.; Klene, M.; Adamo, C.; Cammi, R.; Ochterski, J. W.; Martin, R. L.; Morokuma, K.; Farkas, O.; Foresman, J. B.; Fox, D. J. Gaussian 16, Revision C.01; Gaussian Inc.: Wallingford, CT, 2016.

14 (a) Perdew, J. P.; Burke, K.; Ernzerhof, M. Generalized Gradient Approximation Made Simple. *Phys. Rev. Lett.* **1996**, *77*, 3865-3868; (b) Perdew, J. P.; Ernzerhof, M.; Burke, K. Rationale for Mixing Exact Exchange with Density Functional Approximations. *J. Chem. Phys.* **1996**, *105*, 9982-9985; (c) Ernzerhof, M.; Scuseria, G. E. Assessment of the Perdew−Burke−Ernzerhof Exchange-Correlation Functional. *J. Chem. Phys.* **1999**, *110*, 5029-5036. (d) Adamo, C.; Barone, V. Toward Reliable Density Functional Methods without Adjustable Parameters: The PBE0 model. *J. Chem. Phys.* **1999**, *110*, 6158-6170.

15 (a) Weigend, F.; Furche, F.; Ahlrichs, R. Gaussian Basis Sets of Quadruple Zeta Valence Quality for Atoms H−Kr. *J. Chem. Phys.* **2003**, *119*, 12753-12762. (b) Weigend, F.; Ahlrichs, R. Balanced Basis Sets of Split Valence, Triple Zeta Valence and Quadruple Zeta Valence Quality for H to Rn: Design and Assessment of Accuracy. *Phys. Chem. Chem. Phys.* **2005**, *7*, 3297-3305.

16 (a) Dolg, M.; Wedig, U.; Stoll, H.; Preuss, H. Energy-adjusted Ab Initio Pseudopotentials for the First Row Transition Elements. *J. Chem. Phys.* **1987**, *86*, 866-872; (b) Bergner, A.; Dolg, M.; Küchle, W.; Stoll, H.; Preuß, H. Ab Initio Energy-adjusted Pseudopotentials for Elements of Groups 13−17. *Mol. Phys.* **1993**, *80*, 1431-1441; (c) Igel-Mann, G.; Stoll, H.; Preuss, H. Pseudopotentials for Main Group Elements (IIIa through VIIa). *Mol. Phys.* **1988**, *6*, 1321-1328; (d) Andrae, D.; Häußermann, U.; Dolg, M.; Stoll, H.; Preuß, H. Energy-adjusted *ab initio* pseudopotentials for the second and third row transition elements. *Theor. Chim. Acta* **1990**, *77*, 123-141.

17 (a) Becke, A. D.; Johnson, E. R. A density-functional model of the dispersion interaction. *J. Chem. Phys.* **2005**, *123*, 154101; (b) Grimme, S.; Antony, J.; Ehrlich, S.; Krieg, H. A Consistent and Accurate Ab Initio Parametrization of Density Functional Dispersion Correction (DFT-D) for the 94 Elements H-Pu. *J. Chem. Phys.* **2010**, *132*, 154104; (c) Grimme, S.; Ehrlich, S.; Goerigk, L. Effect of the Damping Function in Dispersion Corrected Density Functional Theory. *J. Comput. Chem.* **2011**, *32*, 1456-1465.

18 (a) Tomasi, J. ; Mennucci, B. ; Cammi, R. Quantum Mechanical Continuum Solvation Models. *Chem. Rev.* **2005**, *105*, 2999-3094; (b) Scalmani, G.; Frisch, M. J. Continuous surface charge

polarizable continuum models of solvation. I. General formalism. *J. Chem. Phys.* **2010**, *132*, 114110.

19 Lu, T.; Chen, F. MultiWFN: A Multifunctional Wavefunction Analyzer. *J. Comput. Chem.* **2012**, *33*, 580.

20 Lindner, C.; Maryasin, B.; Richter, F.; Zipse, H. Methyl Cation Affinity (MCA) Values for Phosphanes. *J. Phys. Org. Chem.* **2010**, *23*, 1036-1042.

21 Erdmann, P.; Leitner, J.; Schwarz, J.; Greb, L. An Extensive Set of Accurate Fluoride Ion Affinities for p-Block Element Lewis Acids and Basic Design Principles for Strong Fluoride Ion Acceptors. *ChemPhysChem* **2020**, *21*, 987-994.

22 Zhao, Y.; Truhlar, D. G. Design of Density Functionals That Are Broadly Accurate for Thermochemistry, Thermochemical Kinetics, and Nonbonded Interactions. *J. Phys. Chem. A* **2005**, *109*, 5656-5667.

23 (a) Avogadro: An Open-Source Molecular Builder and Visualization Tool, version 1.2.0, http://avogadro.openmolecules.net/; (b) Hanwell, M. D.; Curtis, D. E.; Lonie, D. C.; Vandermeersch, T.; Zurek, E.; Hutchison, G. R. Avogadro: an Advanced Semantic Chemical Editor, Visualization, and Analysis Platform. *J. Cheminf.* **2012**, *4*, 17.

24 Knizia, G. IboView, version 3, accessible from http://www.iboview.org.
